# Effect of single and combined nitrogen sources on lipid production by *Rhodotorula kratochvilovae* and *Rhodotorula paludigena*

**DOI:** 10.1186/s13568-026-02075-9

**Published:** 2026-07-02

**Authors:** Neveen Hassan Mansour, Ahmad Mohamad Moharram, Zeinab Soliman

**Affiliations:** 1 Department of Botany and Microbiology, Faculty of Science, Assiut University, Assiut, 71515 Egypt; 2 Assiut University Moubasher Centre for Mycological Science, Assiut University, Assiut, 71515 Egypt

**Keywords:** Oleaginous yeast, Lipid yield, Single and combined nitrogen sources

## Abstract

Oleaginous yeasts of the genus *Rhodotorula* are promising microbial platforms for sustainable lipid production. However, nitrogen availability critically regulates both biomass formation and lipid production, with nitrogen limitation recognized as a key trigger for lipid overproduction. This study investigated the influence of single and combined organic and inorganic nitrogen sources on biomass formation and lipid production by *Rhodotorula kratochvilovae* AUMC17237 and *R. paludigena* AUMC17238. The results revealed strain-specific responses to different nitrogen sources. Among individual nitrogen sources, urea supported the highest biomass production in both strains. Ammonium chloride, when combined with organic nitrogen sources, resulted in enhanced biomass formation. Specifically, its combination with yeast extract yielded the highest biomass in *R. kratochvilovae* (6.282 ± 0.161 g/L), whereas its combination with urea produced the strongest biomass response in *R. paludigena* (6.565 ± 0.324 g/L). Organic nitrogen sources generally favored lipid accumulation when applied individually. Urea produced the highest lipid yield in *R. kratochvilovae* (0.990 ± 0.011 g/L), whereas yeast extract was most effective in *R. paludigena* (0.947 ± 0.033 g/L). Furthermore, combined organic-inorganic nitrogen sources enhanced lipid production, with maximum lipid yields achieved using ammonium sulfate with yeast extract in *R. kratochvilovae* (1.219 ± 0.023 g/L) and with urea in *R. paludigena* (0.909 ± 0.050 g/L). Lipid content varied markedly with nitrogen regime, reaching 32.18 ± 1.023 % of cell dry weight in *R. kratochvilovae* and 42.51 ± 1.171% in *R. paludigena*. Overall, these results demonstrate that both nitrogen type and its combination influence biomass formation and lipid accumulation, highlighting the importance of selecting compatible nitrogen regimes to optimize microbial lipid production in applied bioprocesses.

**Supplementary Information:**

The online version contains supplementary material available at 10.1186/s13568-026-02075-9.

## Introduction

The growing demand for sustainable energy and bio-based products has intensified interest in microbial lipids, commonly referred to as single-cell oils (SCOs), as alternatives to fossil-derived resources (Dias et al. 2023; Poontawee et al. 2023). Oleaginous microorganisms, defined by their ability to accumulate more than 20% of their cell dry weight as intracellular lipids, can reach lipid contents of up to 70–80% under nutrient-limited conditions, particularly under nitrogen deprivation (Singh et al. 2024, 2025). These lipids are suitable for biodiesel production and have applications in the food, pharmaceutical, cosmetic, and nutraceutical sectors (Karnwal et al. 2023; Zantioti et al. 2025). Among oleaginous microorganisms, yeasts are especially attractive due to their rapid growth, high lipid productivity, short life cycles, and tolerance to diverse environmental and process conditions (Caporusso et al. 2021; Watsuntorn et al. 2021). Species of the genus *Rhodotorula* (Basidiomycota) have gained increasing attention as promising cell factories for lipid production. They exhibit broad substrate utilization, including agro-industrial wastes, and are suitable for large-scale bioprocessing (Yen et al. 2019; Kot et al. 2020; Mussagy et al. 2022; Angelicola et al. 2023; Kieliszek et al. 2023). In addition, they possess metabolic pathways that support the co-production of value-added compounds such as carotenoids and other terpenoids (Brunel et al. 2024; Ochoa-Viñals et al. 2024; Zhang et al. 2024; Tong et al. 2025). These features make *Rhodotorula* species suitable candidates for integrated biorefinery applications (Byrtusová et al. 2025; Tong et al. 2025). Nitrogen availability is a key regulatory factor in lipid biosynthesis in oleaginous yeasts. While sufficient nitrogen promotes biomass formation, nitrogen limitation redirects carbon flux toward lipid accumulation (Illman et al. 2000; Pal et al. 2011). The type and availability of nitrogen sources significantly affect lipid yield and composition. In addition to nitrogen concentration, the type of nitrogen source plays a crucial role in determining metabolic responses. Oleaginous yeasts can utilize both organic and inorganic nitrogen sources, each affecting lipid metabolism differently. Organic nitrogen sources (e.g., yeast extract, peptone, and urea) generally enhance lipid accumulation, whereas inorganic sources (e.g., ammonium and nitrate salts) tend to favor cell growth (Anschau 2017; Konzock et al. 2022). Consequently, strategic selection and optimization of nitrogen sources are critical for regulating the balance between biomass formation and lipid accumulation, thereby improving overall lipid productivity (Bao et al. 2021; Watsuntorn et al. 2025). Despite extensive studies on individual nitrogen sources, investigations on the combined use of organic and inorganic nitrogen sources remain limited. Such combinations may provide a strategy to decouple the growth and lipid-accumulation phases, potentially improving overall process efficiency. In this context, the present study evaluates the effects of single and combined nitrogen sources on biomass formation and lipid accumulation in *Rhodotorula kratochvilovae* AUMC 17237 and *R. paludigena* AUMC 17238. The study is framed as a preliminary screening under controlled cultivation conditions to identify nitrogen strategies that balance biomass production and lipid accumulation, providing a basis for future optimization.

## Materials and methods

### Isolation of oleaginous yeasts

Oleaginous yeasts were isolated from ten soil samples collected from cultivated soils in Assiut Governorate, Egypt. The isolation process followed an enrichment step in a glycerol-enriched medium, as described by Pan et al. (2009). Subsequently, oleaginous yeasts were isolated using the dilution plate method according to Johnson and Curl (1972).

### Preparation of growth medium

Dichloran rose Bengal yeast extract malt extract agar (DRYM) medium was prepared as described by Wickerham (1951). It contained (g/L): yeast extract, 3.0; malt extract, 3.0; peptone, 5.0; glucose, 10.0; and agar, 20.0. Chloramphenicol (250 µg/ml) was incorporated as a bacteriostatic agent. The medium was further modified by the addition of dichloran (0.2% in ethanol, w/v), 1.0 ml, as described by Moubasher et al. (2016), to restrict the growth of mucoraceous fungi without affecting other fungal species. In addition, rose Bengal (25 µg/ml) was also added to suppress bacterial growth.

### Screening of oleaginous yeasts

#### Using Sudan Black B staining

Yeast strains were preliminarily screened for intracellular lipids using Sudan Black B staining following Burdon et al. (1942). Under oil-immersion phase-contrast microscopy, the cytoplasm appeared pink, while intracellular lipids were stained bluish-gray or bluish-black. Strains exhibiting visible lipid globules were selected for subsequent quantitative analysis.

#### Using Rhodamine B dye

Oleaginous yeasts were identified using Rhodamine B (10 mg/L) incorporated into agar media, with detection based on fluorescence under UV light (365 nm) or color under visible light as described by Niehus et al. (2018). Pigmented yeasts were cultivated on medium containing glucose 20 g/L, peptone 20 g/L, yeast extract 10 g/L, agar 20 g/L. Following incubation at 28 °C for 2 days, oleaginous strains were distinguished by orange fluorescence.

#### Molecular identification of the yeast strains

The strains were identified based on ribosomal DNA sequence analysis. Yeast cultures were grown on YM medium for 2 days at 25 °C, and then the genomic DNA was extracted. The internal transcribed spacer (ITS) region of nuclear rDNA was amplified using the universal primers ITS1 (5′-TCC GTA GGT GAA CCT GCG G-3′) and ITS4 (5′-TCC TCC GCT TAT TGA TAT GC-3′) and sequenced at SolGent Company, Daejeon, South Korea, as described by Moubasher et al. (2016). Contigs were created from the sequence data using DNAstar. The sequence obtained from each strain was further analyzed using BLAST from the National Center for Biotechnology Information (NCBI). The phylogeny analysis was inferred using the Maximum Likelihood method and General Time Reversible model (Nei and Kumar 2000) of nucleotide substitutions. Evolutionary analyses were conducted in MEGA12 (Kumar et al., 2024) utilizing up to 3 parallel computing threads. All sequences were deposited in GenBank, and the corresponding accession numbers are provided.

### Quantitative screening of oleaginous yeasts

#### Inoculum preparation

Selected yeast strains were cultivated on YM agar plates at 28 °C for 48 h. A loopful of each culture was transferred into 50 mL of inoculation medium containing (g/L): glucose 15, peptone 5, malt extract 3, yeast extract 3 in 100 mL Erlenmeyer flasks and incubated at 28 °C, 180 rpm for 48 h to obtain actively growing cells.

#### Flask culture for lipid production

One milliliter of each yeast inoculum was transferred into a 250 mL Erlenmeyer flask containing 99 mL of the lipid production medium (nitrogen-limited medium) with the following composition (g/L): glucose (as carbon source) 40, Na₂HPO₄·12 H₂O 2, KH₂PO₄ 2, MgSO₄·7 H₂O 1.5, CaCl₂ 0.1, ZnSO₄ 0.02, MnSO₄·5 H₂O 0.1, CuSO₄ 0.1, yeast extract 0.5, and peptone 0.3. The flasks were incubated in an incubator shaker at 180 rpm and 28 °C for 5 days (Starkey 1946; Pan et al. 2009). All experiments were performed in triplicate (*n* = 3), and results are expressed as mean ± standard deviation.

### Effect of nitrogen sources on biomass and lipid production by yeasts

Yeasts were cultivated in glucose-based media at a fixed carbon-to-nitrogen (C/N) ratio of 170:1. The organic nitrogen sources included yeast extract, peptone, and urea, while inorganic sources comprised ammonium sulfate and ammonium chloride. Each nitrogen source was evaluated both individually and in defined combinations, comprising organic–inorganic pairings and organic–organic mixtures. The concentration of each nitrogen source was calculated and adjusted to provide an equal equivalent nitrogen level, based on its respective nitrogen fraction (Table 1), ensuring comparability across all treatments. For each experimental condition, 100 mL of nitrogen-limited medium, prepared with the same basal composition with glucose as the sole carbon source and the designated nitrogen source(s), was dispensed into 250 mL Erlenmeyer flasks. Flasks were inoculated with 1% (v/v) yeast culture and incubated in a shaking incubator at 28 °C and 180 rpm for 5 days. All experiments were conducted in biological triplicate (*n* = 3). At the end of the incubation period, cultures were harvested to determine biomass concentration (cell dry weight, g/L) and lipid production parameters, including lipid yield (g/L) and lipid content (%) (Robles-Iglesias et al. 2023).

**Table 1:** Nitrogen-equivalent concentrations (0.092 g/L) of individual and combined nitrogen sources

Nitrogen source	Equivalent amount (g/L)	Nitrogen source	Equivalent amount (g/L)
Yeast extract	0.92	Yeast extract + Urea (1:1)	0.46 + 0.099
Peptone	0.66	Peptone + NH₄Cl (1:1)	0.33 + 0.176
Urea	0.197	Urea + NH₄Cl (1:1)	0.099 + 0.176
Ammonium sulfate	0.434	Yeast extract + NH₄Cl (1:1)	0.46 + 0.176
Ammonium chloride	0.351	Peptone + (NH₄)₂SO₄ (1:1)	0.33 + 0.217
Peptone + Yeast extract (1:1)	0.46 + 0.33	Urea + (NH₄)₂SO₄ (1:1)	0.099 + 0.217
Urea + Peptone (1:1)	0.33 + 0.099	Yeast extract + (NH₄)₂SO₄ (1:1)	0.46 + 0.217

### Biomass recovery and freeze-drying of yeast cells

After incubation, 100 mL of each yeast culture was centrifuged at 3000 rpm for 15 min. The resulting cell pellet was washed once with sterile distilled water and centrifuged again under the same conditions to remove residual medium components. The supernatant was completely removed, and the pellets were transferred into pre-weighed vials designated for lipid extraction. Samples were then stored at − 80 °C before freeze-drying. Samples were freeze-dried for 48–50 h until a constant weight was obtained. Cell dry weight (biomass concentration) was determined gravimetrically (Jiru et al. 2016).

### Disruption of yeast cells

After freeze-drying, the dried biomass was subjected to mechanical disruption and homogenization. Approximately 0.2 g of sterile sand particles was added to the dried cells in the presence of organic solvents (0.66 mL of chloroform and 0.33 mL of methanol). The tubes were then vortexed, using a Vortex Genie-2 for 5 min to ensure thorough cell disruption and homogenization (Goldberg, 2008).

### Lipid extraction and determination of lipid content

Lipid extraction from cell biomass were performed using the method adopted by Folch et al. (1957) with slight modifications (Vasconcelos et al. 2018). Immediately after cell rupture, the contents of each tube were transferred into a 25 mL Falcon tube. Chloroform, methanol, and K₂HPO₄ buffer were then added at a ratio of 2:1:0.6, resulting in final volumes of 9.66 mL chloroform, 4.83 mL methanol, and 2.9 mL buffer. The mixture was thoroughly shaken and subsequently centrifuged at 5000 rpm for 10 min. Samples were then left to stand at room temperature for 2–3 h to allow complete phase separation. The organic chloroform lipid-containing phase forms the lower phase part, and the aqueous methanol phase forms the upper phase. The lower organic phase (chloroform layer) containing lipids was carefully collected into pre-weighed glass bottles. The solvent was evaporated, and the lipid residue was dried in a hot air oven overnight. The total lipids yield was determined gravimetrically, and the percentage of lipid content (Lipid %) was calculated using the following formula:

$$ {\mathrm{Lipid}}\;\left( \% \right) = \left( {{\mathrm{TL}}/{\mathrm{TB}}} \right) \times {\mathrm{1}}00 $$

where TL is the total extracted lipid, and TB is the total biomass used.

### Statistical analysis

Statistical analyses were performed using IBM SPSS Statistics. The individual and interactive effects of yeast species and nitrogen source formulations on biomass production, lipid yield, and intracellular lipid content were evaluated using two-way analysis of variance (ANOVA). In addition, one-way ANOVAs followed by Tukey’s Honestly Significant Difference (HSD) post-hoc tests were conducted within each species to compare nitrogen treatments and identify homogeneous subsets. All statistical tests were two-tailed, and differences were considered statistically significant at *p* < 0.05.

## Results

### Isolation and qualitative screening of oleaginous yeasts

**Fig. 1:**
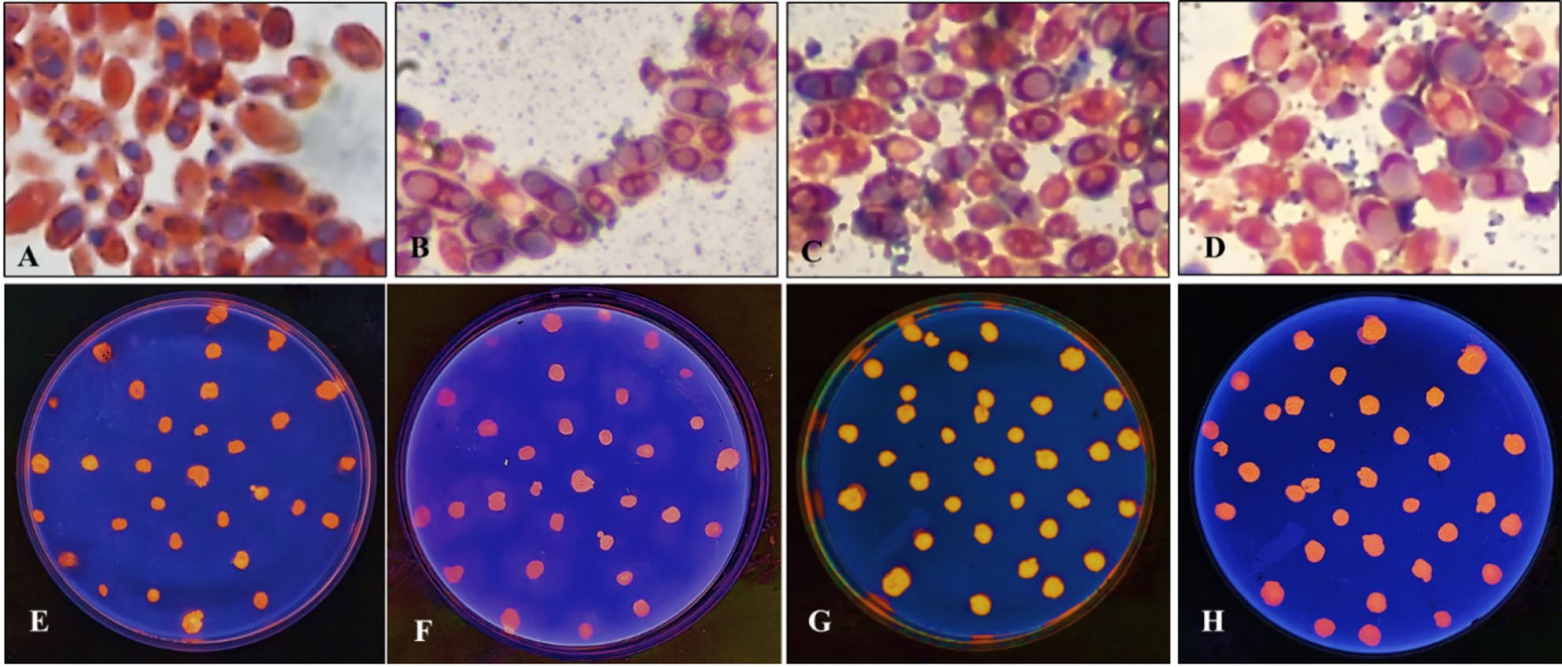
Qualitative screening of oleaginous yeasts. **A**–**D** Sudan Black B-stained cells of *Rhodotorula kratochvilovae* AUMC 17237 (**A**, **B**) and *R. paludigena* AUMC 17238 (**C**, **D**), showing intracellular lipid inclusions under light microscopy. E–H: fluorescence visualization under UV light following cultivation on YPD medium supplemented with 10 mg/L Rhodamine B. **E**, **F**: surface and reverse views of *R. kratochvilovae* cultures; **G**, **H**: surface and reverse views of *R. paludigena* cultures

A total of 104 yeast isolates were recovered from ten enriched soil samples and cultivated on DRYM medium. All isolates were subjected to preliminary qualitative screening for intracellular lipid accumulation using Sudan Black B staining. Several isolates exhibited lipid inclusions; however, two strains, *Rhodotorula kratochvilovae* AUMC 17237 and *R. paludigena* AUMC 17238, showed comparatively larger and more intensely stained intracellular lipid bodies and were therefore selected for further analysis. Rhodamine B dye was subsequently employed as a complementary qualitative assay. Both red-pigmented strains displayed strong orange fluorescence under UV illumination (365 nm), indicating substantial intracellular lipid accumulation. These observations are consistent with the lipid-binding properties of Rhodamine B and support the selection of these strains for quantitative lipid production studies (Fig. 1).

### Molecular identification of the oleaginous yeasts

**Fig. 2:**
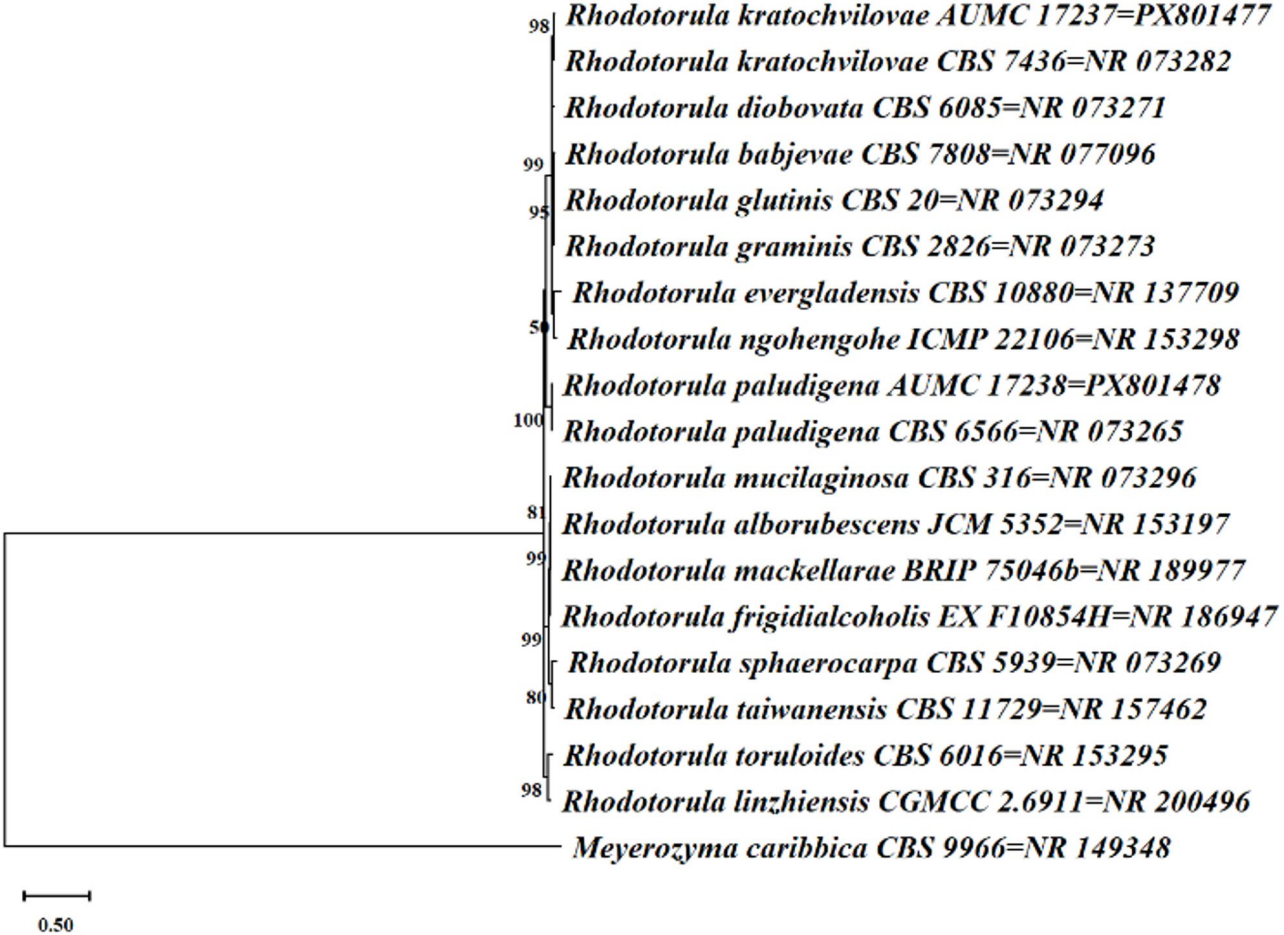
Phylogenetic tree based on ITS rDNA sequences showing the relationships of *Rhodotorula kratochvilovae* AUMC 17237 and *R. paludigena* AUMC 17238 with closely related GenBank reference sequences. The tree was constructed using the Maximum Likelihood method (General Time Reversible model). Bootstrap values (≥ 50%) from 1000 replicates are shown at the nodes. *Meyerozyma caribbica* was used as the outgroup

The two selected strains were identified based on the internal transcribed spacer (ITS) rDNA sequence. Phylogenetic analysis of the ITS region sequences of the two yeast strains, together with their closest GenBank matches, was conducted using the Maximum Likelihood method (Fig. 2). Sequence analysis revealed that the two strains exhibited 99% similarity to their respective type strains. Based on sequence homology and phylogenetic placement, the strains were identified as *Rhodotorula kratochvilovae* and *R. paludigena*. The strains were deposited in the Culture Collection of Assiut University Moubasher Centre for Mycological Science (AUMC), and their ITS gene sequences were submitted to the NCBI GenBank database under accession numbers PX801477 for *Rhodotorula kratochvilovae* AUMC 17237 and PX801478 for *R. paludigena* AUMC 17238.

### Effect of nitrogen sources on biomass production

**Fig. 3:**
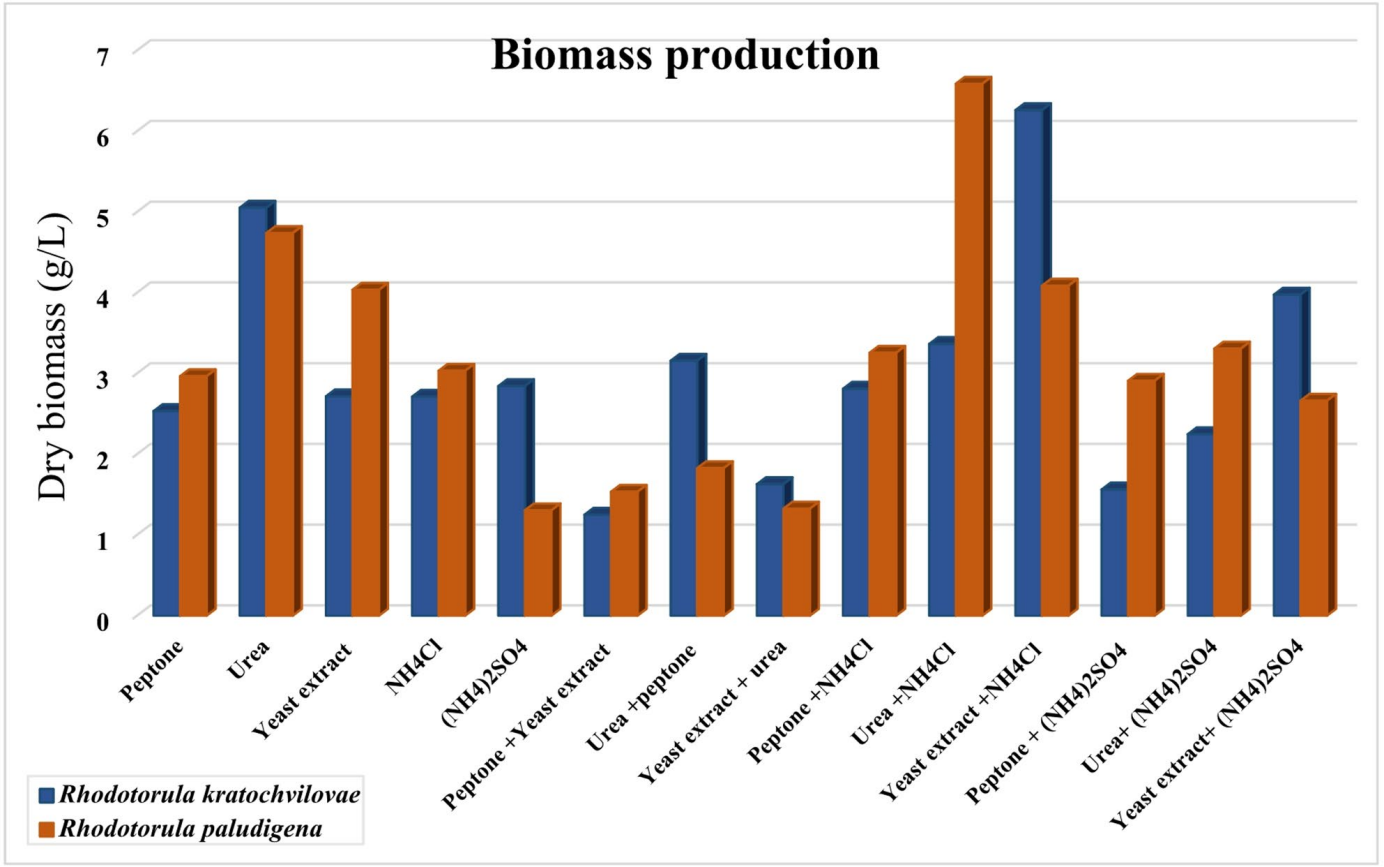
Effect of different single and combined nitrogen sources on biomass formation by *R. kratochvilovae* and *R. paludigena*

This part of the study evaluated the effect of different single and combined nitrogen sources on the biomass of *Rhodotorula kratochvilovae* AUMC17237 and *R. paludigena* AUMC17238, revealing distinct strain-specific growth responses to nitrogen sources (Tables 2 and 3; Fig. 3).

#### In *Rhodotorula kratochvilovae*

Among the single nitrogen sources tested, urea supported the highest biomass production (5.054 ± 0.068 g/L), whereas peptone yielded the lowest biomass (2.503 ± 0.169 g/L). For combined nitrogen sources, the highest biomass (6.282 ± 0.161 g/L) was achieved with the combination of yeast extract and ammonium chloride. In contrast, the yeast extract-peptone combination yielded the lowest biomass (1.264 ± 0.128 g/L). Generally, pairing two organic nitrogen sources consistently resulted in reduced biomass compared to their respective single sources. In contrast, the effect of inorganic nitrogen sources varied with the pairing: ammonium chloride increased biomass when combined with yeast extract or peptone, but decreased biomass when combined with urea. Similarly, ammonium sulfate enhanced biomass in combination with yeast extract but reduced biomass when paired with peptone or urea.

#### In *Rhodotorula paludigena*

With respect to the single nitrogen sources assessed, urea supported the highest biomass production in *Rhodotorula paludigena* (4.634 ± 0.139 g/L), whereas ammonium sulfate yielded the lowest biomass (1.336 ± 0.088 g/L). Among combined nitrogen sources, our results demonstrated that the combination of ammonium chloride with any of the tested organic nitrogen sources enhanced biomass production relative to their individual performances. The mixture of urea and ammonium chloride produced the highest biomass (6.565 ± 0.324 g/L), while the yeast extract-urea combination resulted in the lowest biomass (1.336 ± 0.111 g/L). In contrast, supplementation with ammonium sulfate in combination with the organic nitrogen sources reduced the biomass yield compared to respective single nitrogen sources. As observed for *R. kratochvilovae*, any combination of two organic nitrogen sources led to a decrease in biomass production. Overall, these results indicate that biomass production in *Rhodotorula* species varies with the type and combination of nitrogen sources used.

### Effect of nitrogen sources on lipid yield

#### In *Rhodotorula kratochvilovae*

As shown in Table 2, among the single nitrogen sources, urea resulted in the highest lipid yield (0.990 ± 0.011 g/L), followed by yeast extract (0.698 ± 0.034 g/L), whereas ammonium chloride produced the lowest yield (0.374 ± 0.021 g/L). When applied individually, inorganic nitrogen sources generally produced lower lipid yields than the organic nitrogen sources tested. For the combined nitrogen sources, combinations of nitrogen sources (organic or inorganic) generally resulted in lower lipid yields than the corresponding single nitrogen sources, except for the ammonium sulfate and yeast extract combination, which produced the highest lipid yield (1.219 ± 0.023 g/L). 

#### In *Rhodotorula paludigena*

From the single nitrogen sources, yeast extract produced the highest lipid yield (0.947 ± 0.033 g/L) followed by peptone (0.888 ± 0.040 g/L), whereas ammonium chloride resulted in the lowest (0.306 ± 0.007 g/L). Among combined nitrogen sources, urea combined with ammonium sulfate achieved the highest lipid yield (0.909 ± 0.050 g/L), while its combination with ammonium chloride yielded the lowest (0.361 ± 0.039 g/L). Yeast extract and peptone, as individual organic nitrogen sources, achieved the highest lipid yields. However, their combination with other organic or inorganic nitrogen sources led to lower lipid production (Table 3).

**Fig. 4:**
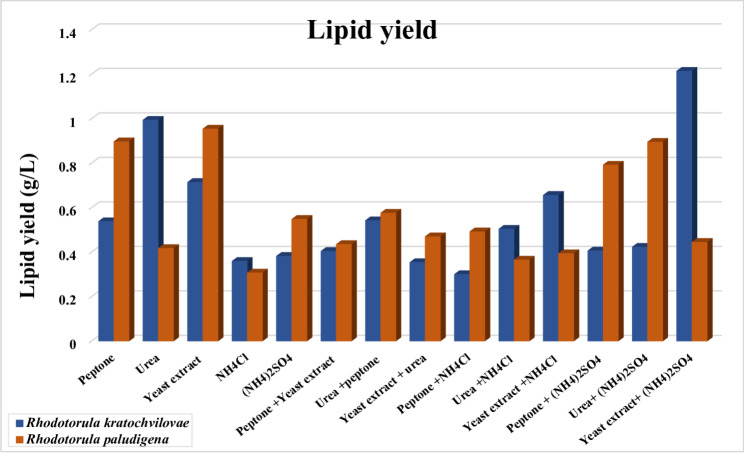
Effect of different single and combined nitrogen sources on lipid yield by *R. kratochvilovae* and *R. paludigena*

Overall, lipid yield varied according to the type and combination of nitrogen sources applied. Organic nitrogen sources generally supported higher lipid yields than inorganic sources when used individually. However, only specific combinations, ammonium sulfate with yeast extract in *R. kratochvilovae* and with urea in *R. paludigena*, resulted in comparatively higher lipid yields. Other combinations, particularly those involving two organic nitrogen sources, were associated with reduced lipid production (Tables 2 and 3; Fig. 4).

### Effect of nitrogen sources on lipid content

#### In *Rhodotorula kratochvilovae*

Considering the individual nitrogen source, the highest lipid content of *Rhodotorula kratochvilovae* was achieved with yeast extract (26.87 ± 0.511%), followed by peptone (21.49 ± 2.822%). The combination of yeast extract and peptone resulted in the highest lipid content (32.18 ± 1.023%), followed by the combinations of ammonium sulfate with yeast extract or peptone (31.49 ± 0.838% and 26.04 ± 0.257%, respectively). In contrast, the lowest lipid contents were recorded with ammonium chloride as a single source (13.73 ± 0.429%), and its combination with yeast extract (10.09 ± 0.362%) (Table 2).

#### In *Rhodotorula paludigena*

The lipid content varied considerably depending on the nitrogen source applied, as shown in Table 3. Among the single nitrogen sources tested, *Rhodotorula paludigena* exhibited the highest lipid content when cultivated with ammonium sulfate (42.51 ± 1.171%), followed by peptone (30.59 ± 1.347%), whereas urea resulted in the lowest value (9.64 ± 1.269%). Regarding combined nitrogen sources, the highest lipid content was obtained with yeast extract-urea mixture (35.59 ± 2.127%), followed by the peptone-urea combination (31.10 ± 2.063%). Conversely, pairing of ammonium chloride with urea led to the lowest lipid content (5.49 ± 0.323%). Combinations involving ammonium chloride were generally associated with lower lipid content.

**Fig. 5:**
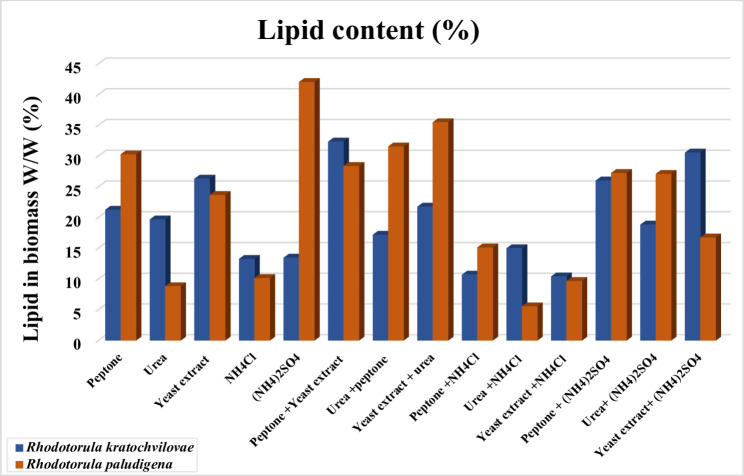
Lipid content of *Rhodotorula kratochvilovae* and *R. paludigena* on different single and combined nitrogen sources

Overall, lipid content in both *Rhodotorula* strains was influenced by the type and combination of nitrogen sources. The response patterns differed between strains, with *R. kratochvilovae* favoring organic nitrogen sources and *R. paludigena* showing higher lipid accumulation with (NH₄)₂SO₄ as a single source. Combined nitrogen sources produced variable effects, depending on the specific pairing (Tables 2 and 3; Fig. 5).

**Table 2:** Effect of different single and combined nitrogen sources on biomass formation and lipid production by the oleaginous yeast *Rhodotorula kratochvilovae* AUMC 17237

Nitrogen source	Biomass (g/L)	Lipid yield (g/L)	Lipid content (% w/w)
Peptone	2.503 ± 0.169^fg^	0.535 ± 0.034^ef^	21.49 ± 2.822^c^
Urea	5.054 ± 0.068^b^	0.990 ± 0.011^b^	19.58 ± 0.098^cd^
Yeast extract	2.600 ± 0.149^fg^	0.698 ± 0.034^c^	26.87 ± 0.511^b^
NH₄Cl	2.726 ± 0.122^f^	0.374 ± 0.021^hi^	13.73 ± 0.429^efg^
(NH₄)₂SO₄	2.769 ± 0.084^f^	0.379 ± 0.020^hi^	14.03 ± 0.518^ef^
Peptone + Yeast extract	1.264 ± 0.128^i^	0.406 ± 0.028 g^hi^	32.18 ± 1.023^a^
Urea + Peptone	3.145 ± 0.096^de^	0.547 ± 0.026^de^	17.39 ± 0.339^de^
Yeast extract + Urea	1.653 ± 0.112^h^	0.352 ± 0.006^hi^	21.34 ± 1.093^c^
Peptone + NH₄Cl	2.814 ± 0.161^ef^	0.312 ± 0.051^i^	11.06 ± 1.162^fg^
Urea + NH₄Cl	3.384 ± 0.151^d^	0.492 ± 0.062^efg^	14.51 ± 1.236^ef^
Yeast extract + NH₄Cl	6.282 ± 0.161^a^	0.634 ± 0.030^cd^	10.09 ± 0.362^g^
Peptone + (NH₄)₂SO₄	1.559 ± 0.090^hi^	0.406 ± 0.020^ghi^	26.04 ± 0.257^b^
Urea + (NH₄)₂SO₄	2.246 ± 0.111^g^	0.445 ± 0.038^fgh^	19.88 ± 2.664 cd
Yeast extract + (NH₄)₂SO₄	3.872 ± 0.106^c^	1.219 ± 0.023^a^	31.49 ± 0.838a

Values represent the mean ± standard deviation of three independent replicates (n = 3). Different superscript alphabetical letters within the same column indicate statistically significant differences among nitrogen source formulations for *Rhodotorula kratochvilovae*, according to one-way ANOVA followed by Tukey’s HSD post-hoc test at p < 0.05. Mean values sharing at least one common letter within the same column are not significantly different.

**Table 3:** Effect of different single and combined nitrogen sources on biomass formation and lipid production by the oleaginous yeast *Rhodotorula paludigena* AUMC17238

Nitrogen source	Biomass (g/L)	Lipid yield (g/L)	Lipid content (% w/w)
Peptone	2.802 ± 0.203^e^	0.888 ± 0.040^a^	30.59 ± 1.347^bc^
Urea	4.634 ± 0.139^b^	0.446 ± 0.045^ef^	9.64 ± 1.269^hi^
Yeast extract	4.127 ± 0.089^c^	0.947 ± 0.033^a^	22.97 ± 1.140^ef^
NH₄Cl	3.058 ± 0.028^de^	0.306 ± 0.007^g^	9.99 ± 0.202^h^
(NH₄)₂SO₄	1.336 ± 0.088^g^	0.567 ± 0.027^cd^	42.51 ± 1.171^a^
Peptone + Yeast extract	1.615 ± 0.130^fg^	0.443 ± 0.019^ef^	27.48 ± 1.028^bcd^
Urea + Peptone	1.868 ± 0.106^f^	0.580 ± 0.008^c^	31.10 ± 2.063^bc^
Yeast extract + Urea	1.336 ± 0.111^g^	0.474 ± 0.012^de^	35.59 ± 2.127^b^
Peptone + NH₄Cl	3.462 ± 0.206^d^	0.467 ± 0.030^e^	13.57 ± 1.647^gh^
Urea + NH₄Cl	6.565 ± 0.324^a^	0.361 ± 0.039^fg^	5.49 ± 0.323^i^
Yeast extract + NH₄Cl	4.147 ± 0.077^bc^	0.401 ± 0.017^ef^	9.67 ± 0.252^hi^
Peptone + (NH₄)₂SO₄	2.929 ± 0.161^e^	0.789 ± 0.023^b^	26.91 ± 0.794^cde^
Urea + (NH₄)₂SO₄	3.468 ± 0.257^d^	0.909 ± 0.050^a^	26.23 ± 0.741^de^
Yeast extract + (NH₄)₂SO₄	2.661 ± 0.105^e^	0.451 ± 0.045^ef^	16.91 ± 1.018^fg^

*Values represent the mean ± standard deviation of three independent replicates (n = 3). Different superscript letters (a–i) within the same column indicate statistically significant differences between the nitrogen source formulations according to a One-Way Analysis of Variance (ANOVA) followed by Tukey’s Honestly Significant Difference (HSD) post-hoc test (*p* < 0.05). Letters are assigned sequentially in descending order of the mean values: the letter ‘a’ denotes the highest statistically significant subset, progressing down to ‘i’, which denotes the lowest statistically significant subset. Mean values sharing at least one common letter (e.g., ‘bc’ or ‘def’) are not significantly different from one another.

### Statistical evaluation of nitrogen source effects

Two-way analysis of variance (ANOVA) revealed that yeast species, nitrogen source, and their interaction significantly affected biomass production, lipid yield, and lipid content (*p* < 0.05). The significant interaction between species and nitrogen source indicated that the response to nitrogen supplementation was species-dependent. Nitrogen source exerted the strongest effect on all measured parameters, as reflected by the high effect sizes (partial η² = 0.974–0.990). Furthermore, the high coefficients of determination (R² = 0.985–0.992) demonstrated that the experimental model explained most of the observed variation. Therefore, subsequent analyses were conducted separately for each species using one-way ANOVA followed by Tukey’s Honestly Significant Difference (HSD) test to determine significant differences among nitrogen treatments (Table 4).

Biomass production and intracellular lipid accumulation were strongly influenced by nitrogen source composition, with distinct responses observed between the two yeast strains (Tables 2 and 3). In both strains, treatments containing ammonium chloride generally promoted higher biomass accumulation, suggesting a stimulatory effect on cellular proliferation. In *Rhodotorula kratochvilovae*, the combination of yeast extract and ammonium chloride produced the highest biomass (6.282 ± 0.161 g/L), significantly exceeding all other treatments (*p* < 0.05). However, this treatment resulted in the lowest lipid content (10.09 ± 0.362%), suggesting an inverse relationship between biomass formation and intracellular lipid accumulation. In contrast, the combination of peptone and yeast extract produced the highest lipid content in this strain (32.18 ± 1.023%) despite lower biomass production. A similar trend was observed in *Rhodotorula paludigena*, where the combination of urea and ammonium chloride yielded the highest biomass (6.565 ± 0.324 g/L), significantly higher than the remaining nitrogen treatments (*p* < 0.05), but it also resulted in the lowest lipid content (5.49 ± 0.323%). Conversely, ammonium sulfate supplementation produced the highest lipid content (42.51 ± 1.171%) while supporting comparatively lower biomass production. These findings demonstrate a clear tradeoff between cellular proliferation and lipid accumulation under different nitrogen regimes.

Lipid yield reflected the combined effects of biomass formation and intracellular lipid content and therefore differed from treatments maximizing either parameter individually. *R. kratochvilovae*, the combination of yeast extract and ammonium sulfate produced the highest lipid yield (1.219 ± 0.023 g/L) together with relatively high lipid content (31.49 ± 0.838%), suggesting a favorable balance between growth and lipid biosynthesis. In contrast, the combination of peptone and yeast extract produced the highest lipid content (32.18 ± 1.023%) but comparatively lower lipid yield (0.406 ± 0.028 g/L), indicating limited biomass formation under these conditions. In *R. paludigena*, ammonium sulfate produced the highest lipid content (42.51 ± 1.171%) but only moderate lipid yield (0.567 ± 0.027 g/L), whereas yeast extract resulted in the highest lipid yield (0.947 ± 0.033 g/L) despite lower lipid content (22.97 ± 1.140%). These differences were statistically significant according to Tukey’s HSD test (*p* < 0.05).

Collectively, the findings demonstrate that high lipid content does not necessarily correspond to high volumetric lipid yield, highlighting the importance of balancing biomass formation and lipid biosynthesis during SCO production. The results further suggest that different nitrogen management strategies may be required for each strain. Under the cultivation conditions employed in this study, the combination of yeast extract and ammonium sulfate appeared suitable for balanced lipid production in *R. kratochvilovae*, whereas a two-stage cultivation strategy may be more appropriate for *R. paludigena*, involving an initial biomass-production phase followed by lipid induction under alternative nitrogen conditions.

**Table 4:** Two-way ANOVA showing the effects of yeast species, nitrogen source, and their interaction on biomass production, lipid yield, and lipid content

Dependent variable	Source	df	F value	*p* value	Partial η²
Biomass	Species	1	23.979	< 0.001	0.300
	Nitrogen sources	13	417.691	< 0.001	0.990
	Species× Nitrogen sources	13	144.400	< 0.001	0.971
	Model statistics	27,56	271.524	< 0.001	R² = 0.992
Lipid yield	Species	1	6.173	0.016	0.099
	Nitrogen sources	13	160.483	< 0.001	0.974
	Species× Nitrogen sources	13	180.752	< 0.001	0.977
	Model statistics	27,56	164.527	< 0.001	R² = 0.988
Lipid content	Species	1	51.419	< 0.001	0.479
	Nitrogen sources	13	177.510	< 0.001	0.976
	Species× Nitrogen sources	13	105.050	< 0.001	0.961
	Model statistics	27,56	137.952	< 0.001	R² = 0.985

## Discussion

The two oleaginous strains investigated in this study, *Rhodotorula kratochvilovae* and *R. paludigena*, exhibited pronounced variability in biomass production, lipid yield, and intracellular lipid content under different nitrogen regimes, reflecting their metabolic flexibility and oleaginous potential. *R. kratochvilovae* achieved its maximum biomass (6.282 ± 0.161 g/L) and lipid yield (1.219 ± 0.023 g/L), with a lipid content of 32.18 ± 1.023%. In contrast, *R. paludigena* reached a slightly higher biomass (6.565 ± 0.324 g/L) but a lower lipid yield (0.947 ± 0.033 g/L), despite exhibiting the highest lipid content (42.51 ± 1.171%). Across all treatments, biomass, lipid yield, and lipid content varied considerably in both strains, these variations underscore the decisive role of nutritional conditions in regulating lipid accumulation in both species. Rather than reflecting simple growth differences, the observed patterns suggest differential carbon partitioning driven by nitrogen availability and source composition. These findings are consistent with Szotkowski et al. (2021) and Ye et al. (2021), who demonstrated that substantial variations in biomass production and lipid yield occur within the same strain depending on the nature of the carbon and nitrogen sources used during cultivation.

The present results demonstrate that urea is the most effective single nitrogen source for promoting biomass and lipid production in *Rhodotorula kratochvilovae*, yielding a maximum biomass of 5.054 ± 0.068 g/L and lipid yield of 0.990 ± 0.011 g/L. A similar trend was observed in *R. paludigena*, which also achieved its highest biomass (4.634 ± 0.139 g/L) under urea supplementation, indicating a consistent growth-promoting effect across both strains, although its influence on lipid accumulation appeared strain-dependent. This enhancement may be attributed to the efficient assimilation of urea, which is hydrolyzed intracellularly via urease and imposes a lower energetic cost compared to ammonium salts (Konzock et al. 2022). Consequently, reduced ATP expenditure for nitrogen assimilation likely improves overall metabolic efficiency, supporting increased biomass formation while maintaining intracellular conditions favorable for lipid biosynthesis, which is strongly regulated by nitrogen availability and carbon flux redistribution in oleaginous yeasts (Szczepańska et al. 2022; Kumar et al. 2025). These findings are consistent with previous reports demonstrating improved growth in urea-based media, for *Geotrichum fermentans*, *Rhodotorula kratochvilovae*, and *R. diobovata* (Zhu et al. 2008; Szotkowski et al. 2021; Osman et al. 2022), as well as broader observations that urea promotes lipid accumulation across oleaginous yeasts (Enshaeieh et al. 2012). Similar effects were also reported in *Yarrowia lipolytica*, where substitution of ammonium sulfate with urea significantly enhanced growth performance (Brabender et al. 2018). This improvement has been attributed to the lower energetic cost of urea assimilation, requiring substantially less ATP per unit of nitrogen compared to ammonium-based sources, which ultimately influences biomass formation, lipid biosynthesis, and glucose uptake rates. (Konzock et al. 2022). This mechanistic framework is consistent with the present results, where biomass of *R. kratochvilovae* increased from 2.769 ± 0.084 g/L with ammonium sulfate to 5.054 ± 0.068 g/L with urea, while *R. paludigena* exhibited a comparable increase from 1.336 ± 0.088 g/L to 4.634 ± 0.139 g/L under the same conditions.

Yeast extract supported the highest lipid yield in *Rhodotorula paludigena* (0.947 ± 0.033 g/L), indicating its effectiveness as a nitrogen source for lipid accumulation. This observation is consistent with previous studies identifying yeast extract as an efficient nitrogen source for lipid production in various oleaginous yeasts (Dyal and Narine 2005). Furthermore, numerous reports indicated that yeast extract, when used as a single nitrogen source, supported the maximal lipid yield in *Rhodotorula glutinis*, *Rhodosporidium toruloides*, *Lipomyces starkeyi*, *Rhodotorula mucilaginosa*, *Trichosporonoides spathulata*, *Kodamaea ohmeri*, *Yarrowia lipolytica*, and *Cutaneotrichosporon oleaginosus* (Kumar et al. 2010; Li et al. 2010; Kitcha and Cheirsilp 2011; Bellou et al. 2016; Awad et al. 2019). The superior performance of yeast extract is likely attributable to its rich nutritional composition of amino acids, vitamins, nucleosides, polypeptides, and minerals, which support robust growth and lipid biosynthesis (Huynh et al. 2020).

Interestingly, ammonium sulfate resulted in the highest cellular lipid content (42.51 ± 1.171%) in *Rhodotorula paludigena*, suggesting that inorganic nitrogen sources may preferentially promote lipid accumulation under specific metabolic conditions. The superiority of ammonium sulfate over urea in *R. paludigena* underscores strain-specific responses to nitrogen sources and the importance of empirical optimization for each strain. This observation is consistent with Probst (2016), who reported NH₄⁺ as an effective nitrogen source, and supported by Madani et al. (2017), who identified ammonium sulfate as more favorable for lipid production. Similarly, Taki et al. (2025) demonstrated that ammonium sulfate supplementation significantly enhanced lipid production in *Lipomyces starkeyi*, attributing this improvement to stimulation of glutathione-related metabolism and enhanced ammonia assimilation, which promoted triacylglycerol (TAG) synthesis.

In the present study, peptone ranked second in lipid production in both *Rhodotorula kratochvilovae* and *R. paludigena*. This trend is consistent with previous reports showing that peptone exhibited slightly lower yet comparable performance to yeast extract in enhancing lipid content in *Rhodotorula glutinis*, *Rhodosporidium toruloides*, *Lipomyces starkeyi*, and *Trichosporonoides spathulata* (Kumar et al. 2010; Kitcha and Cheirsilp 2011). In contrast, Zhu et al. (2008) and Elfeky et al. (2019) identified peptone as the most effective nitrogen source for maximizing lipid accumulation in *Geotrichum fermentans* and *Rhodotorula glutinis*, respectively. These discrepancies highlight the strong species- and strain-specific variability in nitrogen metabolism and its impact on carbon allocation between biomass formation and lipid storage. Overall, these results emphasize that the optimal nitrogen source cannot be generalized across oleaginous yeasts and must be determined empirically for each strain, as differences in nitrogen assimilation pathways and metabolic regulation ultimately govern lipid production efficiency.

Among the combined organic nitrogen sources, the mixture of peptone and urea achieved relatively higher biomass and lipid production in both yeast strains compared to the other organic combinations. However, lipid content responses were strain-specific: the highest lipid content in *Rhodotorula paludigena* (35.59 ± 2.127%) was obtained by the addition of yeast extract to urea, whereas the combination of yeast extract and peptone resulted in the maximum lipid content (31.10 ± 2.063%) in *R. kratochvilovae*. These results suggest that combining nitrogen sources can enhance both growth and lipid accumulation by providing a balanced supply of readily assimilable nitrogen and essential growth factors. This interpretation is consistent with previous findings that a mixture of yeast extract and peptone can enhance lipid production in oleaginous yeasts, including *Kodamaea ohmeri* and *Trichosporonoides spathulata* (Kitcha and Cheirsilp 2011).

Regarding lipid yield, the combination of ammonium sulfate and yeast extract produced the highest lipid yield in *R. kratochvilovae* (1.219 ± 0.023 g/L), whereas its combination with urea yielded the maximum lipid production in *R. paludigena* (0.909 ± 0.050 g/L). These results demonstrate that each strain responds optimally to a distinct organic–inorganic nitrogen combination, reflecting differences in nitrogen assimilation and metabolic regulation. These strain-specific responses underscore the need for tailored nitrogen supplementation strategies to maximize lipid productivity in oleaginous yeasts. These findings are consistent with previous studies reporting improved lipid production under combined nitrogen supplementation. For instance, Enshaeieh et al. (2012) showed that yeast extract combined with ammonium sulfate significantly enhanced lipid synthesis in *Rhodotorula* sp. and *Candida* sp., while Jiru et al. (2017) reported similar improvements in *Rhodotorula kratochvilovae*. In addition, Bardhan et al. (2021) identified yeast extract and ammonium sulfate among the most influential medium components for enhancing biomass and lipid accumulation under batch conditions. The superior performance of mixed nitrogen sources likely arises from the complementary roles of organic and inorganic components. Organic sources provide essential amino acids, vitamins, and cofactors, while inorganic nitrogen sources contribute readily assimilable nitrogen, together enhancing metabolic efficiency and promoting carbon flux toward lipid biosynthesis.

Importantly, lipid yield alone is inadequate as the sole criterion when selecting a nitrogen source for high lipid production, as other parameters, including biomass production and cellular lipid content, are equally important. Although urea promoted high biomass and lipid yield in *R. kratochvilovae*, lipid content remained relatively low under these conditions. Conversely, combinations yielding higher lipid content often resulted in reduced biomass, highlighting a trade-off between cellular growth and lipid accumulation. These results demonstrate that achieving an optimal balance between biomass production and lipid accumulation requires carefully tailored nitrogen strategies rather than reliance on a single nitrogen source.

From an economic perspective, the effectiveness of urea is particularly significant, given its wide availability, low cost, and established use in industrial and agricultural applications. In this study, urea consistently supported high biomass formation in both strains and yielded the highest lipid production in *R. kratochvilovae*. In *R. paludigena*, its combination with ammonium sulfate further enhanced lipid production, suggesting that urea-based nitrogen strategies can be effectively integrated with inorganic sources to improve overall process performance. Such approaches offer a cost-effective and scalable approach for microbial lipid production, particularly in fermentation-based systems where both productivity and economic feasibility are critical.

## Conclusion

This study demonstrated that both the type and combination of nitrogen sources significantly influenced biomass formation and lipid accumulation in the oleaginous yeasts *Rhodotorula kratochvilovae* and *R. paludigena* under flask-scale cultivation conditions. Among the individual nitrogen sources evaluated, urea supported the highest biomass production in both strains, whereas organic nitrogen sources generally promoted greater lipid accumulation. Importantly, the combination of organic and inorganic nitrogen sources enhanced overall performance in a strain-dependent manner. *Rhodotorula kratochvilovae* achieved optimal biomass and lipid yield when yeast extract was combined with ammonium chloride and ammonium sulfate, respectively, whereas *R. paludigena* showed improved biomass and lipid yield when urea was paired with the same inorganic nitrogen sources. These species-specific responses underscore the importance of optimizing nitrogen regimes to achieve an appropriate balance between cellular growth and lipid biosynthesis.

The present study was performed at laboratory scale and evaluated only two yeast species and a selected set of nitrogen sources; therefore, the results are restricted to these experimental conditions and require validation under controlled bioreactor and scale-up systems before potential industrial application can be considered.

### Future work

Future experiments could evaluate a two-stage cultivation strategy for the most promising yeast strain, in which an initial growth phase employs the nitrogen source that maximizes biomass production, followed by a second lipid-induction phase using the nitrogen source that optimizes lipid accumulation. Such an approach may further enhance overall lipid productivity and improve process efficiency at scale.

## Supplementary Information

Below is the link to the electronic supplementary material.

Supplementary Material 1.

## Data Availability

All data generated or analyzed during this study are included in this article and the accompanying submission files. The uploaded file “Final Statistical Results” contains the complete statistical analyses, including ANOVA and Tukey’s HSD outputs, supporting the findings of this study. The ITS sequences of the yeast strains have been deposited in GenBank under accession numbers PX801477 (Rhodotorula kratochvilovae AUMC 17237) and PX801478 (R. paludigena AUMC 17238).
