# Supplementary material for: Effect of single and combined nitrogen sources on lipid production by Rhodotorula kratochvilovae and Rhodotorula paludigena
Source: AMB Express. 2026 Jul 2;16:73. doi: 10.1186/s13568-026-02075-9 (PMC13337978; doi:10.1186/s13568-026-02075-9)
Supplement: Supplementary file 1 — Supplementary Material 1. [file 13568_2026_2075_MOESM1_ESM.doc]

UNIANOVA Bio BY Species NitroS
  /METHOD=SSTYPE(3)
  /INTERCEPT=INCLUDE
  /POSTHOC=Species NitroS(TUKEY)
  /PLOT=PROFILE(Species*NitroS) TYPE=LINE ERRORBAR=CI MEANREFERENCE=NO YAXIS=AUTO
  /EMMEANS=TABLES(OVERALL)
  /EMMEANS=TABLES(Species) COMPARE ADJ(LSD)
  /EMMEANS=TABLES(NitroS) COMPARE ADJ(LSD)
  /EMMEANS=TABLES(Species*NitroS)
  /PRINT ETASQ DESCRIPTIVE HOMOGENEITY
  /CRITERIA=ALPHA(.05)
  /DESIGN=Species NitroS Species*NitroS.


Univariate Analysis of Variance


Notes	
Output Created	10-MAY-2026 19:36:10	
Comments		
Input	Active Dataset	DataSet5	
	Filter	<none>	
	Weight	<none>	
	Split File	Species	
	N of Rows in Working Data File	84	
Missing Value Handling	Definition of Missing	User-defined missing values are treated as missing.	
	Cases Used	Statistics are based on all cases with valid data for all variables in the model.	
Syntax	UNIANOVA Bio BY Species NitroS
  /METHOD=SSTYPE(3)
  /INTERCEPT=INCLUDE
  /POSTHOC=Species NitroS(TUKEY)
  /PLOT=PROFILE(Species*NitroS) TYPE=LINE ERRORBAR=CI MEANREFERENCE=NO YAXIS=AUTO
  /EMMEANS=TABLES(OVERALL)
  /EMMEANS=TABLES(Species) COMPARE ADJ(LSD)
  /EMMEANS=TABLES(NitroS) COMPARE ADJ(LSD)
  /EMMEANS=TABLES(Species*NitroS)
  /PRINT ETASQ DESCRIPTIVE HOMOGENEITY
  /CRITERIA=ALPHA(.05)
  /DESIGN=Species NitroS Species*NitroS.	
Resources	Processor Time	00:00:00.66	
	Elapsed Time	00:00:00.72	


Warnings	
Post hoc tests are not performed for Species in split file Species=RK because there are fewer than three groups.	
Post hoc tests are not performed for Species in split file Species=RP because there are fewer than three groups.	


Species = RK


Between-Subjects Factorsa	
	Value Label	N	
Species	1	RK	42	
NitroS	1	Pep	3	
	2	Urea	3	
	3	YeEx	3	
	4	NCl	3	
	5	NS	3	
	6	Pep+YeEx	3	
	7	Urea+Pep	3	
	8	YeEx+Urea	3	
	9	Pep+NCl	3	
	10	Urea+NCl	3	
	11	YeEx+NCl	3	
	12	Pep+NS	3	
	13	Urea+NS	3	
	14	YeEx+NS	3	

a. Species = RK	


Descriptive Statisticsa	
Dependent Variable:   Bio  	
Species	NitroS	Mean	Std. Deviation	N	
RK	Pep	2.50267	.168560	3	
	Urea	5.05400	.068022	3	
	YeEx	2.59967	.148796	3	
	NCl	2.72600	.121606	3	
	NS	2.76867	.084347	3	
	Pep+YeEx	1.23833	.089075	3	
	Urea+Pep	3.14467	.096095	3	
	YeEx+Urea	1.65333	.112167	3	
	Pep+NCl	2.81400	.161037	3	
	Urea+NCl	3.38367	.150869	3	
	YeEx+NCl	6.28200	.161130	3	
	Pep+NS	1.55867	.090046	3	
	Urea+NS	2.24567	.111006	3	
	YeEx+NS	3.87167	.106039	3	
	Total	2.98879	1.330495	42	
Total	Pep	2.50267	.168560	3	
	Urea	5.05400	.068022	3	
	YeEx	2.59967	.148796	3	
	NCl	2.72600	.121606	3	
	NS	2.76867	.084347	3	
	Pep+YeEx	1.23833	.089075	3	
	Urea+Pep	3.14467	.096095	3	
	YeEx+Urea	1.65333	.112167	3	
	Pep+NCl	2.81400	.161037	3	
	Urea+NCl	3.38367	.150869	3	
	YeEx+NCl	6.28200	.161130	3	
	Pep+NS	1.55867	.090046	3	
	Urea+NS	2.24567	.111006	3	
	YeEx+NS	3.87167	.106039	3	
	Total	2.98879	1.330495	42	

a. Species = RK	


Levene's Test of Equality of Error Variancesa,b,c	
	Levene Statistic	df1	df2	Sig.	
Bio	Based on Mean	.454	13	28	.933	
	Based on Median	.238	13	28	.996	
	Based on Median and with adjusted df	.238	13	20.999	.995	
	Based on trimmed mean	.438	13	28	.940	

Tests the null hypothesis that the error variance of the dependent variable is equal across groups.a,b,c	
a. Species = RK	
b. Dependent variable: Bio	
c. Design: Intercept + Species + NitroS + Species * NitroS	


Tests of Between-Subjects Effectsa	
Dependent Variable:   Bio  	
Source	Type III Sum of Squares	df	Mean Square	F	Sig.	Partial Eta Squared	
Corrected Model	72.153b	13	5.550	364.520	.000	.994	
Intercept	375.179	1	375.179	24640.660	.000	.999	
Species	.000	0	.	.	.	.000	
NitroS	72.153	13	5.550	364.520	.000	.994	
Species * NitroS	.000	0	.	.	.	.000	
Error	.426	28	.015				
Total	447.758	42					
Corrected Total	72.579	41					

a. Species = RK	
b. R Squared = .994 (Adjusted R Squared = .991)	


Estimated Marginal Means


1. Grand Meana	
Dependent Variable:   Bio  	
Mean	Std. Error	95% Confidence Interval	
		Lower Bound	Upper Bound	
2.989	.019	2.950	3.028	

a. Species = RK	


2. Species


Estimatesa	
Dependent Variable:   Bio  	
Species	Mean	Std. Error	95% Confidence Interval	
			Lower Bound	Upper Bound	
RK	2.989	.019	2.950	3.028	

a. Species = RK	


Pairwise Comparisonsa,b	
	

a. Species = RK	
b. This pairwise comparison table cannot be constructed because Species, the factor being compared, has one level.	


Univariate Testsa	
Dependent Variable:   Bio  	
	Sum of Squares	df	Mean Square	F	Sig.	Partial Eta Squared	
Contrast	.000	0	.	.	.	.000	
Error	.426	28	.015				

The F tests the effect of Species. This test is based on the linearly independent pairwise comparisons among the estimated marginal means.a	
a. Species = RK	


3. NitroS


Estimatesa	
Dependent Variable:   Bio  	
NitroS	Mean	Std. Error	95% Confidence Interval	
			Lower Bound	Upper Bound	
Pep	2.503	.071	2.357	2.649	
Urea	5.054	.071	4.908	5.200	
YeEx	2.600	.071	2.454	2.746	
NCl	2.726	.071	2.580	2.872	
NS	2.769	.071	2.623	2.915	
Pep+YeEx	1.238	.071	1.092	1.384	
Urea+Pep	3.145	.071	2.999	3.291	
YeEx+Urea	1.653	.071	1.507	1.799	
Pep+NCl	2.814	.071	2.668	2.960	
Urea+NCl	3.384	.071	3.238	3.530	
YeEx+NCl	6.282	.071	6.136	6.428	
Pep+NS	1.559	.071	1.413	1.705	
Urea+NS	2.246	.071	2.100	2.392	
YeEx+NS	3.872	.071	3.726	4.018	

a. Species = RK	


Pairwise Comparisonsa	
Dependent Variable:   Bio  	
(I) NitroS	(J) NitroS	Mean Difference (I-J)	Std. Error	Sig.c	95% Confidence Interval for Differencec	
					Lower Bound	Upper Bound	
Pep	Urea	-2.551*	.101	.000	-2.758	-2.345	
	YeEx	-.097	.101	.344	-.303	.109	
	NCl	-.223*	.101	.035	-.430	-.017	
	NS	-.266*	.101	.013	-.472	-.060	
	Pep+YeEx	1.264*	.101	.000	1.058	1.471	
	Urea+Pep	-.642*	.101	.000	-.848	-.436	
	YeEx+Urea	.849*	.101	.000	.643	1.056	
	Pep+NCl	-.311*	.101	.004	-.518	-.105	
	Urea+NCl	-.881*	.101	.000	-1.087	-.675	
	YeEx+NCl	-3.779*	.101	.000	-3.986	-3.573	
	Pep+NS	.944*	.101	.000	.738	1.150	
	Urea+NS	.257*	.101	.016	.051	.463	
	YeEx+NS	-1.369*	.101	.000	-1.575	-1.163	
Urea	Pep	2.551*	.101	.000	2.345	2.758	
	YeEx	2.454*	.101	.000	2.248	2.661	
	NCl	2.328*	.101	.000	2.122	2.534	
	NS	2.285*	.101	.000	2.079	2.492	
	Pep+YeEx	3.816*	.101	.000	3.609	4.022	
	Urea+Pep	1.909*	.101	.000	1.703	2.116	
	YeEx+Urea	3.401*	.101	.000	3.194	3.607	
	Pep+NCl	2.240*	.101	.000	2.034	2.446	
	Urea+NCl	1.670*	.101	.000	1.464	1.877	
	YeEx+NCl	-1.228*	.101	.000	-1.434	-1.022	
	Pep+NS	3.495*	.101	.000	3.289	3.702	
	Urea+NS	2.808*	.101	.000	2.602	3.015	
	YeEx+NS	1.182*	.101	.000	.976	1.389	
YeEx	Pep	.097	.101	.344	-.109	.303	
	Urea	-2.454*	.101	.000	-2.661	-2.248	
	NCl	-.126	.101	.220	-.333	.080	
	NS	-.169	.101	.105	-.375	.037	
	Pep+YeEx	1.361*	.101	.000	1.155	1.568	
	Urea+Pep	-.545*	.101	.000	-.751	-.339	
	YeEx+Urea	.946*	.101	.000	.740	1.153	
	Pep+NCl	-.214*	.101	.042	-.421	-.008	
	Urea+NCl	-.784*	.101	.000	-.990	-.578	
	YeEx+NCl	-3.682*	.101	.000	-3.889	-3.476	
	Pep+NS	1.041*	.101	.000	.835	1.247	
	Urea+NS	.354*	.101	.002	.148	.560	
	YeEx+NS	-1.272*	.101	.000	-1.478	-1.066	
NCl	Pep	.223*	.101	.035	.017	.430	
	Urea	-2.328*	.101	.000	-2.534	-2.122	
	YeEx	.126	.101	.220	-.080	.333	
	NS	-.043	.101	.675	-.249	.164	
	Pep+YeEx	1.488*	.101	.000	1.281	1.694	
	Urea+Pep	-.419*	.101	.000	-.625	-.212	
	YeEx+Urea	1.073*	.101	.000	.866	1.279	
	Pep+NCl	-.088	.101	.390	-.294	.118	
	Urea+NCl	-.658*	.101	.000	-.864	-.451	
	YeEx+NCl	-3.556*	.101	.000	-3.762	-3.350	
	Pep+NS	1.167*	.101	.000	.961	1.374	
	Urea+NS	.480*	.101	.000	.274	.687	
	YeEx+NS	-1.146*	.101	.000	-1.352	-.939	
NS	Pep	.266*	.101	.013	.060	.472	
	Urea	-2.285*	.101	.000	-2.492	-2.079	
	YeEx	.169	.101	.105	-.037	.375	
	NCl	.043	.101	.675	-.164	.249	
	Pep+YeEx	1.530*	.101	.000	1.324	1.737	
	Urea+Pep	-.376*	.101	.001	-.582	-.170	
	YeEx+Urea	1.115*	.101	.000	.909	1.322	
	Pep+NCl	-.045	.101	.656	-.252	.161	
	Urea+NCl	-.615*	.101	.000	-.821	-.409	
	YeEx+NCl	-3.513*	.101	.000	-3.720	-3.307	
	Pep+NS	1.210*	.101	.000	1.004	1.416	
	Urea+NS	.523*	.101	.000	.317	.729	
	YeEx+NS	-1.103*	.101	.000	-1.309	-.897	
Pep+YeEx	Pep	-1.264*	.101	.000	-1.471	-1.058	
	Urea	-3.816*	.101	.000	-4.022	-3.609	
	YeEx	-1.361*	.101	.000	-1.568	-1.155	
	NCl	-1.488*	.101	.000	-1.694	-1.281	
	NS	-1.530*	.101	.000	-1.737	-1.324	
	Urea+Pep	-1.906*	.101	.000	-2.113	-1.700	
	YeEx+Urea	-.415*	.101	.000	-.621	-.209	
	Pep+NCl	-1.576*	.101	.000	-1.782	-1.369	
	Urea+NCl	-2.145*	.101	.000	-2.352	-1.939	
	YeEx+NCl	-5.044*	.101	.000	-5.250	-4.837	
	Pep+NS	-.320*	.101	.004	-.527	-.114	
	Urea+NS	-1.007*	.101	.000	-1.214	-.801	
	YeEx+NS	-2.633*	.101	.000	-2.840	-2.427	
Urea+Pep	Pep	.642*	.101	.000	.436	.848	
	Urea	-1.909*	.101	.000	-2.116	-1.703	
	YeEx	.545*	.101	.000	.339	.751	
	NCl	.419*	.101	.000	.212	.625	
	NS	.376*	.101	.001	.170	.582	
	Pep+YeEx	1.906*	.101	.000	1.700	2.113	
	YeEx+Urea	1.491*	.101	.000	1.285	1.698	
	Pep+NCl	.331*	.101	.003	.124	.537	
	Urea+NCl	-.239*	.101	.025	-.445	-.033	
	YeEx+NCl	-3.137*	.101	.000	-3.344	-2.931	
	Pep+NS	1.586*	.101	.000	1.380	1.792	
	Urea+NS	.899*	.101	.000	.693	1.105	
	YeEx+NS	-.727*	.101	.000	-.933	-.521	
YeEx+Urea	Pep	-.849*	.101	.000	-1.056	-.643	
	Urea	-3.401*	.101	.000	-3.607	-3.194	
	YeEx	-.946*	.101	.000	-1.153	-.740	
	NCl	-1.073*	.101	.000	-1.279	-.866	
	NS	-1.115*	.101	.000	-1.322	-.909	
	Pep+YeEx	.415*	.101	.000	.209	.621	
	Urea+Pep	-1.491*	.101	.000	-1.698	-1.285	
	Pep+NCl	-1.161*	.101	.000	-1.367	-.954	
	Urea+NCl	-1.730*	.101	.000	-1.937	-1.524	
	YeEx+NCl	-4.629*	.101	.000	-4.835	-4.422	
	Pep+NS	.095	.101	.355	-.112	.301	
	Urea+NS	-.592*	.101	.000	-.799	-.386	
	YeEx+NS	-2.218*	.101	.000	-2.425	-2.012	
Pep+NCl	Pep	.311*	.101	.004	.105	.518	
	Urea	-2.240*	.101	.000	-2.446	-2.034	
	YeEx	.214*	.101	.042	.008	.421	
	NCl	.088	.101	.390	-.118	.294	
	NS	.045	.101	.656	-.161	.252	
	Pep+YeEx	1.576*	.101	.000	1.369	1.782	
	Urea+Pep	-.331*	.101	.003	-.537	-.124	
	YeEx+Urea	1.161*	.101	.000	.954	1.367	
	Urea+NCl	-.570*	.101	.000	-.776	-.363	
	YeEx+NCl	-3.468*	.101	.000	-3.674	-3.262	
	Pep+NS	1.255*	.101	.000	1.049	1.462	
	Urea+NS	.568*	.101	.000	.362	.775	
	YeEx+NS	-1.058*	.101	.000	-1.264	-.851	
Urea+NCl	Pep	.881*	.101	.000	.675	1.087	
	Urea	-1.670*	.101	.000	-1.877	-1.464	
	YeEx	.784*	.101	.000	.578	.990	
	NCl	.658*	.101	.000	.451	.864	
	NS	.615*	.101	.000	.409	.821	
	Pep+YeEx	2.145*	.101	.000	1.939	2.352	
	Urea+Pep	.239*	.101	.025	.033	.445	
	YeEx+Urea	1.730*	.101	.000	1.524	1.937	
	Pep+NCl	.570*	.101	.000	.363	.776	
	YeEx+NCl	-2.898*	.101	.000	-3.105	-2.692	
	Pep+NS	1.825*	.101	.000	1.619	2.031	
	Urea+NS	1.138*	.101	.000	.932	1.344	
	YeEx+NS	-.488*	.101	.000	-.694	-.282	
YeEx+NCl	Pep	3.779*	.101	.000	3.573	3.986	
	Urea	1.228*	.101	.000	1.022	1.434	
	YeEx	3.682*	.101	.000	3.476	3.889	
	NCl	3.556*	.101	.000	3.350	3.762	
	NS	3.513*	.101	.000	3.307	3.720	
	Pep+YeEx	5.044*	.101	.000	4.837	5.250	
	Urea+Pep	3.137*	.101	.000	2.931	3.344	
	YeEx+Urea	4.629*	.101	.000	4.422	4.835	
	Pep+NCl	3.468*	.101	.000	3.262	3.674	
	Urea+NCl	2.898*	.101	.000	2.692	3.105	
	Pep+NS	4.723*	.101	.000	4.517	4.930	
	Urea+NS	4.036*	.101	.000	3.830	4.243	
	YeEx+NS	2.410*	.101	.000	2.204	2.617	
Pep+NS	Pep	-.944*	.101	.000	-1.150	-.738	
	Urea	-3.495*	.101	.000	-3.702	-3.289	
	YeEx	-1.041*	.101	.000	-1.247	-.835	
	NCl	-1.167*	.101	.000	-1.374	-.961	
	NS	-1.210*	.101	.000	-1.416	-1.004	
	Pep+YeEx	.320*	.101	.004	.114	.527	
	Urea+Pep	-1.586*	.101	.000	-1.792	-1.380	
	YeEx+Urea	-.095	.101	.355	-.301	.112	
	Pep+NCl	-1.255*	.101	.000	-1.462	-1.049	
	Urea+NCl	-1.825*	.101	.000	-2.031	-1.619	
	YeEx+NCl	-4.723*	.101	.000	-4.930	-4.517	
	Urea+NS	-.687*	.101	.000	-.893	-.481	
	YeEx+NS	-2.313*	.101	.000	-2.519	-2.107	
Urea+NS	Pep	-.257*	.101	.016	-.463	-.051	
	Urea	-2.808*	.101	.000	-3.015	-2.602	
	YeEx	-.354*	.101	.002	-.560	-.148	
	NCl	-.480*	.101	.000	-.687	-.274	
	NS	-.523*	.101	.000	-.729	-.317	
	Pep+YeEx	1.007*	.101	.000	.801	1.214	
	Urea+Pep	-.899*	.101	.000	-1.105	-.693	
	YeEx+Urea	.592*	.101	.000	.386	.799	
	Pep+NCl	-.568*	.101	.000	-.775	-.362	
	Urea+NCl	-1.138*	.101	.000	-1.344	-.932	
	YeEx+NCl	-4.036*	.101	.000	-4.243	-3.830	
	Pep+NS	.687*	.101	.000	.481	.893	
	YeEx+NS	-1.626*	.101	.000	-1.832	-1.420	
YeEx+NS	Pep	1.369*	.101	.000	1.163	1.575	
	Urea	-1.182*	.101	.000	-1.389	-.976	
	YeEx	1.272*	.101	.000	1.066	1.478	
	NCl	1.146*	.101	.000	.939	1.352	
	NS	1.103*	.101	.000	.897	1.309	
	Pep+YeEx	2.633*	.101	.000	2.427	2.840	
	Urea+Pep	.727*	.101	.000	.521	.933	
	YeEx+Urea	2.218*	.101	.000	2.012	2.425	
	Pep+NCl	1.058*	.101	.000	.851	1.264	
	Urea+NCl	.488*	.101	.000	.282	.694	
	YeEx+NCl	-2.410*	.101	.000	-2.617	-2.204	
	Pep+NS	2.313*	.101	.000	2.107	2.519	
	Urea+NS	1.626*	.101	.000	1.420	1.832	

Based on estimated marginal meansa	
*. The mean difference is significant at the .05 level.	
a. Species = RK	
c. Adjustment for multiple comparisons: Least Significant Difference (equivalent to no adjustments).	


Univariate Testsa	
Dependent Variable:   Bio  	
	Sum of Squares	df	Mean Square	F	Sig.	Partial Eta Squared	
Contrast	72.153	13	5.550	364.520	.000	.994	
Error	.426	28	.015				

The F tests the effect of NitroS. This test is based on the linearly independent pairwise comparisons among the estimated marginal means.a	
a. Species = RK	


4. Species * NitroSa	
Dependent Variable:   Bio  	
Species	NitroS	Mean	Std. Error	95% Confidence Interval	
				Lower Bound	Upper Bound	
RK	Pep	2.503	.071	2.357	2.649	
	Urea	5.054	.071	4.908	5.200	
	YeEx	2.600	.071	2.454	2.746	
	NCl	2.726	.071	2.580	2.872	
	NS	2.769	.071	2.623	2.915	
	Pep+YeEx	1.238	.071	1.092	1.384	
	Urea+Pep	3.145	.071	2.999	3.291	
	YeEx+Urea	1.653	.071	1.507	1.799	
	Pep+NCl	2.814	.071	2.668	2.960	
	Urea+NCl	3.384	.071	3.238	3.530	
	YeEx+NCl	6.282	.071	6.136	6.428	
	Pep+NS	1.559	.071	1.413	1.705	
	Urea+NS	2.246	.071	2.100	2.392	
	YeEx+NS	3.872	.071	3.726	4.018	

a. Species = RK	


Post Hoc Tests


NitroS


Multiple Comparisonsa	
Dependent Variable:   Bio  	
Tukey HSD  	
(I) NitroS	(J) NitroS	Mean Difference (I-J)	Std. Error	Sig.	95% Confidence Interval	
					Lower Bound	Upper Bound	
Pep	Urea	-2.55133*	.100751	.000	-2.92012	-2.18255	
	YeEx	-.09700	.100751	.999	-.46579	.27179	
	NCl	-.22333	.100751	.622	-.59212	.14545	
	NS	-.26600	.100751	.361	-.63479	.10279	
	Pep+YeEx	1.26433*	.100751	.000	.89555	1.63312	
	Urea+Pep	-.64200*	.100751	.000	-1.01079	-.27321	
	YeEx+Urea	.84933*	.100751	.000	.48055	1.21812	
	Pep+NCl	-.31133	.100751	.166	-.68012	.05745	
	Urea+NCl	-.88100*	.100751	.000	-1.24979	-.51221	
	YeEx+NCl	-3.77933*	.100751	.000	-4.14812	-3.41055	
	Pep+NS	.94400*	.100751	.000	.57521	1.31279	
	Urea+NS	.25700	.100751	.412	-.11179	.62579	
	YeEx+NS	-1.36900*	.100751	.000	-1.73779	-1.00021	
Urea	Pep	2.55133*	.100751	.000	2.18255	2.92012	
	YeEx	2.45433*	.100751	.000	2.08555	2.82312	
	NCl	2.32800*	.100751	.000	1.95921	2.69679	
	NS	2.28533*	.100751	.000	1.91655	2.65412	
	Pep+YeEx	3.81567*	.100751	.000	3.44688	4.18445	
	Urea+Pep	1.90933*	.100751	.000	1.54055	2.27812	
	YeEx+Urea	3.40067*	.100751	.000	3.03188	3.76945	
	Pep+NCl	2.24000*	.100751	.000	1.87121	2.60879	
	Urea+NCl	1.67033*	.100751	.000	1.30155	2.03912	
	YeEx+NCl	-1.22800*	.100751	.000	-1.59679	-.85921	
	Pep+NS	3.49533*	.100751	.000	3.12655	3.86412	
	Urea+NS	2.80833*	.100751	.000	2.43955	3.17712	
	YeEx+NS	1.18233*	.100751	.000	.81355	1.55112	
YeEx	Pep	.09700	.100751	.999	-.27179	.46579	
	Urea	-2.45433*	.100751	.000	-2.82312	-2.08555	
	NCl	-.12633	.100751	.989	-.49512	.24245	
	NS	-.16900	.100751	.907	-.53779	.19979	
	Pep+YeEx	1.36133*	.100751	.000	.99255	1.73012	
	Urea+Pep	-.54500*	.100751	.001	-.91379	-.17621	
	YeEx+Urea	.94633*	.100751	.000	.57755	1.31512	
	Pep+NCl	-.21433	.100751	.678	-.58312	.15445	
	Urea+NCl	-.78400*	.100751	.000	-1.15279	-.41521	
	YeEx+NCl	-3.68233*	.100751	.000	-4.05112	-3.31355	
	Pep+NS	1.04100*	.100751	.000	.67221	1.40979	
	Urea+NS	.35400	.100751	.069	-.01479	.72279	
	YeEx+NS	-1.27200*	.100751	.000	-1.64079	-.90321	
NCl	Pep	.22333	.100751	.622	-.14545	.59212	
	Urea	-2.32800*	.100751	.000	-2.69679	-1.95921	
	YeEx	.12633	.100751	.989	-.24245	.49512	
	NS	-.04267	.100751	1.000	-.41145	.32612	
	Pep+YeEx	1.48767*	.100751	.000	1.11888	1.85645	
	Urea+Pep	-.41867*	.100751	.016	-.78745	-.04988	
	YeEx+Urea	1.07267*	.100751	.000	.70388	1.44145	
	Pep+NCl	-.08800	.100751	1.000	-.45679	.28079	
	Urea+NCl	-.65767*	.100751	.000	-1.02645	-.28888	
	YeEx+NCl	-3.55600*	.100751	.000	-3.92479	-3.18721	
	Pep+NS	1.16733*	.100751	.000	.79855	1.53612	
	Urea+NS	.48033*	.100751	.003	.11155	.84912	
	YeEx+NS	-1.14567*	.100751	.000	-1.51445	-.77688	
NS	Pep	.26600	.100751	.361	-.10279	.63479	
	Urea	-2.28533*	.100751	.000	-2.65412	-1.91655	
	YeEx	.16900	.100751	.907	-.19979	.53779	
	NCl	.04267	.100751	1.000	-.32612	.41145	
	Pep+YeEx	1.53033*	.100751	.000	1.16155	1.89912	
	Urea+Pep	-.37600*	.100751	.042	-.74479	-.00721	
	YeEx+Urea	1.11533*	.100751	.000	.74655	1.48412	
	Pep+NCl	-.04533	.100751	1.000	-.41412	.32345	
	Urea+NCl	-.61500*	.100751	.000	-.98379	-.24621	
	YeEx+NCl	-3.51333*	.100751	.000	-3.88212	-3.14455	
	Pep+NS	1.21000*	.100751	.000	.84121	1.57879	
	Urea+NS	.52300*	.100751	.001	.15421	.89179	
	YeEx+NS	-1.10300*	.100751	.000	-1.47179	-.73421	
Pep+YeEx	Pep	-1.26433*	.100751	.000	-1.63312	-.89555	
	Urea	-3.81567*	.100751	.000	-4.18445	-3.44688	
	YeEx	-1.36133*	.100751	.000	-1.73012	-.99255	
	NCl	-1.48767*	.100751	.000	-1.85645	-1.11888	
	NS	-1.53033*	.100751	.000	-1.89912	-1.16155	
	Urea+Pep	-1.90633*	.100751	.000	-2.27512	-1.53755	
	YeEx+Urea	-.41500*	.100751	.017	-.78379	-.04621	
	Pep+NCl	-1.57567*	.100751	.000	-1.94445	-1.20688	
	Urea+NCl	-2.14533*	.100751	.000	-2.51412	-1.77655	
	YeEx+NCl	-5.04367*	.100751	.000	-5.41245	-4.67488	
	Pep+NS	-.32033	.100751	.139	-.68912	.04845	
	Urea+NS	-1.00733*	.100751	.000	-1.37612	-.63855	
	YeEx+NS	-2.63333*	.100751	.000	-3.00212	-2.26455	
Urea+Pep	Pep	.64200*	.100751	.000	.27321	1.01079	
	Urea	-1.90933*	.100751	.000	-2.27812	-1.54055	
	YeEx	.54500*	.100751	.001	.17621	.91379	
	NCl	.41867*	.100751	.016	.04988	.78745	
	NS	.37600*	.100751	.042	.00721	.74479	
	Pep+YeEx	1.90633*	.100751	.000	1.53755	2.27512	
	YeEx+Urea	1.49133*	.100751	.000	1.12255	1.86012	
	Pep+NCl	.33067	.100751	.113	-.03812	.69945	
	Urea+NCl	-.23900	.100751	.521	-.60779	.12979	
	YeEx+NCl	-3.13733*	.100751	.000	-3.50612	-2.76855	
	Pep+NS	1.58600*	.100751	.000	1.21721	1.95479	
	Urea+NS	.89900*	.100751	.000	.53021	1.26779	
	YeEx+NS	-.72700*	.100751	.000	-1.09579	-.35821	
YeEx+Urea	Pep	-.84933*	.100751	.000	-1.21812	-.48055	
	Urea	-3.40067*	.100751	.000	-3.76945	-3.03188	
	YeEx	-.94633*	.100751	.000	-1.31512	-.57755	
	NCl	-1.07267*	.100751	.000	-1.44145	-.70388	
	NS	-1.11533*	.100751	.000	-1.48412	-.74655	
	Pep+YeEx	.41500*	.100751	.017	.04621	.78379	
	Urea+Pep	-1.49133*	.100751	.000	-1.86012	-1.12255	
	Pep+NCl	-1.16067*	.100751	.000	-1.52945	-.79188	
	Urea+NCl	-1.73033*	.100751	.000	-2.09912	-1.36155	
	YeEx+NCl	-4.62867*	.100751	.000	-4.99745	-4.25988	
	Pep+NS	.09467	.100751	.999	-.27412	.46345	
	Urea+NS	-.59233*	.100751	.000	-.96112	-.22355	
	YeEx+NS	-2.21833*	.100751	.000	-2.58712	-1.84955	
Pep+NCl	Pep	.31133	.100751	.166	-.05745	.68012	
	Urea	-2.24000*	.100751	.000	-2.60879	-1.87121	
	YeEx	.21433	.100751	.678	-.15445	.58312	
	NCl	.08800	.100751	1.000	-.28079	.45679	
	NS	.04533	.100751	1.000	-.32345	.41412	
	Pep+YeEx	1.57567*	.100751	.000	1.20688	1.94445	
	Urea+Pep	-.33067	.100751	.113	-.69945	.03812	
	YeEx+Urea	1.16067*	.100751	.000	.79188	1.52945	
	Urea+NCl	-.56967*	.100751	.000	-.93845	-.20088	
	YeEx+NCl	-3.46800*	.100751	.000	-3.83679	-3.09921	
	Pep+NS	1.25533*	.100751	.000	.88655	1.62412	
	Urea+NS	.56833*	.100751	.000	.19955	.93712	
	YeEx+NS	-1.05767*	.100751	.000	-1.42645	-.68888	
Urea+NCl	Pep	.88100*	.100751	.000	.51221	1.24979	
	Urea	-1.67033*	.100751	.000	-2.03912	-1.30155	
	YeEx	.78400*	.100751	.000	.41521	1.15279	
	NCl	.65767*	.100751	.000	.28888	1.02645	
	NS	.61500*	.100751	.000	.24621	.98379	
	Pep+YeEx	2.14533*	.100751	.000	1.77655	2.51412	
	Urea+Pep	.23900	.100751	.521	-.12979	.60779	
	YeEx+Urea	1.73033*	.100751	.000	1.36155	2.09912	
	Pep+NCl	.56967*	.100751	.000	.20088	.93845	
	YeEx+NCl	-2.89833*	.100751	.000	-3.26712	-2.52955	
	Pep+NS	1.82500*	.100751	.000	1.45621	2.19379	
	Urea+NS	1.13800*	.100751	.000	.76921	1.50679	
	YeEx+NS	-.48800*	.100751	.003	-.85679	-.11921	
YeEx+NCl	Pep	3.77933*	.100751	.000	3.41055	4.14812	
	Urea	1.22800*	.100751	.000	.85921	1.59679	
	YeEx	3.68233*	.100751	.000	3.31355	4.05112	
	NCl	3.55600*	.100751	.000	3.18721	3.92479	
	NS	3.51333*	.100751	.000	3.14455	3.88212	
	Pep+YeEx	5.04367*	.100751	.000	4.67488	5.41245	
	Urea+Pep	3.13733*	.100751	.000	2.76855	3.50612	
	YeEx+Urea	4.62867*	.100751	.000	4.25988	4.99745	
	Pep+NCl	3.46800*	.100751	.000	3.09921	3.83679	
	Urea+NCl	2.89833*	.100751	.000	2.52955	3.26712	
	Pep+NS	4.72333*	.100751	.000	4.35455	5.09212	
	Urea+NS	4.03633*	.100751	.000	3.66755	4.40512	
	YeEx+NS	2.41033*	.100751	.000	2.04155	2.77912	
Pep+NS	Pep	-.94400*	.100751	.000	-1.31279	-.57521	
	Urea	-3.49533*	.100751	.000	-3.86412	-3.12655	
	YeEx	-1.04100*	.100751	.000	-1.40979	-.67221	
	NCl	-1.16733*	.100751	.000	-1.53612	-.79855	
	NS	-1.21000*	.100751	.000	-1.57879	-.84121	
	Pep+YeEx	.32033	.100751	.139	-.04845	.68912	
	Urea+Pep	-1.58600*	.100751	.000	-1.95479	-1.21721	
	YeEx+Urea	-.09467	.100751	.999	-.46345	.27412	
	Pep+NCl	-1.25533*	.100751	.000	-1.62412	-.88655	
	Urea+NCl	-1.82500*	.100751	.000	-2.19379	-1.45621	
	YeEx+NCl	-4.72333*	.100751	.000	-5.09212	-4.35455	
	Urea+NS	-.68700*	.100751	.000	-1.05579	-.31821	
	YeEx+NS	-2.31300*	.100751	.000	-2.68179	-1.94421	
Urea+NS	Pep	-.25700	.100751	.412	-.62579	.11179	
	Urea	-2.80833*	.100751	.000	-3.17712	-2.43955	
	YeEx	-.35400	.100751	.069	-.72279	.01479	
	NCl	-.48033*	.100751	.003	-.84912	-.11155	
	NS	-.52300*	.100751	.001	-.89179	-.15421	
	Pep+YeEx	1.00733*	.100751	.000	.63855	1.37612	
	Urea+Pep	-.89900*	.100751	.000	-1.26779	-.53021	
	YeEx+Urea	.59233*	.100751	.000	.22355	.96112	
	Pep+NCl	-.56833*	.100751	.000	-.93712	-.19955	
	Urea+NCl	-1.13800*	.100751	.000	-1.50679	-.76921	
	YeEx+NCl	-4.03633*	.100751	.000	-4.40512	-3.66755	
	Pep+NS	.68700*	.100751	.000	.31821	1.05579	
	YeEx+NS	-1.62600*	.100751	.000	-1.99479	-1.25721	
YeEx+NS	Pep	1.36900*	.100751	.000	1.00021	1.73779	
	Urea	-1.18233*	.100751	.000	-1.55112	-.81355	
	YeEx	1.27200*	.100751	.000	.90321	1.64079	
	NCl	1.14567*	.100751	.000	.77688	1.51445	
	NS	1.10300*	.100751	.000	.73421	1.47179	
	Pep+YeEx	2.63333*	.100751	.000	2.26455	3.00212	
	Urea+Pep	.72700*	.100751	.000	.35821	1.09579	
	YeEx+Urea	2.21833*	.100751	.000	1.84955	2.58712	
	Pep+NCl	1.05767*	.100751	.000	.68888	1.42645	
	Urea+NCl	.48800*	.100751	.003	.11921	.85679	
	YeEx+NCl	-2.41033*	.100751	.000	-2.77912	-2.04155	
	Pep+NS	2.31300*	.100751	.000	1.94421	2.68179	
	Urea+NS	1.62600*	.100751	.000	1.25721	1.99479	

Based on observed means.
 The error term is Mean Square(Error) = .015.a	
*. The mean difference is significant at the .05 level.	
a. Species = RK	


Homogeneous Subsets


Bioa	
Tukey HSDb,c  	
NitroS	N	Subset			
		1	2	3	4	5	6	7			
Pep+YeEx	3	1.23833									
Pep+NS	3	1.55867	1.55867								
YeEx+Urea	3		1.65333								
Urea+NS	3			2.24567							
Pep	3			2.50267	2.50267						
YeEx	3			2.59967	2.59967						
NCl	3				2.72600						
NS	3				2.76867						
Pep+NCl	3				2.81400	2.81400					
Urea+Pep	3					3.14467	3.14467				
Urea+NCl	3						3.38367				
YeEx+NS	3							3.87167			
Urea	3										
YeEx+NCl	3										
Sig.		.139	.999	.069	.166	.113	.521	1.000			


Profile Plots


Zÿ566ê[Êçóùý~¤¤¤¨éééérV&ª­nnjzff¦µZØ+Êbe'·+·µÄ¿©´´TÛ©¦ÈMHªÊàGá@ù@x]]]_ÒL¡Ñ&Á ÆIIIúü³¡ó-¿°WliiIMMUSÌfskkëå'K.F«ÕêõzõIÅz<¹«²(ILV§¶í3133£¦KK©éiii¡Ûöæ~µÍ/¨·ôå·Øggg;::ÔQÆÚÅÈå'ì1>|8ô¢êêj×××ó üà×rss%. ãöövïÝ»WßRv»½¥¥ESjúþýûåìÙ³gÔµjºÚÏ¯©©IMÝc/ìe<00055%­[·ÎEGFFFRRÒØØXÐEjGCùÕx|P~ðk.«²²2%%E*--íàÁAï677«ÏLöx<jºÌpèÐ¡ääd£Ñh³Ù´c)fggkjjdfýt½¢_yy¹`0<ôÐCÃÃÃQßÜ¯6û^¤>Ò/ôe ü`nÉÌP~(?å Î°òåÊòK|/½ôÒÕ«WyÔåø¾úÕ¯Jüñ¨Êò ü(?Êò ü(?Êò ü(?Êò ü(?Êò$þùÓ§O:îëëÛ±cÇýÜâl6[rr²Á`HMMÝ»wïðð0åGù°Jå299MùéÇ1hnnÞBnø£ü(?V©üDIIÉbåÍô(mÞ¼Y®^[[;33#g'&&l6L)--å± ü(?V£üöíÛ'?[ZZÂ6ÚV§M9þ|jjêöíÛÕü£££eee¦åååcccÚbF£Ì¯ßÂçr¹dÌÉcAùQ~¬FùMOOgff¦§§Ë òKJJÒ¶ÛIÉÍfýl²Xí­dG¦=ötttÊoÁÚQÓé§µ<Û¶mÛÖÕÕåÊðå'¶¶68p BùÍ¼Û«Î~ùË_SÛöªªªdÊîÝ»=d ge¢ßívçååé÷ð;xð ~£ (¿=¶®®N?¥¼¼Ê@¦WTTD9òlòòÂ'ãåËåÒ¦¤§§ë§LLLÈÙ´´4ý¶¶¶uuÉÔÝÝÍcAùÅn||¼¨¨(h¢ÙlV[ý~VVVSÊïþé:X#´üÜn·¼fggû|¾eþM[ÕsA7!Cozvv¶½½]^¯e¼¼<¢òü%Ñ××4Qÿ´Sãh¦ß/¼p5ò@ËO;wNÎ=ztYå´%tLÔ_:>>®Í/Õ¸XòÊÈÈÕj®ßíÏh4F9wäÝ^¥¤¤D±¶öÔEÞa¯®Þ½÷ôuHGuuµºôÀrvûöíòb-ggff>,S¶mÛÆcAùÅèÑG=yòdèôôôt¿ß¯ÞÉqS(?À*¿ÉÉÉÅÊ/33S­¬¬õ±±1íêÚ±½×®]SNLL¨´¶¶òXP~1¿$úûûC§Ës´±±Qò³¼¼<Ê)`ChhhX¬üÚÚÚÔ`¨Oïõááá²²2ãy1Õÿ1·°/þ¾ûÒÒÒÔ±6íAì¿T~ò<ú| õ¤ìééÉÈÈHJJ§loooS(?õ[~å(?Êò£ü(?Êò£ü(?Êò£ü(?Êò£ü(?Êò£üåGùP~ññ¢þ+III&©¢¢Âív³Z@ùå§@[[[ee%«	^~Ñhdµò`-µ¿îmí¿qÿ'YNòÉÉÉj<==]\l0l6µùÉdÚ±cïS~ñZ~§O>xð :[]]=99)I=»Ý®Í¿ÿ~³©©Ifàq¡ü(?âçE]'--MbÎëýeÍf5ðù|ÚüSSS2ðûý©©©¬@Êò Ê/(LJJß`0°)?ÊD(¿°ôdþ¹m~ééé¬@Êò Ê¯ººÚårÉ ¥¥¥°°PÿðáÃ2hjjª©©aR~P~Çf³ÜÜÜmþ£ÑXZZª6þò£üØp¥ÊòïÎÜgUOYëåÊòÖÂçÞ±çWõÔqµ¬7|ÃåGùÃl`îúíNó×3ËuoùYë@ùQ~@<ò»ò>ë(?Ê üå$[þ/½=ÿnïÙçÞþõå$¨½÷¼x¯óÍùm~Ï¿qï/Îõ¾ÃZÖÝzÈ±º½ÊòíÞS¯ÌõÏiïöÞòßû]|P@ùò£ü3¸÷äÅ¹À½_ßÏ'æ]ÃsýWY7åÊòöÞ×òëòSÿ^zÎù.ëë½·£êþO²(ËOõõõÙÙÙ2...66MÆj§Ó/ÓÓÓy(?ÊØ¸îó¥¹·¦æ´wgó»ú½û1kÍíWÞ¸õÒÀýd9Ñ_KKÏçquuõää¤ÝnW3X,îîn´µµ¥¦¦òQ~°½ûñü¾ãóå×ýö½§óßÉ±°À:zQ)¿¤¤$í¢@  Æf³Y$322¢Y(?ÊØ`>öÞkþS]¾ùÒü¾d°þL&ýYI=m>æ6éhièñx?^YYi±X(?Êò0OrÖ³âââíìðððÎ;CË/ì¹V«µ¶¶¶££cbbò£ü(?°ÞõõõIêIºÉx|||×®]]]]¡åW]]ír¹dÐÒÒRXX¨&L¦ÑÑQ¿ßo·Û)?Êò@ùq µµuóæÍA~666þúõ^sÇf³É<¹¹¹Ú6ÂÅzúôiÊò£üÄ2áßco5OWXë@ùQ~ÀZð~6¿én5O®OXë@ùQ~å@ùQ~P~>oß¾F£qË-/^Ô_ät:333Ã¶mÛÔaêÑL¡üÖiù=úè£Çê¥5ååågÏA]]]EEES(?uZ~Û¶mÙlVß9è÷û³²²¢T~õõõ¯°Fèkè®]»N§vVÆ2%RCQ~«Ê`0<yÒd2egg]4fJPù=÷ÜsS¬ú:88XTT¤ñåË#%Dù­±¤¤¤¹oÌÏÏºHÆ(§ðn/`ã(++S»¹wwwËx ü(¿5¾ØF;¹Èï÷«wrÕlÑL¡üëMÁ×þå±ßºÿ,'hÉÚvíÙÄôôtqq±¼°Úl6/Y~2[__*ÈÐýæ±ÑËo÷îÝÉÉÉsßýl2äU^^Û¢ª««Ï;7·°ÉZûi¥²²R¡üTËfåØPöîÝ[UUõðÃ½¼NNNÊÀápØív­üÛÏO½)HuEP~¿´gÏy¢$%%ÉóCÿÔg^K?DJKK¥åOíõDìééÉÈÈÊÌÌìííråØPÆÇÇåusllL?Ñl6«ÏçJýËëb¤kkkY¥ßoHII§N__ßðð°,ËåL_o¿-åØ¯î!I§ß4£í¹üúûû"ëòód[8Hj²þEù°¶åÍlzò.¯ìO<ñëòûÉÉÉòÔVÿ&''/^¼È6?ÖOùUWW»¹=òµÝè#ßèè¨Õjõûý¹¹¹ìçGùýÝ»wkÕ®÷³åÀÇã±ÙlAJN¿ýbGx¨Ýå»ººJKKY¥ßo(((HJJìSË8òS~ñZ~ñòå@ùEÇjµ 8¶ ÑÊO>ø ï^£üâ¾üÔÖ¾ÆÆÆÙÙÙuþÛR~ò»/F£1òGAR~¬Æú¦M#h6µÙlòªYWWvP~KÛ²e<ifff(?Ö¶üôß·¦UÝÔÔTvvvkkk p¹õõõåõ-½ÅÅÅë?þ(?@b_CCCmmmhùÙíö'NhÓòòò(?Ê/ÆçYXÛ@XwÇ^»;Ú¿§±×^ç>pCû¾5­êrrr&&&ç¡ü!iÛ@X·¿QuûäWàôªÐ»téReeePÕExQ¦ü(¿EùùEýW'å'ýGùaõÊozzºªªò`õËÏårY­VýÜÜÜññqmÎ@ ÐÞÞNùQ~1ÊÊÊâ;<X'å'jkk´)555ú#<:;;(?Ê/ògDèáf³ÙëõR~¬Iùù|¾üü|mÊÄÄDffæùóççìÝ²eK[[åGùÅB³ÛíÎÈÈÃáÁ(?Ö¤üz9ÖÎ:ÎÁuæÌÅ®ÊoéçzÒHêÉ`||<È 99òH¨òKMMÎëééÁÑ£GÕOuH´ò«©©ÑçÐïêWXXHù$TùÍ-@&ÞÞ^Hæçç¯ÃßòßFAùÊò ü¢388¸uëÖäädµ·_zzzKKåhåwáÂ ïíPãºº:ÊÕQß´ÉápMQ¯×k·ÛÍf³Á`ÈÎÎ>vì«òúçÁÁA­üzeBù°åWPPàóùBËoÏ=³³³2öûýG°Æ(¿gê¥ú$g>ÏU.?É»ÚÚÚÐòzQöz½êC9@ù-[zzº<±Ôv>)?ùKâðáÃ2ÎÊÊ¢ü5ã91óñã+pò*?ùiµZ'''ÊÏb±?~5(¿/nçÒ¥K¡nxN­HùÉrBËO^+++Êo`` ;;;++Ën·wttô÷÷ó(P~±ÊÏÏWÇöL¦­[·­Ãßò$òú¯:OÊOmÑ¦(N§óÔ©S%%%áÜ¹s¬1Ê/ÁQ~P~.ËjµfhhhîÊò`Ùå7·ðÅªÚ@  9995Fù-ïé:ÎòÖ¡>ÿ«¬æéîÇ×YíÀjÏçËÏÏ×¦TUU<yÒår©êêê:Ä£üb)¿¤Eð©.Àºuë¥M«yÚWËjV³üÃáÐ¦¸ÝîÝ»w«=òÍfó#Gü~?kò¥ü²³³Ï=ëñxÖÿoKùÊÝë7$þb8©áþ«¬vãò¿N:µyóf-ÓÒÒÊÊÊÖçQ½°"¤ü>ýîY°áÊOãóùúúú,KRRV999---@ùªüô:;;ÓÒÒ8ÂH<÷|þëßiò¿¤Æwy´ü|>_ww·ÅbÑØíp8(? 1Ì~èÿíò÷ÿø¯¥ü&ÿ/ýâßì`Ël¬òÝÏÏl6WVV²x&ÿäðG_úúÜ¯Þíõ¹$þî5¥üvéãØ^ QÝ½;ñÞ[ªü®û9|øß»þvVl¸ò[ÙOrÒ/DÓéÌÌÌ4Û¶mëêêråÜ§»×oe«±ç±³2øø«hfåëëE]÷l2***Ün÷J-|×®]ò"«Q)Ëí£8.¿¤¥ÄöIÎ---ûöíQyyùÙ³ó/9uuuòlråÜ?)¿[/ÌýêÝÞ³wßý·z³óeÖ°ÞÊO¶¶¶ÊÊÊZøàà`QQvVÆ/_òÎ ÊïìgjØÌf³úÎA¿ß¯¾g:)Aå×ÜÜü6e9ùÝ?|»®YÊïí¿räsö··ñíá·X3Àr­Zù)F£qVVV¦ÞOëîîñr¯Ê/;wüüüááaýEújÍ òkjj°|o5µä^Êï­ÿå÷ßÚsdøõ+¬ ¿|çÔÙµãîÿ$Ë[@ 99Y§§§åÑf³ÉXßápL¦;v¾/nrÿåÕY###ÚôÅ¾Øk½ÌÖ××§2ô:l¬òÓLNNæææ½¿ôGL4Sx·XA|3°"^¹î~Éóáýd9Þí=úôÁÕÙêêjya[ø2_»Ý®Í¿ÿ~³©©Iff£ÝÞ½«ªª~øaýÄÅ¾Ø~~r»Rê üÂ¤[zzºúziù)ã(§P~ålu´´4	2¯×«.2Íjàóù222´ù§¦¦ÔfjjêÅ&ÆÇÇålÐ'¸-¶ðwUÚ±¸¸¸¶¶Gm£_NNz>É;wîÔ_TYYÙØØ(ùY^^åÊ üS~ÑD¡öþ~þÐ]¤[Zèô%ª¿¿_nQJGm£ú`y6<ôÐCÚ`õìééé¿$ä)ÙÛÛåÊuû7ÞÙRÃIÊON14µ*?m^Ðü333s¼Qù-¶ðwÕn·=ñÄ<jqS~èóü(ÊPîàã¯6¬æéF;ÿôµ,¿êêjË5·ðéiÚüASSSMMMÌå·ØÂ»3£££V«Ur377ýüâ¦üÐçùQ~¬xùy<Í&/Í[Úa¹2CCÑh,--Uÿb»Å¾Øþ%%%ê¹®®.¹i¸ø(¿xDùM)ò[éééªª*ÊÊV~YYYýü#÷óØ¸åúF¾ÙlÖ>CòHò¿$õÜnwFF$ø8@ù$TùiGëHêÉ`||<È@û®@Ê AÊ/55U:¯§§gddDGU>Õ ÑÊ¯¦¦F;C¿«öY«ñ¢îÕÐiGsL¦·ÛÍúß(å'jkkÓÒÒdÐÛÛ+yäçç¯ÃßòP~+C@ ­­­²²õ¿Ê/^P~ÊïþºX> ò£üXÄ+ïÏõºVà$Ë®üdP__=·ðmÅÅÅÁf³ÉXÍàt:óóóebzzzssó²Ê/hu]¸Ìïp8L&Ó;x_8ÊÏjµòIÎ¬ñzÄòkiiñù|2®®®ÔÝnW3X,îîn´µµ¥¦¦F_~§O>xð :vá2ÿþýûeÎ¦¦&G*¾Ë¯¨¨((øí`mËOÛ#Ix©±ÙlV	ÁËÙNÐô´´49íëÂ.ßïÍJÄYù©­³³³ëü·¥ü	Ìd2éÏJêiSôQö:ÇsüøñÊÊJÅ²äþv»pýüëpÃå·<ê;<ââ·¥ü	¬¸¸xddD;;<<¼sçÎÐö»ÕÍjµÖÖÖvttLLLÜOù]¸Ì?333·°Í/==G*¾ËoË-Ú#Jù°Vúúú$õ$Ýd<>>¾k×®®®®ÐV«®®v¹hiiÑ>|×d2JÙíöû)¿°ù>,¦¦¦©ø.?yå¿3ÖüQ~ÄÖÚÚºyófÁ ?Ã¶Çã±Ùl2Onn®¶°££Ãb±¤¦¦>zÉ·ò"Ìvá2CCÑh,--MEßXÛæØJcmÊ/iÛ(¿D+¿8Bù°úøÊòòÓöäc??/?mO>öóHðòG íÚµËétjge,S"EÀ"_Î¶ìXêóÿ@ùQ~¬°ÁÁÁ¢¢"í¬/_¾¼¬b1&(¿U~íçg0ÒÓÓkkk)?VGYYúÞîîn/·ØbK=ÊocÚ«/ôðml·Û)?4®Ù«?¾sÿ'YNÐóóóePPP ÿßéééââbÁ`³Ùd¼dÉlª +**b.?Ô××ggg/vN§Üaµµ¨¹¹çFßììlrrrIIú>ù¹k×®mÛ¶Í-|<äf³ò@3ðõÏ~þäd9¡ß»woUUÕÃ?¬X]]=99)Ã¡m°ßàà`aaa TW¹üZZZ|>ßb÷Áb±H ­­-55çFßÖ­[åqõûýÚ[·nÉ;wÊDõ¶/åÀêß±±1ýDm+DXFFÆbÅ¦'íX\¬ßkk±R]öÉnräcû¹ ±ËO=ð³³¿Þà¬<Ø«ýên#îF.­þþ~yWë"/ßd2éÏÊ«¿6E?gØûàñx?^YYi±X(¿ø(?Évy¨JJJ¼^¯2Voòöõõñn/k^~aßH»½¨¨è'XòÅÅÅúwîÜ:gØû`µZkkk;::&&&(¿ø(¿.ÝüÛÕÕ¥ üXÃò«®®v¹hii),,üFGG¥Éü~nnîûùõõõIêIºÍ-¼×¼k×.u|qÐaïÉdÛÐ¤üâ£ü<[·n/))I~ÊX=´òÊßëç·¥ü³ü<Íf3RrÚö¹GxôööÊ@®´´tÉ[lmmÝ¼y³,_~666½'aïCGGÅbIMM=ú4å7å/(?@ùÝ¬¬¬ììì¡¡!Ê ÁËÏ`0ÄËæYÊP~÷EÉñÈ#LOOkÙCù$`ù-ö½½ÚGõP~	R~IX?àLùÌql/åw¿¦§§«ªª(?VïE]·ÃÉdª¨¨p»Ý+¸pÃ4EÚl6£ÑYWWv$Nùeee©#|ÙÏ5,?mÚÚÚ*++Wpáú¯ñÕnnjj*;;»µµUnÔårÖ××S~	[~¹¹¹¡wÍfõ5¾«_~Ñh¥ÉÙÚÚÚÐìvû'´éCCCyyy_Â<«äqu»Ý2às8÷ùu½ê»&:ÎÌÌLÁ°mÛ6õÑL¡üP7<7^ÍÓÕ+°ÚMÓØ?çÿxÿ'YNVÉÉÉj<==]\,¯6MÆÚüòm2vìØú¾phùÉO«Õª¯6CNNúÒÞhbq_~Ú×üIêÉ`||j2ÐmË5;;++>WÊËËÏ=+ººº(§_ûGÀ÷ÚCG~ïÔjv°Ú¡^^þ¨óg×¿ÿ,g±Ì×âÓ§O<xP­®®VÅ&©g·Ûµù÷ïß/s655ÉÑß¥K´wµ"|åå*kOOÏÈÈ=ª1ªË±cÇxâÐçÙlVí÷û³²²¢T~òÏ @¬¤á9õÖ³ýn¯&--MbNÛóJ^ÕÀçóeddhóOMM©My5]FßpR~ÒßÆ-¿íxý³¤°°0¥MLLäçç«­AéXjÍÞíVßÀóÃ¬`¾¨/YaÁÔÏú¢vp¹«U?%77w||¹±½½òKØòµµµò·ä¯ÈSJê-¶Evww®èV»¬F3ò(?òÓ6éÍ?333·°Í/===ÊòS¯ûÚý_"ßÊ>k6/käI)OMý4)°"Ü´|µSÊïkúÔÏy=p7À:â¨üª««].ZZZ´7ådþÃË ©©Iê-úòóù|ùùùÚÌÌÌóçÏÏ-Ø»eË¶¶6Êò»ß§oeeecc£ägyyyS(?àþõ¿wÌöíòáÌË§÷9Zu±Z8*?Çc³ÙCnnîÈÈ6CCÑh,--Uÿ¢_¸úí¬Óé,((ågee9s&»8+¿MK¹ÏOrÖ?WÔ¸§§'##C+X¨d£Bù÷ï÷õÎùõn¯ÿöÇJ¾#9ÈµAù-Z~IùØÞòb :O½½+å÷Ós2è¬ïýÙ3¯³rÊ®ü²³³Ï=ëñxÖÿoKù1õÏÿOÿ¬ßË­epþTÏË­WX9@oøÀ+?·ÛêÔ©Í7ë?:¨¬¬lllòLCõ³jSz·÷æõÛµ»¾uík6Jùi|>___ÅbIJJÒ*0''§¥¥òâãÿé;¾Ö-å×öøOþGùw_zªÕ±üô:;;ÓÒÒVäÊXWn^¿ÝY?ÿß:ÐòÆ¿`À-?Ï×ÝÝm±Xôöfgg;ÊH0|3lÐòÝÏÏl6WVV²@ùXõcuÐÑ»²Ø í;úµÙlF£133³®®îAß¬RùíÒÇ±½@õÏNOÍÄpò»øWb¸âÍë·Yí@"_AAÏç½¡©©©ìììÖÖÖ@ àr¹ëëë)¿*¿ôIÎðà¿ö¾4ÜjøÎ ®Ë/ôÜjkkCg°ÛíúïðÊËË£ü¡üÂ'9ëÖíIü­æÉ=ñ	«Ð|áK?ú¼ýüýd9Qêëë³³³e<==]\,/Ó6MÆj§Ó/ÓÓÓ,?ùiµZ'''fÈÉÉ2LOå(?ÀzàþäÖî÷åD_~---êýÙêêjUlÃn·«,Kww·ÚÚÚRSS£)¿K.UVVÍa»åGùQ~¬ÜzHZi^ÉE@@Íf³HfddDXNØ½¹ô3HùIÿQ~åÀj3Lú³zÚuÝ)ßãñ?~L[ähÓÎº«Õª;>>®¿íííåGù°ÂGFF´³ÃÃÃ;wî­®Ð7sçvÚ«­­íèè¾ü¡¡ARSS£?Â£³³³  ò£ü(?VX__¤:Àb|||×®]]]]¡ÕU]]ír¹dÐÒÒRXX¨&L¦ÑÑQ¿ßo·ÛU~>/??_"·yþüù¹·lÙÒÖÖFùQ~+¯µµuóæÍA~666m5Çc³ÙdÜÜaGGÅbIMM=úô4ÃáÐOq:²ü¬¬¬3gÎ,v-ÄYùñy~¥üôÝ¶üø<?)?Í¤óöìÙ333#gågIILQDùëÐð'¶ý=-øÅgc¸â·¾wÕP~f³Y:ovvVâ÷ûeJØ¢üõàC÷Íï>7ÃIÊO2.+þìç¬vHòSïíJíûùGÊ¯ó§ï²`ã_zzºt^yyù­[óß!ãõzwïÞ-Sd:åP~*¿îîî°GxP~@"¼æòkxæÿÎ]ÖlÐò[ø¸ÈÉwíÚµuøÛR~@l$õÿö«ÿù¯~(å÷Åÿþ£²¿üÁÛã°Zõf×®]N§S;+c)ùZÞeÇÄ¦M#h6µÙlF£133³®®.ì<³òÉ¾¿yì¥Û¾YõnoÏ«®ÏýEû'×¬`],**ÒÎÊøòåËmebbÓ¦Ïºä©©©ìììÖÖÖ@ àr¹ëëë)?ÊòÖ5é<÷'ó;òJù]xiþ»Ø¿òäÏ~ü³	Ö#ïgs×o¯Ài&ø°²²2õmÝÝÝ2^²ØbN½ ³µµµ¡3Øívý×øåååQ~q_~òGÆÖ­[Õñ¼ééé--- ¯P·î|Þ~^¥üúþ2øîsCßëxÄæÞ·zîãÅ83øEmxx8??_Ú7³éééââbÁ`³Ùd¼dÉlª +**,?ùiµZ'''fÈÉÉQß#¼Ý5.?õIÎúolScýù×Êþò×¼sºcíÿØÝóª5¬C÷î­ªªzøáõ«««U9»Ý®µ×bûù)H­ç"ß¥K*++fð^_¼_FF<xòÑÊ¯··WÆ)))Zùÿî÷'·TùÉÙ/|éGá¬Oãããò*<66¦h6ÕÀçói_µ¹½¤ÞÃ[Úr¤ü¤ÿ(¿/?ý¯ÊOþDàóÝçþhÿsR~òßÚ¥Õ&@ëôÕ=$ªô­¦ÕBäöêïïrýq-_;ër¹¬V«~Jnn®¨6§B;åßå§>ÉYmç'ßï?|ø°³²²(?`òßûèærOþÉR~Ï=;øÐ»ì«Ï|ÆZÖ°üRSS£MÏn·=ñÄÑ¨­­mhhÐ¦ÔÔÔèðèìì,(( üâ»ü.^¼vó¯ÚÞKùëÑÿÜÏ§b8Í¿ÛÛ8Ëu¯^g­kX~ÕÕÕ.×ü¾¹---K¶×èè¨Õjõûý¹¹¹Qîç§ø|¾üü|mÊÄÄDffæùóóGmÙ²¥­­òïò[ø´yÕ±½&iëÖ­A»P~@b/¿ó#¬ îÊÏãñØl6Á %§óáÞÞ^tuu.ëæ~Óé,((ÎÊÊ:sæL;¸)¿xAùÊÌ<þíWc8IùÉ)+>ß=ÎjD(?ý¾¢Êµk×¶nÝºeËÊH°òüX÷ã_òÊï×ü~?ÇöæÓÏæÞøh®×5÷êäÜ»×ç÷X%°ÊoË-"åÄ¥OnÏçÖ|ùpcnäãù`ãßÄÄDÒíÃô$ûZ[[)? ALÍ]_øL/)¿nÎÝÓMlòÓHç­Ã7v)?`ÅÜ½7ÿ±,Úæ'ÞûôÀ*¿°¦§§«ªª(? îÍõ0wïWå÷áÂ·w¼n¯ñÖÙºî[:L&SEEÛí^Á; )jàõzív»Ùl6ÙÙÙÇã±HðòËÊÊ;h?¿ Ã>(? 47yãå÷ÑÍ¹;9çäÜ­;¬`½ß¯ÿdÚÚÚ*++Wpáú/sÓnnÏ=³³³sGyYÀÃ°åzx¿ü@ù	âöùÍ~ãÓóå÷ÎôüN~ïÏ°Võ Ñh¥ÉYÉ»ÚÚÚÐvúHKKãáHØòg<ön·;##Còx«ï>pàå$;¹OçËoðÚüA¾îÃõOF>ùøÍû?Ér"´Z HNNVãéééââbI4Í&cm~yÉ6L;vì_8ìW´Y­VíËÜ´,ËñãÇyX7Jùi_ö"©'ññqyªÉ@¶Q~@âPïö¸?®«]®w;Wàtµk±V×âÓ§O<xP­®®VÅ&©g·Ûµù÷ïß/s655ÉÑß¥K´wµ²³³³²²dÉýýý<Ä ©©©òØ÷ôôÈàèÑ£jÛ¿êÛý¶mÛ&ËÔ_ät:333ÕE]]]QN¡üÊØ(/ê:iiisÚWf³Y|>_FF6ÿÔÔüû~¿_ûÞÅ¾ÌWë<)?é¿Ð4àS§NÈ«ð¹sçx8¶üjjj´ã9ôÏÂÂÂ&ÅÖÖÖ¦þªÐ¦JyyùÙ³gePWWWQQå ò»xñ¢ï³;ÞÛ1¤üî¾ûIWôßô±Öñ Ë/(ÔÁÔÏº½&ì6¿ù.ÕjpsCCCYYY4VÂ¨­­UûröööÊ@Rùùù÷¹ÌóçÏçææê§Hõ§zJE3%¨üüñçïzæ·Þ­âéýîAV; ÖªüÂ~±Ì?33£^4ÓÓÓ£,?õºßÐÐ MIIIQ/¾u¸ÇV²üVÖìì¬<äùÔÜÜ¬®ÿsD£Â»½@¨;³<óþjnÜúÕ¬Â»½]T]]ír¹dÐÒÒ¢½)'ó>|XMMM555ÑÏçËÏÏ×¦TUU<yR-_.ª««;tèå·<.úûCÿê0õh¦P~@¨O_ûâÏWóô_ü#«XÃòóx<6Í`0äææhó744ÈËeii©ÚøýÂÕçx¨±ÛíÞ½wrr²¼Íæ#G<è÷µ±Æå·uëVõÙ.+ûIÎAí$Õ3IÛ(ÍÊu'àÿØ7ÃI®ý½ïÄpÅw®³Ú8*EP~ÊÉÉÑ×&¶ceis»îÜ¹SQeeecc£ägyyyS(? LùùÞp¿÷Ç1¤ü.¼YÃoxN±ÚÊP~yòÔéëëÚ»36N§sÛ¶mREEEêPsí©ÙÓÓ!7)]åÊ5ç½á$å÷ü¿ÃoßxÕ¬7÷óØ¸åg6¥ÌV$û4ÊPIÿùo.ëäíøÚsÿUÊÏñãí­_^îÕg/ÿÕP~N§SÊïàÁKîJùë¥ü·O~1úÓ§'>ÿÆW÷êñR~/<mª-xÿ1Û²àïü«¡üÄæÍC?ïûþð üuâñ7þòGçfýãóå×÷¹ÿGÕ¯ìº=ÁW~ÙÙÙ+xå¬7wþ¿rþûê©±ß?Âã§¿;5ö[M£G.N=ËÊW~ªù._¾¼þ[Êïîíçp»þä^àæ|ùõýéí?xêõü&Y9ÀúzQ9V÷½+u8aoÈëõÚív³Ùl0²³³;ÆãhåÎ@bû¾ÿëõWþáæßþùwÉ½ùí¿:Ü÷Ðk<É6lùø|¾ÐÚ³gOCCÃìììÜÂgëYÀCPåwñâEy¼kjjôÏÊH»¾xåwvÿûWßjßæ×ûÇßøqÙîß»þ1ålòý&7É»ÚÚÚÐöõòz½iii<4	U~ÁÀºõê¯Zû­NR~?÷Û1èóËj4ïu¼úüã÷åDY~2¨¯¯ÏÎÎñôôtqq±$Íf±ÁétæççËÄôôôæææ%ËO~Z­ÖÉÉÉ ,ËñãÇy¹üÁÀºõñÍ¿?Øå©íçßkþßzê¥mWR~ßì(hyõ³?ú?Z9ýB^Õh>~Ñ3Øyÿ'YNôå×ÒÒ¢Þ«®®VÅæp8ìv»VlÝÝÝ2hkkKMM¦ü.]ºTYY4ÃÀÀôeVV,¹£££¿¿;ÑÊ/P~@,wo~iû´ëÏ§Ææ·ù=ÝõNý÷ûqçgÛ¬³õòÓÞÓïo6Õ@B0###ÂrÂ¾§§AÊOú/ô¦Nç©S§JJJÃ¹sçxh(?Ê'¾3>îú<¿ïýäî~4r³æ÷î÷X3Àºb2~ã¯¶@@¢/³°»fy<ãÇKÉY,%÷ÔÎº«Õ:·øCCCYYY<4P~ÚÓýü¸óÎ¡Ã?/[ÆéÕÏýÍÅíý¤üäTÓUø7½¶e-¡åÝÓ¬vàA+..ÑÎïÜ¹34ÝBßÌ[Øi¯¶¶¶££cbb"úòr­mJJJJÐÇ$''óÐ$Bùiò±w^ü°ªÝýéÏ¿ïØóLÃ¾wæÿ÷m»þìßú¯Ï=µ¬%|ãåYíÀÖ××'©'é&ãñññ]»vuuu¶ZuuµËåAKKKaa¡h2FGGý~¿Ýn_Vùù|¾üü|mJUUÕÉ'Õòå¢ºººCñÐ$BùÅ#ÊP>ºþî+ÿýÉÑýÅïuïí»RûþÈ¿-ëÇý_ª¿ð¹K¯?ýÞkgµ« µµuóæÍA~666m5Çc³ÙdÜÜaGGÅbIMM=úô4ÃáÐ¦¸ÝîÝ»w''''%%Íæ#GHMò¸$Tù¾±íÚµ­[·nÙ²òÖ§;¾7Üïýqô§®ÿ·¯þ>xõ»?GÎ×-#oF¿S¬vHÌòº¼Û¬[÷f>¾Ó×åéÆOÿø¿§ÆÞ¿ø7ÿîeÐÙtðb³=úÜÕq [¶lÙQØH)?`=¸;Ú/·§Ï²Ú ËobbBÉ¡É«'Ù×ÚÚJùá_^ùîïüÃ¿xºÏòØo=|ö»;¿~üÎ|î³ÙÏX3°QÊO³>ã¥üTßý/yú÷ë¶Kùýû¯ýÙþóÿÒ8zýV5®üâåÄfÿÙk7|ó'åwìGßÁ×|ýÒèmÖlÄòSÇoÏ-|2Éd2ååån~øË§?Rc)¿£´ößhÝËÊW~öìQûùý÷î¥üÄðçM×>ütVßc/<#Úx^yÇÇÖ]»v9Ní¬eJ¤Xäky69 )jàõzív»Ùl6ÙÙÙÇãaûòKII¸¯¯oxxXÅårÉ@¦S~Àú4rí­/8úÓûÏ:õù=Oý)?9ÉÙÿûÉÏïzKøç¾V;ð ige|ùòåÈÅ¶21±iSAAÏç]ò=fgçÿtôûýGðHÅwùi"È3Lösá>äòÖqÏøÑçÿvY§=MÿIûö¯Ùw|ý¯ÿã7ÿºæûÿYWöJ+«XeeeêÛº»»e¼ä+xÌ/ýAg%ïjkkCg:Ôëõ¦¥¥ñ0Åwù%''Ë<==­6þMNN^¼xm~@âñÝ¹Wñ­©¼~ó.õ÷çÉ=ÍOüÉ¿ÜÿékúTÐóóóePPP 3éââbé0Í&ã%ËOfëëëSYQQ±dùÉO«Õ*4Åb9~ü8xBßîÝ»µ222æ~µýüuËÿén§¯?yúåï'+^ý«ÐÜðÜ¹ÿÓ§9ÐJ^«ªª~øaýÄêêjeC½;7q?¿ÁÁÁÂÂÂ@  ©õò»téReeeÐÙÙÙYYYr£ýýý<úq_~ê¤¤$É>õg#ïRJùkëæäðõá4ðØÈ)+¾ÿâ·XíÀêðÓO4ÍjàóùÔf¹¥Þív,..z7l)jËòþ]²Óé<uêTIIÁ`8wîQÜ_¼ üeòúl÷õNÇ¾þLWüÉ[·XíÀê½º$¾Õ´ñ#_¿þ¸Å¯u¹«5Â²²²x(?Êòkmi£ü¸+¿ÔÔÔhfÓ³ÛíEEEO<ñDôå'jkk´))))Àoì¬>ñW~AGïF>KùAÊÏ3ØÉzâ®üª««].×ÜÂw..Y~£££V«Õï÷çææF¹âóùòóóµ)UUU'OT7-ÕÕÕ:tò£üõîÆßnþ+)¿+§>çºx:0ëgqT~Çf³)9íßGxôööÊ «««´´tY7çp8´)n·[ÑTÙl>räÔ$åGùëÚ'Ã/JðMôHù]µõç¾ò/ßÜeÍåGùfèÌ«s¿z·WO ü(? qÜõyß8ýÕXÊïÃç?á6^s¶°rò£ür/pw°®,à¿­Êïã×:dð^çÉ¯<ÏÊÊòÍ;ÏE³z·÷³éÉËOî¬Ø@åå$;7?y³ác­+å÷ÎsåÔçølØXå´Á@ù	#0ëw¿Þ1_~­wkêmVl¬òGpø$g ü(?òP~°.Ýõyo¼w9ßÕçá>Ï¬v ü(?`¨[Íô"«(?ÊXY¿ÿÓWó4ûSV;P~ÀF-¿ÞÞÞ¼¼<Áët:õÉÙÌÌL¹hÛ¶m]]]QN¡üÖiùåäätIÍÍÍ[·nÕ_T^^~öìYÔÕÕUTTD9òX§å§g2ôgÍfs ßïÏÊÊrJPù8q¢5Byò¯¯¯ïÀú)ú/Qãh¦_ggçMÖåÊ/òòr¯×«¨ÿ"`£Ñå òãÝ^@ù­#ûöíî÷ûçÞÉqS(?uZ~/^Ü¹s§Ûí½¨²²²±±Qò³¼¼<Ê)À:-¿¬¬¬M:¿¼¤¤¤ÌÌÌÞÞÞ(§P~ë´ü(ÊP~åGùP~åGùëÝÍkásþÕ<Ð7ËjÊòÖÀõñ@ÏÛ1-öÊ)+ü«ÕåÄÉ¾«?¾ÃzÊò(?åGù	T~ï¾@ùåGùñã3ß'®w;uz«÷BßSç/5ü°·ñíÏ_]ÞÕ?q¿ÁjÊòÖÀí[½3ò¯ÑÞìkéow¼ýzËþ¯Í^|fàùgµ¦^eµåGùqàù/ÜþE@£=2øÉÿwëÃ»¬ ü(? ¡Ü¹yOÊO¥üÞ¼pEÃçü£Ï±ÃP~XîÝûáçoJÿ©òûÅùòøúgêå$ ù®´]¾Ò åçlkjÿá<Þ¬ ü(? ÑL½ûó×ºútIù½zöµ5¼0ôÊS÷îQ~@ùQ~@ÂyóõzïõkãîÌoók|ú_?ýéô(k(?ÊH(wï~6|¥A¥üâÁ/óA-@ùQ~@zóòé?U~o¿4Çã¦=o±fò£üõÎ7ïêïDzí¯|oàÝ.¿_ß·^ûüG½ßé|çG7¢_ÂÇoòá@ùQ~ÀZpÞ¶ØÃIÊ¯ëèk1çO~ÆjÊòâÆO¯Jù½7ÞyÇµå$8)¿O>~õåP~ÊòÖÙ;7%àb8Iù½3ò¯1¦wÕå¬ïùoãXÍëÝNV;P~°^¹îÞòâs16=ßô?ýèé®ø¥·~ÎjÊòÖÀÕÛÞ¯^á$å'ÃÛ?|ÕåÄÕÏ7÷ý1ÖP~@ù(?ÊHc7oHùðø*6 ü(? AIêí»ÒõV)¿ßùiÆÅýù§VP~$ûv9»¼³³êÝÞg§&Ì?~æÃÏn³fò£üD#÷¾ïÖÜÂ~~ÿâúþd ûéÉqÖP~P®ßñoyñ¹_þñ|Ó?üâÊü¿¦ÑË¿ó+(?ÊH4ÿuìæ9Ý±½;^yáÙ©	ÖP~hN¿ùï.ýðß-U~rö·Ú9Â(?ÊHÐ>£ÿçÎIù¥_lTå$&ulï?½34ïk(?ÊHôÿ2ø ü(?òP~°x?Ýw¥7b¸âw>@ùQ~@Ü_Ì'Ê(?Ê`cÛíÎÊÊît:333Ã¶mÛººº¢Bù¬Óòëéé±X,6¹cååågÏA]]]EEES(?uZ~6mdd$lùÍæ@  ¿ß¯6F3%¨üÎ9ó*kòåîn+?Á4fJPù=ûì³¬Ê_´å¤FcSÊwååî÷ûçÞÉqS(?ø+¿ÊÊÊÆÆFÈÏòòò(§P~ñT~êlOOOFFFRRRfffoooS(?u]~å(?Êò£ü(?Êò£ü(?Êò[ÿ÷ÿÌ3Ï`~øå·J&&&¾ò¯|5Â[O ü@ùòåÊòåÊ¸ÿá7ÆmÛ¶<yR?Cèµ?ÎÚÊ@þl èîîÎÉÉ9úôbåGöå ÊOø;Ù*?a0Bg ûòhå×ÓÓºÍO²¯¦¦5)?Ï×ÜÜÞÔÔ¤A²ïàÁV«58.?½ÔÔÔòòòÎÎNýÚ¼Ç;qâ+(?ñZ~jì1ÍæñxfÐöíyòóóÇÇÇYo@ùãòS;öðÃGa```çÎ¬7 üÄù		»ººº3ÔÔÔh(?q n·Ûb±LLL,6×ëÍËË»vík(?ñ]~bpp°¤¤$Â.¬¬díåÊ(?P~ ü@ùP~ ü@ùòåÊ(?P~(?P~ ü@ùòð@pÁf³%''ÔÔÔ½÷?ØÿP°æò°ª7HIIy ñGùå`lÞ¼Y"¬¶¶vffFÎNLLØl6RZZÊÊÊ@B1Òyú-|.K¦L¦_þã_pþüùÜÜÁ°mÛ¶ÎÎNýä¬ÅbdMQWWW^^KÛ±cÇøø¸~K.Áãñ8p %%E.2Íòz½<d@ùQii©D¤Õ=:::@ð?þIIIú·ûúúÔ¥/_^ì"§Óô&²¶Q_~PRR´òå Fn·;//O¿ÔÕØØXPùOOOÏÌÌ¨RÔN¦ËÙ®®.÷ôôÈ¸¢¢B]TVV&g«ªªü~¿º(999´ü",AáÄÄ¥JõKP~bÔÚÚ*1g0TL¦îîn¥MMM©³2P¨ÎÊ ôèýEaßÕ_%¨$ÍÎÎ|lkk»uëP~VÆììlQQôTWh¥iS¤õê%%%-vÅ°åa	###AÛ#åîñ0å Ff³Y¢J;öBÐ¶Óf¸ví~³²õûý¡KVóÔ!ÃÊ/ÂþþþGyD½ËÉC8p@jûöí###rVBíðáÃ2eÛ¶múJ³Ùln·ûÖ­[rvÏ=êRd^¼pásssÕEj?¿¹¨««KÆ[¶l	-¿KPûùuttÌ-lÿ±Ñhä!Ê@&&&ÒÓÓCßrmmmÕWÉdÒ.üÒ>¦¯¯/è---ê¢Ðcì±Ðò° öïßÏCØïÛ·/--MÍfÓbJ®K.ååå%%%åçç÷ôôè¯~þüy(eff9sF,G]K.ª­­ZæKðz½ÎÈÈåîÙívÏÇãöoZÊå ü$ãÖP~ ü@ùòåÊ(?P~ ü(?P~ ü@ùòåÊõõÿK8+»üzÿ½^ïÞ½FcZZZuuõµk×d¢ßïû]¸pÁ° ³³SM*,,°üÑÑQÍ&ËÏÌÌ¬««[µßå¤üâzùv»jjJ@@ÊÌjµÊXÂn÷îÝ¡3«æ2PSÊÊÊ.^¼¸ØÂeÉÙÙÙ­­­²pË%X__Où üP~k³üäädm,f2dÐÜÜ|èÐ¡wF·oß9+O8¡ÊËË£üP~°üä¢úúúììì ±ÛíÞµkÁ`Ø¹s§Çã	9h!cccL2³:ùùù²ôôt©´Ð%¨·ersszz"ÜU£Ñ(Á§Æ~¿_ÎÊ ªªª¥¥%tæ m~%%%ÚÂÃÊÉÉX¢@ùÀ)¿ÅöóôÏçKW555©>;pà@èÌAË¯®®8ëëë+ª¥»»[mmm©©©¡K2SEØÕÕZz÷î­««åËeùêÎïÞ½[Ú4yÜº×ëU3«ærÏ"¯íMaÊå AÊ/ÂEÚæ4ý8--Må§¾Û´¢m+F¸ú%HºíÙ³G²ovv6òýÿÿÛ·Cå¡ãÁ Y1aÅ0Ä4ðDï@o@vw`°YâÈÁ¼00Áä÷ç;|c¸éûÖ_ÇyÎlÛÎõzm·ÛJ4Û¶W«¹ç§«2á¨	ªÃäµïûÉ=ÊÀßÇQ½'õdµàx<V±9óòî]ty ÏçO§Ó/ÿKÅb19nr0îp8~É=qÕj5Ãè£:RqIù ü|QùYenÅ©Tf?ßý~7'3X«ÕF£çyçó9µü¢ÒÍf©1j¹:ä$Éòk6¾ï?>^ø¢ëºÊÀ_¯×3ÏRUBñ÷üÞM2u°Ýnûý¾ÌårA<¤¶ã8ÍæùïÍ¼×¯3|:ÃP=w<Í 3Ù7Ï_ÞÜï÷Ñ~ïù)LÕëõúùwc¯mÛf-Êåà-¿;<RËïr¹Ôëu¥R£Ñïí7¿ºªT*µZ­Ûíf=ÏSY5LRSU*L&cvc|XB©çº®N+ËÅÂ*+uÙlVÙívãOlE_ív;sßózýº3¿þÂt:ýM1åÊåÊ(?P~ ü@ùòåÊàëüìª9åºk ×IEND®B`


Species = RP


Between-Subjects Factorsa	
	Value Label	N	
Species	2	RP	42	
NitroS	1	Pep	3	
	2	Urea	3	
	3	YeEx	3	
	4	NCl	3	
	5	NS	3	
	6	Pep+YeEx	3	
	7	Urea+Pep	3	
	8	YeEx+Urea	3	
	9	Pep+NCl	3	
	10	Urea+NCl	3	
	11	YeEx+NCl	3	
	12	Pep+NS	3	
	13	Urea+NS	3	
	14	YeEx+NS	3	

a. Species = RP	


Descriptive Statisticsa	
Dependent Variable:   Bio  	
Species	NitroS	Mean	Std. Deviation	N	
RP	Pep	2.80200	.203443	3	
	Urea	4.63400	.139097	3	
	YeEx	4.12700	.089152	3	
	NCl	3.05833	.028378	3	
	NS	1.33567	.087888	3	
	Pep+YeEx	1.61533	.129778	3	
	Urea+Pep	1.86833	.106214	3	
	YeEx+Urea	1.33633	.111433	3	
	Pep+NCl	3.46167	.205510	3	
	Urea+NCl	6.56500	.323561	3	
	YeEx+NCl	4.14733	.077184	3	
	Pep+NS	2.92867	.161401	3	
	Urea+NS	3.46833	.257250	3	
	YeEx+NS	2.66100	.104504	3	
	Total	3.14350	1.412249	42	
Total	Pep	2.80200	.203443	3	
	Urea	4.63400	.139097	3	
	YeEx	4.12700	.089152	3	
	NCl	3.05833	.028378	3	
	NS	1.33567	.087888	3	
	Pep+YeEx	1.61533	.129778	3	
	Urea+Pep	1.86833	.106214	3	
	YeEx+Urea	1.33633	.111433	3	
	Pep+NCl	3.46167	.205510	3	
	Urea+NCl	6.56500	.323561	3	
	YeEx+NCl	4.14733	.077184	3	
	Pep+NS	2.92867	.161401	3	
	Urea+NS	3.46833	.257250	3	
	YeEx+NS	2.66100	.104504	3	
	Total	3.14350	1.412249	42	

a. Species = RP	


Levene's Test of Equality of Error Variancesa,b,c	
	Levene Statistic	df1	df2	Sig.	
Bio	Based on Mean	1.609	13	28	.142	
	Based on Median	.565	13	28	.860	
	Based on Median and with adjusted df	.565	13	12.482	.841	
	Based on trimmed mean	1.520	13	28	.171	

Tests the null hypothesis that the error variance of the dependent variable is equal across groups.a,b,c	
a. Species = RP	
b. Dependent variable: Bio	
c. Design: Intercept + Species + NitroS + Species * NitroS	


Tests of Between-Subjects Effectsa	
Dependent Variable:   Bio  	
Source	Type III Sum of Squares	df	Mean Square	F	Sig.	Partial Eta Squared	
Corrected Model	81.025b	13	6.233	233.441	.000	.991	
Intercept	415.027	1	415.027	15544.577	.000	.998	
Species	.000	0	.	.	.	.000	
NitroS	81.025	13	6.233	233.441	.000	.991	
Species * NitroS	.000	0	.	.	.	.000	
Error	.748	28	.027				
Total	496.799	42					
Corrected Total	81.772	41					

a. Species = RP	
b. R Squared = .991 (Adjusted R Squared = .987)	


Estimated Marginal Means


1. Grand Meana	
Dependent Variable:   Bio  	
Mean	Std. Error	95% Confidence Interval	
		Lower Bound	Upper Bound	
3.143	.025	3.092	3.195	

a. Species = RP	


2. Species


Estimatesa	
Dependent Variable:   Bio  	
Species	Mean	Std. Error	95% Confidence Interval	
			Lower Bound	Upper Bound	
RP	3.143	.025	3.092	3.195	

a. Species = RP	


Pairwise Comparisonsa,b	
	

a. Species = RP	
b. This pairwise comparison table cannot be constructed because Species, the factor being compared, has one level.	


Univariate Testsa	
Dependent Variable:   Bio  	
	Sum of Squares	df	Mean Square	F	Sig.	Partial Eta Squared	
Contrast	.000	0	.	.	.	.000	
Error	.748	28	.027				

The F tests the effect of Species. This test is based on the linearly independent pairwise comparisons among the estimated marginal means.a	
a. Species = RP	


3. NitroS


Estimatesa	
Dependent Variable:   Bio  	
NitroS	Mean	Std. Error	95% Confidence Interval	
			Lower Bound	Upper Bound	
Pep	2.802	.094	2.609	2.995	
Urea	4.634	.094	4.441	4.827	
YeEx	4.127	.094	3.934	4.320	
NCl	3.058	.094	2.865	3.252	
NS	1.336	.094	1.142	1.529	
Pep+YeEx	1.615	.094	1.422	1.809	
Urea+Pep	1.868	.094	1.675	2.062	
YeEx+Urea	1.336	.094	1.143	1.530	
Pep+NCl	3.462	.094	3.268	3.655	
Urea+NCl	6.565	.094	6.372	6.758	
YeEx+NCl	4.147	.094	3.954	4.341	
Pep+NS	2.929	.094	2.735	3.122	
Urea+NS	3.468	.094	3.275	3.662	
YeEx+NS	2.661	.094	2.468	2.854	

a. Species = RP	


Pairwise Comparisonsa	
Dependent Variable:   Bio  	
(I) NitroS	(J) NitroS	Mean Difference (I-J)	Std. Error	Sig.c	95% Confidence Interval for Differencec	
					Lower Bound	Upper Bound	
Pep	Urea	-1.832*	.133	.000	-2.105	-1.559	
	YeEx	-1.325*	.133	.000	-1.598	-1.052	
	NCl	-.256	.133	.065	-.530	.017	
	NS	1.466*	.133	.000	1.193	1.740	
	Pep+YeEx	1.187*	.133	.000	.913	1.460	
	Urea+Pep	.934*	.133	.000	.660	1.207	
	YeEx+Urea	1.466*	.133	.000	1.192	1.739	
	Pep+NCl	-.660*	.133	.000	-.933	-.386	
	Urea+NCl	-3.763*	.133	.000	-4.036	-3.490	
	YeEx+NCl	-1.345*	.133	.000	-1.619	-1.072	
	Pep+NS	-.127	.133	.351	-.400	.147	
	Urea+NS	-.666*	.133	.000	-.940	-.393	
	YeEx+NS	.141	.133	.300	-.132	.414	
Urea	Pep	1.832*	.133	.000	1.559	2.105	
	YeEx	.507*	.133	.001	.234	.780	
	NCl	1.576*	.133	.000	1.302	1.849	
	NS	3.298*	.133	.000	3.025	3.572	
	Pep+YeEx	3.019*	.133	.000	2.745	3.292	
	Urea+Pep	2.766*	.133	.000	2.492	3.039	
	YeEx+Urea	3.298*	.133	.000	3.024	3.571	
	Pep+NCl	1.172*	.133	.000	.899	1.446	
	Urea+NCl	-1.931*	.133	.000	-2.204	-1.658	
	YeEx+NCl	.487*	.133	.001	.213	.760	
	Pep+NS	1.705*	.133	.000	1.432	1.979	
	Urea+NS	1.166*	.133	.000	.892	1.439	
	YeEx+NS	1.973*	.133	.000	1.700	2.246	
YeEx	Pep	1.325*	.133	.000	1.052	1.598	
	Urea	-.507*	.133	.001	-.780	-.234	
	NCl	1.069*	.133	.000	.795	1.342	
	NS	2.791*	.133	.000	2.518	3.065	
	Pep+YeEx	2.512*	.133	.000	2.238	2.785	
	Urea+Pep	2.259*	.133	.000	1.985	2.532	
	YeEx+Urea	2.791*	.133	.000	2.517	3.064	
	Pep+NCl	.665*	.133	.000	.392	.939	
	Urea+NCl	-2.438*	.133	.000	-2.711	-2.165	
	YeEx+NCl	-.020	.133	.880	-.294	.253	
	Pep+NS	1.198*	.133	.000	.925	1.472	
	Urea+NS	.659*	.133	.000	.385	.932	
	YeEx+NS	1.466*	.133	.000	1.193	1.739	
NCl	Pep	.256	.133	.065	-.017	.530	
	Urea	-1.576*	.133	.000	-1.849	-1.302	
	YeEx	-1.069*	.133	.000	-1.342	-.795	
	NS	1.723*	.133	.000	1.449	1.996	
	Pep+YeEx	1.443*	.133	.000	1.170	1.716	
	Urea+Pep	1.190*	.133	.000	.917	1.463	
	YeEx+Urea	1.722*	.133	.000	1.449	1.995	
	Pep+NCl	-.403*	.133	.005	-.677	-.130	
	Urea+NCl	-3.507*	.133	.000	-3.780	-3.233	
	YeEx+NCl	-1.089*	.133	.000	-1.362	-.816	
	Pep+NS	.130	.133	.339	-.144	.403	
	Urea+NS	-.410*	.133	.005	-.683	-.137	
	YeEx+NS	.397*	.133	.006	.124	.671	
NS	Pep	-1.466*	.133	.000	-1.740	-1.193	
	Urea	-3.298*	.133	.000	-3.572	-3.025	
	YeEx	-2.791*	.133	.000	-3.065	-2.518	
	NCl	-1.723*	.133	.000	-1.996	-1.449	
	Pep+YeEx	-.280*	.133	.045	-.553	-.006	
	Urea+Pep	-.533*	.133	.000	-.806	-.259	
	YeEx+Urea	-.001	.133	.996	-.274	.273	
	Pep+NCl	-2.126*	.133	.000	-2.399	-1.853	
	Urea+NCl	-5.229*	.133	.000	-5.503	-4.956	
	YeEx+NCl	-2.812*	.133	.000	-3.085	-2.538	
	Pep+NS	-1.593*	.133	.000	-1.866	-1.320	
	Urea+NS	-2.133*	.133	.000	-2.406	-1.859	
	YeEx+NS	-1.325*	.133	.000	-1.599	-1.052	
Pep+YeEx	Pep	-1.187*	.133	.000	-1.460	-.913	
	Urea	-3.019*	.133	.000	-3.292	-2.745	
	YeEx	-2.512*	.133	.000	-2.785	-2.238	
	NCl	-1.443*	.133	.000	-1.716	-1.170	
	NS	.280*	.133	.045	.006	.553	
	Urea+Pep	-.253	.133	.068	-.526	.020	
	YeEx+Urea	.279*	.133	.046	.006	.552	
	Pep+NCl	-1.846*	.133	.000	-2.120	-1.573	
	Urea+NCl	-4.950*	.133	.000	-5.223	-4.676	
	YeEx+NCl	-2.532*	.133	.000	-2.805	-2.259	
	Pep+NS	-1.313*	.133	.000	-1.587	-1.040	
	Urea+NS	-1.853*	.133	.000	-2.126	-1.580	
	YeEx+NS	-1.046*	.133	.000	-1.319	-.772	
Urea+Pep	Pep	-.934*	.133	.000	-1.207	-.660	
	Urea	-2.766*	.133	.000	-3.039	-2.492	
	YeEx	-2.259*	.133	.000	-2.532	-1.985	
	NCl	-1.190*	.133	.000	-1.463	-.917	
	NS	.533*	.133	.000	.259	.806	
	Pep+YeEx	.253	.133	.068	-.020	.526	
	YeEx+Urea	.532*	.133	.000	.259	.805	
	Pep+NCl	-1.593*	.133	.000	-1.867	-1.320	
	Urea+NCl	-4.697*	.133	.000	-4.970	-4.423	
	YeEx+NCl	-2.279*	.133	.000	-2.552	-2.006	
	Pep+NS	-1.060*	.133	.000	-1.334	-.787	
	Urea+NS	-1.600*	.133	.000	-1.873	-1.327	
	YeEx+NS	-.793*	.133	.000	-1.066	-.519	
YeEx+Urea	Pep	-1.466*	.133	.000	-1.739	-1.192	
	Urea	-3.298*	.133	.000	-3.571	-3.024	
	YeEx	-2.791*	.133	.000	-3.064	-2.517	
	NCl	-1.722*	.133	.000	-1.995	-1.449	
	NS	.001	.133	.996	-.273	.274	
	Pep+YeEx	-.279*	.133	.046	-.552	-.006	
	Urea+Pep	-.532*	.133	.000	-.805	-.259	
	Pep+NCl	-2.125*	.133	.000	-2.399	-1.852	
	Urea+NCl	-5.229*	.133	.000	-5.502	-4.955	
	YeEx+NCl	-2.811*	.133	.000	-3.084	-2.538	
	Pep+NS	-1.592*	.133	.000	-1.866	-1.319	
	Urea+NS	-2.132*	.133	.000	-2.405	-1.859	
	YeEx+NS	-1.325*	.133	.000	-1.598	-1.051	
Pep+NCl	Pep	.660*	.133	.000	.386	.933	
	Urea	-1.172*	.133	.000	-1.446	-.899	
	YeEx	-.665*	.133	.000	-.939	-.392	
	NCl	.403*	.133	.005	.130	.677	
	NS	2.126*	.133	.000	1.853	2.399	
	Pep+YeEx	1.846*	.133	.000	1.573	2.120	
	Urea+Pep	1.593*	.133	.000	1.320	1.867	
	YeEx+Urea	2.125*	.133	.000	1.852	2.399	
	Urea+NCl	-3.103*	.133	.000	-3.377	-2.830	
	YeEx+NCl	-.686*	.133	.000	-.959	-.412	
	Pep+NS	.533*	.133	.000	.260	.806	
	Urea+NS	-.007	.133	.961	-.280	.267	
	YeEx+NS	.801*	.133	.000	.527	1.074	
Urea+NCl	Pep	3.763*	.133	.000	3.490	4.036	
	Urea	1.931*	.133	.000	1.658	2.204	
	YeEx	2.438*	.133	.000	2.165	2.711	
	NCl	3.507*	.133	.000	3.233	3.780	
	NS	5.229*	.133	.000	4.956	5.503	
	Pep+YeEx	4.950*	.133	.000	4.676	5.223	
	Urea+Pep	4.697*	.133	.000	4.423	4.970	
	YeEx+Urea	5.229*	.133	.000	4.955	5.502	
	Pep+NCl	3.103*	.133	.000	2.830	3.377	
	YeEx+NCl	2.418*	.133	.000	2.144	2.691	
	Pep+NS	3.636*	.133	.000	3.363	3.910	
	Urea+NS	3.097*	.133	.000	2.823	3.370	
	YeEx+NS	3.904*	.133	.000	3.631	4.177	
YeEx+NCl	Pep	1.345*	.133	.000	1.072	1.619	
	Urea	-.487*	.133	.001	-.760	-.213	
	YeEx	.020	.133	.880	-.253	.294	
	NCl	1.089*	.133	.000	.816	1.362	
	NS	2.812*	.133	.000	2.538	3.085	
	Pep+YeEx	2.532*	.133	.000	2.259	2.805	
	Urea+Pep	2.279*	.133	.000	2.006	2.552	
	YeEx+Urea	2.811*	.133	.000	2.538	3.084	
	Pep+NCl	.686*	.133	.000	.412	.959	
	Urea+NCl	-2.418*	.133	.000	-2.691	-2.144	
	Pep+NS	1.219*	.133	.000	.945	1.492	
	Urea+NS	.679*	.133	.000	.406	.952	
	YeEx+NS	1.486*	.133	.000	1.213	1.760	
Pep+NS	Pep	.127	.133	.351	-.147	.400	
	Urea	-1.705*	.133	.000	-1.979	-1.432	
	YeEx	-1.198*	.133	.000	-1.472	-.925	
	NCl	-.130	.133	.339	-.403	.144	
	NS	1.593*	.133	.000	1.320	1.866	
	Pep+YeEx	1.313*	.133	.000	1.040	1.587	
	Urea+Pep	1.060*	.133	.000	.787	1.334	
	YeEx+Urea	1.592*	.133	.000	1.319	1.866	
	Pep+NCl	-.533*	.133	.000	-.806	-.260	
	Urea+NCl	-3.636*	.133	.000	-3.910	-3.363	
	YeEx+NCl	-1.219*	.133	.000	-1.492	-.945	
	Urea+NS	-.540*	.133	.000	-.813	-.266	
	YeEx+NS	.268	.133	.055	-.006	.541	
Urea+NS	Pep	.666*	.133	.000	.393	.940	
	Urea	-1.166*	.133	.000	-1.439	-.892	
	YeEx	-.659*	.133	.000	-.932	-.385	
	NCl	.410*	.133	.005	.137	.683	
	NS	2.133*	.133	.000	1.859	2.406	
	Pep+YeEx	1.853*	.133	.000	1.580	2.126	
	Urea+Pep	1.600*	.133	.000	1.327	1.873	
	YeEx+Urea	2.132*	.133	.000	1.859	2.405	
	Pep+NCl	.007	.133	.961	-.267	.280	
	Urea+NCl	-3.097*	.133	.000	-3.370	-2.823	
	YeEx+NCl	-.679*	.133	.000	-.952	-.406	
	Pep+NS	.540*	.133	.000	.266	.813	
	YeEx+NS	.807*	.133	.000	.534	1.081	
YeEx+NS	Pep	-.141	.133	.300	-.414	.132	
	Urea	-1.973*	.133	.000	-2.246	-1.700	
	YeEx	-1.466*	.133	.000	-1.739	-1.193	
	NCl	-.397*	.133	.006	-.671	-.124	
	NS	1.325*	.133	.000	1.052	1.599	
	Pep+YeEx	1.046*	.133	.000	.772	1.319	
	Urea+Pep	.793*	.133	.000	.519	1.066	
	YeEx+Urea	1.325*	.133	.000	1.051	1.598	
	Pep+NCl	-.801*	.133	.000	-1.074	-.527	
	Urea+NCl	-3.904*	.133	.000	-4.177	-3.631	
	YeEx+NCl	-1.486*	.133	.000	-1.760	-1.213	
	Pep+NS	-.268	.133	.055	-.541	.006	
	Urea+NS	-.807*	.133	.000	-1.081	-.534	

Based on estimated marginal meansa	
*. The mean difference is significant at the .05 level.	
a. Species = RP	
c. Adjustment for multiple comparisons: Least Significant Difference (equivalent to no adjustments).	


Univariate Testsa	
Dependent Variable:   Bio  	
	Sum of Squares	df	Mean Square	F	Sig.	Partial Eta Squared	
Contrast	81.025	13	6.233	233.441	.000	.991	
Error	.748	28	.027				

The F tests the effect of NitroS. This test is based on the linearly independent pairwise comparisons among the estimated marginal means.a	
a. Species = RP	


4. Species * NitroSa	
Dependent Variable:   Bio  	
Species	NitroS	Mean	Std. Error	95% Confidence Interval	
				Lower Bound	Upper Bound	
RP	Pep	2.802	.094	2.609	2.995	
	Urea	4.634	.094	4.441	4.827	
	YeEx	4.127	.094	3.934	4.320	
	NCl	3.058	.094	2.865	3.252	
	NS	1.336	.094	1.142	1.529	
	Pep+YeEx	1.615	.094	1.422	1.809	
	Urea+Pep	1.868	.094	1.675	2.062	
	YeEx+Urea	1.336	.094	1.143	1.530	
	Pep+NCl	3.462	.094	3.268	3.655	
	Urea+NCl	6.565	.094	6.372	6.758	
	YeEx+NCl	4.147	.094	3.954	4.341	
	Pep+NS	2.929	.094	2.735	3.122	
	Urea+NS	3.468	.094	3.275	3.662	
	YeEx+NS	2.661	.094	2.468	2.854	

a. Species = RP	


Post Hoc Tests


NitroS


Multiple Comparisonsa	
Dependent Variable:   Bio  	
Tukey HSD  	
(I) NitroS	(J) NitroS	Mean Difference (I-J)	Std. Error	Sig.	95% Confidence Interval	
					Lower Bound	Upper Bound	
Pep	Urea	-1.83200*	.133414	.000	-2.32035	-1.34365	
	YeEx	-1.32500*	.133414	.000	-1.81335	-.83665	
	NCl	-.25633	.133414	.799	-.74468	.23202	
	NS	1.46633*	.133414	.000	.97798	1.95468	
	Pep+YeEx	1.18667*	.133414	.000	.69832	1.67502	
	Urea+Pep	.93367*	.133414	.000	.44532	1.42202	
	YeEx+Urea	1.46567*	.133414	.000	.97732	1.95402	
	Pep+NCl	-.65967*	.133414	.002	-1.14802	-.17132	
	Urea+NCl	-3.76300*	.133414	.000	-4.25135	-3.27465	
	YeEx+NCl	-1.34533*	.133414	.000	-1.83368	-.85698	
	Pep+NS	-.12667	.133414	.999	-.61502	.36168	
	Urea+NS	-.66633*	.133414	.002	-1.15468	-.17798	
	YeEx+NS	.14100	.133414	.998	-.34735	.62935	
Urea	Pep	1.83200*	.133414	.000	1.34365	2.32035	
	YeEx	.50700*	.133414	.036	.01865	.99535	
	NCl	1.57567*	.133414	.000	1.08732	2.06402	
	NS	3.29833*	.133414	.000	2.80998	3.78668	
	Pep+YeEx	3.01867*	.133414	.000	2.53032	3.50702	
	Urea+Pep	2.76567*	.133414	.000	2.27732	3.25402	
	YeEx+Urea	3.29767*	.133414	.000	2.80932	3.78602	
	Pep+NCl	1.17233*	.133414	.000	.68398	1.66068	
	Urea+NCl	-1.93100*	.133414	.000	-2.41935	-1.44265	
	YeEx+NCl	.48667	.133414	.051	-.00168	.97502	
	Pep+NS	1.70533*	.133414	.000	1.21698	2.19368	
	Urea+NS	1.16567*	.133414	.000	.67732	1.65402	
	YeEx+NS	1.97300*	.133414	.000	1.48465	2.46135	
YeEx	Pep	1.32500*	.133414	.000	.83665	1.81335	
	Urea	-.50700*	.133414	.036	-.99535	-.01865	
	NCl	1.06867*	.133414	.000	.58032	1.55702	
	NS	2.79133*	.133414	.000	2.30298	3.27968	
	Pep+YeEx	2.51167*	.133414	.000	2.02332	3.00002	
	Urea+Pep	2.25867*	.133414	.000	1.77032	2.74702	
	YeEx+Urea	2.79067*	.133414	.000	2.30232	3.27902	
	Pep+NCl	.66533*	.133414	.002	.17698	1.15368	
	Urea+NCl	-2.43800*	.133414	.000	-2.92635	-1.94965	
	YeEx+NCl	-.02033	.133414	1.000	-.50868	.46802	
	Pep+NS	1.19833*	.133414	.000	.70998	1.68668	
	Urea+NS	.65867*	.133414	.002	.17032	1.14702	
	YeEx+NS	1.46600*	.133414	.000	.97765	1.95435	
NCl	Pep	.25633	.133414	.799	-.23202	.74468	
	Urea	-1.57567*	.133414	.000	-2.06402	-1.08732	
	YeEx	-1.06867*	.133414	.000	-1.55702	-.58032	
	NS	1.72267*	.133414	.000	1.23432	2.21102	
	Pep+YeEx	1.44300*	.133414	.000	.95465	1.93135	
	Urea+Pep	1.19000*	.133414	.000	.70165	1.67835	
	YeEx+Urea	1.72200*	.133414	.000	1.23365	2.21035	
	Pep+NCl	-.40333	.133414	.188	-.89168	.08502	
	Urea+NCl	-3.50667*	.133414	.000	-3.99502	-3.01832	
	YeEx+NCl	-1.08900*	.133414	.000	-1.57735	-.60065	
	Pep+NS	.12967	.133414	.999	-.35868	.61802	
	Urea+NS	-.41000	.133414	.171	-.89835	.07835	
	YeEx+NS	.39733	.133414	.205	-.09102	.88568	
NS	Pep	-1.46633*	.133414	.000	-1.95468	-.97798	
	Urea	-3.29833*	.133414	.000	-3.78668	-2.80998	
	YeEx	-2.79133*	.133414	.000	-3.27968	-2.30298	
	NCl	-1.72267*	.133414	.000	-2.21102	-1.23432	
	Pep+YeEx	-.27967	.133414	.698	-.76802	.20868	
	Urea+Pep	-.53267*	.133414	.023	-1.02102	-.04432	
	YeEx+Urea	-.00067	.133414	1.000	-.48902	.48768	
	Pep+NCl	-2.12600*	.133414	.000	-2.61435	-1.63765	
	Urea+NCl	-5.22933*	.133414	.000	-5.71768	-4.74098	
	YeEx+NCl	-2.81167*	.133414	.000	-3.30002	-2.32332	
	Pep+NS	-1.59300*	.133414	.000	-2.08135	-1.10465	
	Urea+NS	-2.13267*	.133414	.000	-2.62102	-1.64432	
	YeEx+NS	-1.32533*	.133414	.000	-1.81368	-.83698	
Pep+YeEx	Pep	-1.18667*	.133414	.000	-1.67502	-.69832	
	Urea	-3.01867*	.133414	.000	-3.50702	-2.53032	
	YeEx	-2.51167*	.133414	.000	-3.00002	-2.02332	
	NCl	-1.44300*	.133414	.000	-1.93135	-.95465	
	NS	.27967	.133414	.698	-.20868	.76802	
	Urea+Pep	-.25300	.133414	.812	-.74135	.23535	
	YeEx+Urea	.27900	.133414	.701	-.20935	.76735	
	Pep+NCl	-1.84633*	.133414	.000	-2.33468	-1.35798	
	Urea+NCl	-4.94967*	.133414	.000	-5.43802	-4.46132	
	YeEx+NCl	-2.53200*	.133414	.000	-3.02035	-2.04365	
	Pep+NS	-1.31333*	.133414	.000	-1.80168	-.82498	
	Urea+NS	-1.85300*	.133414	.000	-2.34135	-1.36465	
	YeEx+NS	-1.04567*	.133414	.000	-1.53402	-.55732	
Urea+Pep	Pep	-.93367*	.133414	.000	-1.42202	-.44532	
	Urea	-2.76567*	.133414	.000	-3.25402	-2.27732	
	YeEx	-2.25867*	.133414	.000	-2.74702	-1.77032	
	NCl	-1.19000*	.133414	.000	-1.67835	-.70165	
	NS	.53267*	.133414	.023	.04432	1.02102	
	Pep+YeEx	.25300	.133414	.812	-.23535	.74135	
	YeEx+Urea	.53200*	.133414	.023	.04365	1.02035	
	Pep+NCl	-1.59333*	.133414	.000	-2.08168	-1.10498	
	Urea+NCl	-4.69667*	.133414	.000	-5.18502	-4.20832	
	YeEx+NCl	-2.27900*	.133414	.000	-2.76735	-1.79065	
	Pep+NS	-1.06033*	.133414	.000	-1.54868	-.57198	
	Urea+NS	-1.60000*	.133414	.000	-2.08835	-1.11165	
	YeEx+NS	-.79267*	.133414	.000	-1.28102	-.30432	
YeEx+Urea	Pep	-1.46567*	.133414	.000	-1.95402	-.97732	
	Urea	-3.29767*	.133414	.000	-3.78602	-2.80932	
	YeEx	-2.79067*	.133414	.000	-3.27902	-2.30232	
	NCl	-1.72200*	.133414	.000	-2.21035	-1.23365	
	NS	.00067	.133414	1.000	-.48768	.48902	
	Pep+YeEx	-.27900	.133414	.701	-.76735	.20935	
	Urea+Pep	-.53200*	.133414	.023	-1.02035	-.04365	
	Pep+NCl	-2.12533*	.133414	.000	-2.61368	-1.63698	
	Urea+NCl	-5.22867*	.133414	.000	-5.71702	-4.74032	
	YeEx+NCl	-2.81100*	.133414	.000	-3.29935	-2.32265	
	Pep+NS	-1.59233*	.133414	.000	-2.08068	-1.10398	
	Urea+NS	-2.13200*	.133414	.000	-2.62035	-1.64365	
	YeEx+NS	-1.32467*	.133414	.000	-1.81302	-.83632	
Pep+NCl	Pep	.65967*	.133414	.002	.17132	1.14802	
	Urea	-1.17233*	.133414	.000	-1.66068	-.68398	
	YeEx	-.66533*	.133414	.002	-1.15368	-.17698	
	NCl	.40333	.133414	.188	-.08502	.89168	
	NS	2.12600*	.133414	.000	1.63765	2.61435	
	Pep+YeEx	1.84633*	.133414	.000	1.35798	2.33468	
	Urea+Pep	1.59333*	.133414	.000	1.10498	2.08168	
	YeEx+Urea	2.12533*	.133414	.000	1.63698	2.61368	
	Urea+NCl	-3.10333*	.133414	.000	-3.59168	-2.61498	
	YeEx+NCl	-.68567*	.133414	.001	-1.17402	-.19732	
	Pep+NS	.53300*	.133414	.023	.04465	1.02135	
	Urea+NS	-.00667	.133414	1.000	-.49502	.48168	
	YeEx+NS	.80067*	.133414	.000	.31232	1.28902	
Urea+NCl	Pep	3.76300*	.133414	.000	3.27465	4.25135	
	Urea	1.93100*	.133414	.000	1.44265	2.41935	
	YeEx	2.43800*	.133414	.000	1.94965	2.92635	
	NCl	3.50667*	.133414	.000	3.01832	3.99502	
	NS	5.22933*	.133414	.000	4.74098	5.71768	
	Pep+YeEx	4.94967*	.133414	.000	4.46132	5.43802	
	Urea+Pep	4.69667*	.133414	.000	4.20832	5.18502	
	YeEx+Urea	5.22867*	.133414	.000	4.74032	5.71702	
	Pep+NCl	3.10333*	.133414	.000	2.61498	3.59168	
	YeEx+NCl	2.41767*	.133414	.000	1.92932	2.90602	
	Pep+NS	3.63633*	.133414	.000	3.14798	4.12468	
	Urea+NS	3.09667*	.133414	.000	2.60832	3.58502	
	YeEx+NS	3.90400*	.133414	.000	3.41565	4.39235	
YeEx+NCl	Pep	1.34533*	.133414	.000	.85698	1.83368	
	Urea	-.48667	.133414	.051	-.97502	.00168	
	YeEx	.02033	.133414	1.000	-.46802	.50868	
	NCl	1.08900*	.133414	.000	.60065	1.57735	
	NS	2.81167*	.133414	.000	2.32332	3.30002	
	Pep+YeEx	2.53200*	.133414	.000	2.04365	3.02035	
	Urea+Pep	2.27900*	.133414	.000	1.79065	2.76735	
	YeEx+Urea	2.81100*	.133414	.000	2.32265	3.29935	
	Pep+NCl	.68567*	.133414	.001	.19732	1.17402	
	Urea+NCl	-2.41767*	.133414	.000	-2.90602	-1.92932	
	Pep+NS	1.21867*	.133414	.000	.73032	1.70702	
	Urea+NS	.67900*	.133414	.001	.19065	1.16735	
	YeEx+NS	1.48633*	.133414	.000	.99798	1.97468	
Pep+NS	Pep	.12667	.133414	.999	-.36168	.61502	
	Urea	-1.70533*	.133414	.000	-2.19368	-1.21698	
	YeEx	-1.19833*	.133414	.000	-1.68668	-.70998	
	NCl	-.12967	.133414	.999	-.61802	.35868	
	NS	1.59300*	.133414	.000	1.10465	2.08135	
	Pep+YeEx	1.31333*	.133414	.000	.82498	1.80168	
	Urea+Pep	1.06033*	.133414	.000	.57198	1.54868	
	YeEx+Urea	1.59233*	.133414	.000	1.10398	2.08068	
	Pep+NCl	-.53300*	.133414	.023	-1.02135	-.04465	
	Urea+NCl	-3.63633*	.133414	.000	-4.12468	-3.14798	
	YeEx+NCl	-1.21867*	.133414	.000	-1.70702	-.73032	
	Urea+NS	-.53967*	.133414	.020	-1.02802	-.05132	
	YeEx+NS	.26767	.133414	.752	-.22068	.75602	
Urea+NS	Pep	.66633*	.133414	.002	.17798	1.15468	
	Urea	-1.16567*	.133414	.000	-1.65402	-.67732	
	YeEx	-.65867*	.133414	.002	-1.14702	-.17032	
	NCl	.41000	.133414	.171	-.07835	.89835	
	NS	2.13267*	.133414	.000	1.64432	2.62102	
	Pep+YeEx	1.85300*	.133414	.000	1.36465	2.34135	
	Urea+Pep	1.60000*	.133414	.000	1.11165	2.08835	
	YeEx+Urea	2.13200*	.133414	.000	1.64365	2.62035	
	Pep+NCl	.00667	.133414	1.000	-.48168	.49502	
	Urea+NCl	-3.09667*	.133414	.000	-3.58502	-2.60832	
	YeEx+NCl	-.67900*	.133414	.001	-1.16735	-.19065	
	Pep+NS	.53967*	.133414	.020	.05132	1.02802	
	YeEx+NS	.80733*	.133414	.000	.31898	1.29568	
YeEx+NS	Pep	-.14100	.133414	.998	-.62935	.34735	
	Urea	-1.97300*	.133414	.000	-2.46135	-1.48465	
	YeEx	-1.46600*	.133414	.000	-1.95435	-.97765	
	NCl	-.39733	.133414	.205	-.88568	.09102	
	NS	1.32533*	.133414	.000	.83698	1.81368	
	Pep+YeEx	1.04567*	.133414	.000	.55732	1.53402	
	Urea+Pep	.79267*	.133414	.000	.30432	1.28102	
	YeEx+Urea	1.32467*	.133414	.000	.83632	1.81302	
	Pep+NCl	-.80067*	.133414	.000	-1.28902	-.31232	
	Urea+NCl	-3.90400*	.133414	.000	-4.39235	-3.41565	
	YeEx+NCl	-1.48633*	.133414	.000	-1.97468	-.99798	
	Pep+NS	-.26767	.133414	.752	-.75602	.22068	
	Urea+NS	-.80733*	.133414	.000	-1.29568	-.31898	

Based on observed means.
 The error term is Mean Square(Error) = .027.a	
*. The mean difference is significant at the .05 level.	
a. Species = RP	


Homogeneous Subsets


Bioa	
Tukey HSDb,c  	
NitroS	N	Subset	
		1	2	3	4	5	6	7	
NS	3	1.33567							
YeEx+Urea	3	1.33633							
Pep+YeEx	3	1.61533	1.61533						
Urea+Pep	3		1.86833						
YeEx+NS	3			2.66100					
Pep	3			2.80200					
Pep+NS	3			2.92867					
NCl	3			3.05833	3.05833				
Pep+NCl	3				3.46167				
Urea+NS	3				3.46833				
YeEx	3					4.12700			
YeEx+NCl	3					4.14733	4.14733		
Urea	3						4.63400		
Urea+NCl	3							6.56500	
Sig.		.698	.812	.205	.171	1.000	.051	1.000	

Means for groups in homogeneous subsets are displayed.
 Based on observed means.
 The error term is Mean Square(Error) = .027.a	
a. Species = RP	
b. Uses Harmonic Mean Sample Size = 3.000.	
c. Alpha = .05.	


Profile Plots


ûöUUUMMMi5L2¿~Çã)2'åGùp7Êo~~>'''++K	Á`Ð¶ÛIÉY,ýl²Xí­dG¦Æýû÷wwwAÊoÁÚQÛí§µ<ÛvîÜÙ××çÊòå'®®.9r$Fù"ÞíUg¿þõ¯KÃ©mµµµ2eïÞ½¾e2³2QÍïõzôø=zT¿Q_.ÐØØ¨RUU%e Ó«««ãBù¶Bù	yáñÀÀÀZËÏãñhS²²²ôSfffälff¦þF;;;+++F£ººÙlîïïç± ü7==]VV6Ñb±¨­Ê@ 777Î)aå÷ï|§ä×ë×Áüü|¿ß¿¦òÓ¿i«z.ì&dbäM/--]ºtI^¯e¢¢"¢òKü%ár¹Â&êvjÏ°òê©§>à¹£å'.^¼(gï¿ÿþ5_ØvÈm~2Qéôô´6¿TãJiÊ/.6-rº~·?ÉçÞílwuÄFÔÚS-.zuuðÞ½ç©C:êêêÔ¥G³÷Ýw¼XËÙãÇË;wòXP~	zðÁÏ=9=+++¨wreçÊ°¥Êovv6==¥òËÉÉQgkjj¢^jjJ»ºvlïµk×Ô¥333jGÀ0<_ä/áááÈéòmmmü¬ªªsåØRå'ZZZV*¿®®.õ!êÓû¢^|||ß¾¦eòbª?þ#´¼/þ¡C233Õ±v»ýNì¿-T~ò<û| õ¤ÈÎÎ6òså°yËïÎ¡üåGùP~åGùP~åGùP~p7ø¿÷¹7ïæé·¯ÀjÊòîß½ÿ'û;îæéÛ¼ÄjÊò4so²ò£üÊ@ùQ~å ü(?òP~@ùXãúÙl®®®öz½¬P~1mQÊ¯åg¯nÜdm³ü´q0ìêêª©©aµò°67oÞúö#/ýÅßÿJÊï¯ÿñßöýÝ/_þÕlæòSL&«µùáO_ýzö3ÿz·wà%T eÍ¹ôÊbçð'ë?Érb_0LKKSãùùùòòr£Ñh·Ûe¬Íït:Ífó®]»x_ò£ü|aï®÷¼tûùý¿gq½Í6mùIö555=zT­««¤ÃáÐæ?|ø°ÌÙÖÖ&3ð¸P~Ðâõé¸¬ÆR~?yâ5<ö±vÿl®uÌÌL¹ÅÅ/ÒÐb±¨ßïÏÎÎÖæA ÈÈÈ`R~ÛþÿzÉûáõnß7¾÷<ÛüMX~ñD¡Á`ßh4²)?ÊÀmQ÷ó[¼~5$KùEÝ¤'ó/,,·ùeee±)?ÊÀmêØÞ÷Kul¯dÇöÉU~uuuG¥¥¥ÚüÇA[[[=+ò£üüú<¿æ¯pT/tåçóùìv»Ñh,,,Ðæoii1Ljã(?ÊÀðÀ)EP~Ê ü@ùQ~å 	ñåòÊò@ùåGùIgäwïKÃÝÍÓ·yÕåÜ~äïîÍ»yúíë°Úò£üØ õcu9zå ü@ùQ~P~ ü(?6ç¿WíúO²8ËOÍÍÍùùù2///7v»]Æj·Û]\,³²²ÚÛÛy(?ÊñÙ¿½þìÕõd9ñ_GGßïq]]Ýìì¬N§ÃáP3X­Öþþ~tuueddðQ~ûE=¢üvQ0TcÅ¢ÙÙÙñ,åÀæb6õg%õ´)úÛ¦£¥¡Ïç;uêTMMÕj¥ü(?ÊÍ®¼¼|bbB;;>>¾÷îÈòúf®Ífkhhèîî¡ü(?ÊÍÎårIêIºÉxzzzÏ=åWWWçñxdÐÑÑQZZª&ÍæÉÉÉ@ àp8(?Êò 	tvvnß¾Ýh4ÊÏÖÖÖ?¼ÞëbÎçóÙív§°°PÛFØÝÝmµZ322(?Êò ü(?Êò ü(?Êò ü(?T+?¿ßèÐ!ÉwåÊýEn·;''Çh4îÜ¹S@Q<S(?MZ~>øà©S§Á d_~~¾þ¢ªªª.È ±±±ºº:Î)aå×ÝÝíà¹£¯¡öìq»Ýú-&2%VDCQ~wÕÎ;ÇÆÆ¢^d±XÔ·MÜÜÜ8§ásÜ#wô5ttt´¬¬L;+ãØåG	Q~÷Ñh<ö¬ÙlÎÏÏ¿zõjØEaãx¦ðn/`ëØ·oÚÙ©¿¿_Æ«DåGùÝs¡¥¥%´ü=ÓÅÅÅaicÉçÊ°Ù<ü¿ZúÖå-Yõ,))Ñ¾MÌÏÏF»Ý.ãUËOfs¹ #÷ÂV/¿½÷¦¥¥¿ûÙl6Ë«ªª*±Eeeeiã°vrQ -¿«fgåØR<X[[àÀýÄºººÙÙY8NÃ¡ßJûùA)HuEP~_Ø¿¿<Q<?ôOyæ%°4yj^¼xQÎé/ª©©Q_>-?UYÆ3òl)ÓÓÓò*<55¥h±XÔÀï÷gggkåc9Òååå¬RÊï¤§§ËSÇårËÀjµz<Èô6??_YYi4µÍÔê©900 OVIÌ¡¡¡8§P~-÷êtúM3ÚQ±ËoxxX^¥Y_'Shù"¨mÈú'ÖæAù¶fùeddÄ3¼ Ë+ûéÓ§YßIKK§Îüü¼Úø7;;åÊ·ùQ~lxùÕÕÕy<ÐòùÚÎT1ÊorrÒf³ÂÂBöó£üþÈÞ½µÈj×õìçGù°áåçóùìv»ÑhÓïLµÒj§©¾¾¾ÊÊJV)å÷GJJJd:P±?4òHÖòK ü(?Ê/>6Íh4í(À±½©V~êÃÁ§D~m.åÜå§¶öµ¶¶.--mòßòßºL¦ØIùp7^Ô·ms:aS´ñää¤ÝnWíÆÆÆ¨óò[]^^<i(?îmùè¿oM«º¹¹¹üüüÎÎÎ`0èñxJKK)?Ê/ê[zËËË7üQ~Ô.¿Èòs8gÎÑ¦Q~_Ï³¨8¶¨nN½|srxNS/½"?pCû¾5­êfffVzç¡üÖÀ°í ªÏ¾_ûÙÙ¿ÞÓ÷k#npp°¦¦&¬êb¼(S~_Ê¢ü©ü¢þeÃIùIÿQ~¸å7??_[[Kùp÷ËÏãñØl6ýÂÂÂééimÎ`0xéÒ%ÊòKPnn.ßáÀ&)?ÑÐÐÐÒÒ¢M©¯¯×áÑÛÛ[RRBùQ~?#"ï°X,÷¤üü~qq±6eff&''çòåË¡åóòòººº(?Ê/ê½^ovv¶$øN§9BùpOÊO¨cí¬Ûí.))1¹¹¹çÏ_éZ üV©'¤¦§§Á ÒÒÒ(?*¿é¼	ÜÿýjÀ§º¤ZùÕ××kÇsèwõ+--¥üRªüBËeffÊ`hhHRÅÅÅð·¥üå·UP~ò£ü(¿øîØ±#--MííÕÑÑAù¤Zùõôôo766R~Ü½õmÛNgØ5X\t8Åh4æçç<yÕEù%Hóèè¨V~CCC2NOO§ü¸åWRRâ÷û#Ëoÿþý---KKK2'±Æ(¿gê¥ú$g>Ï» wåö¢¼¸¸¨>ßeeeÉKmçò¿$?.ãÜÜH¾3|N¾3aå'?m6ÛììlXùY­ÖS§N±æ±åwåÊmÑR~DúÄwnCÊOY~òú[SSV~W¯^ÍÏÏÏÍÍu8ÝÝÝÃÃÃ<_âæææÕ±½f³yÇSSSð·¥ü©ü¢þeçIù©í/ÚÅív;w®¢¢Âh4^¼x5Fù¥8Ê°ÊÏãñØl¶ÈòÓmÂ²@ùQ~¬¹üBË_¬ÚÒÒ¢MIOOúÓÒÒXcßÚ^±©ã|)?î~ùùýþââbmJmmíÙ³g=º¨±±ñØ±c¬1Ê/ò3¬Ouà^p:Ú¯×»wï^µG¾Åb9qâD `Q~_~~þ|>ßæÿm)?@ù%Bþz8wîÜöíÛµÌÌÌÜ·oßæ<ªòØ#<ü~¿Ëå²Z­A«ÀÊ ¥ÊO¯··733#<R³üü~¿ÕjÕØït:)?T(¿Èýü,KMMûù¤ZùíÒÇ±½©_~|3âE]÷l6«««½^ïF-|Ï=n·[;+c²ÖNà1Jâò3¬&±Or[é)"O²YìÎ;ûúúâBù¶Nùiã`0ØÕÕUSS³Q-++ÓÎÊxdd$Î;T(¿;¤££ãÐ¡CQ/ªªªºpáåï8§P~-X~ÉdÚÀ¥íÛ·OmUéïïñZ¯Ê/É>ù%êEEÛt ÈÍÍsJXù=úè£#Ü#êõh»o×O­ÿ$Ë[ò¦ÆóóóåååF£Ñn·ËXßétÍæ]»vE¾/nãããÅÅÅ2())Ð¦¯´ð^ëe6Ë¥2r3¶VùìÞ½[=òÜgþ"ýÛÇjÏ°ò§øÜ#êõèÅ¼ÏúÞ[ÿIãÝÞ¦¦¦£Gª³uuu³³³¡å/óu8Úü9ÛÚÚdx6Ú<x°¶¶öÀú+-|¥ýüFGGKKKåv¥ Õ±uËO#OÂÂBýý!#jóu<Sx·°uÞíÕ¥ªÙââ¢ö¾øýþììlmþ¹¹9õFYFFÆªÅ&¦§§ålØ'¸­´ðwUÚ±¼¼¼¡¡Gò­nYYYòÔTOPÇ9òlò'µ­$úù#ß([iiÓW]x¤ááa¹E)Eµ­^~ê/ÙÙÙÝ»wë/ª©©immü¬ªªså ü´Mzaó/,,VØù­´ðwÕáp>G-iÊïËå²Z­òwÀW¾òí½õìÈÎÎÅæääÅ9òP~uuu'´ü¥¥¥ÚüÇA[[[Âå·ÒÂWº36Mr³°°ýü¦üîÐçùÝQ`kÏç³ÛíòÒ,±¥+ó·´´L¦ÊÊJµñ/±[Yiá+í/XQQ¡¶ÎôõõÉMóÀ%Gù%#ÊxJßÚÌÏÏ×ÖÖR~P~HµòËÍÍ5|o/,oÍj¸§½Äj6õ|Ã¶nùF¾o±X´Ï¢ü)¿m%r¢ü`ëüÅ ©çõz³³³e Áçt:epäÈÊH1?ö+ÖlÝòÓÖÔÁôôt0ö]@ùR¤ü222¤ó&&&dpÿý÷«êP~T+¿úúzíxý®~Úg9R~åàn¼¨Gû^µ;tCÚÑf³¹ººÚëõ²þ·JùÌÌLÉ@ÅÅÅð·¥üÊ ü6öÁ`WWWMMë_² üÝò>zôß;õþIV°¥Ê/l9åb(?ÊH7Þzwú®zçÿïR~³þoü»]|J°./¾òlÀI_ùÉ ¹¹9???´ümåååF£Ñn·ËXÍàv»ebVVVûÊ/juF]¸Ìït:Ífó®]»x_8ÊÏf³ñIÎ@óì©ÿþ%ôå»½)ÄüdÍëE=fùuttøý~×ÕÕÍÎÎÊ@jÌáp¨¬Vk¿ººº222â/?É¾¦¦¦£Gª³Q.ó>|Xælkkx¤»üÊÊÊÂOáØ^ 5ÜòÞ°|5¸x]ß|s§ÞûÛægå¼ü´­0r[,5ÌÎÎ±mÑMÏÌÌÓ¾¾!êÂe¶¹¹9È¬DÚÚ×ÚÚº´´´É[ÊH4ßïó*ÔXÊÏ÷6xøáç~ÊÊ6³ÙüGÿxAm>£¾AçóùN:USScµZWÝ_0ÆîQ®n¢üÖFGRü¶éÿµO¯¼úòÝÞ[K7µ)6òòò	íìøøøîÝ»#Û+êV7ÍÖÐÐÐÝÝ=33³òºpaa!´¼Í/++G*¹Ë///OD)? %-^|ÃòÕO~þëÛïö~ïâì_üãì×Kÿ±fMÅårIêIºÝþmzzÏ=­VWWçñÜÞO·££Cûð]³Ù<99)eæp8ÖS~Q.ó?~mmmõõõ<RÉ]~òË#*glþø£ü]öêLé!)¿72ÿôÚßç?À:6¡ÎÎÎíÛ·FùÙÚÚµÕ|>ÝnyµmÝÝÝV«5##£©©iÕ·òbÌuá2KKÉdª¬¬LMEß*TÛ¤>É@b©ÀJHò3¬cÊ(¿T+¿$Bù»oø ü(?ò$Oùiò±@ùR¼ü´=ùØÏH:7?úäú³W8IùÍjHà·XíÄå(?@Qw7OÒ¬vàNÛ³gÛíÖÎÊX¦Ä¾mÍ1±Úçÿò£üØ`£££eeeÚY¬©Ø	ÊoKßJûùÆ¬¬¬Mô>åHmûöíSßÛÑßß/ãµ[b©Gùm­òSõEÞ¡åÆ3°ôÖÓ7Öå-y||¼¸¸X%%%úïð///7v»]Æ«öÌær¹TAVWW' 2hnnÎÏÏ_é>¸Ýn¹ÃjkQ;Ï$(¿¥¥¥´´´õ,òsÏ=;wî-<äò@sõ_>ÿÍ÷6à$ËÁkkk8 XWW7;;+§Ó©m±ßèèhiii0TWL¸ü:::ü~ÿJ÷ÁjµJ ««+##çFß;äqþð%×¯_)»wïêm_ÊØ|·N>yWOÝ¯²Ö»czzZ^|§¦¦ôµ­0aÙÙÙ+´cyy¹~¯­J1r9Ú'»ÉE1îCìÄf,?õÀ/-ýa³|ò`S~ÀfÜÞ0õâ_ÿsy'i¸®o=ÀÏýâ°Ú»÷êm#îÆ.­ááayWëb/ßl6ëÏÊ«¿6E?gÔûàóùN:USScµZ)¿ä(?Évy¨***å¬ü±z×årñn/°	ðéOv%pòôü?%pÅß¹ÊjîaùE#5vi9²²²Ó§O¯zÅòòrý>ããã»wï3ê°ÙlÝÝÝ333_r_OOOÔÍ¿jpäÈÊHR~W/ÿõ$]ùÕÕÕy<ttt®Z~Òd@ °°pÕýü¤¤[hù½æ=ö¨ãÃæzÌf³ÜÜ&åå'äÜ±c<xA~ÊX=´òÊßç·¥üÊØåçóùìv»ÑhÓ¶ÏÅ8Â£¢¢bhhHÒp«ÞbggçöíÛeùò³µµ5ê=zº»»­VkFFFSSå4å,(?òÊo]rssóóóÇÆÆ(?ò¤xùÆdÙ<KùñÝzÌu»ü¾óÃ[ã¡¥ «¶bù©#9xàùùyí3(? ¥¼öîïþ[ã#'¤üþîÑ×à¼Ùîb­ÀV,¿¾·Wû¨ÊHvþ×ôM<%å7ôÄÏþÛÏ¾º3ô/k¶V°y>ÀòÖ×7¼ÿÜázëÚÏï¿¸¼ùÓéyu[®üå$àºÿSß©ÇC%U~Ïÿâ¢F[®':X9@ùÝ6??_[[Kù)àó¥ÏèÁ§ÇUù¹ÙúðÓÏêî~î§¬`s½¨ëv¸2ÍÕÕÕ^¯wît:Ã¦hãÉÉI»Ýn2rrr£ÎÔ)¿ÜÜ/ûù)é¿ýäÈGgyëgÃ·ßímmé;ùÝ#oxß`Í­ü´q0ìêêª©©ÙÀè¿ÆW»¹¹¹¹üüüÎÎN¹QÇSZZÚÜÜLù¥lùFÞa±XÔ×øR~@öMõ_Êþí_(å×ÿýïk:ð#W«ØÌå§L¦ZmiiihhÁáp9sF>66VTTDù¥lùÉ³JW¯×-	>§Ó¹©¾®òÖoþ³ùï=û°ßùæûû&bëÑ6õGßøÖúO²­ÓÒÒ¾ø'<?_^^n4ív»µùå%Ûl6ïÚµ+òáÈò6Mû_mõ¥½ñÄ(¾ü´¯ùÔÁôô´<Õd =ÛÐÓÓù»Ý999ò¬Ý¹s§ú*èx¦P~Àâ;<ñÂû½Ï_rý'YÎJ%¯ÅMMMGUgëêêT±Iê9mþÃËmmm2C<å788¨½¬ÍãÓ<(¿,¿y&&&dpÿý÷«AÂê²´´TTTùªªºpá«««ãBù°U^Ôu233%æ´=¯,øýþììlmþ¹¹9y5FßpR~ÒßÖ-¿úúzíxý³¤´´4±<yòôéÓÏyÖªï'hnnnSÂÊï¡º QR~~ØÄzv§Ë/(ÔÁÔÏYoQ·ù	Çc³ÙôS§§§õ/]ºDù¥lùùÛBCCC2§Tqqqbëª÷Ã.Ò?)Õ8)aåwåÊ%å÷î'X@ÂîUùiôÂæ_XXPK²²²â,?õºßÒÒ¢M©¯¯×áÑÛÛ[RRBù¥rùm ÊÊÊþþþ¨ÏýÇÄ¨âÂ»½@,Þê~5ÓÉ'oÖ~ÅÐ+Ö:pÞí]é¢ºº:çö?ÃíM9ÿøñã2hkkz¿üü~qq±6eff&''GmÑËËËëêê¢ü(¿xµa;häÏù£Dÿ§I<S(? gêwWÀ©ë[Ê)ëv?Ájîaùù|>»Ýn4'&&´ù[ZZL&See¥ÚøÿÂÕçxhgÝnwII,?77÷üùóñÜ%$Yùm[Í:?É9ò¹RSSÓÚÚ*ùYUUçÊ4qíwñÀÝ<ñù@r"(¿ËÏ°í|:ªñÀÀ@vv¶,9''ghh(Î)6¬üòóó/àóù6ÿoKù YÏ7|`Ë×ë=wîÜöíÛõ´oß¾©©)Ê ¥ÊOã÷û].Õj5ZtttP~)U~z½½½rå°éÊÏï÷÷÷÷[­Vý½ùùùN§òHòÜÏÏb±ÔÔÔ°÷æE=âXÝ;tô®,6lûþ&''ív»ÉdÊÉÉill¼Óww©üÂvéãØ^¶NùøýþÈËÏÏïìì§´´´¹¹òK©ò»CäLù°IÊ/òÜZZZ"gp8úïð+**¢üR¡ü«Yç'9S~TõWÿðoé¸¼þ,'ÎòAsss~~¾çççËËËåeÚn·ËXÍàv»ebVVVûªå'?m6ÛììlØ333q)©üåØ¼^ÏûéúO²øË¯££C½?[WW§Íét:5ÕjíïïAWWWFFF<å788XSS6Cí>åGù°q/êi¥íy%A5¶X,j !c9Q÷æÒÏ å'ýGùQ~°©Ýô/~òöÈÝ<ùo³Ú;Íl6ëÏJêiSôÕu§|ÏwêÔ))9õ1m±£M;ëñxl6~Jaaáôô´þ>tò£ü(?à^zâ«ýÉÝ<õwdµwZyyùÄÄvv|||÷îÝÕùfnhy§½îîîøËOÈµZZZ´)õõõú#<zKJJ(?Êòî¥¾ç¯=ÀIî'=û¸âäÂ«¬vàNs¹zêééé=öôõõEVW]]ÇãAGGGii©h6'''ÃáXSùùýþââbmÜzNNÎåËCËöæååuuuQ~å$%)¿?Íz6­ÎÎÎíÛ·FùÙÚÚµÕ|>ÝnyµmÝÝÝV«5##£©©iÕDÁétê§¸ÝîY~nnîùóçWº¬üø<?òlòÓt_ÔòãóüÊ"å§éééÎÛ¿ÿÂÂ2E,åP~Ô)?Å"·´´¤M	2%êGQ~@ò>òzæ¬Øºå§ÞÛÚ+?öóRÉdÿ¿¼ôðW¥ü.=²ë¥Gª®ÈçóÀ,¿¬¬,é¼ªªªë×oÌâââÞ½eL§üÔðjoÃÓMúÔ-R~O=ó»ö½xî?±f`Ë_Ô#<®^½Jù©ÁufkÊúr??ÿÍÏzÎuô7?bÍÀ+¿ÐòÇEÍfÁVTTtíÚµMøÛR~@®úÁÀ÷þôæ­ªüé­Á_|ê+ØTöìÙãv»µ³2)±"`¯å]sLlÛæt:Ã¦hãÉÉI»Ýn2rrr£Î$+¿dAù	¸¼pf×gï©òþ¹S2øIÍÀ±rMett´¬¬L;+ãØÅ¶11±m[IIßïÜÜ ~~ggg0ôx<¥¥¥ÍÍÍåGùÚ/ÿóÛ¯íñùÓï¾Ö3xöO<óc¬ A>úlNþ°ïÛ·Oc[¿W-¶S/ìlKKKCCCäCÿ5¾cccEEE_Òü±cÇ´´4u<oVVVGGå¤w?îhûªûÿYÊïò?þìö½zÕ$ìÖnÿ8ý üEm||¼¸¸X%%%Ú7³ùùùòòr£Ñh·Ûe¼jÉl.Kduuõªå'?m6ÛììlØê7°;qËO³þÛÔXÿ^>å$»On|tiì»R~?ýù×çzY!À¦uðàÁÚÚÚè'ÖÕÕ©,s:Ck¯öó---RZÏÅ.¿ÁÁÁ°b|¡å¬å-<E´òqzz:å¤¾ÃØü¦§§åUxjjJ?Ñb±¨ßï×¾j!vI;½µµåHùIÿQ~)^~ú^üÀ÷ööêUúVÓ¾j!vËK¹þ¸¯õx<6M?¥°°PJTSáÒ¥K_rú$gµOL@àøñã2ÎÍÍ¥üÊÀf(¿xfÓs8eee§O¿üDCCCKK6¥¾¾^GoooII	åÜåwåÊ¨Õö^Ê üÜóò«««óx<2èèè(--]µ½&''m6[ (,,s??Åï÷kSfffrrr._¾Z>°7//¯««òKîò-Z<ÌêØ^³Ù¼cÇ°Ý(?òpËÏçóÙív£Ñ(%§óã¡¡!ôõõUVV®éæN§~Ûí.))ÎÍÍ=þ|;¤)¿dAù_ü>ýÌÿbOà$å'§®èúÕqV;¤Bùé÷U®]»¶cÇ¼¼<ÊØ¦_¢í_ÿ4Ó:ÊZº++övfµ@j_ àØ^`3¾÷æçm÷'pZü¯ÿ¸âçÎj$.¿¼¼¼m1E=òdß×%Öl¹ò1,Ó>HO²¯³³ò(?@*F:o¾±Kù`ãË/ªùùùÚÚZÊ üÜ½uÝ·tÍæêêj¯×»w:aS¾øaqÑápX,£ÑòäI/¿ÜÜ°Ãöó;ìò(?wºü´q0ìêêª©©ÙÀè¿ÌM»¹ýû÷·´´,--ò<±#eË¯°°0òð	ùò6§ës¿ýÑÚN#¿øð«ÃºÏÿæûk½ú»V;p7ËO1Lµ49+y×ÐÐ9CØN_<)[~ò¬ÇÞëõfggË@oõáÝG¡üÍ)ðùÂ³/Ä½ÇóØßÌ>uZÊoÜõ]OûßÎ4­i	ÏO±ÚÍGN|øÁkë?Érb´Z0LKKSãùùùòòrI4»Ý.cm~yÉ6Í»ví|_8êW´Ùl6íËÜ´¬Vë©S§xX·Jùi_ö"©'ééiyªÉ@¶Q~@²ûìì_ßxú¤ü¼/¶ßZÿ´þ?ß5$ÆóVçÍÞ8½Õ·R«ÉkqSSÓÑ£GÕÙºº:UlzCÿðáÃ2g[[ÌOùjï k3z5???77WÜÝÝ=<<ÌCÊå!ýÀÀÀÄÄî¿ÿ~5àqãóOÿGù©Êïý¡ËàóºñìOY7ÀæzQ×ÉÌÌÓö¼²X,jà÷û³³³µùçææBË;çiÂ»Òùj'å'ýn·ûÜ¹s/^äáHÙò«¯¯×çÐ?KJKK)? Üúüúõûÿ75¾]~Ü~èþþ_ÿl¶ò'µC0õóGn¯ºÍOx<ÍãæÆÆÆrssy8R¶üDCCÚshhHò*..NlQ£££%%%òüÛ¹sçÀÀ@Ø999ê¢¾¾¾8§P~Àú]à¿Üü+ôå»½¡àMm¤(¿¨_¬%ó/,,·ùeeeÅY~êu¿¥¥Eõ3oÂ=¾°å·¤ØºººBËh¦ªªªnïiÔØØX]]ç°òâ'®[Þ÷íZü§zû¿ñ7®'û¤üÆ~Õän:ýÒ÷zþ·sñ/aô÷¬uà¶U~uuuGÚr2ÿñãÇeÐÖÖV__ùùýþââbmJmmíÙ³gÕòå"y	>vìEù­ÍåËõS$Õò§ÚÏ°òûÁ~ð°å=þô+Õ?Kà$å×ñØ#	¬v@Ü«òóù|v»Ýh4ÊkëÄÄ6KKÉdª¬¬Tÿâ_¸ú5öz½÷îMKK3òB|âÄ	y¦±R¹üvìØ¡>ÛeC>Éyii)==]ÐÞÞ®®ßAãÂ»½@¤7oy?¹ÿéíksC/>öúôß¿|døå_üvbpMKøÄdµIôî0(¿èkO³ÎczzÂö9Ð§¤úhÊx¦P~ÀúM¾Ö>ïû]hy?¿Ñ§.7Æ_m¹þé5Ö@ùaËÄ<uWØÞëÖjë±¶#j<S(?`TçÝºTå÷jo«æÞüàýWX9@R[Ï7|`ëÅbòÛ¨ì+((-&¼÷nýE555­­·_rägUUUS(?`ýå÷ÚÈù°ò×3@ùÀV,?·Û-åwôèÑUwsi;wî4eeeêã%C_nÈÎÎ6999ÒqN¡üõ|­ýCïoC_¾ÛóæçÒ×çX3°åÊOlß¾=òó¾>ÂãÎ¡üåÖÂ7â?:ðÇïýêÛR~o<zôíÎãô|wMK¸9õ2«R¡üòóóïÄpç¼óô³?üê?&p:Y~îå'¸âßæ)Q~ªùFFF6ÿoKùÊüÜÂG_ÿÔü­Á¬æWÍ®ÿáÜÿø³óMßzîÏ<ý£5,áµß³Ú;þ¢q¬î:zWët:£ÞÐââ¢Ãá°X,F£1??ÿäÉ<.©V~YYYxålB/ýhIR~ü~éñ÷~Ö°eË¯¤¤Äï÷GÞÐþýû[ZZnÿN,ã¡I©ò»rå<Þõõõúgå¤O?þmûûj,å×öÛå×9üÉ¥WY9À)¿Èor¼khh!l_¯ÅÅÅÌÌL*¿m+à eüMÛ5é?U~?;»ü¾ÿë'?cÍy»÷ì[O~ý'YNå'æææüü|ÏÏÏK¢Ùív«Ünwqq±LÌÊÊû­Ðßák³ÙfggÃf°Z­§N±_oJaá¤æþ$õn¿x·wb.pèÇ×|7Y3@b>Æ7Ú»þ,'þòëèèPïÎÕÕÕ©bs:C+¶þþ~tuueddÄS~555a3zUú277WÜÝÝ=<<ÌÃjåD(?@	|¾ðÞìñÞñ¼ÐñÌ¯Û¾r»ü¹üôÈøàðñü«¸ã/êå§½ÿ¦ß#ßb±¨`vvvåDOO?ô_äM»ÝîsçÎUTTÆ/òÐP~p/výýßO<¾¦ÓäØÏ^èûßÅï4¿òBÛZ¯þþÜK¬vàN3Íú³zÚEÝ5Ëçó:uJJÎjµ®º¿ vÖãñØl¶ÐÊ;åææòÐ¤BùiOöóRÞ»ÞSö£îû¥ü¿9púk?îäV°ÙOLLhgÇÇÇµïAÕYä¹¡åöº»»gffâ/?!×jiiÑ¦¤§§ÜGZZM*¶'ûù)ï|íãÏ]¼Z>Âãâ£#~ôYÃ^û½5l*.KROÒMÆÓÓÓöìéëëlµºº:Ç#ÒÒR5Ñl6ONNÃ±¦òóûýÅÅÅÚÚÚÚ³gÏªåËEÇã¡IòKFÀg7NýÙ7¿8¶÷±üFÏ¼Ðù*+Øl:;;·oßn4ågkkkÔVóù|v»]æ),,Ô¶vww[­Ö¦¦¦U?&l§Ó©Mñz½÷îMKK3åÄR<.)U~oì^»vmÇyyyå÷PÅ£Zù]øþíòëm¢üò[~d"ïö)£ñ¯.¾Ò;úòÝ^ÕoÍ±f`«_^^Þ¶¢î@JùÁ×nüÝ¿¾ÿ©öÇsûþýáGÞò;|ü¿:÷æ¡æ·×´v×«¸üfffÔÚ¼z°9y?¹Ù9üÉNmWÞð;¯Hù8ò«3?|ü¥5]øM¾ä¹ü4ó0^Ê¸¤ü®>9Îz­[~Iò(? üÖK¿Zþd ³Ùl4«ªª(?ò¤Zùíß¿_íçõGx<xò(?wÍ=Ün·vVÆ2%V¬ðµ¼kmÛNgØ5X\t8Åh4æçç<y)éË/==]`Ë5>>.«Õêñxd Ó)?òp×ige<22»Ø6&&¶m+))ñûýKÞ¿KKËÒÒRhùCßN,ãJîòÓþDg$íCÑ>äò6O|JÀ%pòk©ûyW|ëÕwYíÀ]°oß>õmýýý2^õ<áþ°³w3º¸¸ÉÃÜå&ðüü¼Úø7;;åÊ¶ùÙÛcs§¿öãNR~ßøOM	çû¬v@ó½ýíý;=üþ$lÉãããÅÅÅ2())Ñ¾MÈËtyy¹tÝnñªå'³¹UÕÕÕ«ü´ÙlÒa3X­ÖS§Nñ§TùíÝ»WÛ9 ;;;ôåV@öó ªO|ÎÏ-¬ÿôñû×ßÚÚÚè'ÖÕÕ©,s:êÝ¹PÌýüFGGKKKÁ ¤Ös±Ëopp°¦¦&l«W¯æçççææÊvwwóè'ù©?,dú3BÆ±w)¥ü¸C¦§§%¼¦¦¦ô-øý~µ&´Ú»½ÒåååaïáF-Em9R~ÒKv»ÝçÎ«¨¨0/^ä1JúòK`K¼ºG$¾Õ´ñcßðð°þ¸¯õx<6-ÆÇÆÆrssy(?Ê;X~ñÌ¦çp8ÊÊÊN>ùmJzzz0ÔÏ¬>ÉW~aGïÆ>KùpË¯®®Îãñ¿s¡´´tÕò´Ùl@ °°0Îýü¿ß_\¬M©­­=ö¬ºi¹¨±±ñØ±c<@åÀ,?Ïg·ÛF£vÌo#<***dÐ××WYY¹¦s:Ú¯×«¾èKªÀb±8qBjò£ü(?Êò£ü(?Êò£üåXùÅFù¤HùVc4)?T(¿dDùÊò ü(?Êò ü(?Êò ü(?Êòî?yîêéYë@ù***2n·[ÍÉÉvîÜÙ××çÊb)x;þ8©KàÝ`­å®  `ppPííí;vìÐ_TUUuáÂ466VWWÇ9ò6ô3OèýOY@ùm<³Ù¬?k±XÁ @nnnSÂÊïGùDIù½õÒk¬ ñDåËå:rä~þÕ8)aå÷øã$JÊïÃ	V°Dåiaa¡ªªjqqQ?Ñ`0hcÉç°òãÝ^`]x·(¿5;;èÐ¡¹¹¹°éYYY@ ´üN®ãBù`ß+WvïÞíõz#/ª©©immü¬ªªsål?ýöýÛå÷ÒlèÍBÁ[¬ üÖ+77wÎ÷ry000m0rrrâBùàë·Ïwývù½ûIhâÐk^ÖP~å$høÝÐGþÛõnï­PèêÞÏÊòRËRðvù)R~ï,Ü¼ýqhöÖP~Zn-oó»yëòoù ûßÏ®q¨P~z&>½õÑå÷þ§!ÿRÈ=û'ò£üMndáÃ]/>µÓ½»þÕ®g.Kù|aW_÷®çzÖ´oÿþ·¬v ü(?àXüøÐ«Ck;zñY)¿CO÷úÍàZ¯þ¨çV;P~T<Ï¿þ&«(?Ê üåP~Êò(?åGùò£üõûôFhìý5n¾÷æKÓ·?ÏoÈóÁËo¯õê_|óò£ü»í³¡7>ÿôñø/»_¬ÿ¥^zýçÏ½òÛ¿¦%ð@ùQ~@rØõâSg¦_»ý_Æm½3õAàó×çXü5å¤ðföÇnÝRå×<3!ÿ>>ÜôÖ+(?ÊH)KK¹¿îÔÊOmüàõWÎ½9ÎÊÊòRÍ¿¶«×;úòÝ^©@rå9ÖP~j.¿ÿåéý|nFÊïoÿÅËÏ~íj¿Ú ü(? Õ<ë¯t¨GÊ/³Ïù÷¯¹ýÁ¬ ü(? 	¼;<òóòÅ»yzáXí@ùQ~À=àÿø«/4	÷Æ+¬v ü(? Hù=ö¯Ï±ò£üÊ@ùQ~å ü(?àxïóÏg*_mó³	ùßgµåGù÷À³¾÷¶=ÙÀIÊo×g¸â¡WXí@ùQ~@rxwxäéc¿ò»RÿäKçYúüsÖ	P~¦zùñÎôÓ.)¿×~Ö÷ìýCýÿà¾Åg9åGù©çÉ¯Ïº^-áñz÷à­¥%mò£üÔøäÓîÿkZ¥ü~±W/?òÌoÛ9È(?ÊH-Á¥Ï»ÿò­×ýªü~÷_ËÀõÝ'aååGù©¦ÿÿö7MÏ¾|·÷cÏOüùÜGo½ÁÊòRÍâûï<ù÷¸Çßíøå_¼óú/ù§å¤¨¥Ï?ÿÝ/»]~ßéxÿµß±Bò£ü§Þíe=åGùò£üÊ@ùQ~å ü(?òP~°nM¿ùÂ?$pò»xïÚ¯8ñ_øå$Uù%|¢üò£ü(?Êò£ü(?Ê ÙÊÏëõæææFNw»Ý999F£qçÎqN¡ü6iùX­ÖmÛ¢Ü±ªªª.È ±±±ºº:Î)À&-?»Ý>11µü,K0A PãV~ßúÖ·.pP ü¢Ý­håg4ÃÆñL	+¿þþþ[Ü#(¿xËÏ`0hcÉç°òãÝ^@ù%AùeeeÐò;¹2så|åWSSÓÚÚ*ùYUUçÊ ÊOÈÎÎ6999CCCqN¡ü6uùÝ! ü(?Êò üÑ?ýÓ?ýìg?ä£>">@ùÝ%333ßøÆ7¾	À=Â[O ü@ùòåÊòåÊ¤ÿáL¦;w=6êÁl6555±êò|å§?ûûû´¶A;wµ$.?ellLâ/Æyyy¬= ü$ù	£ÑÉÄÚÊ@ÒßÀÀÀªÛüY@ùHâòóûýíííYYYmmmQgP;îØ±ãÒ¥K¬= ü$YùéeddTUUõöö®4CzzzEE~å iÊOÁàC=d·Û>_Ô)?åäÉ üòúå'vïÞÝØØHùå õËÏëõZ­ÖÊ(?)^~btt´¢¢òÊ(?P~ ü@ùòå@ùòåÊ(?P~ ü@ùò ü@ùòåàÞèéé±ÛíiiiF£1##ãàÁãããwö?e¬y üÜUíííÛ"¤§§ßÑø£üòplß¾]"¬¡¡aaaAÎÎÌÌØívRYYÉÊÊ@J1LÒyú-|G¦Íæ/þñ/»|ùraa¡ÑhÜ¹sgoo¯~	rÖjµÊE2Ì¦¿¨¯¯¯¨¨H.¥íÚµkzzZ¿ÌUàóù9.Y,cÇ-..òå AaVû÷ïïîîáÿøýÛÁ.K]:22²ÒEn·;ìMdm;¢¾üb,¡¢¢"l	Gå!Ê@¼^oQQ~?©«©©©°ò«ªª_XXP¥¨5L³2quuµºhß¾r¶¶¶6¨ÒÒÒ"Ë/ÆTÎÌÌÈXªT¿å AsF£Q5Ùlîïï×WÚÜÜ:+ê¬"Ñ_õýYùÅXJÒüü|ÉÇ®®®ë×¯óHå`c,--]ºt©¬¬LzKª+²Ò´)ÒúKõÃJWZ~1011¶=RîP~d±X$ª´c/D0l;mk×®é7Ë¥¥¥ÉÙ@ ¹dµ1O2£üb,A~àÔ»Ì999<d@ùHÐ#G¤¨î»ï¾	9+¡vüøq²sçN¥Ùív¯×ýúõ9»ÿ~u©²cÇI/öôôÈ¸°°P]¤öó«¯¯úúúdY~1 öóëîî-oÿ±Édâ!Ê@fff²²²"ßríììÔWÙlÖ.üÒ>Æår]±££C]ylïC=Y~1P]]vÑáÃyÈò¸éééCeffªÂ³ÛíúOìSÉ588XTTd0ôW¿|ù²LrrrÎ?¯¿H£®%544-sÕ%,..?~<;;[f»çp8ü~?P~îØ?~¾i(?òRLËX@ùòåÊ(?P~ ü@ùò ü@ùòåÊ(?P~6×ÿ/Ñlìòïèý_\<xð ÉdÊÌÌ¬««»víLQ£ã²ÞÞ^5ell¬´´4Æò'''ív»,?''§±±ñ®ý^(?¸#åÔËw8sss2Rf6MÆv÷îY5²oß¾+W®¬´pYr~~~gg§,ÜãñH#677S~(?ß½Y~ZZ6>3Í2hoo?vìX;££££÷Ýw_ì¬<sævvll¬¨¨ò@ùHÁòóóóÃÆ^¯wÏ=F£q÷îÝ>/ræ°LMMI0ÉÌrE5ÑívË²²²¤Ò" Þ51îªÉdàSã@  geP[[ÛÑÑ9sØ6¿máQÌÌÌÜ¢@ùÀ)¿öóôßïKWµµµ©>;räHäÌaË¯««8s¹Ñjµö÷÷Ë ««+###r	Rfªûúú"kRïàÁ²|¹¢,_Ýù½÷J¦-[_\T3«ærÏGGGËÊÊb¯íMaÊå EÊ/ÆEÚæ4ý833Så§¾Û´Â¢m+Æ¸ú%Hºíß¿_²oii)öý¯©©DËËË»téÚæ'÷J£,PâRê0òrCCCÇ|P~(?[´üVkm´Òr¢Î,-xêÔ))6«Õ¶ï6zC9==dd$Îßerr2'''rºÊA½ááaµÁ/ò½ÂÂÂééií¬t¤Ä%åò°Ê/##Cm2[µü®_¿®f¶X,j¢ÍfkhhèîîZ~Zi]¸p!jÌEÕÕÕµÿþÈDÝnEó¦¾¾^bII	åò°ÊïðáÃê½T)!ý~~+-äë_ÿºh6'''LÚ^V«µ§§'ôåy1î¿Ì)Á'ééié¹«W¯ªrs*ûÚÚÚÂöt»ÝÚ~±·ùIJw^¾|9´|`o^^º-Êå YË/ÆQËÏëõîÞ½[RiÏÿß¾ÝÚ@JÀ¢*YÓ%Xª¢;àXØ/©Apó÷<Ò÷&¤ëú¼ÛûiþèªiJ)×uõÁã8"ÎÆq¬µ¾.qs¡ßÆø²D¤Þ²,ñÙ<Ïû¾÷ÁÈÊØaJ)sÛ¶çÛ¯ÎóìÏÏ;¯ûôùã­µ_@ù üP~Êåò@ù üP~(?Êåðwn=¿O¿À¸4IEND®B`


SPLIT FILE OFF.
UNIANOVA Bio BY Species NitroS
  /METHOD=SSTYPE(3)
  /INTERCEPT=INCLUDE
  /POSTHOC=Species NitroS(TUKEY)
  /PLOT=PROFILE(Species*NitroS) TYPE=LINE ERRORBAR=CI MEANREFERENCE=NO YAXIS=AUTO
  /EMMEANS=TABLES(OVERALL)
  /EMMEANS=TABLES(Species) COMPARE ADJ(LSD)
  /EMMEANS=TABLES(NitroS) COMPARE ADJ(LSD)
  /EMMEANS=TABLES(Species*NitroS)
  /PRINT ETASQ DESCRIPTIVE HOMOGENEITY
  /CRITERIA=ALPHA(.05)
  /DESIGN=Species NitroS Species*NitroS.


Univariate Analysis of Variance


Notes	
Output Created	10-MAY-2026 19:43:32	
Comments		
Input	Active Dataset	DataSet5	
	Filter	<none>	
	Weight	<none>	
	Split File	<none>	
	N of Rows in Working Data File	84	
Missing Value Handling	Definition of Missing	User-defined missing values are treated as missing.	
	Cases Used	Statistics are based on all cases with valid data for all variables in the model.	
Syntax	UNIANOVA Bio BY Species NitroS
  /METHOD=SSTYPE(3)
  /INTERCEPT=INCLUDE
  /POSTHOC=Species NitroS(TUKEY)
  /PLOT=PROFILE(Species*NitroS) TYPE=LINE ERRORBAR=CI MEANREFERENCE=NO YAXIS=AUTO
  /EMMEANS=TABLES(OVERALL)
  /EMMEANS=TABLES(Species) COMPARE ADJ(LSD)
  /EMMEANS=TABLES(NitroS) COMPARE ADJ(LSD)
  /EMMEANS=TABLES(Species*NitroS)
  /PRINT ETASQ DESCRIPTIVE HOMOGENEITY
  /CRITERIA=ALPHA(.05)
  /DESIGN=Species NitroS Species*NitroS.	
Resources	Processor Time	00:00:00.47	
	Elapsed Time	00:00:01.11	


Warnings	
Post hoc tests are not performed for Species because there are fewer than three groups.	


Between-Subjects Factors	
	Value Label	N	
Species	1	RK	42	
	2	RP	42	
NitroS	1	Pep	6	
	2	Urea	6	
	3	YeEx	6	
	4	NCl	6	
	5	NS	6	
	6	Pep+YeEx	6	
	7	Urea+Pep	6	
	8	YeEx+Urea	6	
	9	Pep+NCl	6	
	10	Urea+NCl	6	
	11	YeEx+NCl	6	
	12	Pep+NS	6	
	13	Urea+NS	6	
	14	YeEx+NS	6	


Descriptive Statistics	
Dependent Variable:   Bio  	
Species	NitroS	Mean	Std. Deviation	N	
RK	Pep	2.50267	.168560	3	
	Urea	5.05400	.068022	3	
	YeEx	2.59967	.148796	3	
	NCl	2.72600	.121606	3	
	NS	2.76867	.084347	3	
	Pep+YeEx	1.23833	.089075	3	
	Urea+Pep	3.14467	.096095	3	
	YeEx+Urea	1.65333	.112167	3	
	Pep+NCl	2.81400	.161037	3	
	Urea+NCl	3.38367	.150869	3	
	YeEx+NCl	6.28200	.161130	3	
	Pep+NS	1.55867	.090046	3	
	Urea+NS	2.24567	.111006	3	
	YeEx+NS	3.87167	.106039	3	
	Total	2.98879	1.330495	42	
RP	Pep	2.80200	.203443	3	
	Urea	4.63400	.139097	3	
	YeEx	4.12700	.089152	3	
	NCl	3.05833	.028378	3	
	NS	1.33567	.087888	3	
	Pep+YeEx	1.61533	.129778	3	
	Urea+Pep	1.86833	.106214	3	
	YeEx+Urea	1.33633	.111433	3	
	Pep+NCl	3.46167	.205510	3	
	Urea+NCl	6.56500	.323561	3	
	YeEx+NCl	4.14733	.077184	3	
	Pep+NS	2.92867	.161401	3	
	Urea+NS	3.46833	.257250	3	
	YeEx+NS	2.66100	.104504	3	
	Total	3.14350	1.412249	42	
Total	Pep	2.65233	.234095	6	
	Urea	4.84400	.250020	6	
	YeEx	3.36333	.843718	6	
	NCl	2.89217	.198421	6	
	NS	2.05217	.788658	6	
	Pep+YeEx	1.42683	.229236	6	
	Urea+Pep	2.50650	.704921	6	
	YeEx+Urea	1.49483	.200365	6	
	Pep+NCl	3.13783	.391291	6	
	Urea+NCl	4.97433	1.757056	6	
	YeEx+NCl	5.21467	1.174653	6	
	Pep+NS	2.24367	.759430	6	
	Urea+NS	2.85700	.692729	6	
	YeEx+NS	3.26633	.669761	6	
	Total	3.06614	1.365910	84	


Levene's Test of Equality of Error Variancesa,b	
	Levene Statistic	df1	df2	Sig.	
Bio	Based on Mean	1.177	27	56	.297	
	Based on Median	.466	27	56	.984	
	Based on Median and with adjusted df	.466	27	22.940	.971	
	Based on trimmed mean	1.121	27	56	.351	

Tests the null hypothesis that the error variance of the dependent variable is equal across groups.a,b	
a. Dependent variable: Bio	
b. Design: Intercept + Species + NitroS + Species * NitroS	


Tests of Between-Subjects Effects	
Dependent Variable:   Bio  	
Source	Type III Sum of Squares	df	Mean Square	F	Sig.	Partial Eta Squared	
Corrected Model	153.680a	27	5.692	271.524	.000	.992	
Intercept	789.703	1	789.703	37672.050	.000	.999	
Species	.503	1	.503	23.979	.000	.300	
NitroS	113.826	13	8.756	417.691	.000	.990	
Species * NitroS	39.351	13	3.027	144.400	.000	.971	
Error	1.174	56	.021				
Total	944.557	84					
Corrected Total	154.854	83					

a. R Squared = .992 (Adjusted R Squared = .989)	


Estimated Marginal Means


1. Grand Mean	
Dependent Variable:   Bio  	
Mean	Std. Error	95% Confidence Interval	
		Lower Bound	Upper Bound	
3.066	.016	3.034	3.098	


2. Species


Estimates	
Dependent Variable:   Bio  	
Species	Mean	Std. Error	95% Confidence Interval	
			Lower Bound	Upper Bound	
RK	2.989	.022	2.944	3.034	
RP	3.143	.022	3.099	3.188	


Pairwise Comparisons	
Dependent Variable:   Bio  	
(I) Species	(J) Species	Mean Difference (I-J)	Std. Error	Sig.b	95% Confidence Interval for Differenceb	
					Lower Bound	Upper Bound	
RK	RP	-.155*	.032	.000	-.218	-.091	
RP	RK	.155*	.032	.000	.091	.218	

Based on estimated marginal means	
*. The mean difference is significant at the .05 level.	
b. Adjustment for multiple comparisons: Least Significant Difference (equivalent to no adjustments).	


Univariate Tests	
Dependent Variable:   Bio  	
	Sum of Squares	df	Mean Square	F	Sig.	Partial Eta Squared	
Contrast	.503	1	.503	23.979	.000	.300	
Error	1.174	56	.021				

The F tests the effect of Species. This test is based on the linearly independent pairwise comparisons among the estimated marginal means.	


3. NitroS


Estimates	
Dependent Variable:   Bio  	
NitroS	Mean	Std. Error	95% Confidence Interval	
			Lower Bound	Upper Bound	
Pep	2.652	.059	2.534	2.771	
Urea	4.844	.059	4.726	4.962	
YeEx	3.363	.059	3.245	3.482	
NCl	2.892	.059	2.774	3.011	
NS	2.052	.059	1.934	2.171	
Pep+YeEx	1.427	.059	1.308	1.545	
Urea+Pep	2.507	.059	2.388	2.625	
YeEx+Urea	1.495	.059	1.376	1.613	
Pep+NCl	3.138	.059	3.019	3.256	
Urea+NCl	4.974	.059	4.856	5.093	
YeEx+NCl	5.215	.059	5.096	5.333	
Pep+NS	2.244	.059	2.125	2.362	
Urea+NS	2.857	.059	2.739	2.975	
YeEx+NS	3.266	.059	3.148	3.385	


Pairwise Comparisons	
Dependent Variable:   Bio  	
(I) NitroS	(J) NitroS	Mean Difference (I-J)	Std. Error	Sig.b	95% Confidence Interval for Differenceb	
					Lower Bound	Upper Bound	
Pep	Urea	-2.192*	.084	.000	-2.359	-2.024	
	YeEx	-.711*	.084	.000	-.878	-.544	
	NCl	-.240*	.084	.006	-.407	-.072	
	NS	.600*	.084	.000	.433	.768	
	Pep+YeEx	1.225*	.084	.000	1.058	1.393	
	Urea+Pep	.146	.084	.087	-.022	.313	
	YeEx+Urea	1.157*	.084	.000	.990	1.325	
	Pep+NCl	-.486*	.084	.000	-.653	-.318	
	Urea+NCl	-2.322*	.084	.000	-2.489	-2.155	
	YeEx+NCl	-2.562*	.084	.000	-2.730	-2.395	
	Pep+NS	.409*	.084	.000	.241	.576	
	Urea+NS	-.205*	.084	.018	-.372	-.037	
	YeEx+NS	-.614*	.084	.000	-.781	-.447	
Urea	Pep	2.192*	.084	.000	2.024	2.359	
	YeEx	1.481*	.084	.000	1.313	1.648	
	NCl	1.952*	.084	.000	1.784	2.119	
	NS	2.792*	.084	.000	2.624	2.959	
	Pep+YeEx	3.417*	.084	.000	3.250	3.585	
	Urea+Pep	2.338*	.084	.000	2.170	2.505	
	YeEx+Urea	3.349*	.084	.000	3.182	3.517	
	Pep+NCl	1.706*	.084	.000	1.539	1.874	
	Urea+NCl	-.130	.084	.125	-.298	.037	
	YeEx+NCl	-.371*	.084	.000	-.538	-.203	
	Pep+NS	2.600*	.084	.000	2.433	2.768	
	Urea+NS	1.987*	.084	.000	1.820	2.154	
	YeEx+NS	1.578*	.084	.000	1.410	1.745	
YeEx	Pep	.711*	.084	.000	.544	.878	
	Urea	-1.481*	.084	.000	-1.648	-1.313	
	NCl	.471*	.084	.000	.304	.639	
	NS	1.311*	.084	.000	1.144	1.479	
	Pep+YeEx	1.936*	.084	.000	1.769	2.104	
	Urea+Pep	.857*	.084	.000	.689	1.024	
	YeEx+Urea	1.868*	.084	.000	1.701	2.036	
	Pep+NCl	.226*	.084	.009	.058	.393	
	Urea+NCl	-1.611*	.084	.000	-1.778	-1.444	
	YeEx+NCl	-1.851*	.084	.000	-2.019	-1.684	
	Pep+NS	1.120*	.084	.000	.952	1.287	
	Urea+NS	.506*	.084	.000	.339	.674	
	YeEx+NS	.097	.084	.251	-.070	.264	
NCl	Pep	.240*	.084	.006	.072	.407	
	Urea	-1.952*	.084	.000	-2.119	-1.784	
	YeEx	-.471*	.084	.000	-.639	-.304	
	NS	.840*	.084	.000	.673	1.007	
	Pep+YeEx	1.465*	.084	.000	1.298	1.633	
	Urea+Pep	.386*	.084	.000	.218	.553	
	YeEx+Urea	1.397*	.084	.000	1.230	1.565	
	Pep+NCl	-.246*	.084	.005	-.413	-.078	
	Urea+NCl	-2.082*	.084	.000	-2.250	-1.915	
	YeEx+NCl	-2.323*	.084	.000	-2.490	-2.155	
	Pep+NS	.649*	.084	.000	.481	.816	
	Urea+NS	.035	.084	.676	-.132	.203	
	YeEx+NS	-.374*	.084	.000	-.542	-.207	
NS	Pep	-.600*	.084	.000	-.768	-.433	
	Urea	-2.792*	.084	.000	-2.959	-2.624	
	YeEx	-1.311*	.084	.000	-1.479	-1.144	
	NCl	-.840*	.084	.000	-1.007	-.673	
	Pep+YeEx	.625*	.084	.000	.458	.793	
	Urea+Pep	-.454*	.084	.000	-.622	-.287	
	YeEx+Urea	.557*	.084	.000	.390	.725	
	Pep+NCl	-1.086*	.084	.000	-1.253	-.918	
	Urea+NCl	-2.922*	.084	.000	-3.090	-2.755	
	YeEx+NCl	-3.163*	.084	.000	-3.330	-2.995	
	Pep+NS	-.191*	.084	.026	-.359	-.024	
	Urea+NS	-.805*	.084	.000	-.972	-.637	
	YeEx+NS	-1.214*	.084	.000	-1.382	-1.047	
Pep+YeEx	Pep	-1.225*	.084	.000	-1.393	-1.058	
	Urea	-3.417*	.084	.000	-3.585	-3.250	
	YeEx	-1.936*	.084	.000	-2.104	-1.769	
	NCl	-1.465*	.084	.000	-1.633	-1.298	
	NS	-.625*	.084	.000	-.793	-.458	
	Urea+Pep	-1.080*	.084	.000	-1.247	-.912	
	YeEx+Urea	-.068	.084	.419	-.235	.099	
	Pep+NCl	-1.711*	.084	.000	-1.878	-1.544	
	Urea+NCl	-3.547*	.084	.000	-3.715	-3.380	
	YeEx+NCl	-3.788*	.084	.000	-3.955	-3.620	
	Pep+NS	-.817*	.084	.000	-.984	-.649	
	Urea+NS	-1.430*	.084	.000	-1.598	-1.263	
	YeEx+NS	-1.839*	.084	.000	-2.007	-1.672	
Urea+Pep	Pep	-.146	.084	.087	-.313	.022	
	Urea	-2.337*	.084	.000	-2.505	-2.170	
	YeEx	-.857*	.084	.000	-1.024	-.689	
	NCl	-.386*	.084	.000	-.553	-.218	
	NS	.454*	.084	.000	.287	.622	
	Pep+YeEx	1.080*	.084	.000	.912	1.247	
	YeEx+Urea	1.012*	.084	.000	.844	1.179	
	Pep+NCl	-.631*	.084	.000	-.799	-.464	
	Urea+NCl	-2.468*	.084	.000	-2.635	-2.300	
	YeEx+NCl	-2.708*	.084	.000	-2.876	-2.541	
	Pep+NS	.263*	.084	.003	.095	.430	
	Urea+NS	-.350*	.084	.000	-.518	-.183	
	YeEx+NS	-.760*	.084	.000	-.927	-.592	
YeEx+Urea	Pep	-1.157*	.084	.000	-1.325	-.990	
	Urea	-3.349*	.084	.000	-3.517	-3.182	
	YeEx	-1.868*	.084	.000	-2.036	-1.701	
	NCl	-1.397*	.084	.000	-1.565	-1.230	
	NS	-.557*	.084	.000	-.725	-.390	
	Pep+YeEx	.068	.084	.419	-.099	.235	
	Urea+Pep	-1.012*	.084	.000	-1.179	-.844	
	Pep+NCl	-1.643*	.084	.000	-1.810	-1.476	
	Urea+NCl	-3.479*	.084	.000	-3.647	-3.312	
	YeEx+NCl	-3.720*	.084	.000	-3.887	-3.552	
	Pep+NS	-.749*	.084	.000	-.916	-.581	
	Urea+NS	-1.362*	.084	.000	-1.530	-1.195	
	YeEx+NS	-1.771*	.084	.000	-1.939	-1.604	
Pep+NCl	Pep	.486*	.084	.000	.318	.653	
	Urea	-1.706*	.084	.000	-1.874	-1.539	
	YeEx	-.226*	.084	.009	-.393	-.058	
	NCl	.246*	.084	.005	.078	.413	
	NS	1.086*	.084	.000	.918	1.253	
	Pep+YeEx	1.711*	.084	.000	1.544	1.878	
	Urea+Pep	.631*	.084	.000	.464	.799	
	YeEx+Urea	1.643*	.084	.000	1.476	1.810	
	Urea+NCl	-1.836*	.084	.000	-2.004	-1.669	
	YeEx+NCl	-2.077*	.084	.000	-2.244	-1.909	
	Pep+NS	.894*	.084	.000	.727	1.062	
	Urea+NS	.281*	.084	.001	.113	.448	
	YeEx+NS	-.128	.084	.130	-.296	.039	
Urea+NCl	Pep	2.322*	.084	.000	2.155	2.489	
	Urea	.130	.084	.125	-.037	.298	
	YeEx	1.611*	.084	.000	1.444	1.778	
	NCl	2.082*	.084	.000	1.915	2.250	
	NS	2.922*	.084	.000	2.755	3.090	
	Pep+YeEx	3.547*	.084	.000	3.380	3.715	
	Urea+Pep	2.468*	.084	.000	2.300	2.635	
	YeEx+Urea	3.479*	.084	.000	3.312	3.647	
	Pep+NCl	1.837*	.084	.000	1.669	2.004	
	YeEx+NCl	-.240*	.084	.006	-.408	-.073	
	Pep+NS	2.731*	.084	.000	2.563	2.898	
	Urea+NS	2.117*	.084	.000	1.950	2.285	
	YeEx+NS	1.708*	.084	.000	1.541	1.875	
YeEx+NCl	Pep	2.562*	.084	.000	2.395	2.730	
	Urea	.371*	.084	.000	.203	.538	
	YeEx	1.851*	.084	.000	1.684	2.019	
	NCl	2.323*	.084	.000	2.155	2.490	
	NS	3.163*	.084	.000	2.995	3.330	
	Pep+YeEx	3.788*	.084	.000	3.620	3.955	
	Urea+Pep	2.708*	.084	.000	2.541	2.876	
	YeEx+Urea	3.720*	.084	.000	3.552	3.887	
	Pep+NCl	2.077*	.084	.000	1.909	2.244	
	Urea+NCl	.240*	.084	.006	.073	.408	
	Pep+NS	2.971*	.084	.000	2.804	3.138	
	Urea+NS	2.358*	.084	.000	2.190	2.525	
	YeEx+NS	1.948*	.084	.000	1.781	2.116	
Pep+NS	Pep	-.409*	.084	.000	-.576	-.241	
	Urea	-2.600*	.084	.000	-2.768	-2.433	
	YeEx	-1.120*	.084	.000	-1.287	-.952	
	NCl	-.649*	.084	.000	-.816	-.481	
	NS	.191*	.084	.026	.024	.359	
	Pep+YeEx	.817*	.084	.000	.649	.984	
	Urea+Pep	-.263*	.084	.003	-.430	-.095	
	YeEx+Urea	.749*	.084	.000	.581	.916	
	Pep+NCl	-.894*	.084	.000	-1.062	-.727	
	Urea+NCl	-2.731*	.084	.000	-2.898	-2.563	
	YeEx+NCl	-2.971*	.084	.000	-3.138	-2.804	
	Urea+NS	-.613*	.084	.000	-.781	-.446	
	YeEx+NS	-1.023*	.084	.000	-1.190	-.855	
Urea+NS	Pep	.205*	.084	.018	.037	.372	
	Urea	-1.987*	.084	.000	-2.154	-1.820	
	YeEx	-.506*	.084	.000	-.674	-.339	
	NCl	-.035	.084	.676	-.203	.132	
	NS	.805*	.084	.000	.637	.972	
	Pep+YeEx	1.430*	.084	.000	1.263	1.598	
	Urea+Pep	.350*	.084	.000	.183	.518	
	YeEx+Urea	1.362*	.084	.000	1.195	1.530	
	Pep+NCl	-.281*	.084	.001	-.448	-.113	
	Urea+NCl	-2.117*	.084	.000	-2.285	-1.950	
	YeEx+NCl	-2.358*	.084	.000	-2.525	-2.190	
	Pep+NS	.613*	.084	.000	.446	.781	
	YeEx+NS	-.409*	.084	.000	-.577	-.242	
YeEx+NS	Pep	.614*	.084	.000	.447	.781	
	Urea	-1.578*	.084	.000	-1.745	-1.410	
	YeEx	-.097	.084	.251	-.264	.070	
	NCl	.374*	.084	.000	.207	.542	
	NS	1.214*	.084	.000	1.047	1.382	
	Pep+YeEx	1.839*	.084	.000	1.672	2.007	
	Urea+Pep	.760*	.084	.000	.592	.927	
	YeEx+Urea	1.771*	.084	.000	1.604	1.939	
	Pep+NCl	.128	.084	.130	-.039	.296	
	Urea+NCl	-1.708*	.084	.000	-1.875	-1.541	
	YeEx+NCl	-1.948*	.084	.000	-2.116	-1.781	
	Pep+NS	1.023*	.084	.000	.855	1.190	
	Urea+NS	.409*	.084	.000	.242	.577	

Based on estimated marginal means	
*. The mean difference is significant at the .05 level.	
b. Adjustment for multiple comparisons: Least Significant Difference (equivalent to no adjustments).	


Univariate Tests	
Dependent Variable:   Bio  	
	Sum of Squares	df	Mean Square	F	Sig.	Partial Eta Squared	
Contrast	113.826	13	8.756	417.691	.000	.990	
Error	1.174	56	.021				

The F tests the effect of NitroS. This test is based on the linearly independent pairwise comparisons among the estimated marginal means.	


4. Species * NitroS	
Dependent Variable:   Bio  	
Species	NitroS	Mean	Std. Error	95% Confidence Interval	
				Lower Bound	Upper Bound	
RK	Pep	2.503	.084	2.335	2.670	
	Urea	5.054	.084	4.887	5.221	
	YeEx	2.600	.084	2.432	2.767	
	NCl	2.726	.084	2.559	2.893	
	NS	2.769	.084	2.601	2.936	
	Pep+YeEx	1.238	.084	1.071	1.406	
	Urea+Pep	3.145	.084	2.977	3.312	
	YeEx+Urea	1.653	.084	1.486	1.821	
	Pep+NCl	2.814	.084	2.647	2.981	
	Urea+NCl	3.384	.084	3.216	3.551	
	YeEx+NCl	6.282	.084	6.115	6.449	
	Pep+NS	1.559	.084	1.391	1.726	
	Urea+NS	2.246	.084	2.078	2.413	
	YeEx+NS	3.872	.084	3.704	4.039	
RP	Pep	2.802	.084	2.635	2.969	
	Urea	4.634	.084	4.467	4.801	
	YeEx	4.127	.084	3.960	4.294	
	NCl	3.058	.084	2.891	3.226	
	NS	1.336	.084	1.168	1.503	
	Pep+YeEx	1.615	.084	1.448	1.783	
	Urea+Pep	1.868	.084	1.701	2.036	
	YeEx+Urea	1.336	.084	1.169	1.504	
	Pep+NCl	3.462	.084	3.294	3.629	
	Urea+NCl	6.565	.084	6.398	6.732	
	YeEx+NCl	4.147	.084	3.980	4.315	
	Pep+NS	2.929	.084	2.761	3.096	
	Urea+NS	3.468	.084	3.301	3.636	
	YeEx+NS	2.661	.084	2.494	2.828	


Post Hoc Tests


NitroS


Multiple Comparisons	
Dependent Variable:   Bio  	
Tukey HSD  	
(I) NitroS	(J) NitroS	Mean Difference (I-J)	Std. Error	Sig.	95% Confidence Interval	
					Lower Bound	Upper Bound	
Pep	Urea	-2.19167*	.083591	.000	-2.48464	-1.89869	
	YeEx	-.71100*	.083591	.000	-1.00398	-.41802	
	NCl	-.23983	.083591	.222	-.53281	.05314	
	NS	.60017*	.083591	.000	.30719	.89314	
	Pep+YeEx	1.22550*	.083591	.000	.93252	1.51848	
	Urea+Pep	.14583	.083591	.890	-.14714	.43881	
	YeEx+Urea	1.15750*	.083591	.000	.86452	1.45048	
	Pep+NCl	-.48550*	.083591	.000	-.77848	-.19252	
	Urea+NCl	-2.32200*	.083591	.000	-2.61498	-2.02902	
	YeEx+NCl	-2.56233*	.083591	.000	-2.85531	-2.26936	
	Pep+NS	.40867*	.083591	.001	.11569	.70164	
	Urea+NS	-.20467	.083591	.460	-.49764	.08831	
	YeEx+NS	-.61400*	.083591	.000	-.90698	-.32102	
Urea	Pep	2.19167*	.083591	.000	1.89869	2.48464	
	YeEx	1.48067*	.083591	.000	1.18769	1.77364	
	NCl	1.95183*	.083591	.000	1.65886	2.24481	
	NS	2.79183*	.083591	.000	2.49886	3.08481	
	Pep+YeEx	3.41717*	.083591	.000	3.12419	3.71014	
	Urea+Pep	2.33750*	.083591	.000	2.04452	2.63048	
	YeEx+Urea	3.34917*	.083591	.000	3.05619	3.64214	
	Pep+NCl	1.70617*	.083591	.000	1.41319	1.99914	
	Urea+NCl	-.13033	.083591	.949	-.42331	.16264	
	YeEx+NCl	-.37067*	.083591	.003	-.66364	-.07769	
	Pep+NS	2.60033*	.083591	.000	2.30736	2.89331	
	Urea+NS	1.98700*	.083591	.000	1.69402	2.27998	
	YeEx+NS	1.57767*	.083591	.000	1.28469	1.87064	
YeEx	Pep	.71100*	.083591	.000	.41802	1.00398	
	Urea	-1.48067*	.083591	.000	-1.77364	-1.18769	
	NCl	.47117*	.083591	.000	.17819	.76414	
	NS	1.31117*	.083591	.000	1.01819	1.60414	
	Pep+YeEx	1.93650*	.083591	.000	1.64352	2.22948	
	Urea+Pep	.85683*	.083591	.000	.56386	1.14981	
	YeEx+Urea	1.86850*	.083591	.000	1.57552	2.16148	
	Pep+NCl	.22550	.083591	.307	-.06748	.51848	
	Urea+NCl	-1.61100*	.083591	.000	-1.90398	-1.31802	
	YeEx+NCl	-1.85133*	.083591	.000	-2.14431	-1.55836	
	Pep+NS	1.11967*	.083591	.000	.82669	1.41264	
	Urea+NS	.50633*	.083591	.000	.21336	.79931	
	YeEx+NS	.09700	.083591	.996	-.19598	.38998	
NCl	Pep	.23983	.083591	.222	-.05314	.53281	
	Urea	-1.95183*	.083591	.000	-2.24481	-1.65886	
	YeEx	-.47117*	.083591	.000	-.76414	-.17819	
	NS	.84000*	.083591	.000	.54702	1.13298	
	Pep+YeEx	1.46533*	.083591	.000	1.17236	1.75831	
	Urea+Pep	.38567*	.083591	.002	.09269	.67864	
	YeEx+Urea	1.39733*	.083591	.000	1.10436	1.69031	
	Pep+NCl	-.24567	.083591	.192	-.53864	.04731	
	Urea+NCl	-2.08217*	.083591	.000	-2.37514	-1.78919	
	YeEx+NCl	-2.32250*	.083591	.000	-2.61548	-2.02952	
	Pep+NS	.64850*	.083591	.000	.35552	.94148	
	Urea+NS	.03517	.083591	1.000	-.25781	.32814	
	YeEx+NS	-.37417*	.083591	.003	-.66714	-.08119	
NS	Pep	-.60017*	.083591	.000	-.89314	-.30719	
	Urea	-2.79183*	.083591	.000	-3.08481	-2.49886	
	YeEx	-1.31117*	.083591	.000	-1.60414	-1.01819	
	NCl	-.84000*	.083591	.000	-1.13298	-.54702	
	Pep+YeEx	.62533*	.083591	.000	.33236	.91831	
	Urea+Pep	-.45433*	.083591	.000	-.74731	-.16136	
	YeEx+Urea	.55733*	.083591	.000	.26436	.85031	
	Pep+NCl	-1.08567*	.083591	.000	-1.37864	-.79269	
	Urea+NCl	-2.92217*	.083591	.000	-3.21514	-2.62919	
	YeEx+NCl	-3.16250*	.083591	.000	-3.45548	-2.86952	
	Pep+NS	-.19150	.083591	.568	-.48448	.10148	
	Urea+NS	-.80483*	.083591	.000	-1.09781	-.51186	
	YeEx+NS	-1.21417*	.083591	.000	-1.50714	-.92119	
Pep+YeEx	Pep	-1.22550*	.083591	.000	-1.51848	-.93252	
	Urea	-3.41717*	.083591	.000	-3.71014	-3.12419	
	YeEx	-1.93650*	.083591	.000	-2.22948	-1.64352	
	NCl	-1.46533*	.083591	.000	-1.75831	-1.17236	
	NS	-.62533*	.083591	.000	-.91831	-.33236	
	Urea+Pep	-1.07967*	.083591	.000	-1.37264	-.78669	
	YeEx+Urea	-.06800	.083591	1.000	-.36098	.22498	
	Pep+NCl	-1.71100*	.083591	.000	-2.00398	-1.41802	
	Urea+NCl	-3.54750*	.083591	.000	-3.84048	-3.25452	
	YeEx+NCl	-3.78783*	.083591	.000	-4.08081	-3.49486	
	Pep+NS	-.81683*	.083591	.000	-1.10981	-.52386	
	Urea+NS	-1.43017*	.083591	.000	-1.72314	-1.13719	
	YeEx+NS	-1.83950*	.083591	.000	-2.13248	-1.54652	
Urea+Pep	Pep	-.14583	.083591	.890	-.43881	.14714	
	Urea	-2.33750*	.083591	.000	-2.63048	-2.04452	
	YeEx	-.85683*	.083591	.000	-1.14981	-.56386	
	NCl	-.38567*	.083591	.002	-.67864	-.09269	
	NS	.45433*	.083591	.000	.16136	.74731	
	Pep+YeEx	1.07967*	.083591	.000	.78669	1.37264	
	YeEx+Urea	1.01167*	.083591	.000	.71869	1.30464	
	Pep+NCl	-.63133*	.083591	.000	-.92431	-.33836	
	Urea+NCl	-2.46783*	.083591	.000	-2.76081	-2.17486	
	YeEx+NCl	-2.70817*	.083591	.000	-3.00114	-2.41519	
	Pep+NS	.26283	.083591	.123	-.03014	.55581	
	Urea+NS	-.35050*	.083591	.007	-.64348	-.05752	
	YeEx+NS	-.75983*	.083591	.000	-1.05281	-.46686	
YeEx+Urea	Pep	-1.15750*	.083591	.000	-1.45048	-.86452	
	Urea	-3.34917*	.083591	.000	-3.64214	-3.05619	
	YeEx	-1.86850*	.083591	.000	-2.16148	-1.57552	
	NCl	-1.39733*	.083591	.000	-1.69031	-1.10436	
	NS	-.55733*	.083591	.000	-.85031	-.26436	
	Pep+YeEx	.06800	.083591	1.000	-.22498	.36098	
	Urea+Pep	-1.01167*	.083591	.000	-1.30464	-.71869	
	Pep+NCl	-1.64300*	.083591	.000	-1.93598	-1.35002	
	Urea+NCl	-3.47950*	.083591	.000	-3.77248	-3.18652	
	YeEx+NCl	-3.71983*	.083591	.000	-4.01281	-3.42686	
	Pep+NS	-.74883*	.083591	.000	-1.04181	-.45586	
	Urea+NS	-1.36217*	.083591	.000	-1.65514	-1.06919	
	YeEx+NS	-1.77150*	.083591	.000	-2.06448	-1.47852	
Pep+NCl	Pep	.48550*	.083591	.000	.19252	.77848	
	Urea	-1.70617*	.083591	.000	-1.99914	-1.41319	
	YeEx	-.22550	.083591	.307	-.51848	.06748	
	NCl	.24567	.083591	.192	-.04731	.53864	
	NS	1.08567*	.083591	.000	.79269	1.37864	
	Pep+YeEx	1.71100*	.083591	.000	1.41802	2.00398	
	Urea+Pep	.63133*	.083591	.000	.33836	.92431	
	YeEx+Urea	1.64300*	.083591	.000	1.35002	1.93598	
	Urea+NCl	-1.83650*	.083591	.000	-2.12948	-1.54352	
	YeEx+NCl	-2.07683*	.083591	.000	-2.36981	-1.78386	
	Pep+NS	.89417*	.083591	.000	.60119	1.18714	
	Urea+NS	.28083	.083591	.073	-.01214	.57381	
	YeEx+NS	-.12850	.083591	.954	-.42148	.16448	
Urea+NCl	Pep	2.32200*	.083591	.000	2.02902	2.61498	
	Urea	.13033	.083591	.949	-.16264	.42331	
	YeEx	1.61100*	.083591	.000	1.31802	1.90398	
	NCl	2.08217*	.083591	.000	1.78919	2.37514	
	NS	2.92217*	.083591	.000	2.62919	3.21514	
	Pep+YeEx	3.54750*	.083591	.000	3.25452	3.84048	
	Urea+Pep	2.46783*	.083591	.000	2.17486	2.76081	
	YeEx+Urea	3.47950*	.083591	.000	3.18652	3.77248	
	Pep+NCl	1.83650*	.083591	.000	1.54352	2.12948	
	YeEx+NCl	-.24033	.083591	.219	-.53331	.05264	
	Pep+NS	2.73067*	.083591	.000	2.43769	3.02364	
	Urea+NS	2.11733*	.083591	.000	1.82436	2.41031	
	YeEx+NS	1.70800*	.083591	.000	1.41502	2.00098	
YeEx+NCl	Pep	2.56233*	.083591	.000	2.26936	2.85531	
	Urea	.37067*	.083591	.003	.07769	.66364	
	YeEx	1.85133*	.083591	.000	1.55836	2.14431	
	NCl	2.32250*	.083591	.000	2.02952	2.61548	
	NS	3.16250*	.083591	.000	2.86952	3.45548	
	Pep+YeEx	3.78783*	.083591	.000	3.49486	4.08081	
	Urea+Pep	2.70817*	.083591	.000	2.41519	3.00114	
	YeEx+Urea	3.71983*	.083591	.000	3.42686	4.01281	
	Pep+NCl	2.07683*	.083591	.000	1.78386	2.36981	
	Urea+NCl	.24033	.083591	.219	-.05264	.53331	
	Pep+NS	2.97100*	.083591	.000	2.67802	3.26398	
	Urea+NS	2.35767*	.083591	.000	2.06469	2.65064	
	YeEx+NS	1.94833*	.083591	.000	1.65536	2.24131	
Pep+NS	Pep	-.40867*	.083591	.001	-.70164	-.11569	
	Urea	-2.60033*	.083591	.000	-2.89331	-2.30736	
	YeEx	-1.11967*	.083591	.000	-1.41264	-.82669	
	NCl	-.64850*	.083591	.000	-.94148	-.35552	
	NS	.19150	.083591	.568	-.10148	.48448	
	Pep+YeEx	.81683*	.083591	.000	.52386	1.10981	
	Urea+Pep	-.26283	.083591	.123	-.55581	.03014	
	YeEx+Urea	.74883*	.083591	.000	.45586	1.04181	
	Pep+NCl	-.89417*	.083591	.000	-1.18714	-.60119	
	Urea+NCl	-2.73067*	.083591	.000	-3.02364	-2.43769	
	YeEx+NCl	-2.97100*	.083591	.000	-3.26398	-2.67802	
	Urea+NS	-.61333*	.083591	.000	-.90631	-.32036	
	YeEx+NS	-1.02267*	.083591	.000	-1.31564	-.72969	
Urea+NS	Pep	.20467	.083591	.460	-.08831	.49764	
	Urea	-1.98700*	.083591	.000	-2.27998	-1.69402	
	YeEx	-.50633*	.083591	.000	-.79931	-.21336	
	NCl	-.03517	.083591	1.000	-.32814	.25781	
	NS	.80483*	.083591	.000	.51186	1.09781	
	Pep+YeEx	1.43017*	.083591	.000	1.13719	1.72314	
	Urea+Pep	.35050*	.083591	.007	.05752	.64348	
	YeEx+Urea	1.36217*	.083591	.000	1.06919	1.65514	
	Pep+NCl	-.28083	.083591	.073	-.57381	.01214	
	Urea+NCl	-2.11733*	.083591	.000	-2.41031	-1.82436	
	YeEx+NCl	-2.35767*	.083591	.000	-2.65064	-2.06469	
	Pep+NS	.61333*	.083591	.000	.32036	.90631	
	YeEx+NS	-.40933*	.083591	.001	-.70231	-.11636	
YeEx+NS	Pep	.61400*	.083591	.000	.32102	.90698	
	Urea	-1.57767*	.083591	.000	-1.87064	-1.28469	
	YeEx	-.09700	.083591	.996	-.38998	.19598	
	NCl	.37417*	.083591	.003	.08119	.66714	
	NS	1.21417*	.083591	.000	.92119	1.50714	
	Pep+YeEx	1.83950*	.083591	.000	1.54652	2.13248	
	Urea+Pep	.75983*	.083591	.000	.46686	1.05281	
	YeEx+Urea	1.77150*	.083591	.000	1.47852	2.06448	
	Pep+NCl	.12850	.083591	.954	-.16448	.42148	
	Urea+NCl	-1.70800*	.083591	.000	-2.00098	-1.41502	
	YeEx+NCl	-1.94833*	.083591	.000	-2.24131	-1.65536	
	Pep+NS	1.02267*	.083591	.000	.72969	1.31564	
	Urea+NS	.40933*	.083591	.001	.11636	.70231	

Based on observed means.
 The error term is Mean Square(Error) = .021.	
*. The mean difference is significant at the .05 level.	


Homogeneous Subsets


Bio	
Tukey HSDa,b  	
NitroS	N	Subset			
		1	2	3	4	5	6	7			
Pep+YeEx	6	1.42683									
YeEx+Urea	6	1.49483									
NS	6		2.05217								
Pep+NS	6		2.24367	2.24367							
Urea+Pep	6			2.50650	2.50650						
Pep	6				2.65233	2.65233					
Urea+NS	6					2.85700	2.85700				
NCl	6					2.89217	2.89217				
Pep+NCl	6						3.13783	3.13783			
YeEx+NS	6							3.26633			
YeEx	6							3.36333			
Urea	6										
Urea+NCl	6										
YeEx+NCl	6										
Sig.		1.000	.568	.123	.890	.222	.073	.307			


Profile Plots


:ÓÎHM6¡Zæó0?ó0?ËãÀ·oßÆUÌÏöùæonÜ¸«ÌæóùÁü`~0?ÌæóùÁü`~0?ùA>@llì éúñ¢¢¢ùóçæwêÔ©ÇÑÑqÙ²eUUU¸0?0Næ7mÚ´ÆÆFcÌO;>Î=;E::äæóÆÉü   ¡ÌÏt#>:íÕÙÙI"ËåRJpp0®Ìæù-_¾þAë¼«cSÒÒÒçÎËä¯­­		q 44´®®-ÖÞÞòk¿áÅB9q-`~0?`<Ì¯½½ÝÍÍÍÅÅ"#0?;;;ö½³³³v6*mJ¦<Âáp,YªT*q	`~0?`"ÉÉÉYµjóSëµö26l cÞíSJ`` tÐ&%2ù%É9s´ø­Y³Fû¥ ùÁü17?bÑ¢EÏËË3ÕüÄb1âââ¢"hÓÉÉIû IIIÁÁÁÙÝÁÁ!77×æ7ò;ù¬ýP(tss£ûlöìÙYYYF¦ÀüLóH$ÎÎÎr¹Ü$óÓn´e|Nç¨è¾¾¾yóæQ9sæàZÀüFÅ©S§bbb´SBCC)"N?kLù$æG;w67mÚdùiÈtòÓyçGÚÖ××³ùÉRCó3º¥è7N"ÝmÌBáîîndùíØ±# ÆÚü   fÄÆ ¶Ç|$`ÐÝaÂí0C:"""OW­ZEsçÎ­©©¡ÍÎÎÎõë×SÊìÙ³!U0¿C÷YQQN¢öï	&nLùåää(	bÌ¯±±qÚ´iC³6èîuuuìîìØÞö ÓP¤¤$HÌoÐÏ___ýtínöööF¦ µÀäiíeàóùC_rr2Ó!½oÐÝ«ªªBBBìÕÿÁ´Ë-_¾ÜÉÉÛÁår333q!`~#gË-ûöíÓO§Ì¯%úKq#S`~k~ô+¤¤¤D?=,,,>>"ô~ó°³··×yçêêjggçææVXXhdÌÀrÍoìùæóùÁü`~0?ÌæóùÁü`~0?Ì`m´½7K^g@`~01ð¶ç¾³D0uùÀÄP'zYVÝjj`|q;R@`~`M¾X¹zÃ?ÔÿÃ¢E$	ªÀüælÓüØ¸R©LNNCµÀü½½=ªÀüæ&OÕß·>ü=QbÀüJåÔ©Sx@@Ãár¹gó'&&:88Ì?íÂ0?L0Þ~Ò¾×]ñÿ8iÇ±bÌÒb3¼ìVJºúG¤²þ¡Ì´/66vÍ5ÌfDDDcc#EHõx<Å3!!2àºÀü`~0|µÿæÚ­×vñ®MOºÿÁ')Ow¢ZÀu-Hæd2ó³³3Ëå®®®lþ¦¦&ÍoÂÑÑóùÀqóNãòÏ3×~Lk¯ ½fÃ®|Ô0`~ÆH¡~~ùÁü`Âà*.e=¢È.~qðßRÔ¿|#0¿A_éQþÎNÍ[dBáââùÁü`"ÍïÇ§/V¾÷qc~=ò>ùEDDÅb???6ÿúõë)ùÁü`ÂÈ.½÷ñU²*eZÉ·¼#0?©TÊår9WMMÏçÛÛÛ3/ÿÌæ¡7E|²âË«oøéëý!w+^ÞØ"Cåq0EóùÀxÐ#ï>r1/íþÃ´ß°bcæïþr!>é~G×kÔùÌ`#í-Lßq¬üMÄLÎ`À0?w^ÈÔ"é¸>%jX ýý*2¼Õ¯=ÕùæùÁülUj¹j[úxõËT;°4%¯Ön½¶aW~ÛK¹þ§0?Ìæg+t+4*fb`|q;jRZEöMQÈêËôþþÁoNæóÔ¨òjU®£ÕQßÀÔkWGå|¸îÊK´Óuiß;K2¨sÌÀüxÛsÉáÆ3 Î'×C]o¬.FïÌæÀÓß¯â*óÒnÞiDmùÁüÌØ,-²-9÷HÚºQ¾fiïç£ÏZa~æó³iªjÕ,ÕötÕÉBuÃT	°"Òsë?ø$åRÖ#TÅ$§á_?|<#hô¡þ_B4?ÄÅÅyxxP¼½½=  Ãáp¹3B¡··7%º¸¸=×æóð3Y©:qSuá®*«JóÎïÁsÕþÈ°dÝ½[Þßýô9VGcöP7h~@.×LÑØ¨éiÈãñ¹¹¹INNvttDeÂü`~À¸'V.R+U?·ö?Ó¼ùÀ²)­lY¸öJ|ÒEo?j§ùÙÙÙ±)oæ±wvvf"$®®®Æ`~0?0¨²ªHþ4*U»®2ó©v^EÍÌ±üóÌ²êVÔk´7IõØm¢«R©4:::,,ÌÓÓæóùË  NÈü®×¨v_UíÍV]¼«iðÀ"asì8V,ëîEmq    ¦¦Ý¬ªªZ°`¾ùÚëëë*`~0?°:4÷²çMkoRuºHµ3Suá.êEznýÂµWòÅ¨0nêºQ¼¾¾Þßß?++Kßü"""ÄbÍ)üüüDÚÚZBÁãñ`~0?°$ª³·U³T?¨¨e¯5½ýNÜT/V? ÀÃæØ°+ó¶ñ'))iúôéþÆÇÇÿü¼×9©TÊår)û055ÕÓÓÓÑÑ166æóùK¢©Cc~»®ªKEj¹V#ùßáª3·Õcq^0QU·.æ¥]È¬j^ùÁüiÉY$%ùSËWW7©û¨+0¾X¹üóÌgèùÁüÀøÃcfÚ¿×ÔåÏðþUuÒðÍÙ»OôÈûPÌ¯ù14wªïiúæÕâý;é5K"Ó±/æój~m¯T©åÿË| ùèèz½qOÁ]ùÍW¨Ìæãa³FãLÛÒ5Á¤]2¨NÝRíºªºi`î=_¸ö ½90?0UNµêÐuÃî,UtÆHv<xM^©ùù@ÝåSÁé÷í>Q²òË«Ì,¡PÈnRRIÀ` a~0?`sô)ÕEõü¡þL¥ªNº$2ý`Â]æFEEÅ¼yóØM6?TÌæ&ò^Í¼ÐÌ")êK¿*>éþÂµW0X,!!!Ìº¹¹¹F`~0?t(UêRêHêtf@L@ÒÖÍÛ»aW~GÆ	ÑQ~©"yôÊÑ)¹ªªÊÛÛ">>>Úkø¶··p8.KñaÍ²1¹hÑ"ßX!Ë/_noo?cÆíB¡Ýµ³gÏf~ÐóÆò¨U³Ü±|Lô¹õèORè/ª¨Ìo6¥o rô_¶lYxxøÒ¥Kµ#""5ïªy<k~Cõó«¨¨ðóóS*dÌæ7&lÙ²%::n5Ò>íBCCO:Eæ÷1)0?`Ì oÀÿCÛKù§ßÝßÁÀZ¨¯¯'«««ÓNtvvfß°¸ºº²æg rÇ¨¨(T)Ìo=veeå Ñ]KFHBáîîndùíÜ¹3áþñbzÏÎôÚéÙWPÃ'Rÿ°*é³íR¯¤£6Y§§»Òi¿Õ³³³3ÆüJJJ8"l	æ7ÐM¶oß>ÒÒRtâÆ¤è_vvök£÷¤ïÒ]å¬¾ëÕ¯;d¨IEw·üÈùþ×FÔ0#e~ÆdÓÇãÍ7oçÎ°%ßB?Dø|¾Z«ªöGlÜÞÞÞÈ´öQ·ö,²7[³NuoÎÞ[y[µ>Ýõ.""B,SD øùùk~µµµ¾¾¾ÂËËýü`~cËP/íè#º1)0?`NÿÛyþb	ÛæBfmàªd¬ÌlÌü¤R)Ë¥+;æ×À   ÂÂBdee£Ja~cý(9wîú£´?§ý52æÌ¼W³ÜÞløM"ëîÝrðVøæìõm¨Ìo<hoo§ßô£ÄÛÛ[ûG	ýÍËËsuuµ³³sssc~ócèç1´ÍPtïyÈêËGÎ+zûQ-óæõ?ÍÐXÄúéïWí>Q²$2½¬ºµùÁü¥J]þìÿ=`@«Ì±ãX1s`~0?Lñ¿#yÚº¤×¬¾ï9ªóù`:XÄJh)_»õZøæì:ÑKÔæóÀþWøXÓX×.{90?fõ¿äo¦ÿY=ò¾ÇóÒ0ØæCÊÄÄD6^[[ËåríííÝÜÜbbbÍ`~F8uêTõÀÌàG.=¤`	áaÛòÏ31Ø¶ùùøøh¯´ËZ]SSGRRR©Å~~~qqq0?ßHX²d	³ü3ÝLÚ/[¶æÀ/üKLgSª>ø$%û¦UlÛüø|~TT¾ùñx¼=ö°ésæÌùÁüFÂ´iÓè¦)**ªªª¢§§'ý ¥ÃüøXd"´uGlÉY»õZcµ,×ç·¾NØdp~«ùÑ____v¥]ÖêfÎ)òEi¿0fÞ¼y¡_L¢ÌCþ÷c©º©õ1¦dæ7|ðIÊÙ*,Â,¾;½E)£T¾ÃéXÃ1ðÇùÀÔ©Sé¦ioog^þÑï¼ó`XÿS>V¼¦ú^¨·a³Ó#ï>r1/­ª+¬IóPÿÃùÿÁüÀ_`` Û·ÏÕÕUý·èç?üê'ªC×U§ÔQæâaÛÈôÇÛ^bTæ'µS¼¼¼êëëÙJ¥2%%æó!>>>vvv¤ííí´Iqü¶0?`¡°KÀËW?x÷£$>éþÂµWòÅ¨0iÍâóùlJdd¤öÌÌLzvÃü`~6ÌX:U'nªä©KEð¿ðôyçÚ­×6ìÊÇ«>óËåÞÞÞlH$rssKKKSì1cFrr2ÌæóÀ`Ù£6¨yÎX2óBV_¤×`0ù1$&&j§Bãîî~ôèÑ¡ö0¿áñõõ¥;iÊ/ÁØ^Fë?jäÕÂÿÓ#ïÛrðÖòÏ3±/¹ù1¹haD0?¥¹SR¦Ú¥ñ?ÙkÔ>UuÒÅ¼´'ï`eócÞöÅÇÇ÷õYú»0?`­´½R¥Uhü/«þÇ¢èíç*BV_Æ`?ó³···.0?`ÝÈ^«rª5þV¡níäñôy'o.sÀxß3Èü:;;a~Ý7K«nîuß¸*9`ÌY¥7  Àòåæly¯ZØ ÿñCZ4Ö¨u÷~µÿæÈtæ1¿)C±½9Jõ=±fè3·Õ%6?`YuëÂµWö¼ÓÑÎ0Aæg7ÛÀ8Á,r,_uâ¦­.¢èígVæ(­lÁ`Èú):)LD&ñx<gggz:xxlÛ¶Õó³`~ÀÆyÔª:Y¨:|DÐü¯±E¾9û«ý71aÍÏÇÇG.ëß%Kø|>3B¡øbÔÌÏl´··Ãü%@ÈÿJEîV3#õÚcæÀó#½Ò7?8Læääùwww¬áÅùßùbÍà¼Z+õ¿¶ò»òW~yµáY®'°1Zþ½ùñ¿!ÔÿoóS,¬ÕØØ¨c~ÑÑÑ¨y`óóòòÒÞáììL¿'`~L´=½R¥[£ÿ19&ÜUôöã2Û£¿¯µ¯÷éèCßsó+((Ó1¿ÒÒRwwwZRR«ó!ÌLÎÄÕÕ"$|ÌÑ«V­ù`YþQIq?YR½#çËIûHþpé0í¡þÏ#ó#ÿÓNaâp8çÎCÁüFx17©EêëëJ%E¦NóÀ²üïzÆÿRË-Öÿu,ÿ<skls0óÅ¾¾¾úæÇRYYéîîùGGGº±òòòjjj(²iÓ&&Y]°Dä½o!ÿk´¬ÉO_¬|ÅÅÌüFi~DTTÏgS¦M¦T*µ3[à`æÉçÐîêçççóÀ¢ýïà5ÍÐ°HÛKù§ßÝàmÏmláâ`óËåÞÞÞlJxxø¾ûÄb1óQLLÌºuëPc0¿B?,Áá!¤»Í¿-Ì_ÀLÍ,2qþÇÌÛrúb%æmÀæG0Ýî¸D"	:u*=£¿øâBùÙ80?ùßÉÂqZÑÛ¿ãXñGýtÿáùÁüGÿðüÍÐãâUuÒågùõÈûPý`MæWQQ1kÖ,æ2mº¸¸V	»ù_rrúbå¤`0Xùeddè¬ÛÁÄcbb`~X·ÿíÎ2ûÐÍWÌ`ÌÛVi~ÌÎ¬ùR|Ú´i0?¬2ó.Á`õæÇÎäÌ33æóÆÐrãÅ7üñý/»Pí¦a%@dÝ½Ì`,ÂÖm~...äyÌ>2?B±~ýz[àäà0?¤õ³gj¦øPÁúaM°ú»H«iöÝ²ú2s-_NNÎÁ`Vù±àÅ7|r8ÔÃÀ.|OÝÜiÌ§/V.{¥¸¼	À?ÔµºÝ;88,Z´H"«p¡PÈnRR9mplÁü¦¦&ooofl/Ýj³fÍª««³ÀoóùóÐ§TÔi!ÿk²õöéóÎµ[¯a0ãi~lT&''«ðyóæ±/++3òd­µóùs¢T©KEªC×UçõÎÌoøàó©ÕÌ>Í¯ôÈFÉ»Ë½½½Y<!$$$++"¹¹¹7uwóùMð°Yu$Ouº"¤m/å[c0ükþå×/>üK^ÙR*S§NeâíííËåRÍèàà0þ|ývau«ªªbVgõññ©©©aÓ*|¨¯OÙhîë0¿)ÃÁóù± 5rÛûJY7ªÂR`9YØ(w÷§©ûNÞÁ`&à¡þËÖÞØØØ5kÖ0êÅ|y<Å3!!2k~Ä²eËÂÃÃ.]ª8TáCõó«¨¨ðóó£ãA2;k2?»!À¬.`,PÔ<yòë9ÿöá?Ï­ýooK·Ç£N,þ~ÕéÑëÓ:äÛpAÍ	L&c>rvvf"r¹ÜÕÕÍßÔ¤¥P(56¢¾¾6uzóU¸S%wÂU³>óóðð8uêT*µüoó³z·xÙUçÐûã¯=ÔûäyÃ¿~ØùjfÂyú¼sùç?æhîTýXªñ¿'j^þ0~æg²rÚùõß×U~ú°ëSRRBG$SÄU³óH$>ºöÏËÕó³Èùk~ ²ýüºoü¡f&f0GêµÇº9:TÉ÷T®«êÍ»ÀTóc_ééäïìÔLÏ¤P(\~CnàTy<Þ¼yóvîÜ«f5æÇBÂ^TTäééIÏZàÌ3ÌÖ¿ïë8"Í®ù'_Ñ[+~NéöxJy«çöýÞ'ÏUrêj|h)ß°+Á²×ª²±X`¼lEDDÅbÐÓÙÏÏÍ¿~ýz$$$DFFØü*|¨©­­õõõ%ÝôòòB??ë3¿_üôÏÌtrrÍ¦¦¦¡¦|nnngöìÙÌ¨rcR`~6åÎ¶~vP=ðÎ¯Îý÷=7ËÚöô/lmZ%~Ý_ôxFÐÃò­sýÝ_ýùéüðçK¿¦]Úcìü>ãUN±¢æI_³5i9ø£s¼ìQåT«öç?4Àæ'J¹=I¶Øa¹ÏçÛÛÛ3/ÿFv¡ª¿`PPPaa!EèIMÆ³2óËå¹¹¹ÚÖÃÃ#11q¥ÑÏåËúQhhè©S§(Ã7&ægK·Õþ··»o2­½JY7Ùi~Î¾g­ò²ZR=>òÅµ»I"Xÿ/¡[3ÅÔPä·üÙþNÊ(Ù|¤íÀyÊI%+êÄxe8üèíßwòÎÂµWJ+[LÛ³[¡ÊªRí¼ªú¡D@å"ùé¢ßÏÏÙÙ9,,lýüHûýÊW*êîÌ¢ÀÆ¤èß®]»25sc×Jçw+]ß«rK;ïý53=cåd'^È=p<Ëþ~Ù%¬¹³`eÙ¿-¬tû]õÿù÷ÿ¿÷i³ôå%Eù²åm¡]hÇI~	Î	Ò®¾ð·MÉ/ý4²²ÓªIøéõwé/f'ý»L*`~ÀZÍO§K¹ÆöRi,àp8ÞÞÞUUUÚi;bâÆ¤èßÕ«WÓÝÞñléæGÿgYÍØo¿zëÅ4É¡>;ðì£¯¼·ºÁwÙ£ÿñ^ÍúûxÖ(ÒéÓæmÇ)'å§½dOm¸òSs®I¥¿f(«£«÷Ö#elnÿÉBÅý§=¯ºqoÉEIÀhVø×üÆh&çÆÆF///ííÕ´öÚ$¾G_³TQóäUæ­ÓW¤;NK6Æ²­Éu®¿øÏÿN§'ò[.~]óß¾£³yôbçYV=¥GÞ÷Õþ[rÌ¼2»Üñõç¸·ÀBÍÏn8F?³º¹¸¸(µÖàscR`~0¿ñÜ<ÔÎüÔåÿ9¢fJØô¹#;6Ù2 7ôÙOîáÊZU'14X¨ù3gÎdz666.X°@û£°°°øxÍô744ÔÈÌÏ2ÕPQó¤ûFiçY¤ìØdRÃ:×ß1PH)E3ec,©!åÔ@±ÉñI÷®½RVÝ:XþÅü©9Î[o½ÅÎñÃtAÍËËsuuµ³³ssscFóùY#ÒvëµÇeÝã;Hª:s[uð¦À´ú`«kÑØ]-H$¨Åó³@HkHhL5S|(`G60ßø´&gß¬¾ß0aß³ñ¥*µ7þW*ÉÉÉaaa¨ÌÏË)MË£Æ3ô¿°ýiäFÙÜöR¾5¶hÃ®üÆÙÄ¶W?û_'ø`bÌO§ýb1LæóÀr1Ðî¹lqÐÑã~ëëýþbAcÿÛyþRWX=/ºÕ­¯Ì¨ãÌ"qqqoooàp8âL¡PèííM...gÏ5ÉüJåÔ©Sø SþÄÄDùóç£]æóÀ"èïWL¸ûÑg?¤:6YÞ«Ê«Õ¼ÿÿkçq»úQÂãvãÍO ÈåçLOz²1ÇdðôôÌÍÍ¥Hrr²£££ñæGÚ»fÍfsÐÂ)ÿ+(gBBeÀõ·Jóëùü`~'Ow®üòjôÛ]¯Í<ÁcYÿ;_¬afû,¦5®Ôë]½ùÇ$»ºº(g¨µwµ7Hæd2Â)[SSz`Î5­Öa~ã0Ìñ!õÚãk¯dß«Àñ,ïÕLøêÌm+ò?ªúî¦fHÓv´í!ð@íMR=6E[M#J£££ÃÂÂ<==í/h ûà kç·@=ùÙ,0?t´uoØO"ãvPsMVªÔåÏÞøßcåOÈØ°©¿ìH7ù¤"   ¦¦Ý¬ªªbgÆÕv¯AßºùúúFEE¥¦¦D¢Ñß SþÎÎNõë,[0¿öööððpLieËÈôôÜúþ~Ë&Ó[zøý¯*÷g+óklohÀ¢¢"R=R7×××ûûûgeeé»ZDDX,¦@ ðóócjkkÉÌx<ÞhÌoÐÂ)ÿúõë)+eõæçîîÎápÐÏkAÖÝËæ§9ÌÍP­ÉMï¬éþÿ:òûÖ÷>ýû_lfÝd0¤¤¤éÓ§ÓCþ2Zé»T*år¹ÇËËGêéééèè;ì2Z8åçóùöööÁÁÁÌË?`ÅæGV¿û§³³3ÛÙæEQ'z¹$2ßÉ;c¸ï¢zÔ¢äç)÷f)N]ï8bë&ÃüÕÛÆ)¨Û1?Rxº¢ÄÕÕ"$|YµjÌ¢¿_u!³v1/ízÑSÛÿ¶"©*ùÞ KX×ºÉ0?óe~ìoR=Ô××+J°:Âü°%¯Ön½öÕþã9câÑ ²n2CÃ¿ÕNÔÿ²w2°F°ÂM££#y^^^^MME6mÚÄD0«C^±øORÎ¦TYÚ`qõ?3-bÌØd¾µ²qáç-kwQ²7YZüNíhÒ±hwÒÍÆÏêþÇï/ýµ§Öm~ìxí®~ìH¼oßÉ;K"Óï?|1ÙëúÂ]õ1ìüFou]¸Öû#Óüä×Õ¹<ü'ß:×ß5üëbÿ¹7oO¤°j¨êëgÊé¾QJª×ÿâ%ÛÚÛúÙARLÜÕ4?"**ÊÉÉ"!ôöö¶Àoó²½k¯9_n«9Fî»³TÉ÷Ô­ÓxÚ×,Õ¾Ê)îü>cP5|äü[RÃºÿù'¾Ë$cÅï­©û¹´cÿË.ú×0Áæg-ÀüÀ¤B^³$2½¸¼	U1¨ÿ½YäÇRQÃçamî8-ò[þÈÙÒUýþû|ÌæÀÏ4<ëßÍÛ;¹s¥J]òDuðêÄMõ#Ú°óûr>²=¶µ÷åÑ[+qÝÀPøûûBvâbHX×dnæg`kæçëë°R¯=|úbå$Ì1úêÍ$ÿ,±ó"çc´Fîü?Hûý÷ù=·ïã¡¨¨¨7o»Iñ²²2m2óTæG7ð1`l/ãL¼oÃ®ü>û©Nôµ1µ¾ñ¿Ï-äú_v5-ªý¿æÕüÿ,Ú%$$Y±-77â¦ÛÈTæ7¹ÌyÛß×gé]Èa~À).oZ¸öÊî%Ìaÿ;_¬:|COl!Kc&gÛ£ôàë;ûÍ¨«ªªA>>>ìâiD@@=²¹Åõ3ÊVTTÄä¢EFl~óððêB!0%º¸¸=÷³U|[°IúûU|AEÈêËbeq£¹Suá®êÐuuác%@`~`ôóúd÷>P9ú/[¶,<<|éÒ¥ÚILLäñx¬ÕÏ¯¢¢ÂÏÏO©TA2;Øüê<==I.)ìèèÃÌoÆt]­bf°A?ùÇÊ]¯Qcå³ÕujÙÕ0ÌD==ëêê´I««ëPÆ¦¹c@@@TT¶Ïjúå°ýé#ÒGç`Ø %X,¦KE7åËÌØÌ`úÁcîªý9ªÌÆ/óùtì%þLÃ¦URRÂáp×uËwppÐÞ$ÕcS´szR©4:::,,ÌÓÓægæ7e0¶1TÉ«O¿»ÁãìµêÆCÿ¥U¨_ö¬¾fiïç¦¦åQ5S|F°£©Å6¿ARÇ7oÞÎ;Ý1  @»OaUUÕôsz¾¾¾QQQ©©©"ægæg7ÛÀQôÁ')Xcb`¹pW-n3uï§óÃÉáÆ3àÁü"""Äb1E»¼ªÓª­­%'S(^^^Ãöó+**"Õ#uS´5ûûû3ãurzt,:&ÌÏ:ÌÏùkGÑÛöÞb^ZUµ1±þ§YÄÿÙ;¿ÑT*år¹L?g`GPPPaa!EÈá=bRRÒôéÓ©|ú?èz©©©±±±0?Ì©½yæWûobeKA©R?ÓøßÉBu5VÉØ´ù±=ùÐÏq õÚã>I¹õ9,×ÿ¨ï7jVÛ3?¶'úù0¦09xÛs1ÃÒýïQ«êÌmÕÁkêRZ.Û2?kæ¬¼bqàªäó©ÕxÕg5¤ªJ4þWøþùÁü0yßî%K"Óï?|Ú°>:4S@ïÏQÕOàÐ`~óª³öÜß0?äaÛòÏ3·Æaeë¦íÆÿvg©rªá1?¦Wþð6Î®ÍóÀÎ¦T-{åzÑS´ðÚrÍ äÔÙÕªÔòñ¨~Í¯¯¯oêÔ©AAAÌêmô×ßßöìÙêÙÈüØuú`~Ì±qOAÛK9jÃÖXäÁ·üê½§ª¯>þñAµ-I»°?¹êZï_-Z$HÌXxbb¢N¯­­år¹ööönnn111æ6b~³fÍ¢ëªP(ØîînJY°`%2Í¾0?%¯XüÁ')§/VâU³#sûÇÿôñÎÀ÷|*ìãsK	±Ñëm?kdf:læÇÆJerrrXX÷ññÑ^Æ=GRRT,ûùùÅÅÅÁülÖü?acîÃÒ#ïÛrðÖGý9&>uQ½f	àïêÇÃ¿)>ûó·çQmÀ$óc°··7Wi´Éçóµ»ï³x<Þ=ØôÊÊÊ9sæÀülÖü]éºÉd2Ú¤¿gyÐÚaÖ·-Lßwòá¤þwèºfè0¿IÅÆÒÅëï>|~g¡WS*S§NeâíííËåRÍèàà0þ|ývaó£¿¾¾¾ì2¾l3g2ö#£ÀêÍ/##cÐ±½YYYLdÕªU0?ôéïW¾X¹v½è)jcò¢T©KEo)¦ÙùM:Òò¦Ñ¶×­Cii_llì5kÍÆØHõØÅ3!!2c~l2Á@ãÌÏÍÅ³fÍ¢LRSs½éå|[°Ú^ÊyÛs·¼y[ÀªTGòT®«ï±«â#ñÍÑçÕ"tÃ=Ôµprr"cã¶ýM.»ºº²ù4KN+GGGýBX´Ìüæ7©ÍÏZù!3¿a1/þ¢*.%ª?/r¿QµóêÍ½¶ë=£ú±ëÃægÚÙÙéç×··Aßù©^÷øúúj§xyyÕ××³9JeJJÌÏfÍÏÝÝÝÃÃ£²²æÀ°Èºw+æmÏml¡6À¤$yª½ÙïÒ>ÞûÁÙ½_5nÿþ­ý~õÊì*Tù±¯ôtò33²)#ÍâóùlJdd¤öÌÌLÍýJ°ëóKYukÈêË·I×á©o7äîÚWÃo>ßÞÓþ÷øSQ3`æÁtÄ~~~lþõë×S$!!ìÍxóËåÞÞÞlH$rssKKKSì1cFrr2ÌÏfÍÉ±yóæööv¥Ò¢!`~`¢`s,ÿ<ðã©<tåìaõYñ¾£ÍÛÏiR*_o¿#0?©TÊår9WMMÏçÛÛÛ3/ÿ/<11Q;E(úøøPùîîîG5æµßPëö²Ý`~`óôy'9ßcÅ·Dö÷üGÇ/ÆÇïÜð<úMHú(ú$j)ßØåLàóHf~ÃÈtÌÛL¢±£ñLIBè?4o?ñÄlþá¦oÏ«_È^Çdíá¡~ÌL¤ùY0?0´½oØÏÛEx´÷´_(OúÏ³öÝÿëMé¿¿svÙÞzöeïmÞ~N¹3óLì7§ñ¨(`.F³Âùýòÿ¯ööððp´U·.{9Âw¥2õãsKöþjíÅÕUé]ò7=®Ü"üa÷çÛ¿gßÜ9ßö+ûQc	6?wwwf/úùSÚêï>ÏÐ§ÛÎvÞþ'ï,ÿ<³áY®/09ÍÕõ#ßûWÁJmáÓ¦[ÑsüpSô¹§í"T`âÍÏËËKx³³3;oø`VÓInnn¤³gÏÎÊÊ22ægá$mËúâ7Æ3´7uÝ×Á`0,¯û^ß¨Ë]qµïþ_¯H¸Hngx¬Þ° ó³··'KH$®®®!ácFzx¹Þ¾¾¾9sæè_hhè©S§(³hÑ"#S`~Î«=¤b¦ÆG°#eÿXÍ=t>µzIdzqy.+TønÖìÈùvîßùðREòWÆNñóXù±+úêQ¤¾¾^©TRdêÔ©#+pÛ¶m;wîÔ7?gggf¾@BáîîndùíÞ½û*°~m<÷÷°åOrJæÇë/~y"¸:@ãÉÇW_á»Û'86è33M-!ó`Ìóès¨IÛ`lêzÎ1½KÅ&&&u ÚÚZ.kooïææ3Ö'ÆÛüéZæååÕÔÔPdÓ¦MLdd³ºD"oooÆu>Ò.¢c~ô¯®X??)Øo)'sãÑÿuåÂOÕ]]¯piKèÞæ´¯Þ9ôvèÉ?(<þðy%ö%ÝQÆ5¨¶¥SÁp!,1?¹ ¦¦&¤¤$zÅb??¿¸¸8M_dd$;C»«»8Içææzha¢Ö^$çÄíøùl½ý;ôÙOÌXÊËbòr¾r2øLIBKW³ö§?^8tâè×¦Mß.¥0)àX&¶îfÉ«Ñ*g|ÌO%7>¥Çãi¯á[YY9gÎMz`åf'''RôËÛÛ4mÇ,Ú¹¸¸(µÖÂÒÆ¤Àü`~cDYuëÈô'ï`0 ê¥õûoìõ7ìÌ5v4íRE2:Æ2¡_yi£TæG¸¸8õÀÌkËåRÉ éÙMôô<öì°æGu2Ì9S$£ÀÍoLÎLïþ×LaJCCCLùÙÞC[ß;ºñßÆ­ÔLÌÚ§/V.{¥´²WÂGvÈÂñ"þPÂÀD=:µÍO 0í³±%&&òxoVñôôdÚÜ1¿zìêd0Ð×æó3ööeâyyy®®®vvvnnnF¦Àül>E_ìòÄs_¦§ÌßùÇåÙ£|åïéóÎµ[¯mØ9 |ðQ6Q'À2ÍíE1# Õ£! =43e0´3ùÿÁü&ùMÌäÌÎ½Ì£á?*ûlkoiz¥Û	dæ7|ðIýÅµ4v4/âùÂ,íMR=6EçþÃZ*FGGÉyzzÛ_ÝÅ¾¾¾Ú)^^^õõõÚçó³)ó³íù¤È+I­¤ÈåÝ¹[Þ#ì¾úzþáq8tÛKùÖØ"æ´t53Âç÷îþËËP'À2	¨©©a7«ªª,X o]ú¹êNQQQ©©©"ÈxóSôõçóùlJdd¤öÌÌLMÇ©S§è·å[µsýT1Í%qÛ¸ÇR÷æ~tbÓÄVßlèéz=FÇ-«nY/¨À`É&|[áÖBQQ©3À¢¾¾Þßß]ÎJÛº"""Äb1E;Cmm­B¡àñx&÷ööfSèènnniiiê½3fÌHNNùÙùI$L>U@''§ºº:#U·DÿþxS'ÛÚi×õó_ýT^Å¸ðõüÃôâfMÑÛOÂÁVø¢2¿)~ZÜ¯ìGµk!))Íþ2Cõ­K*r¹ãååÅ¾#LMMõôôtttVÑt20kw±B¡ÐÇÇÊwww?zôèP+3?mÙ§t»ØÙÙ±8sæLú%ófçæ÷Hþ¯¹¸,aýåÎ±¯ú=½u%O³Ý]¸÷Ã3¿Í¹YóêeÏÅ,Â»5¶9&]òÎKÉ¬ð`~Æéää`LyVÝBæ·eA0¹b¨^J¯¾Ùpi×õýKÎ¦îÍ­½ýÄ¤æàÔk1cR	ßÛ1s7¥o¼Y_áÀü!Ësss¡@ÚÿtVôù3bÒLÎ]ÒWäLspÜJí[÷YbÈ¶îO¿»±%9l×¯3ªÒµRP-ßè÷ósvvC??`iæÇB¶GÎuìÖÑð·qX^¥ÓÝúÁ')§/Vö÷«PÏ¶*|ë/Gúîÿ5ýð`~FïùË.Û,ßü´a:f*]È4×Üj8ûãýk¯ü¡m~e?I^Tæ7oÇÌ%á#ùð`~#4?Ìä¬Ñü´yõ²ç§ã·?ýÃób|tnØæ``EÂw£.÷ë¯æøÍýó¥äövTæ7ì39cx÷LËÔðÅoPÁôÇydß-æ¥eæ7(ûO+(?âBÿú+L®0ã1`Ü(~Zµ¾åI>0Ið÷÷ì&Å)Å±8É21eNç~írjkk¹½½½[LLÌ y5ó³@2ìüãISc~#ØBG«=º¬»wËÁ[á³>×Õ;¦Sà¥]×½¶ew¦@`vúý$|;r¾;/ìÌ§ñ>0Ù¨¨¨7o»Iñ²²2ÃÆf2ÅÇÇG.ëÜÔÔäáá¤T*Åb±_\Ìæó@YuëòÏ3&ÜUô3	iÉ)àÞÏ 9ØÒ¨lªÊüoÑé?/â×´T£NÀ¤%$$Y·#77âÃÛUOgÏçGEEégàñxÚ¹UVVÎ3æóùq¥¿_öÞGýTUgòÈ$¨M»9âO+P¥%|ûoìä¿ÿÁÀSÂøzi=êX÷ÕåÏÌ¨_RUUåííMí5|ÛÛÛ8Ë¥ø°îEÙhÑ°æ§Xù·±±Q'ÃÌ3ÕäÌèæó&Ð,yÅÛ»ãXñ(áeÉüâVÐ<Ô´Tï¾¾+äd09ßo­KøjòbÊ/67e¢Ê¨T¥!dTê¾lÙ²ððð¥Kj'FDD0ZÈãñX÷ª_EER©$dÎ°ùéd0Ðéæóù1çRÖ£k¯dß·Xíæ`Æko?As°yyÚ.:èÜ£ïð=<²ÆoüÃRãÞ65~÷ìHwÎ$¤¾¾¤Jgz]ggg&"Ë]]]q/rÇ6ÜAM-Ìüæóù§£ëõÖØ¢µ[¯5¶ÈÆô@ä¥°ÍÁè8;ñCNûìýUTæ7EOnMÂÕÕ®§­ËÛÿ.n`ÂÓ]OªvÍ°·iÛª|vS,ûúúj§xyy²9JeJJÌÏ*Íóù+¢¸¼iáÚ+|AÅ°9ÌÛÌt$Ds°ñ´t5Æòßà7_g|u÷YédæFo~ÆdÓÇãÍ7oçÎÆÅçóÙÈÈHí>>>0?«4?í©û5?Ìç,R½	wW~yµNôrO,°ööíæ`Xà 0oøV$.÷Ùû+Þ¥uY5W'á>óÅ~~~ÃºWmm­¯¯¯B¡ðòò2²÷ööfSD"[ZZz``ï3a~Vi~,tñ,YÒÙ©yÑß   JÉÍÍùa9r¾|1/ÍÔðÎa³ýiMêo%¬¼øá]a%mÝûÙNß[)@s°zàà^bØµÛkýåÈ+©æÌk~R©Ëår829vÌ¯ô(/,,¤HVVVpp°IKLLÔN>>>thww÷£G8I`æçììL¯¯ïççýD ¶)ÌàþÃù¦Fþçùú@áþzéàéRôQé5#Ê~¥vsð$#¾Ï-õÙû«¿Vb9ÝÁïEOö¿äí¯èhFm&Þüd:æ~~`ì8±äo¨O;º^oØÿéw7Ú^Ê­åMªæà.y§¶ð]ªH¦ÜÕ"yW¼÷·Ï»±oÞí=®Bó7`bÍÏÅÅ</44´»[Ó&É)Òa~`üÍ/3¿!dõåó©Õã9Ã¼°ÍÁQþGÈ/íºnÍÁ¤w$y¤z>#Iw¿þFDÖ¿åí7_$ø)n~yfj0æ;hGÒÒROócs4<ë°¯©3:ØJçyÝ÷:£*¾Ï-ÜKléB«¥Qå|)ñýª~¶_AU|þAÔ`"ÍO=0iäÌ3ììì¦N:gÎü¶0?6?ãáµ^d¿z;6qóOÞÌßúË¿ÚívæC©t÷ÉÒXzqIá÷Ë.wcß;HX¼óT`ÍÏZùÙëÛ"£sV&§^Ì×8ZM"XZÙ2Iji&ù#d;v´Ê,áÜtïx¿±£7í°× «J²Ó9UuìúG§Ïÿ6ëÐ;øóÒ¾yýóä2ïüëó¯Â _Ìæ7i ÉYÌïOR6ìÊ_¶á§-Ù±¢ÁfD»9øëù'°9ïfÁ¦ô¾ûá3@¿ª¿Eþ¬¬­0ë¹àûÇwW®[+|ÿo·Þ=»8õÒ¾³ïýñK_; v¿®UX°o~ýí¦µW.zëÐû)×Ö¡l~³fÍ:u*3×ÅÅE ÀüÙ)«n%ák¼bZ³oÞ_qñ¯_eõ÷«P9dåÙæ`²ÀõoþpO"j7áä¿¸àP½´×bPÉÛûàïK¯(üõwB(N)9u'ËÕ^ÚT~àê+oðÉöíB®VÈÛ?ÿ2ÿÝ¼ïw¿ûýåÝ2Ô-ò¡®µÃ¢E$OLLÔIa"2Çã9;;s8mÛ¶áZØ²ù139k¯ØÆÄcbb`~À¼Ä'Ý?REî_.oÎ¾_#!DÍèÐÑ*»T¸ù'RÀïN¾É,M¯ê¾2Ó(ûGe~ácy!oºß~;·ùRÒ#ä­º5ïó;T­?_ §é)`SHÑ×óª±êy~<©^ELHý¥-ÒLE¡gsMÇÝ$AÈýïä/ö*¸½a9cãJ¥2999,,Ìûøøh/ãËnÉ%|>ÙW¡P|1.Í««+]ûÖü)>mÚ40/|AEz®Æ0bÏÞük²¢·¿¿_¸*53Ê~åóZÉ3%LsðÞÏ¤îÍ­+yJé#+°¼±o~ì<ÿ¸w÷ßØ;9¯C!%c$ïÈÃ¯ÉcÞä%ÔíJv$¯_õó`#¹ôéòôË[5¯÷N?/8-vßøù°ù1ØÛÛ«4Ú$½ÒÏ ³^«L&srrÂå°YócaÍ~g`Ý^0dß­ÝzlÛß@)¨#-°úfCÖ±[ûeóÏ)|äydðÑæä¼Ç]7[Òµ%ï¿wÜ_ÍHPM7è«¸¾¶ªëW¢Æ´Uf÷¾Iû;ÌÏö?É7d!<É2àjô8:u*ooo G3Ë¥8?11ÑÁÁaþüùúíÂ.ÎëëëË.ãËfðôôÆe,æÇÌäÌ¼ç#óS(ë×¯§¸»»;Ìr>ò¼-oÅÌ/¯X²úrU5c*=]¯Ë³^ü6gïg¢<i[VIj¥~sp½´þpÁ¡@þûócçEe~cÛÂ§#yÛÊW1G¶¼´®§eÏóãkÎDTÄ4:bÛùÙ6/ÛjÚ^< rr5Ò¾ØØØ5kÖ0±êñx<6ÿ+(gBBe0ÆüØd6Cii©=÷©äÔÔÔ[6¿Agr¦æGÚ£~Úa|è«o^©ÙðeÖþr1ýÌ½ÆFv×>%j]¨­4½êìçW¶q1ÍÁù?ó±ÂWü´¸ß¶Vc$L.åé¡$2ß©NÑÑ,¹ZW¶?þÚóf¨káääD2'½äììÌDär¹««+¿©I³V¸B¡pttÔ/EÛóÈüG¼Âq8sçÎárØ¬ùtßx3cfÍUWWgßæg<y©¾Ódj8àÖâ¿¥`GMP`ÙÓÁiéjýñè'ÿõéÊÐO7¾³ßªÓ×OÊ[õ"Ó<2<FòhM<mÛVd>Ißq?n1tÖëÌÕ§Xð0æ÷¦ÒïÞ¡0)àÆlæg2]³tòë÷Ñô!®²²ÒÛý9ÍÏZùÙ§O.þÛeÔ¹Op/ñãsKçøÍ¦ô7ë^÷½îSôUßl`æþýñ¼ÔÊÇÒOÉKvæÄ£íä­º5OGò:#ïð¦1·àôþG÷v¿×py«fdn	k?-xßÔp<qÁÑóo`G¸Ãa~ì+=ü¥æGDEEñù|6eÚ´iJå/þs`û4?í_o$--³fÍ1cÌÀü,.yç¥df9Ýõ#á<§ôiß¹/Ó9b%C&vá8¼¦#y	u»vÜ_­-yIOÜlI¥äi£¥à´vc.ùß°¯÷°(óÅ~~~lþõë×S$!!!22ÒxóËåÞÞÞlJxxø¾ûòé£uë0åød2?úé±½æg0««ðùîÿ5	Å>ýÊÇÒë§ù¾z;vïgÆaÉ¡$océbFòr/ÕtÜ5äýüÿ^cn[ÕuØ°^óJ¥Í^^^555l~>oooÌ¼ü3¾ðÄÄD6E"2¾¿øâ2[3¿3fL1È /a~@®×ÍW¦ûn¾³D0)LÎ?ØåtG |Â,wi×õ<IH.xãLÉ(_2w¿ý6#yüýq¼Õ¶OÑY_ÌØÞÈsÊÌ¯6gÀ2`d¦`~ºD"»Øî¢Úö%%%ÁüÀ°ì8VL7äoR	³ºÚÛ1sÿ*Xy©"¹KnþVZ¾Òô*¦S cYÉ#Ó<3G¾7u±]÷Æ§1wdæ'ûäQùHóc!Ï³À]µðôygYu«	¡ìyYÚÃ²äª²Ê.>(ËbÚîÕ­^ÛÛË®®6¦Â7(Q[ÎÛLspÜJÓÜÒùL[òHï"nû3G)ã&y¿0°ÆÜG÷v¿Ç4æÒf¿ÜBÄU¤îÞôþ»fg4+|Ék~¿hoùs?ûÕÅê&fZ¾;Mê×êÍêç]¨Ê¦J>ÿ¸w?>·Tp/qÜ]¾öÇGq;7nÜ¶~ý[¾øýíØ·oFE³²Ù¬?«è°]÷HøHûÌÞóLóswwçp8:ýütÀühìR?X1?¢ãµún3*¦¼±,&ÿ #|gJZºÆ£N´¯eÞäº|-IÞ ÍÁ­ãñM»1÷ÞÞ÷­td.Ì`Aæçåå¥?¼ÃÙÙ7æÌFÃKuë@/½º6uá3õæ_Ñ³I[OÛEûoìä¿¿èôñIäº|­ñoò<ÉKùzþa~Ä1l]¹0?5½½=©D"quu¥	3ÒÕªU0?`f»Ôõ?íP4©¥Ýêû­,kÖ¬ÒÖ3IVf«Ö-óo÷õ]æ¼A¯e%ÏåkÌtò?BÉ£Lb§Ý['¹j¹QJÊãV¿útn-ÖBXù±+úêQ¤¾¾^©TRÄçïùY=L?¿¶7­½ý*ue«úY§¦ÍRÈÿæàÖW6¹DÞáC!'?8Hæ7ú7|f_¾v4XûI£ßXµ5ævu<iñÀøð¢øÌ¢æÓM7óW´$ÙÖRnR	øocr=ÔõÆêÑè]*611qÐÉd2çììÌáp<<<¶mÛëbkæçèèH×;//¯¦¦"6mb"É	¯Õ%ÏÕ¥Mê¢FM<Oz>yºåºFªDÊS×¦)ÔV>	ß)aü¢Óä¿¿#çÛòÆ²~¥É^«/yæZ¾ÖìhwdGë4Ú«èXþÕ=N¯®8nl¸w¤êÆÖê»qå%)Tó«nî¨º½Ç*ãÿßÈÇÇG.ëhÉ%|>¿¯OóoV¡P|1.M_dd$;C»«»8Ì¥J#|ÅÏÕ=½Cæ!ÛkëQ?n×þ%¬~¡n©»­È[ºÏ$||néüØyQßð¹ã Ë×êKÞØÍlFV6±ÍÁÇþöãïÇvÚXc®¡KJzHcÿ×öUåi^(%O»7c)^0æ§¿é]TT~÷>2ÌÉÉ	Æ¦ÌO=°r3s])Bèíímßæg;°cAÑ¯Ñ¾ÊVÍXòÍ¾¯5úh´÷´_ªHþÏ³à7ðxÃ§½²+yc·|íHþ?s«Ïüýê°Ön>ðaìw8úcTæ¯lT²våóGýµ%w³zR'Hõ^¿¥çPxÏ¾_ýÝOÆóò¿ÄÇ?ªº±õÍst­7þ°îí¿ô»wF¨#Í"qqqê×HÑ¸ÅB¡ÝèââröìÙaÍþúúú666êdðôôÆ%¶qó3#>>>tçÍ=;//Oû#º)ÝÜÜ²²²LùÁü´<Bõ¦G í`hpë+²'KøB-]ÍÊþ*XùvÌ¾õ:««»|­µKÞ/} 1·áòÖ_4æþãõÎ1v]ëµM¦÷µRÚ¨×hîNFé2¾>¿õuÂ¦+vþ¹Ó¯"CJ÷jýtH'?º>U¤ìW¤î½y¡ï~íK(?Æ£òsVåm§%voÆ`ÈºAóLûlDDcl<5¶ÜÜ$''ë¯¿:¨ùéd(--%¿tww§SSSKJ02	æg26ºçûÉÙÙYû£ÐÐÐS§NQ$&&fÑ¢EF¦Àü`~Ãö6jZëÛÕírõ¸/éÛ­èÎ¨JHúdîß¬¿YôäV¿²Â¯·_ÊþÎ'¥Ï®©>¹iÌm.:?lc.Y 0¹<FsÄü|/HéúÊûÜ|ó.í0)üÄgdoäp¤t²µÞ(Òý'ÒøL£t©zo§]hÇþº»TJÖ>üáº»^m|vlÌ=@æ§lnèÞü~ðþÆ;·.¤T¾ù¡Ë>^I]]]3e0´3ùÑóZÿÐB¡ðÀAAAçÜ¹s¸4¶f~³fÍbæv1ãLÎiii^^^Ú)t§2w­B¡ F¦èß=²õó8÷nOA½YºsïÚ-ñYAÝë-yU×9¹czþééûöþÍ^g~sù³½×¿Ü»vcÞÒÕï¯¸ùÖºü?P|Wî¸Üo®Å]ÊþÑV/åµËçÎn+:ü;»Þ+Þÿ§ñå^ägefZNfFæücÏíXÌß<ÿÐþU§vþúÿ·w.`M^é¾g³y<ÇÝ½=uO=îc»·ugw÷ÓZfSk¶C§ã¸;n;ÚZJkkoVj­ÚzEDÅ**"ÊM@D®r¿[!$$$@HBîä¼ás2Kðÿ=ëáYYù¾Eø¾ü|×ZïºäÈ¹iw²ãnäÜ¾ZxýtÙÅ¬³ëNÖÛÙ|`ÿËß>_'Ùõ¼ì=s~J.úôej§gé:§³èú¡ÞÆðúäÝ§ßØ|éO5A=ï-¯ýÿüÝqýR>úßsk3ôËZ*îß¿LÎÇÇgÄù|>Å¦¡'VWWÛ÷6¿EY¿,zm¯^¯9s&õf3ÏÀºC¦îHù¥¤¤(+VöùsWNdáz1ªî^-·ÓP)4å·KÚuMêe¯bLþR¹B^ÓQRxð¿î¼öjÌÏÞJÿÕ;y/ÿ9õÇÅë¿eí¼ÞÂ»Q,Ìj5yüMï¥ñ_³NýuÒ*¢îÞXõßÓÕSÈü2ùàº°¯½þÁµ§nµÇG*B±ÇWö(Î¼¯øîMåÊO¹²g5ÒStF+²¢¥iJV²¹V)j×VÀé¹WÙíxI*i»~ÿnyÌÕYû"Râòk:>dqýR_³fÍ¶<¬­­]½zµ½ºÙæ&í%$$ðx<ÇÍÏ40×?,,ÌÒBßàà"fyù=$yt¿óóómîôc<gÎACÖ¦¿l2íHF]NKa.ë¦³åÃüWÉáFq"µÚO^c¿yð·AfSÎo3ÕKÍ35N!Ê4b¼2MxëjÓ£5ïå¯ÛóÜY+·fþç¡²÷ï·Oæöµ5ÛÛZ!Èc_Ù^qlmÄ»ââ[*a¿ójÍ¢5ÿu]ÊymÜ1ó,ºª½k­2Ï¢ûhUû¿Îß±óÆïöô=rfýñäP$Ò¹æJêzÈë/]Zèl¹í»ª¬£8>d¦ô]LªGêFu.ëëëk2Z»ÚöíÛù|>U¢¢¢,8¦OÎáp´Zm@@Sæ§V«-[fiÙ¶mÛÑ£Gþé©ààà;wâÖxùÍ=ÛzöÀb´#¤w$3ËH¡#-0?§¯7IÞyÐÙÅþc`áêQHÅhèqî%ðµÉ-.Ù/îÑJkºIò¢Cë>ÞSþ·ó_,þõÞò-_lßºauøÀ;;kïØ,ÚhºâÒØ¦Û3¶ÇOsJ³l3N÷ Êz³0â¯³èÌ,º¸c$fÑqI©+[7m¶¡º'så«×).oå&S¥"á(U½üúêËøÜÃýü¾@éçu5©TêççGÇ,Y²Ä#LHHðññ5kVHHÈ¹`l`öîbêdíÚµ3fÌðòò"CØ½7ó½<Çüé~¿óÎ;rùdXX´hÅ2$±Ä¨6nÜÈ¼é§¿¿¿-0?§G¼GÔô¬³åJÙÿÈ_>©ôí£|­ÆþIUõýÌÂKÑyß.ùø¢õ$yn8Xµ#Ôv¥LúÛË~ÀÍþ0>pÅ±üwÔbY1½jùº§µ¢ÍKþ®úÌ&Vðëæ¹¥ñjn#!:R:2<j§gÉùÌ]DÑYc¢³lwä·WÚ2dioÍÉÿJnïcÌ`Àç`2Í ÿXØ¯ýÝòÈÅÓDV­Z%­ÿo=wîvÞ¼yä¶Àü<(öß/øÙ¸þf¸6·#)®õ|pÝÇm"Éû¸äãµoø:®9ì!;¶U¢Ëof:âaSîgI<wüg¼öfTy¤çßÀ¨+¢ÓäÝîIæ_øsÍáË¾PÈ¯íà¯»¿EÇ(!º	Ã5sÄhÔ2IG)»êbUY0¿9¥,îc~M7uÉ4¿íñæç8[®'üðý¼å£89nô·¨ôvO#ya [Iò,^0¼VeÚÐgÿúzeEV0£LÛ,,`É[ÛèG]EGV×õoëÁuß½RqèÅúc¯	Âß'h&kÝã[àxç!÷5UQ®üó]+©P¥w÷*ªPcëÙß×dîUæ+ÿè?z?þÅ0=Ø|ÈÆØüç«¨¨pý¿æç0áT©/¿ú#2¿ôw×Üwê:WV-Jj9ÅþlOù¶øR9XµÃÉk=ÿPâÈöµ£¡¨µ((eïó!«6^ùí¹ü0AÀ,|ÍBJ&mb¢³E÷Hé,³èFÑ©¥¥ñÍwöWÙTºü]7'× VxÒÛÒ2Lþ·ÿåsc8üèâ[íÃÛò´0qâ7¼ü[IïUf_Ñ>eí¾Ú¹#ÿÎ±¢c­7ñ_jÆØô0|Á`ÍoÎ9ã´Âæì¹Üøí'¥.çüüý¼PýÃ×;Ôm)Ó-<²::këÃUL$/¼ñÀ]ÁµâÎfEJï²T*á[öÒ©w0Ø,¤©ëÑÒàZ³Kb¨%½°%D7è,ºGJ7lÙE­5å(©c²Ú_ÏTxËõõj¬¤ÆÇK_·ÝÜ[]Â­¿%é(Õ¨Yn"é5Nj¹r7>'ïô7±¥-øL²ù¥¥¥ù2ÂÀüÀøQÓ]Lª×«ë¾÷_¿QÃ;ÅþÌzu­%G÷ëí0NùßPÃµß±¬#¾¡/P¡ÊàÂ7(£ZBtÒ9¢cF<Î,:¥ ¶=ççZ@Å±µôSR0â¦eRàþÏn8¸:»"óZâÝsá	¡'ï_Ë¹^XMZîÔ|¸§ðäÒ17N¿ÊÚñIAìÖPÇZð¹cóûÞ<æ0?`OtËé~¸åkÙßÏ[ÞÂµbKî¿¿[øÒÁª¿Mj»ÂDòÈÇä7ZïùUT¡£ïË,j3=ä÷7Ë+ôùÉ#è¥s&D÷80æ¶$¬ÝÀ¾²Ì¯·µb¨<,Sëá`fR`ÖâaU¡HÇf]8ûnè7¯øuØ¡×.0AÄaJÐÚú§Êî§B¯ýçF<ÑºàfÆØü¼+<Às³ùxëG®s×rÍ1¿ñ­Æåï¼0æ¿+åÊ9¹6ì%¾ ½E­E;¢ë;´Ywõñ^®)·¥ÿA½!óþaº¾äîd-tesYaÌ`®9+e÷µÀÐ<Ëppy»¯WÓßoì±[¹É5å¡µWm9*ñPæ]¾L_Ó®­hÕd±ûXÊÏc:÷ÆKC3»ï¾´æQ2?ÇCâëë[XXhyHujq6v3ºPdd¤MSQ(³gÏ&+X¸pá¾ûpÜÞüÜ»ó÷Å'EÏ«²""S~Læ§>¿+¹v÷cÕG¯è 	ßc?ù,é	e37ß>Dg^ØéäõLÌ@lsEù×ayup|éõ=7|Oî%8âËK9wsXd.¥Z]Ü1GN2÷M,(^úAdÇUñ¦0ÑÆ³BªÐCj¤§èËyò é¡dº'1?©Âðç1®3µjÕ*ËCª¿þrt7h?Ë/·ôeéyÓ¦Maaaz½ùcM«Õîwæóº`ùxÚÙr$ùyËGq"ó"©_£2ïÆgÒu=¸põç#?Y³ïÅöCë7SlXÒÌ¢cFØÏ¢³QW>Ò¾zé¸.Æ`îã 3ôKzî¿FæâÊ$sçôæEg¥g>¼&:-î[ÿ´*à½Ì~ó_~±áÆÁ¯rC¯sÓ³Ê+*«ªjÙìäÎ]7ß<H©­KL­¯O¾öÕ±k·rSRòÒò=x¨»æ´V¹em_¨_ýufÇ¶ÌÌLªhl£V=¤wAAAöØ*'xo·4?ËL>ÌóCY[i,+ÆÁrýîo".ÿ0(ócwùmOzádOxÂ¼k¹¿»Yüulå-:&¦ðJLÖ©Û©Gn'~ëö§ÑWß¾¼=úì¢þ:ê»5Q_ý¢s×sfï'O_lÿä»ûÑ³û|ë/*Ó/Yè&-½0lí1±:Æ$ÈæäÕý¹ÃÈcµÌY"sïGJþûrÇúÓÂMa¢WÅôÌÉÆÖgß-+++½XQSÝÒJ]QÔí_m»OÇ)hI>óÿ¥k_È¾¾üA$ï·#^¿òù¥=~!·Òãîæ&¥0?÷âü»1aÛo=~¡~lz®­­]¶lU/_nÙèêêZ³fyÕG4?:,??1Èõë×h~ôsÅÀæýû÷ãùYfòa0úî~.¨û¾F.úú§â%×þYzk°äûíeÿ$×sìÇÊý¿6¹ÙiÑ%Ô&ÑåÞÒWe3!:O¸=úíÇ~òîíÉµI.½ÙlÛÍÀ¦.Çji«õ`.Õ©2GÆöYL'	½Ì¸ßMè¥ï×¨èÄVuB]u«þ¸Ê`ÐÈ:kHªj*Î4±oJÅZ£o!ew_aëö7iû_6/Ô Jy»G<!QþËÀ´]/«hVø#³yóæmÛ¶½ùæÖÛ·og´,222  ÀâgCÍóc±X+W®4dÞürrr6nÜhs@iiéÂçÏO¿4!!¡¸¸7È]ÍÏù¹;Ú¸cÊÜ=ÂÆMû72¿öÿ+­Jz[ÞyPÂIÀy²K¸C%¿iÐ­JCGªÇl§§:A¥U¹ÓO²Ç«Å¦Bù§Ha´Ì­	ûÇæ22×Ð¡£~¨7ksèí§E¤z$|¤$¤óGIx²ð¢ËÆù>¶)"áHfMv¶OgL;GB¸kÃÍ]«Ãö<êat%>Àp¹¯ÆÆFëÆÙ³g?ú?¡Z=wîÜAÎrÇ5kÖØáj~ÈüÈÿì.,,<~üøºuë¼½½¯]»äÞæg?°ÛÑÑñÌ3Ï,X°æÆEºê«Wûµò°ôåy?ÑktéWúNn³=L×ªè:'á¿&¨ÿ~Gë¹Õ_$n_~äG$|±¬R@÷¿ÆGÀ!#GÜ[WÜþÀÍs9Ò56£±¹»ß¨èåÙUáu¬sÂ¶zØoã%8dÅ­Ç³C¶DîùÜµO-Cúz5Ö'Û;ýï92Åïü/§°ñQùj¶o±5¼ù¨¬wÐÁçóW¬X1LÏÕÕÕóçÏÇò4óÓjµÔÑ^àÅÍjúþv¼D¹yâÊ©?%óÊ¹þÍÉ|ûÃnv¼[±ýÚW~èüÝ/Ãï^ËÉ*hUj<dÀÜNñ^Å9aÛÛoT0H¼ÆDæèadÎ!á3êZøÍ)dMìd~n°1ôJäv	G2I~uöðfÕ;¶'ÌÏË)k;¶)`æ7kÖ,G³&  `ÕªUrÜü   °°0KËÌ3mvù1cn[ß¾7,¾É`~À«eu$g9UÞ¿¹;ýgÌ]~qWLõS»2ßMüãHÿËç~íÊ¶[1»ÓÚÓ>¾»+>âÃSõMòÎz[Îx³Ì¥"Èûë`®±ßÔ¥~,¸]&YÉð¸K9R45£.+s ÓöÊ:k¸ÛæÕ8·©N-øz¢¿ºwýóäÈ/î¾ÿzä®ÃÆÏÏ0óÛ¾;Ï§JTTÔÊ+G4?³bÅ­V»dÉçù1¨ÕêeËYZ¶mÛvôèQæWÓSÁÁÁ;wîÄrKóãñxÌJKèØÒ¾èèhó_ÃMdi^µo6>-âþP«.7õë]ùYVæ²¯lwt0W¥3	zÍ&µ~e§³ÜBæA£Ø7ëXçøÍ)½=-c>k®ãîÃ9ÏÔReÛÛi¾x*WÒ%ïïÊq*LT*õóóóöö&³¬ùfÇºuëòòò¨úê«¯:õë"##--díÚµ3fÌ 7=öîÝ»É&qÜÒü,¸æ2^»Ð«ëîT,©~çoêf§ßùiUáÙôÞË_¤Æß]ÿÍùâÓ¬;1ôwãèï×hYÝâOÛþEÔôlwÇGôÐ¥ðÑ`®ÕÊ ÖÁÈÜýå¢Ëì¾ÙY.ìzÐ&ÉjK½#8tUðîÏ¹!·SôòÛxidìªp2?ó¾þqÿÍùã¥Ï£Dÿµ¯êÓHáÇÿêÜÞØÎ·Ôïå:Þ|ÈÆØüÜ+ô¸¤õK9kiUÍO¨ÒÆþ*iùó3JÉÛÆýãX/øeÐ©«ÈüDÜµoÓ¨rÉ'Çöz%ÁÜÊSRÏÖUTóÖ2gÛ&²ÌÐCÛÈXcäõªÄ§eR(OaïyþÔÉÍ×÷¾xú;ÿKßÑkõøÜL²ù1ÀìéÓ§ûûûÃü#(»¯t	w8XrËYZíGòy±Ox[:o_KÊñ¨CFA´êryçAQÓ³íOä[´Dæí|(½ùLêÙß½q#xï7ÁQ[O±9Á¢3¤ªG9©p»LªÇèÊÞäaBDÚÖeWBÐ¯Î4·Ð0éæ·iÓ&fÑh´(°yófG[â:V3Îì>ö¢¢&¯üãÕ·ØQVp7áëoÏ®w¼*ì´¦×6y¾úïËÚßRözñ¹A·ó²Ï3g9j¿Â½wæÝâÃ/ÛY¾ª¼¤M¦¥ÌÎÔ>0#°H`ªëÏ£R-	YÚxiãebH;_pèµø¸¸ùÍ9</??¿¶¶*>>>|>*Ôó#¢¹ü9ÜDó6»#aÙ¢¶­©ó]ûe^uõ«ÉEÂ2Ùö#­í¼Ï3g=K¥5å¨¥Y6,né6¯.Ø,êÜ/ÂdÀGþçîÿL`~2?ËR U«VQÙûöñ@ª0¼Þ±/åìêà?-=ôïÇ¾Up§BéØÍ§3Á¹R98R­$E»'']û.¥Ôí(	ãsö@Ü.âäTæÔ­ie?ÙÒðr3ï¸»Ùàee.çZÛ¤YîÓÚäæ 3#°µÇtm¬²¸Å>À]ÍoÆäy]]]LðO ¤¥¥!æÆ¼9æ¯óQG»J©1ó4ïµ£yÛÎë?G7R¡#sÍÁ9êNb)éÄâf5uÂéGl5z=×eío	ê¿/æùSjìcï5DdYKþ×o4¸Ù§+Ñ¥65wÊE®9#Ð¥ª4×ä^ø`~·4¿µk×Zæö1[b'.çÉÉÒ¨ò_W/=´jËEó´¹ßµ£É¨G/UÆcZ|¿F%éîhoxJÄý))`_wõ`.UäÜ"Ø3÷/2k0oÂ3EWÍAÁIÚ,IÈ"lË©c|cKÖâè©ÎÝ?;Ne'RÁç`ÍX¾|¹i_WWi Ã¯¯¯þµ0?wçNòÜóJ&æG&îýHÉ$¾$;9¯¸5ãËÚð+ý¢îÆÊ¶¼?+D.úñÌËdêÑx=Ì®Áæ5Â]jó"ã/|Ý26y^uYûNàù=NÁç`ìÍÏ]ù¹;Ì<¿R1?µ®?(^êà<¿±eÐÁN¥QåvEMÏHIL8Ahô&±Ò<°P0N@KBfXXäIøæóZp¥Ü¢Ö"ÇË­òÏ§>wô·>¦Êgñ¼"§zÐèG©bF½VVáà`®¶¯¤G4~©]cÿØ-	YX¥'<x¸ùÙ¬Þþ!ÌEPÂ®U|&²´uµ:e¹ÇÖ6ÇåìÊRz$ákoÍªc«­s¯	|óùá$|UzàYúºFÎçÇæçýÍ`îã­ÕÐëZ­ê';ùþ¤»ß]û@`§Ê>h4êºeìVnrMÅ6ë°-gb¶ÐÌL4ú¾Ò,gKÖåäp£8ÊP©UÌk5ZJÛ2N×]ü3+«Í¹$|ÊîpFé'ÕI=ÿN3Àzé£@`k^aÀÇåÜ®*n¨».hÔ2üÌØõíßY JXßQÕù+øõ¨ÄÅ·ÔÒ	ò0&5`'ßIØ+=>°OÑ!lÊj¨¸Ê/íª)îÈ'`i0¢æ§SÊ:+¸±_íÕ]üS[ÆéÉÝEIÈdfRjûJ<ì)v~võevU¸Ñ+ç'ðÙÍÛùÁü<¸rEP¼ÔÙrâXHÆ¡7:eo¬øóëWä¼Uº¡ùÎ~YõR@º¤jezp°ñ¢¦g$AU®û¦44=]~sJMÅ&öM¨¤O%üPfF _h`~0?÷¦¸Y]ÜëlI+8¶~ä#ä×Ò.ÞÎ	=õäÉ³g/Å+èl­w]ÔúõîP«wË¹õ·jÊC[ïP]¯s&ç"àæ7<0?0µm§+­+=ø<ûÊvyKé æÑ#Æ6FR~ä%:ïiÌ§ÝôO¶I¨V¦»¦ZÍºÀkJê±7à:æç5ÞÞÞ0?0¶ôõïrc¿ä§VnÕf°_WjMk»ØÙ­)G4Ë£H¼çú0©EÜ2©]$;´e§&*ã@sG`~î¬ú>ûÊvò¿öK¤wF½¶=ûbUÈzÎµò#/5D~ .¾5LÞÏQÀÔLvèIIhÀW[ÖÄ¾)äMæó"²Â$å	Táß?Y~Øl¯öüË¾óUj'qeîä*àD¦´l¡ËLàIªôúIC·öjñÏ`~0?¡ýÁqñ-ª´e­<ñª¾¯Ç VTÿ5®mj@ÙI½¶a¬:g¶Ðm¨»^Ç:×ÆK3çºÚj@ «ãQ P]@æósgz[+ªB7ð1£½6Ç+cá/©·µ7ü) §®M?F=yÙ9ù&HòWÜ@ Àü`~î?-¤&ìM·÷T¿ÎO=Aþçj9ùr¾î:ÿ]Äý1UÌ©G<EÛ+ë¬á5%U4±oJÅZÜ]ÿ~æóóºØÙµÿTöÝ²Â<lõî8¡í+wìhù)`xvhÒ;±°T¯ußÒ-c»Üxîcbk°.óù÷À2ÚB¯å*»ÃÅ-/´7<ÕÙ¶¥KrGØM¶GEØ3^	YK °*h3q»LRI@ æó0?O¤ß¨ï±[¹ñµÇØª7[ßQÉo=SîZh&¡ÂÄÇ+;L-Ýf)DóùÛKF.ë¬árnWð¨®Óöþ²(X*ø½ó¤¬Jc4H§âRhM|¹ÙÿÛM2@Ìæ`~îuB~sJoOËPøúÊ¾Þ$©àíOIø¯m^·rd©Ciªë4ÌãÂ*Ì¶*)+ÅÙBÚWzà£8ûnàë ¥_¯V¦w?´¾¢è:§×r§â[ÓØoêV¬@`Ã@ PkÀ¿YÌæFO[Æif§]§iQHEÛ+ñÈ+i0hÆ8!K¿^£ÊKöqÚ#Þ3EÐÍÚÇL^óè0Àü`~L0,5gÆ/!NS'&ÿëhù¹ ¶¯d^î~©Kmjî6UÍ@¶Ô,F£Çûó%yyyK.õöö^²dIaa¡õSôpÞ¼yôÔâÅSSSlùÔ~£¢/<dWOðjz]«¼ó ç+jz¶KhpÁ-»óåæ@à3éÈ<þpeó[´hQNNU"""yæë§üýýÃÃÃ©¼~ýz[`~Àso !¿9¥¶2lÒwT#TvKZ_1+ pZÞß¯ª7f ØÒmª&Î@ p¨t$=ël)n'RW6?k¦OnýpöìÙF£9ÏV«?¾-6æwøðáû¸éÉ9Ù7óÃ*äÍÍ¾A-®óò²2£~V_ñ<¯vAMékyAiiÉSöfeßÏ¨É,fWëòZzsZ²+ÓsÓî§=f·Ü¬ò¾.þ-x0óüüü­[·Z·xÛÔi±1¿»wï*pºdm­ÍYìêVé¦úx¡ L.¹òî·ËÄáâÖÍÎ:Z^£:µLå;¨wk:åíýmúOªì®+m£ÄX$À?Ïæ`~ ËýýýmþxyyYêÓ¦Ms°£½À½Æs-	Y&xßXþýs^áaãÓÖWÝáû¾¯Ì@Ô<2ñzÌ8µkpkyÀ<ÒüÁ-[BÛ¹9sæhµZf$ê¶ÀüëÃ$dá7§T°«Â'wßØ* FÛ%5=KØ+=®×µNõÍlÂê0¯aw·sdi0Ìà©æ¶zõjdk7n¼páUè§¿¿¿-0?à²X²Y>×u@sjÀI) ç+ï<8E7±Æ:X*4géR+ÍÂ|ÙÿôØSàYæ7þüïYñèUT²³³çÎëåå5oÞ¼¼¼<[`~ÀÕP÷u2	YX¥'-t)´*VÛWBæ'âþU¦nj@k¬u¦ö^SîÑS2sîhv§©¨Ý¬%Béd~ãÌLL>~KjMÅÚÊ°á·Ð"èµHhM °Vb>á¯£½-ÝæFùÁüb4êº¤uÌjìªpa[R! ÄùDjÀAQhÍ;´·#,X%£7¥`~0?àJ*£J¸Û$|ôS*®T÷uâ²Aß®ì¹ÞÉ÷6>M¨ÇLuäõèFÍÿèù`~0?à(v~6§&¢¦<×Ô%­3u¸,£Àhè!µ¿ÕÞðý¤:µLÅ!í3¯í Û³öz1ÚùÁüÜc[kYc9ØJn'ïæxL>a[N¯ñÜ±¢¿_£Ç0©;ùþ¤S.5 »Ó<æÛ 3·ý¯Hð×Åóù¹5û:gËÝ-*r¸QHEß×ÿø/[§ííÖµrkÊCØ7%¢MÈâ2Èdr©üÚMÛÌ+?°°óùMAj¯iIþ&þ÷ÞuË¹Û$|ô³§3¥²¸ºwjÀådÜv¶ÊÌãå4âD*xË`~æç*±Hð°¡îzMÅ7ÝQÍ#qÇÔÚ÷r*-AéZrFq"¼O0?ós¥B àe°«Â©t´ !Ëb°Gäy©³ÊëÛrp£0?Û#TñS-UéÉ£8^ß7Ìëa¶Ðå5%ÕVq9·%¥HÈâN¨kíp ;´gäù`~0¿©k~âäÊâ#TX÷[êÆÆütÚ^¨¤¹!Ù`äZpwÜsjÀìÐ.P©3õh--E'ÀüûR¦¹·MUù 4=¬,DswJÝ5úµº*q;?»¡öjMÅªÈ»¹X±áa¸Àj±Ùá&²Ì¸)TO#ï¯JÉ)Ë£má!µs*`ÔõÊyüæÚÊ06ëXX¤Rv`ÇcPÂI!;mÐ'áuh&Þ©R þ6?ÉýÛ·xMµÎÙÓqë0?à®ç5ÄÓØV&æ¦Ë¢RÂze_çÈa?­F.ë¬avTkbßÄxîTV@µ2]Ö¾MÀyQ@ºÊe_íÁ¦ªÙ÷o¼PpïRo<_poe^r·NùÁü¦Õ´Üäó»Söà³¾÷ûn½¬HÜ¤¼µFûªâîUF`_öî¾ÂCêc2ÅÛmuÅÑU%gX	ÍzäÂQ@M_Ax°ñis^É~¦Î¥^aItnÚÍ>Å^NoKeÞVÖCÜ;Ìæ7%hÍÐ§½£Òöõß¹Wq¶·§¬ÉÚõhÅ¾¯_Ùa±b®éAC]nfeÞÅÊÜå	÷O×e¢¼·MEjHHH²ÈhâÃfG¬ÓÔ^Ó6Þ1oò!*5P'ÔÕãÌ îV]Næ×Ñüï¢¦g©¢í+q!_7²ö6Tù½]?/-ZD¾ùéÑ¸_ÌoJÐo0­©È;Ut+¡4ý,ëá¥âØ¨.Î£Á/£Q'ë¬±ì¨6|>²:r»ÎC¾ls[GæWqZCûE!y!J´8"µÓ³e!fGdßÔ2(azùpDÏA¯å7áþXÀyÉ3®¿®S«©îíÎ®¸gxõ9[Y_)N¾àÞ¿fÇyßø»ä+2nÏ¹õ©7Sþ×½k¸MÌoJ QËÈêØw¹¡Ù¥iaßª*ãø-÷ÅÂ¢ÆºU¥Á-ñ$c».ã2¶<llvÄêKZÆ³w÷ÝÛ¦t¸ÙÚùÙzÆ©+¦i¹:MÌó6>-kkyaºuZ¶¢¬îFóy¾y?W¼VÉXÝ?¤F~/éòìû7¨N-oVäÐ³tù_¨Î¢s[êè)óg×_F©«yÉ¸;ÌoJ yTXÛVØS[yU|ßê	YHéG$É#Õ³vDAÆáfÆé¡eJ"fíÌp3Ñ0¤Êî+mÔ½IÔ"ÒôY¬Õ·m,ÀXÝÜ´duävÕQûVÖÃ/êË­­®S;òÛUm4ü$7yGmIDATqKe^`mñS·/µ5ÎONëâ¦`~0¿)Ak¬³*I¥éæ|*e«ôÛý!#öò#ðöY;"3ÜlÈ8"3ÜLPVZ;"Ç­>EA·$E" Ó:ØTe±º_æßy:ãÒaduôóéÌçó©Ý«#d¬¼p¬^O·NKæ÷¿ï]ÿ»¤+?Ï¿K/÷óùM:ÅåüæÓ£9¢__ÙÃ]¤«qDf¸Ì¯,äoq)äX¶2ÕÂ.#¯z©(me^òÛÿãîU*T¡ÔHOÑöVg4ô¨ä7eío=JØun¼SZFæóBÚÊ0ò?Æü´9i_´WÆâöSÉÿì­3%Ñ­:;¼Õü:÷+ûõ¾nñ§¤|sjÀòÂÈ:EeBgË·e«/¥âD*øwùf9^ÚKºËîæO.¹¶èö­úS§SÁä9Ó°S]¶2ÌÄY¶b	×3¯~ZæÕYF`GouN`¿F­¸Çl"nyAÞyp(L(¢«:ïvÌ¸d£ø2Ëø6Ìot_¤)¸ìÎ:â0S]¶2ÌDGtjv¼çÕ=>¾¹d¿¨éÙ_ôHè¡õ³JD.hs¶ý¼Þº£8ÞÃp!4ró¥³ÏÉ«-?7©ôpÕÇÝ-S®¨²+÷õÄ~ÐõN×7»£-'Xßöñ¡m­ÞnzïÃÚ>©ûà`M`hÕkÄÕOçl¾Û(pÝøaÎ-=Lþ'âþP­Lïï×¤Ò_älùúÛ§7Gq"¼30?àöëXçp&ù.8Ù~:µ¬.·¶³ªXÑZ¢~J¢ýÒf±f×È( ÷°ñé?¥ËâlùíÞb2¿QHoTÌÀüÀz^ÝÀÚ/m¶kfÍÍºæIâ©×µæñCãê¶ÞªüùÎîü¸LI]®K|HöíMÍi_ðfÀüq°©ÔÁÙ)­èÄ(N¤âRsÂ&×ê<c^Ã kVl®ÑhöÊNJø¯	8Ov¶mPÉoRË0ÇËH|³ÌÏí¹WSâl©,>Be'RéPJ=û?;.ÉMÜK|])?b¿FÙsä¯½á)1Ïê6¨÷<ü&;-0ùÎïÒÄ5ÈaùÁüÜ¥B ë¬q¶¾ûVÎåQHÅhÔyªÕ92ëÙV7æø8ùâtü#:")`_oTðçÉ_È;ôíýzýý%¾È)9øûFvìØß;*«qk0?ßÔÂó¶4fvâ§Ögt¬¹2LcÉhqÄø7G,:¬.=avDÎm³#ÌÃÍ®§õ¡¬íÝö§X7>L7O§ª·öüâ`~0?[Z#ÛKÀê<	²ßâ8¢µ#¦üé#ÆýFöAjÆîÄÔ÷²²>¾³©ÁdÞ4DûlËùÁü`~æÕqrÄ¥§3Èó%&þÞl~]Òø7óùM%ÚÔª×K3ÿ1õ¾|×`Úë0¯LíÅ	xªNe´·à»Ü½pe0?ßTá7ûþçÜùÿö Þ·0õqÔ$²B.#E»*àæÖ|Q_¾õpøAXÌ«IYXRüÜ	w65¤½_yo¡J&ÁeÀü`~S-Ò¾)3Ú«ïï­4sW]ÉPVGG¦uÉêÎóla©&£;ÔòfEõóu#Ä ,p)øù©nùÕÞÓõÉpA0?ßT	ËQe§âÉôèøxsÝÿI»ÁXÝeYÕýCj¤Õme=¤CZØp]§Vë	ÜdrÀü`~S÷kÏóÍóÜ·WürÄÏóïíy%_!«³ÕÀü`~né©ÉjmoZ§ðG9wpeÌ`~0?O£[§%á#íû]FnÉ¿fÇÝòpeÌ`~0?D¤é­4ó¦ûäùéÑÚðuÜÄÊÊ¥Î[kTFq"Ì¸=8ÿ~×¸9$¾ÙèlaÌo'"RùÁü<âVzÜ¾½Ìæ`~Ìæóù0?Ì0?+²¥2ïI'²´ô)pÙÀü`~`È/µ5NdQèõ¸ì`~®D"?¾aaá¼yó¼½½/^ê`ÌÀEÍ/;;ÛÇÇçßäùûûS%88xýúõ¶ÀüüüüüØlö æ7öl£ÑH­VËi±1¿#G¤ÌÀüY···MÝóKNNî&	ù9j~^^^ú´iÓl±1?öæçæ7gÎ­VkÉ¥º-0?÷3¿7^¸p*ôÓßßßÁ;ó0;;îÜ¹^^^óæÍËËËs°æàÒæ7NÀüóùÀü`~0?ÌæóùÀü`~0?ÌÏyöìÙsãÆ`èîî|ßÁãñ>ÿüó½À$¡'ó0?ó0?óùùcöîü[¦M¶xñâ£GZ`VXXØþýûqõG¾á-xyyM>éÒ¥!!!¸tð/BëF£133sÑ¢E¯:óö)õ¯­­]¹råñãÇqõ0?ài_ÕÕÕô]8èÐ>0¥Þðù[°`®æ<óðöö¶?Ú¦ÔÞiÓ¦áê`~À3¿³³³íC ¤¸b`¼á­©­­]²d	®æ<íP­VGDDÌ3çòåËÖö½óÎ;+V¬ÀSáoøÌ3ÏÄÅÅáê`~À¾­5k¿¿JJõAÞûö>|xöÞ3g®[·Îúù÷þ"d*F£ñÀ~~~R©ÔæËÜ>:fÙ²e×xðW	óÿEÈ°oß¾7ß|sJKKW¯^ë¦È`~À¿	»àààa´LÀãßðóüE(H|||x<ÞP(¥Kvttàê©ððä/BÅb­[·n7nÜ«¦È`~æó0?ó0?ó0?ó0?ó0?ùùùùù0UHNNöóó1c··÷¬Y³6oÞ[;¾(àÊÌ0¡DDD|Ï3g«üÁüæ~ðÉårzÈãñüüü¨åÕW_ÅÅÀ£6myuÏçSËôéÓýã 11qÉ%ÞÞÞ/NII±îúøøÐStfýTjjêÒ¥Ké)êíùççr¹ÖØT*ÝºuëÌ3é©Ù³gïÜ¹S¡PàÌ0J^õU0R«M6%$$FÛüxyyYççç3ÏVTTõTaa¡Í ²%hm~Ãô°nÝ:ÞyçÜ2ùFD"Yºt©õ?²«ÆÆFóó÷÷ïêêËå)ZÚéajj*Õ³³³©¾~ýzæ©×_nÛ¶M«Õ2OÍ1ÃÞüé1BGu²RëÀü£$::dÎÛÛq²éÓ§gffZ[P(dRDæ!UìWX?5èø¬µùÓ£¤.$Q©T¸Sóz½>..nÕªUä[d]öfi!G´~Ö//¯¡NÔüéÍfÛÄ#éåá6Ì0JfÏMReYAF·³ÐÑÑa1c=Ôjµö=3Á<fÉð0æ7LÅÅÅ_|ñ3Ê<oÞ<Ü2ùFÉÖ­[É¨î96MIÔ>üðCjY¼x±µ¥ùùùI$JµqãFz¸iÓ&æYFÈvîÜI¾Lõ%K0O1óüé©ÔÔTª/X°ÀÞüéç`ÿQÚ´i¸eó7gÎû!×èèhkK>ºå)Ò/Küü|£¢¢§ì×ö8pÀÞüéaýúõ6O½õÖ[¸eó.»eË'x1<???ëråää,]ºÔËËkÙ²eÙÙÙÖ§'&&R#=5oÞ¼3gÎX?Eý0gÑSAAA6ØB¡øðÃçÎKÓËP«Õ¸_óÛ?~ì´0?ÌÌàQL×`~æ`~æ`~æ`~æ`~æó0?ó0?ó0?ó0?k¾ÆØö?®¯_¡PlÞ¼yÚ´iO<ñÄöíÛ;::¨Q«Õú%''Â´TWW¯rþ9õ?oÞ¼ààà	û»0?ósëþB!UF#Ù+¨Nb·víZûç#¨Â´¼þúëiiiCuN=/0:::çóùä¡¡¡0?ÌóþgÌa©M>*;wîæÅ0õÜsÏ¯¶<¬®®^ºt)Ìóx ùÑS¡¡¡.´©K$___ooïÕ«WK¥Rûm:ill$a¢éD¦±°°pÙ²eÔÃ9sÈÒì`e½¼¼,Y=ÌK6m	S×jµô*Û¶m²?Ø&æ·nÝ:Kç²hÑ"7)FùÀ¸ßPóü¨Bþ¤V«mêäU/_fülëÖ­öÛô¿ûv³üü|:iôññÉÌÌ¤JLLÌ¬Y³ì 3c055ÕÞ&­Ù¼yspp0õO'RÿÌ_»v-¹éè·+æ`Æùzå,kÕªUÃ_Ë 0Ìóxùó%f]â':ý´ö6Ë6Xâtâ0/ÀºR·M6öéõúá_WW×ÆIÑ,XÇÄüèU1âHÚH¿"//Ï~ÍÌóLQó±nq£¡úô`rÁýû÷±ùøøØÌ½³À(Ï9³¢¢ÂÁ¿ÃáÌ7Ï¾ÑAkýk,YÂår-É#I.a~`ß¬Y³PÙæ§R©gÏÍ4®X±"(((!!Çãj~ÓTæ%&&fÓ¦MöØ_^^ÉnÍÖ+<H/_óÀüSÈüÞzë-f,LÈzßP|ôÑGTÉÉÉ	`§OÎáp´Z-µê^>>>ÉÉÉ¦¿ÌÌæõÓ$|Tár¹äs¥¥¥L#ý:Fû._¾l3S°°°Ð2Ãoø)ygbb¢i`aïßóÀüîj~Ã¬ðÔü$ÉêÕ«I|­×öÕ?yÕüùó×¬Y#ËÆ³Y³fúëHÎ/^ìååÅ¬ÆæWê-_¾[´hQtt4ÓHZI¯pÚ´i¤7o¶±%è©üü|¦n½æcÐ×O¯éþ3gÎ8bÌó0?óùùùùùÀãÿgYï ûIEND®B`


UNIANOVA yie BY Species NitroS
  /METHOD=SSTYPE(3)
  /INTERCEPT=INCLUDE
  /POSTHOC=Species NitroS(TUKEY)
  /PLOT=PROFILE(Species*NitroS) TYPE=LINE ERRORBAR=CI MEANREFERENCE=NO YAXIS=AUTO
  /EMMEANS=TABLES(OVERALL)
  /EMMEANS=TABLES(Species) COMPARE ADJ(LSD)
  /EMMEANS=TABLES(NitroS) COMPARE ADJ(LSD)
  /EMMEANS=TABLES(Species*NitroS)
  /PRINT ETASQ DESCRIPTIVE HOMOGENEITY
  /CRITERIA=ALPHA(.05)
  /DESIGN=Species NitroS Species*NitroS.


Univariate Analysis of Variance


Notes	
Output Created	10-MAY-2026 19:46:07	
Comments		
Input	Active Dataset	DataSet5	
	Filter	<none>	
	Weight	<none>	
	Split File	<none>	
	N of Rows in Working Data File	84	
Missing Value Handling	Definition of Missing	User-defined missing values are treated as missing.	
	Cases Used	Statistics are based on all cases with valid data for all variables in the model.	
Syntax	UNIANOVA yie BY Species NitroS
  /METHOD=SSTYPE(3)
  /INTERCEPT=INCLUDE
  /POSTHOC=Species NitroS(TUKEY)
  /PLOT=PROFILE(Species*NitroS) TYPE=LINE ERRORBAR=CI MEANREFERENCE=NO YAXIS=AUTO
  /EMMEANS=TABLES(OVERALL)
  /EMMEANS=TABLES(Species) COMPARE ADJ(LSD)
  /EMMEANS=TABLES(NitroS) COMPARE ADJ(LSD)
  /EMMEANS=TABLES(Species*NitroS)
  /PRINT ETASQ DESCRIPTIVE HOMOGENEITY
  /CRITERIA=ALPHA(.05)
  /DESIGN=Species NitroS Species*NitroS.	
Resources	Processor Time	00:00:00.36	
	Elapsed Time	00:00:00.39	


Warnings	
Post hoc tests are not performed for Species because there are fewer than three groups.	


Between-Subjects Factors	
	Value Label	N	
Species	1	RK	42	
	2	RP	42	
NitroS	1	Pep	6	
	2	Urea	6	
	3	YeEx	6	
	4	NCl	6	
	5	NS	6	
	6	Pep+YeEx	6	
	7	Urea+Pep	6	
	8	YeEx+Urea	6	
	9	Pep+NCl	6	
	10	Urea+NCl	6	
	11	YeEx+NCl	6	
	12	Pep+NS	6	
	13	Urea+NS	6	
	14	YeEx+NS	6	


Descriptive Statistics	
Dependent Variable:   yie  	
Species	NitroS	Mean	Std. Deviation	N	
RK	Pep	.53467	.034122	3	
	Urea	.98967	.011150	3	
	YeEx	.69833	.034298	3	
	NCl	.37433	.020793	3	
	NS	.37867	.020207	3	
	Pep+YeEx	.40600	.028054	3	
	Urea+Pep	.54700	.025865	3	
	YeEx+Urea	.35200	.006245	3	
	Pep+NCl	.31233	.050639	3	
	Urea+NCl	.49200	.061879	3	
	YeEx+NCl	.63367	.030436	3	
	Pep+NS	.40567	.019502	3	
	Urea+NS	.44467	.038397	3	
	YeEx+NS	1.21867	.023352	3	
	Total	.55626	.255295	42	
RP	Pep	.88800	.040262	3	
	Urea	.44567	.044557	3	
	YeEx	.94733	.032517	3	
	NCl	.30567	.007095	3	
	NS	.56733	.026858	3	
	Pep+YeEx	.44300	.019287	3	
	Urea+Pep	.57967	.008145	3	
	YeEx+Urea	.47400	.011533	3	
	Pep+NCl	.46733	.029956	3	
	Urea+NCl	.36133	.038631	3	
	YeEx+NCl	.40133	.017214	3	
	Pep+NS	.78867	.023180	3	
	Urea+NS	.90900	.050478	3	
	YeEx+NS	.45067	.044770	3	
	Total	.57350	.213474	42	
Total	Pep	.71133	.196386	6	
	Urea	.71767	.299374	6	
	YeEx	.82283	.139620	6	
	NCl	.34000	.040095	6	
	NS	.47300	.105501	6	
	Pep+YeEx	.42450	.029569	6	
	Urea+Pep	.56333	.024784	6	
	YeEx+Urea	.41300	.067335	6	
	Pep+NCl	.38983	.092694	6	
	Urea+NCl	.42667	.085151	6	
	YeEx+NCl	.51750	.129162	6	
	Pep+NS	.59717	.210651	6	
	Urea+NS	.67683	.257470	6	
	YeEx+NS	.83467	.421861	6	
	Total	.56488	.234054	84	


Levene's Test of Equality of Error Variancesa,b	
	Levene Statistic	df1	df2	Sig.	
yie	Based on Mean	1.641	27	56	.059	
	Based on Median	.493	27	56	.976	
	Based on Median and with adjusted df	.493	27	30.401	.967	
	Based on trimmed mean	1.530	27	56	.090	

Tests the null hypothesis that the error variance of the dependent variable is equal across groups.a,b	
a. Dependent variable: yie	
b. Design: Intercept + Species + NitroS + Species * NitroS	


Tests of Between-Subjects Effects	
Dependent Variable:   yie  	
Source	Type III Sum of Squares	df	Mean Square	F	Sig.	Partial Eta Squared	
Corrected Model	4.490a	27	.166	164.527	.000	.988	
Intercept	26.804	1	26.804	26516.965	.000	.998	
Species	.006	1	.006	6.173	.016	.099	
NitroS	2.109	13	.162	160.483	.000	.974	
Species * NitroS	2.375	13	.183	180.752	.000	.977	
Error	.057	56	.001				
Total	31.350	84					
Corrected Total	4.547	83					

a. R Squared = .988 (Adjusted R Squared = .982)	


Estimated Marginal Means


1. Grand Mean	
Dependent Variable:   yie  	
Mean	Std. Error	95% Confidence Interval	
		Lower Bound	Upper Bound	
.565	.003	.558	.572	


2. Species


Estimates	
Dependent Variable:   yie  	
Species	Mean	Std. Error	95% Confidence Interval	
			Lower Bound	Upper Bound	
RK	.556	.005	.546	.566	
RP	.574	.005	.564	.583	


Pairwise Comparisons	
Dependent Variable:   yie  	
(I) Species	(J) Species	Mean Difference (I-J)	Std. Error	Sig.b	95% Confidence Interval for Differenceb	
					Lower Bound	Upper Bound	
RK	RP	-.017*	.007	.016	-.031	-.003	
RP	RK	.017*	.007	.016	.003	.031	

Based on estimated marginal means	
*. The mean difference is significant at the .05 level.	
b. Adjustment for multiple comparisons: Least Significant Difference (equivalent to no adjustments).	


Univariate Tests	
Dependent Variable:   yie  	
	Sum of Squares	df	Mean Square	F	Sig.	Partial Eta Squared	
Contrast	.006	1	.006	6.173	.016	.099	
Error	.057	56	.001				

The F tests the effect of Species. This test is based on the linearly independent pairwise comparisons among the estimated marginal means.	


3. NitroS


Estimates	
Dependent Variable:   yie  	
NitroS	Mean	Std. Error	95% Confidence Interval	
			Lower Bound	Upper Bound	
Pep	.711	.013	.685	.737	
Urea	.718	.013	.692	.744	
YeEx	.823	.013	.797	.849	
NCl	.340	.013	.314	.366	
NS	.473	.013	.447	.499	
Pep+YeEx	.425	.013	.398	.451	
Urea+Pep	.563	.013	.537	.589	
YeEx+Urea	.413	.013	.387	.439	
Pep+NCl	.390	.013	.364	.416	
Urea+NCl	.427	.013	.401	.453	
YeEx+NCl	.518	.013	.491	.544	
Pep+NS	.597	.013	.571	.623	
Urea+NS	.677	.013	.651	.703	
YeEx+NS	.835	.013	.809	.861	


Pairwise Comparisons	
Dependent Variable:   yie  	
(I) NitroS	(J) NitroS	Mean Difference (I-J)	Std. Error	Sig.b	95% Confidence Interval for Differenceb	
					Lower Bound	Upper Bound	
Pep	Urea	-.006	.018	.731	-.043	.030	
	YeEx	-.111*	.018	.000	-.148	-.075	
	NCl	.371*	.018	.000	.335	.408	
	NS	.238*	.018	.000	.202	.275	
	Pep+YeEx	.287*	.018	.000	.250	.324	
	Urea+Pep	.148*	.018	.000	.111	.185	
	YeEx+Urea	.298*	.018	.000	.262	.335	
	Pep+NCl	.322*	.018	.000	.285	.358	
	Urea+NCl	.285*	.018	.000	.248	.321	
	YeEx+NCl	.194*	.018	.000	.157	.231	
	Pep+NS	.114*	.018	.000	.077	.151	
	Urea+NS	.035	.018	.065	-.002	.071	
	YeEx+NS	-.123*	.018	.000	-.160	-.087	
Urea	Pep	.006	.018	.731	-.030	.043	
	YeEx	-.105*	.018	.000	-.142	-.068	
	NCl	.378*	.018	.000	.341	.414	
	NS	.245*	.018	.000	.208	.281	
	Pep+YeEx	.293*	.018	.000	.256	.330	
	Urea+Pep	.154*	.018	.000	.118	.191	
	YeEx+Urea	.305*	.018	.000	.268	.341	
	Pep+NCl	.328*	.018	.000	.291	.365	
	Urea+NCl	.291*	.018	.000	.254	.328	
	YeEx+NCl	.200*	.018	.000	.163	.237	
	Pep+NS	.121*	.018	.000	.084	.157	
	Urea+NS	.041*	.018	.030	.004	.078	
	YeEx+NS	-.117*	.018	.000	-.154	-.080	
YeEx	Pep	.111*	.018	.000	.075	.148	
	Urea	.105*	.018	.000	.068	.142	
	NCl	.483*	.018	.000	.446	.520	
	NS	.350*	.018	.000	.313	.387	
	Pep+YeEx	.398*	.018	.000	.362	.435	
	Urea+Pep	.260*	.018	.000	.223	.296	
	YeEx+Urea	.410*	.018	.000	.373	.447	
	Pep+NCl	.433*	.018	.000	.396	.470	
	Urea+NCl	.396*	.018	.000	.359	.433	
	YeEx+NCl	.305*	.018	.000	.269	.342	
	Pep+NS	.226*	.018	.000	.189	.262	
	Urea+NS	.146*	.018	.000	.109	.183	
	YeEx+NS	-.012	.018	.522	-.049	.025	
NCl	Pep	-.371*	.018	.000	-.408	-.335	
	Urea	-.378*	.018	.000	-.414	-.341	
	YeEx	-.483*	.018	.000	-.520	-.446	
	NS	-.133*	.018	.000	-.170	-.096	
	Pep+YeEx	-.085*	.018	.000	-.121	-.048	
	Urea+Pep	-.223*	.018	.000	-.260	-.187	
	YeEx+Urea	-.073*	.018	.000	-.110	-.036	
	Pep+NCl	-.050*	.018	.009	-.087	-.013	
	Urea+NCl	-.087*	.018	.000	-.123	-.050	
	YeEx+NCl	-.178*	.018	.000	-.214	-.141	
	Pep+NS	-.257*	.018	.000	-.294	-.220	
	Urea+NS	-.337*	.018	.000	-.374	-.300	
	YeEx+NS	-.495*	.018	.000	-.531	-.458	
NS	Pep	-.238*	.018	.000	-.275	-.202	
	Urea	-.245*	.018	.000	-.281	-.208	
	YeEx	-.350*	.018	.000	-.387	-.313	
	NCl	.133*	.018	.000	.096	.170	
	Pep+YeEx	.049*	.018	.011	.012	.085	
	Urea+Pep	-.090*	.018	.000	-.127	-.054	
	YeEx+Urea	.060*	.018	.002	.023	.097	
	Pep+NCl	.083*	.018	.000	.046	.120	
	Urea+NCl	.046*	.018	.014	.010	.083	
	YeEx+NCl	-.045*	.018	.019	-.081	-.008	
	Pep+NS	-.124*	.018	.000	-.161	-.087	
	Urea+NS	-.204*	.018	.000	-.241	-.167	
	YeEx+NS	-.362*	.018	.000	-.398	-.325	
Pep+YeEx	Pep	-.287*	.018	.000	-.324	-.250	
	Urea	-.293*	.018	.000	-.330	-.256	
	YeEx	-.398*	.018	.000	-.435	-.362	
	NCl	.085*	.018	.000	.048	.121	
	NS	-.049*	.018	.011	-.085	-.012	
	Urea+Pep	-.139*	.018	.000	-.176	-.102	
	YeEx+Urea	.011	.018	.534	-.025	.048	
	Pep+NCl	.035	.018	.064	-.002	.071	
	Urea+NCl	-.002	.018	.906	-.039	.035	
	YeEx+NCl	-.093*	.018	.000	-.130	-.056	
	Pep+NS	-.173*	.018	.000	-.209	-.136	
	Urea+NS	-.252*	.018	.000	-.289	-.216	
	YeEx+NS	-.410*	.018	.000	-.447	-.373	
Urea+Pep	Pep	-.148*	.018	.000	-.185	-.111	
	Urea	-.154*	.018	.000	-.191	-.118	
	YeEx	-.260*	.018	.000	-.296	-.223	
	NCl	.223*	.018	.000	.187	.260	
	NS	.090*	.018	.000	.054	.127	
	Pep+YeEx	.139*	.018	.000	.102	.176	
	YeEx+Urea	.150*	.018	.000	.114	.187	
	Pep+NCl	.173*	.018	.000	.137	.210	
	Urea+NCl	.137*	.018	.000	.100	.173	
	YeEx+NCl	.046*	.018	.015	.009	.083	
	Pep+NS	-.034	.018	.071	-.071	.003	
	Urea+NS	-.114*	.018	.000	-.150	-.077	
	YeEx+NS	-.271*	.018	.000	-.308	-.235	
YeEx+Urea	Pep	-.298*	.018	.000	-.335	-.262	
	Urea	-.305*	.018	.000	-.341	-.268	
	YeEx	-.410*	.018	.000	-.447	-.373	
	NCl	.073*	.018	.000	.036	.110	
	NS	-.060*	.018	.002	-.097	-.023	
	Pep+YeEx	-.011	.018	.534	-.048	.025	
	Urea+Pep	-.150*	.018	.000	-.187	-.114	
	Pep+NCl	.023	.018	.212	-.014	.060	
	Urea+NCl	-.014	.018	.460	-.050	.023	
	YeEx+NCl	-.105*	.018	.000	-.141	-.068	
	Pep+NS	-.184*	.018	.000	-.221	-.147	
	Urea+NS	-.264*	.018	.000	-.301	-.227	
	YeEx+NS	-.422*	.018	.000	-.458	-.385	
Pep+NCl	Pep	-.322*	.018	.000	-.358	-.285	
	Urea	-.328*	.018	.000	-.365	-.291	
	YeEx	-.433*	.018	.000	-.470	-.396	
	NCl	.050*	.018	.009	.013	.087	
	NS	-.083*	.018	.000	-.120	-.046	
	Pep+YeEx	-.035	.018	.064	-.071	.002	
	Urea+Pep	-.173*	.018	.000	-.210	-.137	
	YeEx+Urea	-.023	.018	.212	-.060	.014	
	Urea+NCl	-.037*	.018	.050	-.074	-6.219E-5	
	YeEx+NCl	-.128*	.018	.000	-.164	-.091	
	Pep+NS	-.207*	.018	.000	-.244	-.171	
	Urea+NS	-.287*	.018	.000	-.324	-.250	
	YeEx+NS	-.445*	.018	.000	-.482	-.408	
Urea+NCl	Pep	-.285*	.018	.000	-.321	-.248	
	Urea	-.291*	.018	.000	-.328	-.254	
	YeEx	-.396*	.018	.000	-.433	-.359	
	NCl	.087*	.018	.000	.050	.123	
	NS	-.046*	.018	.014	-.083	-.010	
	Pep+YeEx	.002	.018	.906	-.035	.039	
	Urea+Pep	-.137*	.018	.000	-.173	-.100	
	YeEx+Urea	.014	.018	.460	-.023	.050	
	Pep+NCl	.037*	.018	.050	6.219E-5	.074	
	YeEx+NCl	-.091*	.018	.000	-.128	-.054	
	Pep+NS	-.170*	.018	.000	-.207	-.134	
	Urea+NS	-.250*	.018	.000	-.287	-.213	
	YeEx+NS	-.408*	.018	.000	-.445	-.371	
YeEx+NCl	Pep	-.194*	.018	.000	-.231	-.157	
	Urea	-.200*	.018	.000	-.237	-.163	
	YeEx	-.305*	.018	.000	-.342	-.269	
	NCl	.178*	.018	.000	.141	.214	
	NS	.045*	.018	.019	.008	.081	
	Pep+YeEx	.093*	.018	.000	.056	.130	
	Urea+Pep	-.046*	.018	.015	-.083	-.009	
	YeEx+Urea	.105*	.018	.000	.068	.141	
	Pep+NCl	.128*	.018	.000	.091	.164	
	Urea+NCl	.091*	.018	.000	.054	.128	
	Pep+NS	-.080*	.018	.000	-.116	-.043	
	Urea+NS	-.159*	.018	.000	-.196	-.123	
	YeEx+NS	-.317*	.018	.000	-.354	-.280	
Pep+NS	Pep	-.114*	.018	.000	-.151	-.077	
	Urea	-.121*	.018	.000	-.157	-.084	
	YeEx	-.226*	.018	.000	-.262	-.189	
	NCl	.257*	.018	.000	.220	.294	
	NS	.124*	.018	.000	.087	.161	
	Pep+YeEx	.173*	.018	.000	.136	.209	
	Urea+Pep	.034	.018	.071	-.003	.071	
	YeEx+Urea	.184*	.018	.000	.147	.221	
	Pep+NCl	.207*	.018	.000	.171	.244	
	Urea+NCl	.170*	.018	.000	.134	.207	
	YeEx+NCl	.080*	.018	.000	.043	.116	
	Urea+NS	-.080*	.018	.000	-.116	-.043	
	YeEx+NS	-.237*	.018	.000	-.274	-.201	
Urea+NS	Pep	-.035	.018	.065	-.071	.002	
	Urea	-.041*	.018	.030	-.078	-.004	
	YeEx	-.146*	.018	.000	-.183	-.109	
	NCl	.337*	.018	.000	.300	.374	
	NS	.204*	.018	.000	.167	.241	
	Pep+YeEx	.252*	.018	.000	.216	.289	
	Urea+Pep	.114*	.018	.000	.077	.150	
	YeEx+Urea	.264*	.018	.000	.227	.301	
	Pep+NCl	.287*	.018	.000	.250	.324	
	Urea+NCl	.250*	.018	.000	.213	.287	
	YeEx+NCl	.159*	.018	.000	.123	.196	
	Pep+NS	.080*	.018	.000	.043	.116	
	YeEx+NS	-.158*	.018	.000	-.195	-.121	
YeEx+NS	Pep	.123*	.018	.000	.087	.160	
	Urea	.117*	.018	.000	.080	.154	
	YeEx	.012	.018	.522	-.025	.049	
	NCl	.495*	.018	.000	.458	.531	
	NS	.362*	.018	.000	.325	.398	
	Pep+YeEx	.410*	.018	.000	.373	.447	
	Urea+Pep	.271*	.018	.000	.235	.308	
	YeEx+Urea	.422*	.018	.000	.385	.458	
	Pep+NCl	.445*	.018	.000	.408	.482	
	Urea+NCl	.408*	.018	.000	.371	.445	
	YeEx+NCl	.317*	.018	.000	.280	.354	
	Pep+NS	.237*	.018	.000	.201	.274	
	Urea+NS	.158*	.018	.000	.121	.195	

Based on estimated marginal means	
*. The mean difference is significant at the .05 level.	
b. Adjustment for multiple comparisons: Least Significant Difference (equivalent to no adjustments).	


Univariate Tests	
Dependent Variable:   yie  	
	Sum of Squares	df	Mean Square	F	Sig.	Partial Eta Squared	
Contrast	2.109	13	.162	160.483	.000	.974	
Error	.057	56	.001				

The F tests the effect of NitroS. This test is based on the linearly independent pairwise comparisons among the estimated marginal means.	


4. Species * NitroS	
Dependent Variable:   yie  	
Species	NitroS	Mean	Std. Error	95% Confidence Interval	
				Lower Bound	Upper Bound	
RK	Pep	.535	.018	.498	.571	
	Urea	.990	.018	.953	1.026	
	YeEx	.698	.018	.662	.735	
	NCl	.374	.018	.338	.411	
	NS	.379	.018	.342	.415	
	Pep+YeEx	.406	.018	.369	.443	
	Urea+Pep	.547	.018	.510	.584	
	YeEx+Urea	.352	.018	.315	.389	
	Pep+NCl	.312	.018	.276	.349	
	Urea+NCl	.492	.018	.455	.529	
	YeEx+NCl	.634	.018	.597	.670	
	Pep+NS	.406	.018	.369	.442	
	Urea+NS	.445	.018	.408	.481	
	YeEx+NS	1.219	.018	1.182	1.255	
RP	Pep	.888	.018	.851	.925	
	Urea	.446	.018	.409	.482	
	YeEx	.947	.018	.911	.984	
	NCl	.306	.018	.269	.342	
	NS	.567	.018	.531	.604	
	Pep+YeEx	.443	.018	.406	.480	
	Urea+Pep	.580	.018	.543	.616	
	YeEx+Urea	.474	.018	.437	.511	
	Pep+NCl	.467	.018	.431	.504	
	Urea+NCl	.361	.018	.325	.398	
	YeEx+NCl	.401	.018	.365	.438	
	Pep+NS	.789	.018	.752	.825	
	Urea+NS	.909	.018	.872	.946	
	YeEx+NS	.451	.018	.414	.487	


Post Hoc Tests


NitroS


Multiple Comparisons	
Dependent Variable:   yie  	
Tukey HSD  	
(I) NitroS	(J) NitroS	Mean Difference (I-J)	Std. Error	Sig.	95% Confidence Interval	
					Lower Bound	Upper Bound	
Pep	Urea	-.00633	.018356	1.000	-.07067	.05800	
	YeEx	-.11150*	.018356	.000	-.17583	-.04717	
	NCl	.37133*	.018356	.000	.30700	.43567	
	NS	.23833*	.018356	.000	.17400	.30267	
	Pep+YeEx	.28683*	.018356	.000	.22250	.35117	
	Urea+Pep	.14800*	.018356	.000	.08367	.21233	
	YeEx+Urea	.29833*	.018356	.000	.23400	.36267	
	Pep+NCl	.32150*	.018356	.000	.25717	.38583	
	Urea+NCl	.28467*	.018356	.000	.22033	.34900	
	YeEx+NCl	.19383*	.018356	.000	.12950	.25817	
	Pep+NS	.11417*	.018356	.000	.04983	.17850	
	Urea+NS	.03450	.018356	.828	-.02983	.09883	
	YeEx+NS	-.12333*	.018356	.000	-.18767	-.05900	
Urea	Pep	.00633	.018356	1.000	-.05800	.07067	
	YeEx	-.10517*	.018356	.000	-.16950	-.04083	
	NCl	.37767*	.018356	.000	.31333	.44200	
	NS	.24467*	.018356	.000	.18033	.30900	
	Pep+YeEx	.29317*	.018356	.000	.22883	.35750	
	Urea+Pep	.15433*	.018356	.000	.09000	.21867	
	YeEx+Urea	.30467*	.018356	.000	.24033	.36900	
	Pep+NCl	.32783*	.018356	.000	.26350	.39217	
	Urea+NCl	.29100*	.018356	.000	.22667	.35533	
	YeEx+NCl	.20017*	.018356	.000	.13583	.26450	
	Pep+NS	.12050*	.018356	.000	.05617	.18483	
	Urea+NS	.04083	.018356	.614	-.02350	.10517	
	YeEx+NS	-.11700*	.018356	.000	-.18133	-.05267	
YeEx	Pep	.11150*	.018356	.000	.04717	.17583	
	Urea	.10517*	.018356	.000	.04083	.16950	
	NCl	.48283*	.018356	.000	.41850	.54717	
	NS	.34983*	.018356	.000	.28550	.41417	
	Pep+YeEx	.39833*	.018356	.000	.33400	.46267	
	Urea+Pep	.25950*	.018356	.000	.19517	.32383	
	YeEx+Urea	.40983*	.018356	.000	.34550	.47417	
	Pep+NCl	.43300*	.018356	.000	.36867	.49733	
	Urea+NCl	.39617*	.018356	.000	.33183	.46050	
	YeEx+NCl	.30533*	.018356	.000	.24100	.36967	
	Pep+NS	.22567*	.018356	.000	.16133	.29000	
	Urea+NS	.14600*	.018356	.000	.08167	.21033	
	YeEx+NS	-.01183	.018356	1.000	-.07617	.05250	
NCl	Pep	-.37133*	.018356	.000	-.43567	-.30700	
	Urea	-.37767*	.018356	.000	-.44200	-.31333	
	YeEx	-.48283*	.018356	.000	-.54717	-.41850	
	NS	-.13300*	.018356	.000	-.19733	-.06867	
	Pep+YeEx	-.08450*	.018356	.002	-.14883	-.02017	
	Urea+Pep	-.22333*	.018356	.000	-.28767	-.15900	
	YeEx+Urea	-.07300*	.018356	.013	-.13733	-.00867	
	Pep+NCl	-.04983	.018356	.298	-.11417	.01450	
	Urea+NCl	-.08667*	.018356	.001	-.15100	-.02233	
	YeEx+NCl	-.17750*	.018356	.000	-.24183	-.11317	
	Pep+NS	-.25717*	.018356	.000	-.32150	-.19283	
	Urea+NS	-.33683*	.018356	.000	-.40117	-.27250	
	YeEx+NS	-.49467*	.018356	.000	-.55900	-.43033	
NS	Pep	-.23833*	.018356	.000	-.30267	-.17400	
	Urea	-.24467*	.018356	.000	-.30900	-.18033	
	YeEx	-.34983*	.018356	.000	-.41417	-.28550	
	NCl	.13300*	.018356	.000	.06867	.19733	
	Pep+YeEx	.04850	.018356	.339	-.01583	.11283	
	Urea+Pep	-.09033*	.018356	.001	-.15467	-.02600	
	YeEx+Urea	.06000	.018356	.091	-.00433	.12433	
	Pep+NCl	.08317*	.018356	.002	.01883	.14750	
	Urea+NCl	.04633	.018356	.411	-.01800	.11067	
	YeEx+NCl	-.04450	.018356	.477	-.10883	.01983	
	Pep+NS	-.12417*	.018356	.000	-.18850	-.05983	
	Urea+NS	-.20383*	.018356	.000	-.26817	-.13950	
	YeEx+NS	-.36167*	.018356	.000	-.42600	-.29733	
Pep+YeEx	Pep	-.28683*	.018356	.000	-.35117	-.22250	
	Urea	-.29317*	.018356	.000	-.35750	-.22883	
	YeEx	-.39833*	.018356	.000	-.46267	-.33400	
	NCl	.08450*	.018356	.002	.02017	.14883	
	NS	-.04850	.018356	.339	-.11283	.01583	
	Urea+Pep	-.13883*	.018356	.000	-.20317	-.07450	
	YeEx+Urea	.01150	.018356	1.000	-.05283	.07583	
	Pep+NCl	.03467	.018356	.824	-.02967	.09900	
	Urea+NCl	-.00217	.018356	1.000	-.06650	.06217	
	YeEx+NCl	-.09300*	.018356	.000	-.15733	-.02867	
	Pep+NS	-.17267*	.018356	.000	-.23700	-.10833	
	Urea+NS	-.25233*	.018356	.000	-.31667	-.18800	
	YeEx+NS	-.41017*	.018356	.000	-.47450	-.34583	
Urea+Pep	Pep	-.14800*	.018356	.000	-.21233	-.08367	
	Urea	-.15433*	.018356	.000	-.21867	-.09000	
	YeEx	-.25950*	.018356	.000	-.32383	-.19517	
	NCl	.22333*	.018356	.000	.15900	.28767	
	NS	.09033*	.018356	.001	.02600	.15467	
	Pep+YeEx	.13883*	.018356	.000	.07450	.20317	
	YeEx+Urea	.15033*	.018356	.000	.08600	.21467	
	Pep+NCl	.17350*	.018356	.000	.10917	.23783	
	Urea+NCl	.13667*	.018356	.000	.07233	.20100	
	YeEx+NCl	.04583	.018356	.428	-.01850	.11017	
	Pep+NS	-.03383	.018356	.847	-.09817	.03050	
	Urea+NS	-.11350*	.018356	.000	-.17783	-.04917	
	YeEx+NS	-.27133*	.018356	.000	-.33567	-.20700	
YeEx+Urea	Pep	-.29833*	.018356	.000	-.36267	-.23400	
	Urea	-.30467*	.018356	.000	-.36900	-.24033	
	YeEx	-.40983*	.018356	.000	-.47417	-.34550	
	NCl	.07300*	.018356	.013	.00867	.13733	
	NS	-.06000	.018356	.091	-.12433	.00433	
	Pep+YeEx	-.01150	.018356	1.000	-.07583	.05283	
	Urea+Pep	-.15033*	.018356	.000	-.21467	-.08600	
	Pep+NCl	.02317	.018356	.991	-.04117	.08750	
	Urea+NCl	-.01367	.018356	1.000	-.07800	.05067	
	YeEx+NCl	-.10450*	.018356	.000	-.16883	-.04017	
	Pep+NS	-.18417*	.018356	.000	-.24850	-.11983	
	Urea+NS	-.26383*	.018356	.000	-.32817	-.19950	
	YeEx+NS	-.42167*	.018356	.000	-.48600	-.35733	
Pep+NCl	Pep	-.32150*	.018356	.000	-.38583	-.25717	
	Urea	-.32783*	.018356	.000	-.39217	-.26350	
	YeEx	-.43300*	.018356	.000	-.49733	-.36867	
	NCl	.04983	.018356	.298	-.01450	.11417	
	NS	-.08317*	.018356	.002	-.14750	-.01883	
	Pep+YeEx	-.03467	.018356	.824	-.09900	.02967	
	Urea+Pep	-.17350*	.018356	.000	-.23783	-.10917	
	YeEx+Urea	-.02317	.018356	.991	-.08750	.04117	
	Urea+NCl	-.03683	.018356	.757	-.10117	.02750	
	YeEx+NCl	-.12767*	.018356	.000	-.19200	-.06333	
	Pep+NS	-.20733*	.018356	.000	-.27167	-.14300	
	Urea+NS	-.28700*	.018356	.000	-.35133	-.22267	
	YeEx+NS	-.44483*	.018356	.000	-.50917	-.38050	
Urea+NCl	Pep	-.28467*	.018356	.000	-.34900	-.22033	
	Urea	-.29100*	.018356	.000	-.35533	-.22667	
	YeEx	-.39617*	.018356	.000	-.46050	-.33183	
	NCl	.08667*	.018356	.001	.02233	.15100	
	NS	-.04633	.018356	.411	-.11067	.01800	
	Pep+YeEx	.00217	.018356	1.000	-.06217	.06650	
	Urea+Pep	-.13667*	.018356	.000	-.20100	-.07233	
	YeEx+Urea	.01367	.018356	1.000	-.05067	.07800	
	Pep+NCl	.03683	.018356	.757	-.02750	.10117	
	YeEx+NCl	-.09083*	.018356	.001	-.15517	-.02650	
	Pep+NS	-.17050*	.018356	.000	-.23483	-.10617	
	Urea+NS	-.25017*	.018356	.000	-.31450	-.18583	
	YeEx+NS	-.40800*	.018356	.000	-.47233	-.34367	
YeEx+NCl	Pep	-.19383*	.018356	.000	-.25817	-.12950	
	Urea	-.20017*	.018356	.000	-.26450	-.13583	
	YeEx	-.30533*	.018356	.000	-.36967	-.24100	
	NCl	.17750*	.018356	.000	.11317	.24183	
	NS	.04450	.018356	.477	-.01983	.10883	
	Pep+YeEx	.09300*	.018356	.000	.02867	.15733	
	Urea+Pep	-.04583	.018356	.428	-.11017	.01850	
	YeEx+Urea	.10450*	.018356	.000	.04017	.16883	
	Pep+NCl	.12767*	.018356	.000	.06333	.19200	
	Urea+NCl	.09083*	.018356	.001	.02650	.15517	
	Pep+NS	-.07967*	.018356	.004	-.14400	-.01533	
	Urea+NS	-.15933*	.018356	.000	-.22367	-.09500	
	YeEx+NS	-.31717*	.018356	.000	-.38150	-.25283	
Pep+NS	Pep	-.11417*	.018356	.000	-.17850	-.04983	
	Urea	-.12050*	.018356	.000	-.18483	-.05617	
	YeEx	-.22567*	.018356	.000	-.29000	-.16133	
	NCl	.25717*	.018356	.000	.19283	.32150	
	NS	.12417*	.018356	.000	.05983	.18850	
	Pep+YeEx	.17267*	.018356	.000	.10833	.23700	
	Urea+Pep	.03383	.018356	.847	-.03050	.09817	
	YeEx+Urea	.18417*	.018356	.000	.11983	.24850	
	Pep+NCl	.20733*	.018356	.000	.14300	.27167	
	Urea+NCl	.17050*	.018356	.000	.10617	.23483	
	YeEx+NCl	.07967*	.018356	.004	.01533	.14400	
	Urea+NS	-.07967*	.018356	.004	-.14400	-.01533	
	YeEx+NS	-.23750*	.018356	.000	-.30183	-.17317	
Urea+NS	Pep	-.03450	.018356	.828	-.09883	.02983	
	Urea	-.04083	.018356	.614	-.10517	.02350	
	YeEx	-.14600*	.018356	.000	-.21033	-.08167	
	NCl	.33683*	.018356	.000	.27250	.40117	
	NS	.20383*	.018356	.000	.13950	.26817	
	Pep+YeEx	.25233*	.018356	.000	.18800	.31667	
	Urea+Pep	.11350*	.018356	.000	.04917	.17783	
	YeEx+Urea	.26383*	.018356	.000	.19950	.32817	
	Pep+NCl	.28700*	.018356	.000	.22267	.35133	
	Urea+NCl	.25017*	.018356	.000	.18583	.31450	
	YeEx+NCl	.15933*	.018356	.000	.09500	.22367	
	Pep+NS	.07967*	.018356	.004	.01533	.14400	
	YeEx+NS	-.15783*	.018356	.000	-.22217	-.09350	
YeEx+NS	Pep	.12333*	.018356	.000	.05900	.18767	
	Urea	.11700*	.018356	.000	.05267	.18133	
	YeEx	.01183	.018356	1.000	-.05250	.07617	
	NCl	.49467*	.018356	.000	.43033	.55900	
	NS	.36167*	.018356	.000	.29733	.42600	
	Pep+YeEx	.41017*	.018356	.000	.34583	.47450	
	Urea+Pep	.27133*	.018356	.000	.20700	.33567	
	YeEx+Urea	.42167*	.018356	.000	.35733	.48600	
	Pep+NCl	.44483*	.018356	.000	.38050	.50917	
	Urea+NCl	.40800*	.018356	.000	.34367	.47233	
	YeEx+NCl	.31717*	.018356	.000	.25283	.38150	
	Pep+NS	.23750*	.018356	.000	.17317	.30183	
	Urea+NS	.15783*	.018356	.000	.09350	.22217	

Based on observed means.
 The error term is Mean Square(Error) = .001.	
*. The mean difference is significant at the .05 level.	


Homogeneous Subsets


yie	
Tukey HSDa,b  	
NitroS	N	Subset		
		1	2	3	4	5	6	7		
NCl	6	.34000								
Pep+NCl	6	.38983	.38983							
YeEx+Urea	6		.41300	.41300						
Pep+YeEx	6		.42450	.42450						
Urea+NCl	6		.42667	.42667						
NS	6			.47300	.47300					
YeEx+NCl	6				.51750	.51750				
Urea+Pep	6					.56333	.56333			
Pep+NS	6						.59717			
Urea+NS	6							.67683		
Pep	6							.71133		
Urea	6							.71767		
YeEx	6									
YeEx+NS	6									
Sig.		.298	.757	.091	.477	.428	.847	.614		


Profile Plots


£FY1áììl___úàsÏ=×ÚÚÿôÓO;99­]»V*²_ñ¿üÅÃÃyä·qãFæ-999>>>²½~ýºþqÒ[«V­bÞ¢ï5¯J¡¡¡bèÂëÖ­3øÉ£bs owqqY½z5I»t`tjÁÁÁÒ¸(ôÃeee$gÎãlÛ¶M&Yø¥393EgþK':Ú.cD"eËäVWW§¿qódoo/§F£a#)ìîîNñô®å§ù¦f~dwèwÞyÇBóÓ'  nüì.«)æ+ãüËËËÉiô#L(`ák&>!!^SSS)ÏçS8>>^ÿ#s$ë×¯§ý+V¬0o~$úé7mÚdáNûtÌÝ¤_:ÑÑNta HÈáôÀRLILLdcx<ÅkSÀü¦ÕÁ61·ÕÆÆFgffRxéÒ¥&uÄXq"""4MAA³K·mÚ½~ý:ÉLO/ÊÂb±Xÿ-üÃÂÂh777ÂL¬Ñ.%èâÍ«H$b;Ñ+Ã~dÒ£zÿý÷u:Ó|¥ÿíU¢úÐÇ£££§WS=3E7éNt´f.vïÞMo­[·N>ÓIæÏ9ÈU«V±1~~~SZZjð)3§ù¦l~t÷¥°··7Ý­ÓÓÓ&j/4V2!ý]æA!9ÁÕj5©¹sw'¥0ÿ²eËb·È9h·½½Ù5xÞj²sÅ$1d¢NNN6þ:óG%H¬µµÙ%2o~ìãT¦(Ø-/©¢ôK':Z3>îîîúÅÅ«««ùóbÎÞ*,,¤0½RÏøSæOóVÌ=½­fddXh~8bAAÁÓ@óècF&U¥=ö0MwôJá©iï2o~¥´¼(¦z:fnJ_ªcæÂÐkb	ENzù>|iIýÃÇ¨HMæ²ù1~ôÑGÌcMOOÏY4?øáÙÙÙÂL&LÃÉ ­níííª3ézHáiÕèÔÛü&*ÿ)éNÇLÑMéKcL^ú0ZiÐæG^~2ÌaÂÂBz¥SP©TÆ2sj`ÊæÇtçbú1S;99é¿Õ7Î´Íi*//×étãògôbÏ=___&%Ó±éiGæUI£Ñ0_M¯LGe3Ó"EÇ000@¾Âáp¦g~SúÒ)¢Ò×ÉCíÛ·3ýüã0GaÉåÇôdz"r¹aæÔ0?iu0ÓÏ-Á²cÇæ-v^flÁôÌõ$WWW¶oAþÆx	;;ðÖ¼*±bA¯S:*í^úW¯^==óÒNétÌÝ¾ÔÂCºº:®xîîîlLóæË~ª¦¦Æäa95Ì0eóëëëÛ·o£èNrFÊÅLk7=óÉdaaaNNNä9;?Aþ£ãÓæùùùØQ|BBþ0RâU«VM:f¸Ø|,<*9gddxÓÑ§g¦&åw©©~©å§c¦è¦ô¥^´mÜ¸Ñiú.ýÁ169yyQçn¢Ã0U`~0Ë0M£ãO]ç|||P23A§Ó5662CP ÀüÀ*`úùÑ­âïxzzb0?°úúúöîÝûÔSO14)pàÀËY¶læêÕ«ËÊÊPÀüÌÀüÌÀüfLLLÌ½÷PëùÙ?¼uëj0?ÌæóùÀü`~0?ÌæóùÀü`~ó|¸¸8ñÆáâââ   |cvv6ÃYºt©£££Ë¶mÛjjjP0?0Oæ·lÙ2©Tjùé§AJJÊ#èÛ!0?0OæGNd~Ä[ÈSO=Eìíí¥ÝÆÆFC1ë×¯G]Àü`~À|ßöíÛéU 4<6lÐVÇÆ~ÝÅÅeõêÕLzH´qãFçqÂÂÂêêêØl(½~D"¡JºùÁüù0?BáéééîîNiÛnG&çææ¦²e%SqttÜ¼ysff¦N§CÀü`~À¼ÒÓÓ)°k×.3æ7jô´Ùÿý÷Éá¶½Ý»wSÌºuëäãPv)I/ÉV­Z¥ßÃïwÞÑo0?0çæGlÚ´ÂS5?DÂÆ¸»»ëÇ466Ò®«««þ¦¥¥­_¿ÞÑÑù¸³³s~~>êæ7Mè÷×Dïfgg³lII§§']yO?ýtnn®ÉEb~tussóööV©TS2?ý¶Ï|EµF£ÉÈÈX³f%Xµjêæ7èÏDÃè"cÝ°°°¤¤$ÄÆÆÒ10?ÄüÔÔTÚ=pàÀÌO?C¦AEê¿+Ùôd©!ùMÃß¡C¢££Ùwéúc~¦¨Õj¦Ð8ÆÀü9,sm~Dhh(3bÃ¤í1oõcòãÌ0áuëÖ)ÆatDDD0ïîÚµvW¯^M7kÚíííÝ·oÅ<ýôÓ*ß®cãHúÙáççÇü¶`[¤õ[§MÆ_^^X æÁü¤Ré²eË&2?OOOf7<<ÜäÇëêêØ³cÛÛÛÙ1ÓÐ´´4HÌoÍ~v0Hõ¸°ï:99ÁÓ^çi/ÇÈüÒÓÓñÌì&?^SS³qãF§qÂÂÂôÇb±xûöí®®®ÌØùÍ¾ùÏBD?;ßOôJa10?Û3?ãwÃÃÃ)@¯ô£ÄdÌÀÌo¢ÝOOÏ¢¢"10?«6¿9æÌæóùÁü`~0?ÌæóùÁü`~0?ÌæóÌæØúMýï8888;;oÚ´I&¡XÌ°OócÃ:.===<<Å`~ÀüäÙÛ;f¾½Ç1?N·téR&¬P(BBB9Ùô|>ßÙÙ9((Ïa~0?`NèÐÉÚoò>íDæGÚ÷Î;ï0»R©¤zM¿cÇJL	P/0?`;7u=]Iæúúú·ÜÜÜJ¥òðð`Ó·¶¶R@­V»¸¸ a~0?ÀÌÏ)tpp0NïèèùÁü0?Mz¾··wt¼ÍÏÝÝóùö`~ 00M¿oß>$''ïÝ»óùö`~r¹Ãá8::úúúB6=ÇsrrZ¿~=Óø`~0?`Ñ"ùÁüùÁü+|Àü`~0?ÌæóùÀü`~À¬ßÔÆêbô.ùÁüÀüÌæÌ6ùpCËÌ7MsÌÀü`~¬'¿z½~yèÌ7ñ/Ã,4?ÄÇÇSX¡P8::r83	JJJüüü(ÒÝÝ=%%uóùÖS7k~@¥RQ8""B*RÏçs¹O~~>ÒÓÓ]\P0?`cæçààÀ¾¥Óé° ôðð°$óùÖ³³³þ.©£/sKô`ÕP.GEEûøøÀü`~0?ÀÚ			ìnMMÍÚµkÍÏäÃÜÈÈÈÌÌÌÆÆFÌææÒÌª´C¹ó¹õw¢ØvIqq1©©Åbqpppnn®±ùEDDH$ÀÀ@&ÒÙÙY$©Õj.óùÁüÀëB)/âòT·CÛ4>HRÞbØ+iiiO=õ££#½&&&þt¿×9¹áp(¯¯/ÛFéãããââóùÁüuwö^ôs(æóùæóù0?ù0?ù0?ù0?;ÔÃÙ§ÿú¯	ûs2~§ ®´	eÁÁÁ%%%ì.)ÆÅóùkA§Õ%ìþ*õOYY'oGo8WqãqÔ+gªZQ2@TVV®Y³Ý¥pyy¹yóC¡Áü`~Àzy#$ó#ÿcöeÕPJ6nÜÈ¬ÛOáI$æóùk&'¾¨4³×å|ñR@Ú"¿@Élì¬«é3ß(kjjüüü(àïï¯¿¯B¡		qttäp8Ôü(Yqq1c6mBÁü`~`È=÷vj£­ÌQÊûqN£d¶EdÎÁYûg¾Q>ÆoÛ¶m÷îÝ[¶lÑJ¥àóù5¿úùUVVêt:2HææóóMCEKÔ+gÈö§½ir?íü±XLWWW§éææÆT*k~fò!w		DÂü`~`Á¸u¡äïÌ;WqN'ï»ö×b6§(ZQ2ðÓÝÝHéô[õ,1¿ÒÒRGGG2E'ÌonÉd^^^ÆñEEE«V­¢«Ð××µN¯óôÓO3Zc`~öGó£v2¿¿¬/I¯TÝLºbs¼¹%)%Éôár¹kÖ¬FyÂüæ×â+)²råJ%%%Q 66éjc`~×¯_ï¶Ïõ¸[_=Ëî¥ÿpèåÓ5÷jQ2+g¡Ì/""B"P@ Nj~"(  @­Vûúú¢Ìoáp8B¡pÒqæÎÎÎ£ã½t:èÒd	cÌïÄùÀö9ûç/#_×¹|&óàK§']Gá¬2?¹æ¹;æ×ÌÐÐÐ¢¢"äææ®_¿ÂóïKVâââ]»vQ®`6	Çài¯]brõ¶Ã¡gäQ>_oooXXX__Ù>ªÉßâ1?¢½^~,ì<3ólÛü¤RéöíÛ[[ËÝÝ]­V?Û¥°Éß¢2?B)ïÛÎÏ/Òiu((ÀVÍ///oíÚµ2	OLL¤½ù-6óóþÁáä×Îq34jÊ°1ócv½¼¼º xxx888xzz2Qc`~VNÚ¡Ü3&ÿó[Àü¬Ycøæ9Úßÿú;[þ­P?`~0?`ÜºPz,ì<æy`~`QPqãñ!ÎiÑ½ÀÎoêKðù|6,8§§gll¬É4ægëÖ­[ºtéèøÌàÎÎÎÆ£+`~`©+mzåÌ£;OPû6?ývY«kmmõööNKKÓét$000>>æó7of¦IÆ¶mÛ`~ÀzhÉ¿u¡E°cóãñxÆæÇår?ýôS6¾ªªjÕªU0?ßtX¶l]4ÅÅÅ555ðññ¡ x°*z:úb·¦^)@Q¡KJ>0Û¥¿½°+í²V·bÅÆÆÆ|5óÚ/æ¢Y³fèW©¿ÌX	Ê¡sÜ´C¹áX@4ßggÌ|£|®°°0<<ÜÀêW4ùÁü¦ÉÒ¥Ké¢Q(LãýÎÈËËC°ZtZÉ/â2Y J`W7õ¿;ùÌÌù­[·íÛçáá1ú÷V@ôóÖ,W>Éß)èïDiìÏü$I@@~¯¯¯X,þéß NóùMÒ>BA»¶Â³ùn&Ý?þúE°3ó#"##y<³wï^ý999tïùÁüì0àA0zÃ¹æGí(J¥òóócc===¯_¿>:>°wùòåééé0?Ì,:Þ¬zå½¢(öd~Ï×)))ñ÷÷wttôòòJHHèSæ79t%-ù9ÛlñÍ8§K3«P`~ÀLæ¢/|fÁüµÑ^/?vþfÒVÒó¦µ/11Q£±ö	Ò`~ÀJyÜv~Ú¡ß899ÙJ0Ï räÿÑ7êÁa	/_Næ×ÛÛóvF­IÞwüòægfÞë?°+äÅnMU´ö¢(Àü~Å`l/°iòÎÞÞpòæ÷3&c­SUs,ì¼¬±E°ú%|>ß 	ôõõq¹77º;:tÅó³`~`ª<ºóäç´è^`æçïï¯R©ÍoóæÍ<C­V8Jæ7k(Ý»wÃüPWÚDòW^¢X¿ùÞEFFÁ¸¾¾>WWWÌoxyya`ÇÈ»£^9s;µEÚü¦­þÏÂ&þÿÌot|a-©Tj`~>>>QQQ(y0æçëëk<¼ÃÍÍ~OÀüÝ hí=þúÈ`VÐj:4ÃM3ß´có+,,70¿²²2ooo///.YZZZùMf&gLæááA>fè]»vÁü=1¨ß)¸òIùXéMýïGæGþ§ÃPRRêèèùMó:c.,R=ÅbNG¥KÂü¡QkøsöÝt²@ÀjÍO"KUUJæ7\Â*((8pàÀ¬.À.ÑiuW>É;þúLõ°Zó#"##y<³lÙ2îgÏ+¬°ØùíÝ»Ï¡ßÕ/00æì;_> ùSÊûQë4?JåççÇÆìÞ½û³Ï>H$Ì[±±±öìAÁü¦	ý°`Q,®6+<[EJÒ+Iþ0Ï3À:Í`ºÝ3aL¶nÝº¥KÒ=ÚÍÍíÃ?T«Õ(1ó³è^CÔ+gÄ?4£(Àü`~Àþiªj%ù+ËªAQX,æWYY¹råJ¦vÝÝÝÌ,ÚëåÑÎÝLº¢`ÿæm°nùERÞ·ú.3see%k~EEE^¶lÌ,C¼Ë×c0Ï36?v&gÖü1Xló¥ÊMÞwM=8Ò`æçîîNÇ´óù©ÕêûöQØ'ùyàzLAìÖÔ>;4¿¼¼¼%¦`VùEÈ­¥CÏ¶d(À¼ÞÔõºÝ;;;oÚ´I&µDÁÁÁ%%%ì.)ÆÑudæG´¶¶úùù1céR[¹re]]-ÌÌ7üÉ»Qù4?6¬ÓéÒÓÓÃÃÃg+óÊÊÊ5kÖ°».//·ð`½­óóIuAý±°óMU­(mC3ßUæeËÉÉiV<aãÆ¹¹¹ÈÏÏ§ðT?`~0?`ç´×Ë¿~ááÍZ@_Ý¾¶üæo¿,È0#[:néÒ¥LX¡P8::r8³éù|¾³³sPPñsacu«©©aVgõ÷÷lüDOtú¬¸¸1ÈM6áz°ó[2Ì8_XäÈ»Hþ0Ï3`>nê?Ú÷Î;ï0»R©tt|1_.Ë¦ß±c¥LNN¦±mÛ¶Ý»woÙ²E?r¢Ì'êçWYYHßKÉ|Øù9LfuaP9·õèMLõkócquu%!ëëûq777& R©<<<Øô­­c=RÔjµË¤ÆFÅbÚ5èÍ?QæfÜ1$$$22µfæçíí$Ë­ÿla~`¡Ð¨5)|=¦ESó³DÙrúéÛk&ÊÍ8~ÒÌ)--¥o$SD­ÙùÉd²§zJÿçÅÆ­sT/ÌXü¾IýSY 0ÏæÇ6é¤ïííoósww¶ùM¹Cår¹kÖ¬F­Ùù±°ûøøæ³¸bÅ@ó2x§¼çü×ó¹éúð|o&Ýß)èïDÕæÓü"""$	èîÈ¦ß·o÷îÝ;mó(óF$núúú¢í>999®®®á,§ã'ëNu.ñ§m¤MÓÜ±°§|;µìÄæEk/j0oæ'Ë9££#É;,Òóx<''§õë×3Óû2¨¿`hhhQQrssé«Qq6f~**??ßÇÇG¿j½½½ù|>ÌÌyäp¶üî<9v^üC3ª`¦`~÷ósssG??ó³æGíCÏ¢60?`ægÐ¥cÌoª´d$7£BÂLVø×ü03ùMYcWôswÓ*P§¬Úü&cÚ39Ëd2///ãøOOOÊöé§fÖ´$æó³rú»c·¦¦ÊÅ<Ï¬×üæf°ñ[aaaIIIeù³$æó³~C¼Ë)|©þ,.óãp8B¡Ð¤ù¹¹¹étc"jµi´$ÆÀü.^¼(6ÎãÓ_>þÕ&¡óêÇ»ÿ*ºÏ>NêQõ£ø·.Ø*ÀÛº©Õ5æèØ]ÎÎÎ6mÉd*ßl^»£?_X	[c`~çÏ¯¶LÍë|ôÿü0ø¿n/RXø¿ÍM»9;þá¯ýþî¨hì»1?6¬ÓéÒÓÓÃÃÃ!U0¿Y6?ý!#Ìp$Kbð´×èÏ»_¿<TÛÙÍ>íôôµìéo&ÝzåLó£vT7ÀzÌÏ ãl1Læ7ûæçîî®V«Gõ´$ægOÈöÇuÅ@ÇÞµnÁ#­¶[Yëö¢æÃµ$-¨qìÎÑþYØ:,4?ÄÇÇSX¡P8::r83	JJJüüü(î)))S2?N·téR&l2sJÏçóðæg©ù'&&R^ÃÂÂ,ùÙù)â¾3?îñÇÿó7ÏíT?n´?ó#D÷>úüAR¯­í­^a¹ù	J5:¾Ì.³Z.ÙËeøøøäççS ==ÝÅÅÅró#íçw]Sú;vPÊäädJú·IóÓùüL^a­§§'³Þ%10?BqëÉ¯^Q©§½Ý	W/NüOÿ®ë°¿mÉ¢7+Í¬B½Ûo¤nñ9üOó¹¡Ì×MÝÈüØ1½ÅÉHé¦i&ÖÞÕßuuu%ëëë39%kmm"g¬À6Ìoîæó;`~vôµÛÙö­óz¥ëÓÚÿóÅ¦çwª¾¯±¿íéè#ù»tõn7Ü^­Lêÿ9Ü4>HÊ|Qáìì¬¿KªÇÆèK¡Éf¹nrnµIûùÏ½êÌÏnùÙ$|õËCÿÀð?©ÊÇ¦Hèý2bÚvýÍþÿòþ¸íüìS¨÷ÅÌç§HþP`RBBBÂzÔÔÔ¬]»ÖØ½L¶ºDFFfff666ÎÄüLfNéG'èìÁüÅîÝ»a~`0ÉYÛ­nØWç¢Ì°·*Vãf`gÊLJqq1©©Åbqpp0»¾«EDDH$ÀÀ@&ÒÙÙY$q¹ÜÉÌ)ý¾û(¼wï^ÔÍ££#Öíh~¤âÜÐþ'MÜÎÿÑ7¼ËÊ!Ô>Ì3¤¥¥=õÔStS¦WfÈ£±«ÉårCi|Ù6ÂÌÌL¸¸¸Iç1ÀdæÇã999­_¿iü6l~TµÆÝ?ÝÜÜØÎ0?0oæGh´ôn­ÛÝ	W(lOgúnìÖT¥¼Ì³%KPöc~¤ðT£2ÌÃÃ$||>»víùù7?Õ÷5Ïn¼¼éh7ÜN-Þp®½^kæÌ,ù±c¼Iõ( u:ØIa~`þÍot¼ñ¯+æRí/(±=5þ=¼Y8ôlSU+.EÂz`ÿ×ï?wò7Ò)JØ(XáÃ®ÌÏÅÅ<¯  @(RàÀL³º5?áÆçv6<»uàVÝ~ó£v¿êzvOqÃÝÏ¾ôÅþ>Cc7juZ`!ÍoïÞ½ìxý®~ì0üÈù¦ ÝSÝKüi³<½øaÿG`WHãó;ícð¬±+zÃ¹ôJ»²muÌ¯oÕå3Oð¯'$¢di~Ddd¤««+(@èççggó³B·:òæa×ðÏÿ!ú?Ötóìdn[¥¼?vkjN|®"$/*÷Ðèx?¿?¢°ýÑ:ÞË(À­óý9wëÚÍ´/Ê!^Äåë1¨Y[A'JåZ¸Ý8µá»¸´	¢çù&òÒÕç@ÊóZ¶?®Îã%ûöE§Õ	æÐym¶âKYGÌoÕWÿò¦ ~lt`ü±çrÎm+;ü[Úè­?8bí¤9èo(óEEpppII	»Ka1',Ë;elæg`oæm¡ú¾FüË°ÆçvUÙÃ8+äãf¨Q³6Êf¨¸áî±G×ñ^8ñì¾k¿®Ê|Ô^Ãôó»ý[ôóRYY¹fÍvÂåååS2¶iÊÌoQ]XÂÇ±½ÀÊÑhåK¬óöE¥¶õÓ¹u¡4n;ó<Ûýüw¯¼M¶·æãì?g×d)U?­pÀí=zâo?O#y`l/0ÏÆÛòóó)<UcêÁüù1­µ?lùc4Í÷ÿqÃàr[?²¬caçåÍÝ¨V+§IÑZvñMÁNÿãÏ¼º%¡(^Øþh"¥P¤$n¾qdEg7úþÄ,lAÎ555Ì Kvñ4B¡PÐ-ÃáPxR?£dÅÅÅAnÚ´iÚæGøøxooï¡¤¤"ÝÝÝSRRpmØù1kxØÄÙÂüÀDôÿºÎã¥Öí¶>ò£º ä¯¡¢ujmiîä²÷rpügÿù;Q~ó§½(CBR i¸1<óò1Î|Û¶m»wïÞ²e~dDDT:68Ïçr¹¬MÔÏ¯²²200P§ÓA2¶ù	J5Ñ1øøø==ÝÅÅ×ßòåË©^mbf0¶³Ì¯Î+¤?ç®MHHv8ôì£;OP§ÖÛ¼pâÙüí×¿9]ÐVªÒv6awîßNõSÌ*XÅbº/×ÕÕéGº¹¹10MrÇÈÈH3iÆù°ýé-ÒG3Ç`Þ 5D"¡ª¢Ãúåæ&Eq«Îã¥æOÓÜa»gÑ^/?vþnZ*tAÐoÞó?þiÉ_g'½yM>Õíc;¿=üÛi|6TÇ"½»j3iÞ´JKKæ:óù;;;ëïê±1ú)M÷ññùÙù-í6¶[ÙöÖáÚ_u'Ý³èïÛÎÏ=:oH¤ü7R·<sÌð¯d~äd3ÉSÝÓ&JåÆÝ@æ÷0þw=uÅ(g0=ó3ù Õ¼iq¹Ü5kÖDGGOúÁý>555k×®5Niò"##333a~¶a~±½À¦QÝjÓÓ¾¨Ï¾©þæ»ûM÷OÜ:ÿ	ß¾k¯V¦ÎJæ:º·µùæg¾xÌ¯·¡¬"æÕ^ñ;ùEDDH$vyU3¦%ÈÉÔjµ¯¯ï¤ýüIõHÝFÇ53ãR<gggú.ú"MmóSbD£íú4EôÏÛî´/:­.yßµsÜÈßìBnGGG¶GÎGæGþgIóÞq!ÐÂ-%ë/§OCÇ~sø9;ÿÅzËs ócËåÇÑÑLm33Â#44´¨hlqHr¸õë×OúiiiO=õåO¯&Ää1dffúøø¸¸¸ÄÅÅÁü`~0?°ð¨ë$ÛÅ¿¼÷ÐFåïzL/âò rµ9´:-éÝç§Â/¼NÂ÷FêÁþT÷èS>ÿiªÛÃþd~Óø m¨8ÀìÛýüÀb ÷bví/:ÞûL×7`ÇwöÞñ×/(ZQSeH3]u kPÜàø"sZØ¼7sZn«ýòã^»ÿÑ­£¯ýQÊhXócò¡X$hÚä-[>¶Ýi_î¦UDo8×^Á!í)æ½º%àÄ³ôJáÙê½g!]57+Onø!úÅîÇyc~YG³¡v`~¶ÌÌÒ¾:ZÂÿ¤í´½¥2*n<>Ä9-º×z4	32ç`pü´ÈÚ?óÁ¹Ó¹Æ¤5Â¢T®²©çâýOCScæGåþÎÃ¤·´ª>Ôæóó®o@öÑäÝg3lîàµ=ûðf-ê®ÁL¶üFêÏOåâù]!­©KÛ_ÍÛÚ-º3:Ö³pô¬®ØK·¯Å¼ýïßdó½lUXHó¨³þÜß0?`OôçÝoxæ÷àuÄ¶¼E$zåLIzåb®>¦y$YKí@Ö~?×RÔJYCÖWÛK:ÍÉEÈµ#ìÓÞ¯Ëû?ÎÀózÀÓ«Ïxxf×æù;cD£ÿ-QôÏwBa:rEkï±°ópgr;2<ò¼Õ1¿f÷*¤åHZU_Káùòë$¹'û»ôßºtOSÕOkòÇÍ,pû¹vüéÒü4ÍÒ¥KCCCÕÛè588øé§ÝÌ]§æìµ°Aòòg~¯*ÙÐa*÷]K;«Óêì¾Är1I©Þó±«æ½ve5X "æUcçcH½§ü,Wñ_v.þs¦Í¯H·#	æ&¾©ëµ¿8;;oÚ´I&Íbæ|>ß D"ãäääéék2°ó[¹r%Õ«ZýÓ<·³víZdûÂüÝÓûen­Û¶5íF­IùàkçY<</8þu¼Éüæm6KP6?:·³Vðþ ìñ»ÂVõÉÝ;Ïwl;ÛFaöioJq/ÅãÏ36¬ÓéÒÓÓÃÃÃg1sýe|Ù¯kmmõööNKK£/H$ñññ0?»5?æçFóÓ>º`~`ñ iîhÙòqýòÐ[e6tØW>Éß)èï´Z`Ï8ñì³¸ÚlÑ×üPx!¢æìöÞÃëdX;òÝ£?d´Ý¨P+ì/»@æwê»î·/vtèð·,1?''§ÙÊvy<~÷6ËýôÓOÙøªªªU«VÁüìÖü<<<¨^CCCûúÆæ W3yñ´,6·ê¼BZ·GÚÐ´/ygïØ"o¶ÕÆ$ýÅsçy²å)¡îi«¿òç§6¶Ý½8¤Ò.»µµHÓnÕ¿¾:1GTþ¨iPñÓ[·h¸üùÁ²Ã¿M¿]¯èhÑÿ%þ$­ýe¿Û÷ýÆo|ÿWÓétK.eÂ"$$ÄÑÑÃáPMÏçó½°Ëø²	V¬XÁ,Úk7¿ììlcsssÀ®]»`~`Q1¢Rwüñdí/zÎm+Çü G½áHfCåÜ®lck<»zA&[¶­ªOzW°¹úôr¸ùÜð÷hô¨åªÖo]Cii_\Ü;ï¼ÃìFDD0ÆFªÇ¼¤ô;vì ÉÉÉÀó+,,d ³	Ì<ÜùÙ¡ùdåÊô£éRJaaê~aXÏÙÂüÀ|2xïá_½Þüoï7´ØÄî5=+þ¡ÙÊ³BZÎ×°ÙX&E§l)<_»ÌÂíuÊ¦ò±)ÿ÷£ÓùqüÛ¥%÷xÛ×¹w%Þ2ÀÌ¿ÄÅuS×ÃÕÕdyG°ÏßT*¾µµut¼q&,úGæGþó[Ôæg+ÀüÀ<36íËóu/ÑëJmýÌÌóPomÆ.K¶~áuf¸Vgí3étÕÜ|÷øê_Ø§®ZÝ£7ÞJé¸tO)é²hlMZ©òíø%Rèàà`ÞØÞL¶ù7÷èÇøúúÅ?Í®Óé222`~vk~^^^ÞÞÞUUU0?L[KUSÐn[ù!kì:vþÖRk8ýÅs÷]ÛkÃ5&B!,xt~·(;ÐúéÐ])ë#áÛ¹óppX;byn0?0sócôÒ33²©ÕjwwwÍäñxlÌÞ½õGxäääøûûÃüìÖüèW­Ô+Ì, =ç¿ýÃóïfýÊ¡¸íüë12ÕÁâ¹'n·Îá¡l*^ ­_ZÃÄÔËO×½ý{|~÷ôÖ^ù_DDÓK ²é÷íÛGääd²7ËÍO¥Rùùù±1×¯_Ø»|ùòôôtÝ3ã£>R(:UO7ó¦MÞ²åã:¯þk_<C=8|ÁÿèÌÏ76)øï^yY]Â¶Ò¼Ç2Ð^76t7þw]U7FtZ¥JW(<Þù_UÙ?ÙX`~`ææ'Ë9£££¯¯¯P(dÓóx<''§õë×3gÎçóõcJJJüýý)//¯K	ØªùM´n/Ûæ>½_æÖy¼Ôþ'Ak>NV'8söÝôAå¶·ÝFåY]#Wømg§ÍÕ©VÕ×óYùumÅtêÁzÙðé[=ÛÏµËQT·¨õ[N#¯É7Ñ:þâÀôLÀü&Äa¬gg°:QèV¶½u¸ÖíÅî+V¾àïõØ­©Jyÿ,æ©TáçÅ­Ùxný[Çíl´*uukQÊÃ¸×èu W~K8ÖÈ·#©=¥¸W¦4Q³åMCf>7ü¹eó³!`~Àª`¦iÜ>TUoÍÇYY½áÜçyÖê´ÒòØÛ'ÿ#å÷Áñ/ì»¶÷ëªLR@Û­¾¶£ìêÃøß5å|&mi½tO¹ý{ä5yQÝÔFo°Ìdóûùozb÷îÝ0?&±ñi_jÝ^öÅÿÞ¬zåLó£ö©~°³¿ójeú_ÿquÌ¯_=»îØÍ£wÄ64"çWæT%l]úÃO²ºv$µ¹Ý#íÖà,óóòòbFø¢Ó@-lh|nç_½>xÇzçÝ­+m:Ä9ýèÎIS2ÍI%á^>võ»WÞ<à·+íd±ÖÇ5g·W_øïooì¹$?¶ÀnÿÖÒ,óóõõ5ÞáææÆÎóÀº®Ôþ"¨í­ÃÚn¥uaHv,ì|YVÉwªÞ;âBf²e¦÷ÞÍe¶Þ¼§ÏB*¾ú	o¤Ûz¶íäîê5®[À¢3?'''R=LæááA>f¤·õ,×ó¶ÂØ´/áªóxÉj§QÊûc·¦êÏó«ý¼ðÔþöÏF¤ý×Òd±õ2ÜßUÿõÑïòNÿïÔ¶hä,^ócWô#Õ£X,ÖétXºt)Ìi@ÚgÍÓ¾*âßü2îÀcyG]ýêÙuGò>±Þ¦]|°GøÍçw?ÛpêóO3Êf8¹µ²§¡«³z>7üA-®ºÑXÝ9½KÙòùü¾H$q8'''OOÏØØØ¹>0ßæçââBuYPP )pàÀ&Y]6Ö9í´G*xÀ#uËÿ²/ü£ï%4Éìµ4mÉõäâ£¡'î´µõÌÎèzáW¥ÇçsÃ_Ìo¾Èßß_¥RQkk«··wZZN§H$ñññ0?»2¿½÷²ã9ô»ú±ÃÀüÌ´/MA»Õu:f-µ·3k©EædÖRÓiuirçzçAÞ§ÍúæfÞ§[®úÃíR5LÑÒ&½û¨òþ"ì	Y×@¬æå3?æg¼Ç4NÀårõ×ð­ªªZµjÌÏ®Ìot|åfWWWQ,ÐÏÏÏÏæl¶ó OôÏÏó´/ÒéÕÊô×öxöÔ-gy&×RË=7vkª¢µ×>J»ºE>ýÎõOwåþàqµ='ùÙ[ÿøÍï¸×g¾Q>âãã½½½GÇg^		qttäp8fÐ½"ÝÝÝSRR&5?zJ¥	V¬XÑØØh>6?[æl¡ªúyöE«ÓÞoºÿyá©ð¯Ç¿p kvMÖ¤½÷n§=kÓò×?¤ËxÐuþnÖgoßÛÖQû½µ!ÌXzS7k~y>ÁÏçr¹Lüü|¤§§»¸¸Xb~ááá	ÌôõùÁü`~LvÚ]ßÀlj¥fåÔ-d~Ò©ùåÃµÇÂÎOcç§¶8>¿ÏéòoþTûº¬"gDgóiÃüÀ´Í[ÞÒé~ äææÆH=<<Ìä³Äú	ÈüÈÿ`~ÂüLÆ¬ÏäG¿Hèzúé§FÇ¦===ÜÜ10?`O0Ó¾Ô/ù´/Òéb©^Àg÷]Ûµ2&-î5D½rÐ&qX;Â,°ûßÉOò¼øÍí%Úz¸ùqvvÖß%Õccô­ËäÍZ.GEEÉÑ­vÒþì®D"	ÐñõõÅúÇó³+ósYÛK¿Q®ôêååE°°°¤¤$ÄÆÆnÚ´ÉdÌØ½_æNoÚf¸FdÎAf¸Æ[Çá³rTÍÚIþî|ùÀ®­GÃ,°èj[É7üÊØÒ[<kv>!!!B¡Ý­©©Y»v­±u?Ìï´I7YËÍot¼¯?ÇccöîÝ«?Â#''ÇßßægWæçííM²E¿æúXé~	ôÊôQ%dÚ®Õj5ãÆ1æGFXísçÜë÷Ô¸¬ù~_tá­ó3ò2¢/y-áßÿùØ3âC£ÒQÌÕë7ÿ¶>!ùo­°Ä.dÿðÇõOKÿÊt#ílé%ÿ]÷µõ×õ½»W(//=q¿øË;wnáâ·uæôFY\LªÇ´Åâàà`öñ¾uEDDH$c3v"gggHDwO.;%óS©T~~~l»§§çõë×GÇö._¾<==ægæ'Ébbbzê)V]]]7nÜXWW7GÇZRRB×%½ÒÅ=úóÎLØ8ÆÀü®]»Ö	½ÐSXçVÿ/ÿÙ^ZiðVKË7ýòÿúÂ©5¸ò^vyEÎõ!57´ÄlIáÿ5»£½ÃHÜÜùUqÇ%·îå·]ÿ^VWWñùïj.ìiyTbU8»ºüìãê´?|^ûèjÕv	®|f®ïëiiitk¦; ½&&&t5¹áp(¯¯/ÛFéãããââ7©¢$`ÖîÒ¿_ûûûSþ^^^			ØùéË>y].¬®X±~IÌî±ÒeD¿KéU ßÐÉÉÉdöûÆ`ÚveÛÕÊôYû]Í×ÿµÔÔÃÉû®ÑF,fôÆösíå*6õ4R¹5g·+Êm¥rûGg4Aöio¤ ±>=`!ÍOWW×9áaÜçîî®V«g»6óï³>ùÏàßqÿù7ÇüIûHþªfåÊ'yñ;ó/ýCº¬Êþ=dÈ¾.ï)µCék­JØ,¯´Ò¡»Ñ&-µQ@Úø]uùÐjªÄã,¤ù©Tªüü|f(~ç?ýfN@@@ee%ÊËËî¢áááLS6½ùE6u¼?/<u3îÓêÿõÛ¶·k»Öp×cb·¦*åýóóuÒnMrQïösí×äÇØîïjÈ:Rój[ñ%Ûr>ÖæBiãM±èJÕqðè(°ægÜÏÏÍÍÄk.úù1=D"Óo^)L1EEE&c`~ÀÎËÅ$yÌl,ov^­L×_]ö¥Îã¥9óÙrJÒ+o¯Ã`ÃÚ¢º±)ZÞ¾Øq¥¬OÞ7fxZU_KáùÊØôJa[¬heOÃãçTÈµI§½ê¿ÀA¾yÛ;s`~ÀFÒÝ2ÍúçN(¿«óiÙò±¶³Á¾º >ê3âg=g¼K÷o¥t|!'ùcØÑie2+b^mÈ:2¤ÚkµC²¶ïbÑeo£XtYüø²äÉ·5§Û¤wk*xýíø,¤ùÍÛLÎ0?°ØÐlÙÌâ¹&Ñõ´¿üO±ð×|SUëáÐ³7ÏM¶¨å(v$µùµõh~rÁÊªÍµ÷mÑùT]ÒÆÕå	MâlÕà#@GtFø*ËNPüÐP7þ.c~1ë39ÃüÀb@²å ¸53®1xï¡ø7H^Þ3ÜÐ²°ç%kìÞp®$½r&(U?ÞøîÑíþôVSùãwE©ºËB'yCzG§6Ñ-39	.))aw)L1æ$`ÅÙ¦,Ktî×ÏG$q8'''OOÏØØXi-óV´GJ÷¦`ç3Ç|Ã/¼þyá)ò?ílJÑhåGÎ×þ"¨ûlÆÂ£RÞ·z:ëÎ5t'õîHjÏïvk~æÊi­àýªÍ]57m«ÒGFt=øñeQu¼£B§p4ÌXHeeå5kØ]7¶Ù%KüýýU*qÎ­­­ÞÞÞiii:N"ÆÇÇÃü`~0?°a÷NÜ:ÿ	³x®åÏs§ZØÐðìÖg~?xïá²zpøì»éWÞÔ¨5¤gØ¼&ÿ@ñ ¯H÷³ÜzÚ¡»²¶5tW£µO2GÚ×£¨#4æ,gãÆÌºùùùÔØ¦­z»</22Ò8ËÕ_Ì­ªªjÕªU0?Ì,"ÉÉóÈöfñ(â¾ªu±ó oD¥^¨Ó×iuirÏq3ÌË¼O+¸?6zã³°Õðh¡»ã^³¹¡»Ì´ÌU?ÄIäX>Væg<V4ÏÂöÐð·bMMßèøòúkø*GGGCáIÝ1ËeAnÚ´iRó|YdU?Á+ÕäfÑ;Ìæ¬­NKzûäÆsëIøÞHÝ"xÀ£æ½I¤³[ºa_ýòÐö%ûTáÙwÓû»j´¼iè¬®÷ø²K÷2¥aKÞNÛV|º;ÜßeC×y¤!·¦×")ê@ý1]5Y1[vqæÛ¶mÛ½÷-[ô##""-ãóùu¯úùUVVêt:2HÖçÌ_aaaxx¸A3þa~0?°74CLó^ÀgÙX.W¤uöw.ø)3nÕy¼Ôº=rç|Î/ÝÊÊ_ÿ.ãA	i_YãØ<ÌÆÎÇÝrí¯64téÌW÷èKR7YF38L`~`JÅb*éuÝÜÜJ¥òðð°Ä½ÈCBBá4E62?ò?ÌæÒ)Þ»WÞ&áûß'$VµVi­¬#9ù_ïÅì:»iQ¯).Æçw¿l8EËÏTµ©¼æìvÛºKÎ×ÕYMÆV[sQ!ÄàhiºU/üjª[EéqÚ¦ñAÚð÷¸HïîFRerÚ5óîUZZJÞ¦?nc¢üÙ]D ãëëK&Ê¦Ôét0?4?Ìç>C¡â»'nõìºçcW[OóypÚfôÆ¢|¸ötFV3³1ýÒ¾ªÍ½âû¶r1hûÛ[îó5Ô]3èÌG.Ø&½;þ6a~...$ÓËå®Y³&::Úró#"##y<³wï^ý999Ìú«0?Û3?ý©ûLæó&E£à?"í¿9æû)¿?vó(ùßü×-F4ZÙG_Ôy¼4oÓ¾H»5gn÷¼<6EKmûpCEKÔ+gÝyb(ÓiCÖr>ye­Ýèk<ÉÀÑ«êÅ°óH$ºH$P«Õ¾¾¾öócP©T~~~lLcc£§§çõë×GÇö._¾<==ægæÇM·yóæÞÞ±sôJ1ùùù0?`¯Üoº$ïu¼WÇüú¯ÿHò×®l³é3ªªo|nçNû¢ÕÕMÑò_v£z@Yc×ñ×/°ó<kUM9UÄ¼ÚRx^§´þÒÑ)ê_1³1kµCøÖf~r¹Ãá8::É±c~Íð [yQQrss×¯_?¥¯ãóùú1%%%þþþôÕ^^^			fØù¹¹¹Qåi4?õÎ¡Ãv ùû`@=pG(8þçÖÛbóÞ¤t'ýElÜìNû"ïÓ¦*ß¾ØqòFwuéòþØ­©7Nßi-J!çÞâÙÄt-#:MWgµ°2Qø0ÎÌ6o~ÌO²=óC??`	äO$RSÝ|ÿmÓø mSmkR4^(M~S°óùØÕLóX.¶ãÑ4wH_û@üfeÁ_ò<²=r>2?ò?ó[ï_?¹1òÂÛÇ;%Ö_PZí33_½ð«>¥ËÅb~îîîäyaaa´Û××·nÝ:¡xïDyêÆß4>H%kà2«kÈÚ·fÓùÏÉ­±i_¼BH§7íKÿîFõÀ|Yä5yQÝ v²°ºbfènXp0gÒy¡¡nÉe±ô:¤êÂ_1`q_~~¾Éeee0?0GÀÇ¿0ëÙvöw^(M~#uKÀgwð·)æ	Û-ÚB&çk÷èØÈ+#KX0£7Þ¾ØA¯ì¶¨ñUwÛ©°Ã8®½yöÝôA¥Õ©vR"]©~ß&½;¬Vâ/°Íot|ÒÈ+V8;;;88,]ºtÕªUíííVx¶0?I~h.Ê=ôêÙuAqk"s.¶æ=óôçÝoxv«äå=æ3Éµ#ÞuOøê_ª6wUÝ0º3éþñ×/(Z­bx,33_í£KÂÊDyGp»ùÙ0?Kgçå4îÕ=«c~ÍL¶«ÕÚÈÔ!óí=*uçAÞØÈ¾0ùÑÖ£¹tOùfr»ÑhUÜÌª»fîeÕDo8'kÇ©$y­÷Iøê_uw	1óù1?vñ=úøÇÙþº*Óú'[¶ªêÝ*þeF¾ÀnÆ>KùFÝ^"¨ÝháÐÝêú¨WÎ4Uµ.Àùu77æU?o¬ÏÂÌïgTVV®réÒ¥Ìx^www@óVb~äv¹Âoáá^§ÏâyîôÑh»®þ×w·úAâÒ>?­e­`#:­ìAæÃ¸×ÄWÿ¢îÂðêÃ¡g+n<·Ó$Ï#Û#çkm.$ÿC½[º©ë-£åìì¼iÓ&L6óù|ÿjúú¸£££··÷¡CPöl~ÌLÎú+¶1áØØX#~wø·§PIS+«M*I|S°suÌ¯#Òþ+µì¢ÏÆ2ÔËÏÜîÙó°xíDÿ×Kýy.§Ö+¾_°¹ÿÖéHõÊvç¹òÚ]w°^ø°2±£õ>:ó5?6¬ÓéÒÓÓÃÃÃg1sýe|Ù¯Û¼y3ÇcföU«Õê°[óóðð º¯¬¬dÍ¯¨¨ÂË-ùYìmÓù'æ¯ÿñgâõß%,n¸$ïJÀIø×³ÿ+ü½÷fjxlÝý;¤wæTõ3vÇ¦ñx©í­ÃæG~ê	/DR¹Ê¦òCOGßñ×/äÄé´³ßÓ$OÞQAÂWûèfcvc~NNN³íÞEFF'0X¯µ¯¯ÏÕÕÕa·æÇ.öÂýÎÀº½`.PªzâÖ¤]»ÿ´GúêÙu_WeRàrE3½~^xbPG¦Ô¹Ý³#©ýÔwcìSgwÇÕº½hrÁ_µRÖìaÜk]57gå`Cñ;irgQþÕÊ6éÝêñbÑtæó¤!Wò$g¶®F·ã¥K2aBB·fCa6=Ïwvv2~.lrqÞv_6OTTªu±33ÓÎGæ§V«÷íÛGa///]Èùdíý?¿ªÖª·/Güï£ÿïê_s¯îÉ®É²df0)dV¥OTÑÙc£7RÍ¯½1p«¬áß7ínhùñãã«îóµfwÕ]r>2¿ä×f>Õßªë§ÙÑÌ;Ý]Â®ÎêoÏD®FÚ÷Î;ï0»±êq¹ý;(err2%°ÄüÙ'Èl²²2oooºïSÎ¥¥¥¨b6¿¼¼<39ÓÅó³Ë¼O®V¦S 2ç/Où%'á_coôWx;[0ì¾ÜþIV×ÃfGo¨Ô²ýqu/uâ·&WÄ¼ÚZ2»Î§ON|QìÖÔþîiæ¯ìm¬~UõCô.:ó»©ëáêêJ2××÷ã z777& R©<<<Øô­­ccçÕjµq&,úGæÇÜâÔ°¤¤$&&&44ÔÑÑ155Õa·æGÐuãççÇíuvv^¹re]]-ÌÏÖI*I<vóèèxßÚÏ;:Þíü%3s)ZÞ¾ØqéRÒ5åÓFtÚöo/>øèÅª79Êâ¹>ÚôÊS¦4ÕßÈN!46ãaÒXg>ìÒü,B¦kAzã>Z&ÛüD`æëªªª¬ð¹Mó³`~¶yÿñgî7Ýgö¨Þì<që8JfÚôé²*ûÿ ÑvK88½tÌª»uiûUò¦®Kµ¿9¯ëÓ##<Ä9mÉTá~Y	Xt¥GQJÓüØ&=ô½½cdÔjµ»»»æGDFFòx<6fÙ²e:ÝÏþ°ýþ¯ööö+W._¾æfÒ¾ ¸5/ñ") díÇ£ÞéQÛ>ßýVJGJq/§ç|­E©ÜÇ)ïöáe¸¡Eºa_ýòÐÁ;åszu¥MQ¯Ýk(j°SÚxóaYl8 ¯³ùEDDH$cÃA`` ~ß¾HNNÞ»w¯åæ§R©üüüØÝ»wöÙgLþôVllì=PÈüè§Æö¹cH3ô§ëþËÉ@ÌÏ7ÈðnT¼ÖùöÅË×Þ0Qi]Úþ³ÛÂSò­¼üÉ_Û®¿i»sw:-"Éyûû¤u×j*x­ÍÍ êÀüär9Ã¡[³¯¯¯P(dÓóx<''§õë×3gÎçóÙL¶nÝ:¦ÓÛ~H&ê°7ó[¾|ù³lXùYaæëö.BÚz4)Å½o&MÑRúD5í©QÈùkeìÆöÁÙWmgwëöÈ:¯¾ës8ÞKÖØ½áÙ£cÝ5]ÕÂI¢êÎ:Ý0êé"ùÒØØè0Û]TÒ¾´´4ù-8dxEu×ää|î)eÊé?×ªú¤·x1¯¶·|èîàrñ?n¼¼GÓ&£sTÊûO½qéÊÑ´Ggjk.ö(Deóc!Ï³Â»0? É#Õ+¥ãã9Éß°vdÚYé4j²=r¾¦ÏÈÿ¦úñ¶ã'Ç¦¹DáÙ=MõPo¤ ¢$!vûé/^E>°Wf²ÂX¼ægB±÷nÜ	òº9åíPÖØ6º9Ú«²ï"eæa>õ]÷¤öä¢ÞiLÑò³Òiå9U	²)f´2ºNÒ¸½áÙ­3èoo¬Ïª~ÏÌÌ§QkR>øú7C=¼0æçåååèèhÐÏÏ`ØÌæqÛHhÊc~Óø`hTe·ZÀÌÃüVJÇûi?-°;zÅ÷ÉùD©Ü¾æ³u]¦Ôy¼$ÿ[âLÿ½âÇòWÞ<þúE+Vs¹1?___ãánnnì¼á0?`±¿éþT·«qÑÍKÆiÒØá²Õ-ê7º·mÏ7±ÀîtÔª©¯æìvÌúÑj;ÿí½g·ª¾¯ÒGtR=QuJ½ð«.ÙÃ:óÝLº½áÜæy¥899êÉd2ð1#½wíÚór k¿ÏáêµÌo¤MÚ#µÒëÒÝ¨x/ÛsI6)ZôaîV%lWæÌå½³kÔ¶ëoÌù¬îoÞ­©àÍÌ×ß>iúôÊ¨WÎ4?jÇÌ²ù±+úêQ@,ët:XáüÝ0?+D1¨ êÖ[¦9yc¤Í>f~®m>s»gGÒØ»åMC³2ªa¸¿«!ëHEÌ«mÅFæ¥´ÝJ2¿:¯²À	MTÕ%iÈ­.O6ÞVOajÀêúCÓu¥Mø+è¦n4VwFïR¶|>ßäõõõq¹77GGGooïC¡^ìÍü\¾B!8À03;Æzìº¹O|X;rK8x ½ó­±vÛzfgñY­ª¯¥ð|eìFzÆÐÝ2p«¬áßK_ûÀ`äÇølÌ_×TðdmßkµÓyFßTÕõÊ²¬üÉß¬¿¿¿J¥2þ¢Í7óx<fì¿Z­þpT]ßÞ½Ùñú]ýØÅa`~æ7sHòHõ¶k¼66EËlM]ÂÝ÷ÚÌîÎè0Tê?|ü?£ûjdD×£Õ>ºT/üJÙÛ8ÃùZD2¿;_>À_ùÍbÎÌâÆ	Úúúú]Q5ve~£ã+73õZTTD²@???+<[ý_nÍHLÞ¨Zc÷gJWÞ4ôIV×¤ö3·¤Ý³yÊ]57¡»²'Öp²åÕ¢ßù·'?úûfMC:ú¢7Ë=8Àzxpüå²Ã¿ùFùXh~÷ööy-$$ÃáPIPRRB÷ntwwOIIÔüè5  @*$ðññBÛ¹ùÙ0? ³oäìã7F3ýíha½hÿ.ãAßK²÷ø²Õ³2zº+¼1Cw§ÁÐPwscÞ£Ê3­Í§Sj$ÛgÉÈKÏWÞ¿Sv(W³~-ES7k~y>ÁÏçr¹¬±åççS ==ÝxýUæWXXn ¬¬üÒËËrÎÌÌ,--E½Àü`~`º¨GN~7ZÚ0rküio÷àÈéÛ£¥vvµíÃñùcS´¼Ñ]Ý2Ë«)¤µ÷çaè®ô)%õYÂI­÷ÙÎ|Ã-÷Ô/íÏµ:V°û+ÚCøKÇüØ¹ué-îÇnnn?þOU©<<<Ìä³Äú	ÈüÈÿ¿º¤¤$&&&44ÔÑÑ155Ucoæ·råJfnÌäær¾ÌQý~~ò1ù³ôGo¤*å³½ÊYO3tWö sÁodD×Ý%üÿÛ;¨¦²tß÷Z.×åõÙµ¯n=»n·Ï×Õm¯mÛÕm×­î¶k°²,§¶,m»´,ËRq¦-K-'T'DD&AEfA@çyaH!2ùíx¨TLCCàÿ[±vNÎÙ9ßÞûÛM´Gu!=Ý5æ<Ln|ão¬Í'¬µà/¿©n[ïybü1IÉÌ3_øÌ«Õú-f6è5Çsrr"&·páÂÔ?lkk[¶lvèÕÕÕo¾ù&ÞIe~o½õáHæöëëBr­¶¢]Wy¹@~jëØK¶þuðw2û¬>C5u·ÊýÓqºk|2ªþ.v!­ÊA	_².íËKº´/­u±×Ó.¯»ËkçãL>V®£Ñôkkk?øàSu3íÌÕ>´çèèËd2Í7?íó±þ>>>ú-³gÏÖ)&`7ß+A$¼ßyyyFïôægóä14©uZ*æçªè£¹ ¹¥ÍiÒv	µjý6ÄðçÛ#Î/´îìWV«8y!®kZââq^Ó¢¿ÏnÏª)÷jkN"uó¤0~¾®í£ÖZð7=¨èÒjßÖj6þ¤Àd»GæåÕ#êFêcÅÉÉÉ¦®fooßÖ¦ûkÕ'â9s&NËåùÉd²¥Kê·ìÚµËÅÅj<åæævàÀ¼5ÊüæÎk8zæFnnV7ÐÛ+WjBu.H¶ÄVhn>ÕØ]È¿~Ýï"»ÓiRÊúÂJ¥k©öÚÒsnÓµPPùjÊ<8¹eæÓÈäÜãîôÿ3Ï9àUüÕSRú½ÛôüüUIFXXØOúÓéÓ§~~~ºÇ³³³#û,^¼X#]¸pá9sÜÝÝ_Æhjí.ªÎårW­Z5kÖ¬iÓ¦C8vì±I¼/ÊüÈû½oß>`¢/óÉ#zw+Ms9iÀóã|=bmY&¼T7ù÷v:Õ¬L ÕØaky­ïvúýCã;uW?Vé§Ì§~Õ fuóO_5ÿ÷g¤òê§Ç(mwúøNMFþª0¿Aþ±0û`´Pªu>m÷°CÖºÚ&ÍÃ"ÍÕdM@¶9ÝÁ£±Àî Èx­¨3Õ^[Âq|óUúé²1÷µ¼b6f#ø^ÖJûBåyÎ¼_¿*ÌÏ,X`ÙÄö¸:Y¼õlì»©ÙÖ]`wpöµ&¹Tylê©NÇ©»ùÊ<tùd£5²Ði_<±ÛÖû	·²ÆèUwisÚÆ´uÍr¾òòòÿÛÂü¦¨ùÂêº£Ëu8ÝÁÆpöµØÝÔÝL?â|ìjå¸umKDlbÔ`>¯H¥aowTñ_éå¤Â~÷íî7yeÚ.ñK1[çpØü5kßë¯¿ÀfÌÏ±.A`x©æFªæaµºõìÞì=j¹´³ ´ÒmkRÚ7>ïÁ`>^We8,·^¾ñ¿WLû"*ÄøØÉÿÖ>üÀD0¿ÔÔTb~Ô0£iûöí3fÌ?>yQíóÉ%óæÍ>ú¢E¨ë¦[`~0?3J£­aij4Þ?tÐFuÝAé¥eTylj=*é%ìäÕ£óù³î`¾!NÊµJÚ©W7ô²Gq²LüTÜ2Ò¢hÈÖ6Zp )¸]¬l~?«Ïð8sæZ­&ÚG­9½aÃRqssÛ¸qã [Ì/--M&éõÄüF¥e¾DSÞ¦)×$/ñ´NÓÒ­+9B(UF©v«ÅäáhÿöfYÝÝ¯êvõ5ËåJXm5e-ÖðPId]ÇnÑÿýÏÝüUrÅí$ÞÎ¾¼î.·µgÎ×ñ%§é×#-ÒÊ¼$wÌØ¼ùM«ÏðX´hQuuµá¹sçREr¹ZÆtùw§¡19!±#-ÇóñHJ¹ç['¼üb<ùIó3:Ü/ìé±ÀÊxµ¨ñ	KËêkëgÉAWb¢£ÆþýJLxáSVàá>Ñ>NI·|K®+ÿåZR±¸ß÷O¯¸â6q~/VN2··ÉÁ¨~©¯X±¢  @ÿÔÉÆn,=xðÀhUD"ßÎÄ,Xpþüy¸ÍßA>4...3gÎ$já³nAoïDï·­ 7EkÁ@.¥z tP¾æFª*¦Ä¸ö5JìmK¾Yíµ¥»"~qL=¢¶ÂÇâlÌcÏ9àÓ¾T=kpúøNcQëùúiñª|¤/§²²rùòåú¤>üüKË<oÐvÞ~ûmÃA_ú·lÙâãã£T*© Ì±çàùíó.]JmÑ?;cÆA·Àüáô)Ã³ýÜkËÜe.Ou=Â±ÚNGLÔþÌ³Âunê®|¬çh4ê^^]C]­ÒTÔjM¼S¯öJõW[=þ¿JÓ_§Ê+Ñö+ñ78ih­f3JÛ_½.B¸~ýzjZZ©¿ÔØ,V=£äÚÑÑÑt£(H$zíµ×ð°IóÓä³q~¯¿þº¾NÈjMòzÖtÌXá÷ù»ÌÞàKµíÈ2]²è |M]ËäYÑÕJ9++83áêØ¯ºK$¯»«ÀÑ×Û88,æÓ¾ðÚù×ÝMõÍW«ÆïwçË´ý¥Ñ:ó+èÐ¶ðµüEN|÷GúØ¿z!íµ¬¼ýöÛúÙ½½½+W®$ßvvv¤þRó#»åååQi:n~Ðe|-[ÖÑÑa´ÃÂðOóÓä³q~ööö÷ïß×>fS+LoÞ¼ZüÜ°aÃ [`~þË¹ßÂ,e¤Y17¥F2äÚÝÁÏ-P#æòhÔªîxjê®Û<ÆïTÒEeæ#?%âNÛ¶öWKû"äÝ·?xp*q,Rý"Wi;´=ÒÞ^Z[Ù©íâ¶gÛ¶m»víúüóÏ¾C)-ðàÁ¡Cô~6Ô8?ê«V­VÔûÜðæE¾v())Y°`Áo¾I^466¶¨¨o­ßåóùeíÚµD(É3Ôÿ1o¼ñ±Ìyóæåääºæ7Ñiáë´PXp )r³åY¾À.e	ÕÔì`rÄôT§Tm¡Ù;jÇøÝöµ0êÃó±Û³Æ&óØð*i_óñÝ)yO7<.6óÃ8?±B[ÊÁmÁ ß/ä;w.UÉdärP3¸ãÊ+úp5E;Äüÿ¶àêêºzõjò%NqiÇnggç/ùËùóçO´ßæ7yµÄ¶&¾Rlµv©î`já8ÏvK:ü«îîWyc¹ÛÀ`¾Úàr/.§xOà°ðwÉ¹ÇÝéÿþgsF9²kKäïÞ·qn[ï,Ï3ù£_©+D×úúW2f	ucª4õjztÆÓ-ïFbxùÚÂqÙmqÉuU^¡ ÛY­êÓæµãÌm15¼ùQ4Yï 1?B[[Û²eËi¹ººÚ4ó°yóËåd#Öí¶e~¾vcx£²Àn·È°;Xg|ýRn3#êLû§Ü²Ø±t>"yDõê*ï4ÔôõÒ5êI> ¿ºù§¯ÿû3Yqíp®&Z÷÷¢Æ%_IsÛ$¬`X)µÜ,(-Ó/[ÄØXÎùÁçè¼cù?G4ð*<É(Uhý	¦ßKkTyÅ=¬]<ÚgÚÜæ7gÎsv3äÐ¡CË/¿|ù²ùæGpttôññÑo=¶QÃY³fá²Ió?þeÐÌ]&"¦5ÒÄîÉh·à@Røc§«aÉÏÇõl¿Ûy7K0ªì~Aú~PàÝÍ­gòÈ¬öÐ+7VwåÔ]¥RÊnÏª.ugÐ#DÂ6[ýé]MúpaÜvqPí¹«)kdwÓUÉõºQtÄÏL]­²s@Ôê¿5ÒBkß3×L';V~ë@ûäµôFÚó/äùê¼§s ·W¦TUJêîà¾,0?û¶6Ý_zhh(5V~xó£ÓéË-Ëå/6sL&[ºt©~Ë®]»\&O¹¹¹8poMÉ¤frèCÇíùRÔ,+¸DvpÓÚ-9°H¨ïÀ%/ü:°óhnÝ±¾©:<Ê¯­dzïw6§;Ø*H%]­j0©õ¯MÜ¤ACkzWcö(Wý F¡5½«~W#n§É1Mjß»ªËÞ|²åWÄñ#KûRÝìôñÖ×IÂÑ³ú#TyÅÚüvM;ÕðÿpßÇ³³³>:19ýßafx¬^½.¼víÚ½Üô[¸ªU«fÍEÜ`îÜ¹Ç£RpÛ3?=£1æ§6ËK·|´SWq5©8ß­§|Rûó§¦îV¸®i9'ïû~À¾iwp·Èº¯«ÀA¨)÷êdå`0ß ®¦­©2¯fÚª­iÓÏ¶1­é]( Õ~÷ V­éÒ¾¼¹²sÿ¥i,juY?óoò ²³å/ßr¿»êÌO¥Ñj¬áOó³!`~SÜü2u|¥ø_¡ºvIE¦Äh¼Ê¤j¯-ôûº«ïöÌñì`ÓÞÏ¾~WÔÃ(¦Ý¡ùu×çª¼,ÍPÔéµLWÔÕô¡µçÝ CÖ~p5¹jb~¨º´/Á	æÏü`Ñ¹çí¼"+­u*eW¿8]ÐíÜÝ¶Õðÿº+øGù]'8Í ¶§Û+ê½CvÀ0ÎæGrµÏGÌ9súôé¦¹ô`~ÀjôHkÓØ:ócöé4þö».É½¤>^yy­åõ÷ö;ßð½4®öYæAæFªææ3MT6»Y[Ñifï§²É)K©-ñj.$ª«ÜÒEþdÔS(e°4»ñ_Ú?9¬h1·×Î¿þYP²w®Å/ª7ùA=¬ÆïÙ¿ aÏ-¹´X7w@KÄó¸mëdµTyÅÄÙ¿RÈªp§ùmÙ²ç§V«lÛ¶æ¬qRNmGg~¤NG6äbxEÍ²Ë	=_v2ëé$Ða?¥ù¥tûj·M=ñ÷~Ös5	5¿ÍdÍÝÓz-G8¥õ÷óÛ©Õ¥î­[ÏÆ<6hªî³>?~¯Ç5ÄÌàTØïñUhØùd3ùP«x2Ñ"pDæ8M¿îjy¿¯ë´D©R©M?Ù_Tá¬Ê+ìaíRÊx§ãl~³gÏ&W[[K*.lkk#²æ¬OªÓ#úÞ^¢Dµ¹BÝÚ»¨-¥fWëþôjû~b£×xbÁ  ¦@èýìoll¸På¶WðXÓ+±Nï'Õü6h²h°A¨­ðédå÷Ëzð©²rzËoþÑò»­¤bÖ[ñ<ÏóP|oëæzïô°v°Åaü×ñOòP.-&OV?drq7?ýT åËµ&Ìh¬ÛóºK7¿&·Kg~D­è<TsYÝéíÂìmî÷Ù:rsµºîºé]-2X`i¨nênºOëVV©Ö5ù>Y´Úý)/*VàM+õéé¬ÙÇRÅ÷nûa×aµHbüÝû6ÎÇ>ë*ZÅü ~çÑ®÷Y?ã¶­ãwã_%óL ó5kñ¼ÞÞ^*ø×ÑÑ%ó2.É½QÑº¬._ßåäñµùíDÔd]ÒÜ¾Û·cºé"¥äyhmª©rbÄùZÑùô©êçtäÖVø4U²sÔAyºA´ÍV<uPrx¬Í'?_'Í.)júû%Ù¡ç®_üÄÿ.§é×½ì½º²2Zl¼·aºA³º¦ûÆ²ªÀºæ·jÕ*ýØ>j)@ó£#a¥µäõUçéz:á±lnJËw'³¯¥[1¾g¨Q«¨©»-ñÎý½£¾Ö5¯¦Ì£­9I"2XÔD©Ö6tiÒë5Þ:,ÓudøqRnã+9.)Û_H|¨ìõÃëb®ÐöZ?t;§ú>t^ëÃkç×b=o¬ªëáí·ß6mÑ¾Þ^Ýââ¤¾bÅ	øÛÂül¤jq:[[ÕUÝIÌYÙ+Ìj?À¯®Ì1öTºQû¨Ï¾	ÛMñT6fâÃí*kKÇ:¼®I¨&R8ÚÉ¢'*¾³óBãOÿÚyÊºÂiú5Ø#òg4b¯,viµ¯Õó<×%¯÷0QÌÏVùÙ:9§um°:µÖNKg7µH¶øpÆýÄÄµDøj·[ËGõ45¿ÖDD«ôëbx0_·HÕ¨yø<GLH¡6¡åð¹ÀÇÛúIíÍ¶Èí7kÉöaj,j=ýÞíºìfæóÆ´²åu]f¸ÖQ-¤NÏ|PC*ÏÙ;Üéæ·@ÍÂý½-ñÎÕ^[xIõ(&+&Çëª Â×PÒÓ]Cð+µÍÝ:ôÍÖÜ|ªë.a,zòBØ#¥Ë±§ìñýÉFòF©â]ð«ÿ·ÿáEöeE>ø]'G´´Ó~LÀ:æg4wø0?0ÎÞÙ:ÃµÎwLÔäBMÝUËGÑr!§#·¦ÌA	Û¬ÿ¢~mU&©f ;8¶B[ÃÒÊSähØÓØ"ìQi_ïl·zÙsyÝÝÜ°£íäµHûcYpÀüß$Ûáßyúrþ§»£uëQ¨Æz/q>jênGºÏ¨NÝØmÍIU%näçKóY.¡.^ª¹üDGêÚ¶ÉpøÀY-<þ5üø½î³>Ã¤òÄn[ïG4ÕÀü`~À,x"ÕkÄüt)ÇVû¨©»UîêÔ]F-ìki¢=ª­ðátäOf>µFÛÄÕ=34WuK:/´Í%ÝFØ3«Íö®öO7Í_-ÍrBTØï»?¹T¿Ìæ,äêÍÂ»£ÇøEjQSw¥ÜæQz	ZÙÓ]C«ô£Uù[a0µÈuÉ¢©@àÍ§FKLLô½¦â$°g&Âð§TÚ_8¸B«ÔaçÝ¶ÞOùaÌØù	[ËðÑìGoê.¹ºÔ½öhTóYµf ;8(_s)QÌ k¼	#FcÃø½>°'?ÀYWE$aowlû¡ 8a¨b¯§]ÿ,¨ÔÌoÓÂ· 9mkî¦=Ñ&¶É¹ý½¡GGuên?ÿÁ|6´Ì.µvpBµná`bAùÚ¦1ª]T`Ou¥þ?ô9öòëöÌAËøùº¶öÁù=ó/<oç|@¾R4÷ÌL$xÒÖÌ5»Y¸æëÔóTò>NK¼sënYì(9HØÆ GÔyp:rr¡¿ß¯¬³@RF­;ø<Økú5»ñT`Ïê¸RÕÖ§áÇïñMûRDsù(®È·«%E1¢¸]Bn¤GQ÷ÕÌox`~`¨gôl;õáèØ§MRõgMRSw«Ü?%?Gcê®F£îé®i¨¡Uúñº*ÆgÇèÑ-ÒdÐºïæ¼zw°RÑ*DR=VÃÏº+£Ük1²r:óí-¿Û:hÚ³y_ýÎXÜ1Ö1¿i/cúôé0?ðR:øÊÜüâÍøûÑ´OOæ|°#æKñçr%#jaéÀj¥¼«(¼üÆªxgØú¯J¥ÀÑDÄï¡M	£ÕL,øßHº©ÉB+Ø#¥µcâöÌïÑ0÷Cî)OLn¸½UÜSóìÔ¾óQÃi¥fS©Ã¿¿aÁM`ó³E`~4þFOöHËÇÇóùYp )à]·|zvÇ¦Ð£âZë[BÌjË¨*qcÐ#æíÈ®qÖ>®ãDìõ¦sx­ïíj¿Z^bôê/É#G­2ËþïÄïMZÛæó³y25Q13OR¹T±no,³«¿¡Cò©C²ù-búUK­º[w÷+"Vÿ©Á|Õ¥î66c´-0$_s9Aá÷HòøZOÞÚNúÛ£ueÜékû!gç£´/Ra¿ÇW¡GbÌÉóóÀü`~S÷eñiRqõ/ùÛöðíß&º?îýÏ9ÄÖV°[Y-Ë+ãµ2¢ÎÆÔ]FM¯ö8ÍOà°F½îæuÊ[òäu`º.YtPþd];X-ó#þÇ÷òÄ½lUº[½÷ÿc7vë7Z<ïí¸yø*øà`~ÀVIÉfºF*Õ=&äì¯O&ç#æçr·xÿ¹§kvGoq?~-hbx½¤ºÛ#Qí£¦îßXeõUw5jewWYmOu`·<²ïé½!JµnÉ¡»'²âÚæÿþìÖ»ýÁu,î@Ú«xIfÎ²³×Ü<Â¼½/»rî¨sIâ#£ÝXÅñui÷£ü|ïuåÔU²39ÐïºÌ]¿ôªÊ¨e.«ÚoZw¼_Àåçk¢=öµLò	C@öú¸Ø3?¡E¿:¥Ë¦ÿøÜßï-úôBÑgNTyò§£a¿Ø»æ¬~¾ä|t!þçN¯¼BTÒÞ=_´ÑÉt·an2@´Ä;ËXÜ+kÂ/îÝ¾/`ýÞû3©Nù¡ñ´ìâV@eé*ÀT6æÊä§DÜ9¥ÞÁAä¡Fì©5³ÿQkOøUã^ÊúÀ7ÞùIàíÜ&ÿ|äGyîüG×Oúê·òf¨ßÿ	ò#í÷_>jçüf¨ï¼~?	ñ3Üç¥7ÌØ<	>W]X=¢Cúój|¶6°ë©-R²ª¾;%y/ºöøµ¬íß&­Ú¹Å!þèLÐÊØ§Måu]=üt8øºr/V[R!"×ß½¿ä`ÝÁ2ÛKSÐÞx^NÊ±vþæ¶÷ÙÚjËîÔ÷OûHÏ ²?¦tsxìïý2?RYéóOBýæÜ.0?0µÌOÀ(¬ØUë»TßS¥Òttô£]H[¿7æ¯_ÛI¥&<Ëk¥&¨Õ¾^zCm0<ÜZ<CÔ§µÃÊ=ÐwßHµéî`U7µùã¿6èÓ¾´T°>¾SÜ `ªYyÊXÇÆï¸¥ÝéLÌO¨à¯Øé|ÙwÌæóIg#5u[«VÊ--âyµ¼¤ÌfÐÊsîy;¿¿zÛ· çFr²ÏýÄØ§æm%Döuîb®è ÿ¤³ùô«âNSÔ'¶Ùî`ZQòÖw%§eFnay××ãô³2wïç ËÕ±=ºÙ¿ª¦~ÂÜ0?Ìïâæs®k:B­5uW*éb6Å×y´4Æµµ·V°c6DT?Ëk%²8¢ü2ZÜ/Íò_°¡[·mwl$OMô³7í®aM¨@ <*GPº÷ÉKÜ.Z+z²KB»!#o[w°MÍgnl¹t6òZñ×Ó¡~Aì>OÌOÄR'ï|µwÌæóûÁùÚyçkMr±Öª»»¹!ª¦Ü«]¨TîTh81?âÄ#$^Hì8"1EwÂùRÎõrºZÞ×öZþÌï<*DêÍGCuGQiÇ¾;x(É#Þ^Vé×ÏHPpJTâNµæûÿúä¼¼÷~äæ»þTÑe¯?½ËéèÍ½çs÷ý£õèÎKÄüV5¦Æ,Ý;æóùi5j¯2©ÒmK¼³¼óê¯¢Ñ¨ûzjë«¹%C9ß0ôðeåu]ThðèÌMÜúM"QCÐÊ¤Ìf"cÜS¬Ëëø'»ñÆoX;D=riñ$[<ã¹Ø¾ØYfõî`Y¯¦¦jËPÖÞ=s>Þ"^òtG©¤­âìÎøëõÃWó¡;&´ÝË¸s¶xáÜÁd·K±û¯mÿnùÍ£__$æwiýc»New$àùÁülø4³wáÊ¹«ñâøÉiÇàîÔFGç¨o®øöö×Ôáï¾Üo4je·ª®òNKcÏ°bf>¹BÕÊ<Ëk­#'c&uýÞhÐ²H^î?ò1?ßy´«åý>LôD¥ìB5b5¬WLI^K¢:`@òáB*ä!1?âd²é±Ý2v7%¾=è6í»o?%ªwµúÑ¾4NT°Z¡~aª²½ù§¯ïlïH-?¹üæÏ5Gvézw%ÿéò7neá¾ùÁü¦ù:xì_×å¼æÔiÿo_=w5îøÑ[Ç¿q==ÑÙ«à¥-o~ò~»=«¦ÌÙ?fùô¡AÐÊ£W2·~¸jg$ùIå± 4¨VõÉÄO<×îöMDõt38ü)úë&a`Ï^6;Ø·RÅH¼'»$G6ÿJò´Ï»nÏ¥²Ãý/9i¿âtÙVºcbÇýR^&±Àÿï¡Tõ¸Dþß·ÿðÍ¼`o]NÄüþW¼ïéõ/¯ôÂùÁü¦ÂÖò*MJi++T¤ÜæjÏÊ¯ÔSûìÕWÝJºÚ©´*ÿaóThÊ/c4Í/3 /¦S&¶GOuEn3Æ¥ZVÞ-|X/¹§tJæ;æÒÐläQ/(É#þG,p(É#(Ôr¶ÝÖâé÷`á*H/Û,ª3éOÈç^Ë»;~ýí·Ïi¢¾±÷½Ù0?ßÔé×UN*íiÞe×?ªt[ÏÊð«p]óªBÙ×Â G4ÔOðÌ|=|a~ÏÄþõÐû»áâµ'$ì_9yWÛ[G3²-£kmjN.GÍºÐGò(É«ñ±uÖ©ot6(°OÎ£õÆ·ù6_±Ó>ÅñM¤N¶íäY«Ý²ö¸Üê%x|lÇy*«KxJÙ`~ÌÏf¹[Xpc¤¥,tWÉHJ³``ò¼îzÍ=Ý2»æÄ_fwÐuÒè·rSïÇTzzª®ÄW 7<òäQ/?Þpí`ÏñÓÒº'qÍ~×k)^°psÕÞ°ÏìÎxÓQzC!S«Z¤"Rh¢¾t*álf@#)×5Ôk¶Wä²®$í½ë®GÞ¿ñÙé Ýº£´ýÊ:¿__B&gÌæg³Ôré¹-¹fgÅ!qq§rZrrC÷ó#[RÇ;ß)¥L©w±kÊ½Zcúe=ùú(­A¤ ÛÙüuÒRO¿ú¨Á	á<ÓHÞË%Ïbr´¾Ò4NÒ;².éÁùVÏþËqúø¬Âú V®y1ë)];N+¥tmsYæùOHù]öãùÏ"HòðGñ¤ü[b0yè35ãrjîÕ4ª]K/½AJ¥KVÍlRê]sÝòHiqË/¿ô´ôHÍáIð¶;ç>t­;Ôìv!IèUf~ÁMó¶F­j;Þðà_OBo¬ì,­òØ¤_×¤.Vkzm§#W!NÄßñy`OÌ÷×özX;ù½Ê:iC4P<s/yN¼0ÇéR¶|>7ÇIâvP16ç¦*JÚöTço/Û^ìþiÞOswþ3ç£­Ù¹ñ¯»¦üßIW¦Åß#õ¶%i1î'V¸'r¯'f¤%d¸éVÝ%­QÍ>d5SÁ¼â>õºÝòÞµ¶õíGKÌ,­ßÓ÷ç¤n½wù½ÛÌý¹ôÃùMGÌoÜ70?`ÛáKÜIÌ¯9æ¤³ÑÌ£DÂ¶Æ8Z?¯«B¥Xó[M½á¯ÑbÃa?É³¢¸¢5ÂÈÍÂ°ügºÜê¼¿ùJ2ítQ%lû¨0¶ýWF4±4âjT¤6RÞÉI ÏÝ¶gl/9Tî±¿äèîm_æüywþ'GË®Ñnø7=fWÒD´É6$ýÐ<%Cªzó³;ãÍ/ÁSNÞÚwñâû·'Ø[ìÉy<¢pÇÀüÍþ-1?söÔhÔ=Ý5u!¤ôõÒ'È`¾Ñì½:úË#$^H-C2Ò·uîÎ.Ü[²Ç«ìÄÅúß0®Õñpußî_¶>X·ã|Ù:Ü¿>Hû85¨Ûæ²LJõÈ±Á6Ò8l£¼¼´þ4¨Ñ­¾õ§¯ß»ü|ÅNßi(Z_©ÅoÀtÉ¾V=øêFTÖ¹¸@Ìï`ÑÚKnæ§TJ»»Êê*ï´2DÂ¶q?çq	ìYáhzoVí¥]·n:`·;âýmV³Úéñ:ÄSþô^N>ØFÊ¿%o#çWv6¸ÎÃ­Õï$;Ø¶I +ûøù	¼g=5"Fß°ñ¶aÑÒl¼âx±â[ÄüÿKïÞãï	.ÐYàÃ"mA³¶Ë¼wø9½ï?|Ìæóy¿ÕQSîÕÁ|68L§âA`O/mT¬æAF¶ÛCJÍE l§vÐu>?º¶ÌáYþ¸üCþÛ¯¤üãDÂÛÂÖÙé£ORmvy+VÉµÔ²f´GòâýÔ²f¤³âÅ<-eR!=jâ-Q="|Dû¬Ò³ÏÑ$×j®§hn§kjÌìù`~0?`,7qþEÌ&4N&u´5'ÕUÞárÇ%3RÑ*îéå8?WQß¨©´N ''R4Si£Âl¦Ý£¤x1ë_ÚC:"äÑòñÁí>Wê*ØñeÚê-qü#|Ã¶³¯ªõiÎÊp¯~Í¥GôªzóG7¤g1Ý"mY.xùæn6«QÛÜ="ù`~0¿IB/o;íÇ#-þAÌÏI±nï*Øk`_)clÍ®/²êã8ú±è_F¢m¬/ífL~)qDJ-6$OØ¦¦"y¥îýÇt¼A5tÔàHW(6ãUréMÔÂÔñ©BS·ßO=z:!Õ=;Õ7ørì®¤¼t·An2@ÒOö>zßÌÒõðÏ¬Àßtÿ!1d1?ÞÃ¿´þôvgÐo»ßóàOæ4¢îÁüÚebNbavO×Y?^ìýezØÏR~â3#á.%mÿìÿ³§÷ÉvÓ¾QªSi3ké¸bØ]k(yæ/kfÊP+5¤ræÖÖç0s¨ÞñMËcLÜÞHuzâýå]ù"Ø¿×z_¯½¼Vzz­»CzoxV_¾&O9­p»ôÁY|IDATm¯O¼½ÿd¼ÃËn2@t<8A»¾ÆÌR~Ù®üò_I¥äÒ_H©¹òQóç«®~)åWW]ù++ÂË</yÎv¹ÎvÙþ¶<ÎÇ0ÒöæÓ0òðçéÊK46Ã¾Ñ»­¥¾ÄÈ¦Ñu_ÿOvíLÚ®û"Yý8NÅµ"T£1yÃçÉ³µ¼UÐUw1ÖïkçuN?>âñ·]Á+w?øüDøg÷bª'Ù2$I9-^3ÒrÇÉ¯ùJ¬À¶Í/!!áG?8íyóæM>Ñ¢EÉÉÉnùMpøôlVV%9ì´³¤¼=ûá® ûûOÞ;ZºËñþKNr+ÌÑ3ü<)÷¢.D;ò8Þ%!Þ%)áFö÷Üd÷Ê§ÞÍüÎFÚÏÉ¥ÅºÏ'g°ÁmýdÜ³®XWòjïë!M¼0ì®W7¥gÒ3Öhb£eH,È/3qXEýË1¢rÖ7ÀÍO©T.Y²Do~6lð÷÷'77·7ºÅÈü"##9ÀfyXXD*÷2Ï¥zaB¾Ug³ªÙm¬æãk¯ÿÏÆ¿°_²Z=Ù¬½õ9]áÝ·3ùOö"×c7I<,ôî©é&;ÝFéÚÙmUm%OÂk]/Ú.X³?å¹5×âé!ä)²Mµµ³J+OÒk½ï^pÏ´?ýdí¨ÛÂvH<ãA6F&VÈnæÓÞÕÚ!Îé¿%÷LS]¢º¤ÛÊb«p+°i`æ7Î?ùòe½ùÍ;W­ÖÍËåo¾ùæ [ÌïöíÛyÀfIÌyYpãvVX@úYb~Ñ¹érÝïæ[Ü`qáÃªÒôÊOkþYûsR©-ßWZäi[W&;¥(ýAeªý§æøoÚ£·ò"V¢6òcvv%hO¾ÖøÌ·.3¼"7«`tO#/+"÷_îõK¹foú:û½CÙkÎçØ»çëoÝËÊÎá~7åKâ³qîüpë£u"¾>ûí¥x×;)%<ÉPï9Orf;ÃI!ÿQìÓÏÜñÙö0ó[ ÷ÌÀüÌÉd.]ºÞü¦O®ªnAoï$£KO+tóO?EÌ/µÀ5®"dDËr(­2Ñ×Ëü¨£þ?8ßör¤Â¬J«QÚÄÐ/kF­]ûÌAµVôx8ã´Ô½öH'OØ¦VÉWýòF¹ôÆqâ-+.¬`Ç>mr¹[¬_ÎþLªáªt"b¼Þ>rnIÍæØÚÄñIIõaÇó#[of&ÞÊAÍ¸ilÕüÖ®]¦;éïÍoÚ´iúggÌ1èß§_ wªGT,ÑýOó*^¾?GÁo­â6F±ªN¶þ­¹ðCnËgºÒü?bÏtL%y¤R|c@òÈª±ò«aÇÈoå±=Óü2¤âì]H6¦d3;:EôCÓ¤¹Rv9¥ëìcÝÒpws´2î)a~?z²åõ×_Ëu_zä'©ºæ7Á!¾R4ÒòìJzq·BtÐ&$oÐ/TcD*¤7ÎËcX¾¬ª¾;*¹áf`éñkY[¿I¤&e6gûã&¯ÌYg~Ú²6WÛ3?C¤*7oöóó#òsÃnùMpFó°Øü?vì~ìw÷Èß[³ÖuÕû8²¾6:·!¼£òXKÑ'MùËÚÊwvÒ¼y'×¨Íx§eÍ,xK¬ZÃVBzÖB®P52ùThðÌÍÜíß&°õÑÝÑ_xâäO6V°ÛûT*ÍØ[WvãcÜ.SÅüôªgô0##ã7Þ6mÚ¼yórrrÝóL¹7ºãöGyXøÔKRãÑweIGõôp~ÍëøBÌ÷WÊ&N7®B¬émøaÅÌï¤Û%1QÛËcï(½	«~aU'odoý&è _Æ3¤"<^RÝÉáa~ßDægëôÊ´#Äü¸mëú²wtÔ¼¦VñÆýÜÒTwíÓ¸MºdÈÉ$×d5÷tÉÉâNµjj!¤g-T*Ms_FaÛ½èÚsîy_x²éÐãO÷Çí:âä[7©§a~Ìo*Ò¾W¾Vg~!ºÌÿ8á0+o®"; ¢©9ÍQïhF¿``L^Ý]wmú7Ýµ©ûtËÉkSt×¨ãµR¨åMÂj*¤ç÷`á*ÃoÙR&BzVD$:xæfîÖoÿúE81Â]J¿XD/¬`s$·_àÑyöñ¤YÈóùM]òXÕ±if¨4×_GÖøfgºW]ÎõÍÛöØ>:ájTÜ¥ÈÈ³¡Ç#"ý÷EÞ?öÙ1:ñztªt¦oL^PLñ£¾vº×¡UÕ#,îTs+UE¥_þEq»è¿ã6éºkÉCb~-)ºeÍû*ø¥¼Ìøö ÏúÓTHï|ÅNÓå1ÀÁáKª;£Üï½¹~oÌG;"¾:ñ¨¡~%æ!BIòÁ7±3qäpÒ.,æó³a¢ÊÃe¡»|.ùî¯¢ýKÅG*ÒkË¼Èù÷ýÙw¢oçW]cdízv /nWxMFP´e(LõD<í÷!½Nlh³ûÕêû>:R¼^?J!½T¦$&QØ[wÕ·hÿ¹§«vFêóËÜ®%OôHzø²5»£íyêz[Y-ñ?Ìæ7P<âþÁw>ÿQßzÎE|a£F"i#"]¨l»ñrqRvtF­îKØÔöUcáþÜªC¡Ì£Øíûö¾KØ¡äì*©Ë2ßoúCÎÉßVdRËªæóÃ¯HOÒóm¸@%X9_±ÓîÝê[Þ­ ËÉw>~,®9æl¶eÜFAÞ·wù=ÙÊÎ<&fÂF5Ð]Û¢(uïÏ>%²KµVô$ç¬¬Ü³¡`å)ù¡S½(úÛ©9ÍÄóíç#æGüX qAéåPáCJÉCé­]ýß	¢nô¡ dûý(½à¦ëTHÏ¡hkíÐf÷Ü®¤v	C¥Á/Û#Ð2yÃe]ÜZë|Êm§TïËiÇ÷GÛE=ûÍÈ¶ðB°QzÔ1ÉÏRç`b~D~rÏËJnêBnJ©FÀÔEòã>ýd;Ñ»ODñ[uËßèoVgGkY³ç¨j®PÕd+ò¢QrvøèrÑî_	¢<ú<ÉìOjmH,cÆF6zÖ¦Bzå_ú7^JeÓBïþ$4v3°T"ª?;¨ÛÛÈäoý&WóùM	êÈkîéÄ2?z¢à²,r­èÉ.]<ò3ûäµgë$oÂÅÅ¾ÄªÄú<ÄgS>wLµÛñîîÿ9ÿGÏàßGßþ]±ëÜëÙ¡ÅáC`»p$kvGç±ùm:ô¸OØo&è ®æó³UJyÑ­¾fï(ï4Rñ¸àÊËäáÝTç+¡Uw¢ÍmD¢ëZs&S	V¾È~çHñz×Ú#LnJ»ñÃÃDûü4C>N&Jª;WíüÇáÇí ÷eã²æóÖ!¾=Èålýñ¸=¤BÌR9cÿmÔó[ e&CæL¦A3ZC¦¾âK·jô¡©v.SsS 6T¦¼äY°vOLG§WóùÙ6ýÒ|q_EÈ)p ÅÝ~ô¥â']´à<VE°ù-¢Q[­­n»ÝêK-FTïxÉ¦q_ñVoFáC"ÔÜ¡ÂF86§º7¿KþýÀIÁ5ÀüÀ¢¯ã(§aù]ÿ+fÉ¯ÜÃ¶ók.XØ^õ«NJÞöª_Þj9­¯4eÒ£Ç°¹oMÃF¨¼þe+ü«vYañÙn¿ñso$×óé0ð;Ý¤×¯_íMøÄIÑ±F>¤§x;åÇ6»9£>|t1¿m~¸ÌÏæèa¹vmoâZ$å¥ò¡Ï¥ØxÅ¹j/ñ<b×kcÅÛ2ÌèÃ¡r>Âü0?x;ñÞÖY«5Ãå1lÒôÒ8QXñÖú1yÙ´svóÀü`~àUÍO¦­xK-ÖâIü¯SÖËkvh+C+ØIÍ#-ýà/[B-8|0?`Û¤²ÃÏ<:º'qµgýév	ã¥ûS9V¨QzÇK6Ùç¯ ÂÜt=ETz?0vhÁÐÃq±ÃCÒÃeÁ'ó6ÌmÚw+w»Å¸Ø'­o:X¸-eî SI[Å¹]I¡ÍîÎUç.ÛJ%X)åeW0^øp¨Îå>$§MÎï&æó³y(oôY8·úL)t^¼ä±'~-ÙëD»:#årôÚÇïÉß%Î=WúøbCú®¢2£FúX¼È×MÓÌØ~öøP¼ï7äE-0?0 =gYÂ5E;´Ê5ÏÛÖï¯ºcç³'÷ÁÚ¶Ä#]O^Ú¬æ,aÄ±½ô/?RÄ©êòÔÌ öKÌIµJ×øµÏûNJ½/½%>òGÒÞÌæ7%kñLféÆ-6ßÚ_°²UÜ Vö|+&  *Ëô/]ùÓ/mÔ	âÁßdÆvú»ôæWºé)ä	^ìpei²®ÛJ/JÎ­û=ïíU¤øÄEÀü`~S~Ñ·ÅÊTÒèVß#ÅëµÏý½MûWØöKt±5ÓSÉcoéú±úH|äºðáþßüI§gV-º#ýb?à`~0¿©B`ã¥[nÕ;X¸*¸é:ñ?äayøUyô½HæDòí_tÊ(( Àü`~S¢îg§Ë·~÷°OÌÕâ|ïþªõêû#®É|¿ÁÀü`~So/þN³«dÞ%GËüéDpBf¨Àü`~æPäEK.¬x[K#êÅÀü`~0?&3ÈäùÁü`~0?óÌæÌ`~0?0?ÌæÆÐfwâp#-_d¿CÒÓßË`~ÌtAEvgüXJË`~ÌF©Ë¢Ý¿ËkùÀø0°PïÌæóùÁü`~0?ÌæóùÁü`~0?ÌæóùÀü`~0?ÌæóùÀü`~0¿ÑäôéÓ>lÆ	>ù0¿1Éd<yò,0N ë	ÀüÌÀüÌÀü`~æ`~æÕ>/2cÆE¹¸¸î`z®x=Ó¦M9sæ%KÜÝÝqé0?0I¾ªÕê´´´·ÞzKÿUgj~Ð>0¥>ðµµµï¼ó««+®æ&Û!Euu5ù.thRx½üÍ?Wóó0útÓ `Jà1c®æ&çaFFih®"xCjkk/^«ùÉöE(ÉîÝ»÷úë¯î@´oß¾Ë-ÃSá¯øË_þ2::WóáÐ9sælØ°!))Ép'ïùóç¯]»&÷ÞÙ³g¯^½ÚpùÛþ"¤*jµúÒ¥Kvvv<ÏhýØ>²ÏÒ¥K®ÄxÌLþ/BóçÏþùçÃìPRRòÁàº)òÌ_"vnnnÃìààà À¤ÿÀÌLæ/B.»páB&9Ô"hÉ%¸z`*|àæ&ó!¡²²rõêÕÃì°yóf0E>ðó0?ùùùùùÀüÌÀüÌÀüÌÀüÌÀüÌ©BBBÝ¬Y³¦O>gÎmÛ¶ÕÖÖîå9¸òó)÷îÝû	³gÏUùùÌ0üô§?%æèè(ÈC&iggG¶¬]»`~IÅ3çFøÚÚÚÈ3güñ?çñãÇ/>ú¢E[ .";ÝJNN^²d	y´öÞï1Ã6_ÚÇÛ¹sçìÙ³ÉSsçÎ=pàH$Â[0?¬]»HQ«-[¶ÄÆÆªÕjã?þçL6Í°;8//z¶¼¼|¨§:õqDCó¦Õ«Wµ°oß>¼eóXË]²dá?bWFæ·aÃÞÞ^@@¢ÞáÈvò099Ô322HãÆÔSë×¯'wíÚ%Ë©§fÍej~Ã´@!É$ub¥-ù,$,,ÈÜôéÓ)'9sfZZ¡¥±Ùlê!©PH=$ÓÙ!OÚ?kh~Ã´@)é>FFFJ$¼SóX¥R½|ùrâ[ÄºL-M¿8¢á³L6m¨5¿aZ ÑhFñHrzxæ°¹sç©ÒÏ½ ¨ÕjS·ÓïÐÙÙi5ky(ËM[¦yÔáaÌo(N:Eõ2Ï7oÀü²sçNbTï¾û.F#¨9rlY´h¡¥ÙÙÙq¹D²yófòpË-Ô³8pøbBB©/^¼zççàà@JNN&õùóçß0-PãübccµÏã¤>cÆ¼eóXÉ|ýõ×M»ÂÂ-mæÌú§~é³ÀäååJ=e:·÷Ò¥K¦æ7L7n4zjÇxËæ°±ûö×^2<;;;Ãreee-Y²dÚ´iK.ÍÈÈ0<üñãÇd#yjÞ¼y^^^Ov¨£ÈSFm¾´HtäÈ7ÞxìLNïÐ¡C2ïÀü£öÇÖæùù&3ë0?ó0?ó0?ó0?ó0?óùùùù`bÝ_ÃºíêùD¢mÛ¶Í1ãµ×^³··ïìì$årù ¿QBBÂôç$%%Q[ª««ßyçaÚ§Óévvv¤ýyóæ¹¹¹ÙïùÀ¨M·èÐ!6M*jµÙ²eËHÝªU«Lw¦@*Ôõë×§¦¦Õ8iyÁaaa¤ñ¶¶6â0?ÌóögÍ¥¯?9s&©Ü»wïÀÃU©¬¬|÷Ýw×Êk×®éVWW/Y²æù&¡ù§<<<,X`Tçr¹+V¬>ú|ÀãñLw6j¤±±ÙHm,((Xºt)iáõ×_'fÚÕ-;mÚ´Ågddsª3fÌ ÂGÕår9yH*»ví5ÝÙ(æ·zõjãòÖ[o1Ìq1ZÌFÅüçG*Äd2QxU`` åg;wî4ÝÙ¨"gyyyä@jãÂÓÒÒH%22rÎ9¦-3£099ÙÔ&Ù¶miHÚ§N~ÕªUÄMg=¼ºH$¢v¦@Î¼²²rùòåÃ_§0ÌóLóæ)8Í°þÚk¯QuòÓÐÛô;5¢9ÃºmÙ²hR©þü7oÞLmþüùÑÑÑTÌ%¤A"ÄM$/c:çæù¦¨ù½´®w£¡ÚtgâNNNÄØ.öN¿Õ¡<öìòòr3:>oÞ<ÓíRTTDüLç|²xñb¡H<È%ÌóL!ó3g#&DÌì¥æ'H¨çÎKml££cll,ÉÔüô¦åïï?¨ÌJddä-[L15?;;»­É#gxA|ûí·a~`ß;¨¾TbBãüjäèÑ£¤uèÐ!jãÌ3étº'[u¯&$$h¿7Ìù=ðÁ >WRRBm$/Gi_`` ÑHÁý¿ác~DLw>~üXû|bïüùó©×ù`~[5¿afxj~÷> ª´bÅÃ¹½CµO¼êÍ7ßr¥@  6ÆÆÆ93g»»û /GälÑ¢EÓ¦M£fcóDõÞ~ûm²Û[o½Fm$ZIÎpÆD1·mÛfØcK OåååQuÃ9?9ªò+xyycÌó0?óùùùùùÀãÿírüH2ÿIEND®B`


UNIANOVA cont BY Species NitroS
  /METHOD=SSTYPE(3)
  /INTERCEPT=INCLUDE
  /POSTHOC=Species NitroS(TUKEY)
  /PLOT=PROFILE(Species*NitroS) TYPE=LINE ERRORBAR=CI MEANREFERENCE=NO YAXIS=AUTO
  /EMMEANS=TABLES(OVERALL)
  /EMMEANS=TABLES(Species) COMPARE ADJ(LSD)
  /EMMEANS=TABLES(NitroS) COMPARE ADJ(LSD)
  /EMMEANS=TABLES(Species*NitroS)
  /PRINT ETASQ DESCRIPTIVE HOMOGENEITY
  /CRITERIA=ALPHA(.05)
  /DESIGN=Species NitroS Species*NitroS.


Univariate Analysis of Variance


Notes	
Output Created	10-MAY-2026 19:46:37	
Comments		
Input	Active Dataset	DataSet5	
	Filter	<none>	
	Weight	<none>	
	Split File	<none>	
	N of Rows in Working Data File	84	
Missing Value Handling	Definition of Missing	User-defined missing values are treated as missing.	
	Cases Used	Statistics are based on all cases with valid data for all variables in the model.	
Syntax	UNIANOVA cont BY Species NitroS
  /METHOD=SSTYPE(3)
  /INTERCEPT=INCLUDE
  /POSTHOC=Species NitroS(TUKEY)
  /PLOT=PROFILE(Species*NitroS) TYPE=LINE ERRORBAR=CI MEANREFERENCE=NO YAXIS=AUTO
  /EMMEANS=TABLES(OVERALL)
  /EMMEANS=TABLES(Species) COMPARE ADJ(LSD)
  /EMMEANS=TABLES(NitroS) COMPARE ADJ(LSD)
  /EMMEANS=TABLES(Species*NitroS)
  /PRINT ETASQ DESCRIPTIVE HOMOGENEITY
  /CRITERIA=ALPHA(.05)
  /DESIGN=Species NitroS Species*NitroS.	
Resources	Processor Time	00:00:00.25	
	Elapsed Time	00:00:00.45	


Warnings	
Post hoc tests are not performed for Species because there are fewer than three groups.	


Between-Subjects Factors	
	Value Label	N	
Species	1	RK	42	
	2	RP	42	
NitroS	1	Pep	6	
	2	Urea	6	
	3	YeEx	6	
	4	NCl	6	
	5	NS	6	
	6	Pep+YeEx	6	
	7	Urea+Pep	6	
	8	YeEx+Urea	6	
	9	Pep+NCl	6	
	10	Urea+NCl	6	
	11	YeEx+NCl	6	
	12	Pep+NS	6	
	13	Urea+NS	6	
	14	YeEx+NS	6	


Descriptive Statistics	
Dependent Variable:   cont  	
Species	NitroS	Mean	Std. Deviation	N	
RK	Pep	21.49000	2.823314	3	
	Urea	19.58333	.100167	3	
	YeEx	26.87667	.512868	3	
	NCl	13.73333	.428291	3	
	NS	14.03000	.517977	3	
	Pep+YeEx	32.17667	1.022562	3	
	Urea+Pep	17.39000	.338674	3	
	YeEx+Urea	21.33667	1.092352	3	
	Pep+NCl	11.05667	1.161737	3	
	Urea+NCl	14.51000	1.234058	3	
	YeEx+NCl	10.08667	.361156	3	
	Pep+NS	26.03333	.257747	3	
	Urea+NS	19.88333	2.661228	3	
	YeEx+NS	31.48667	.839424	3	
	Total	19.97667	7.016441	42	
RP	Pep	31.85000	3.496298	3	
	Urea	9.64000	1.269173	3	
	YeEx	22.96667	1.140365	3	
	NCl	9.99333	.202320	3	
	NS	42.51333	1.170741	3	
	Pep+YeEx	27.48000	1.028056	3	
	Urea+Pep	31.10333	2.063355	3	
	YeEx+Urea	35.58667	2.127401	3	
	Pep+NCl	13.56667	1.646856	3	
	Urea+NCl	5.49333	.322542	3	
	YeEx+NCl	9.67333	.251661	3	
	Pep+NS	26.91333	.793872	3	
	Urea+NS	26.23333	.740698	3	
	YeEx+NS	16.91000	1.018381	3	
	Total	22.13738	11.144552	42	
Total	Pep	26.67000	6.346413	6	
	Urea	14.61167	5.505388	6	
	YeEx	24.92167	2.282940	6	
	NCl	11.86333	2.070272	6	
	NS	28.27167	15.621961	6	
	Pep+YeEx	29.82833	2.731047	6	
	Urea+Pep	24.24667	7.626631	6	
	YeEx+Urea	28.46167	7.950244	6	
	Pep+NCl	12.31167	1.874763	6	
	Urea+NCl	10.00167	5.004084	6	
	YeEx+NCl	9.88000	.358831	6	
	Pep+NS	26.47333	.714833	6	
	Urea+NS	23.05833	3.892179	6	
	YeEx+NS	24.19833	8.027481	6	
	Total	21.05702	9.319453	84	


Levene's Test of Equality of Error Variancesa,b	
	Levene Statistic	df1	df2	Sig.	
cont	Based on Mean	3.365	27	56	.000	
	Based on Median	.841	27	56	.682	
	Based on Median and with adjusted df	.841	27	14.177	.663	
	Based on trimmed mean	3.099	27	56	.000	

Tests the null hypothesis that the error variance of the dependent variable is equal across groups.a,b	
a. Dependent variable: cont	
b. Design: Intercept + Species + NitroS + Species * NitroS	


Tests of Between-Subjects Effects	
Dependent Variable:   cont  	
Source	Type III Sum of Squares	df	Mean Square	F	Sig.	Partial Eta Squared	
Corrected Model	7101.957a	27	263.035	137.952	.000	.985	
Intercept	37245.453	1	37245.453	19533.780	.000	.997	
Species	98.042	1	98.042	51.419	.000	.479	
NitroS	4400.007	13	338.462	177.510	.000	.976	
Species * NitroS	2603.907	13	200.301	105.050	.000	.961	
Error	106.776	56	1.907				
Total	44454.186	84					
Corrected Total	7208.733	83					

a. R Squared = .985 (Adjusted R Squared = .978)	


Estimated Marginal Means


1. Grand Mean	
Dependent Variable:   cont  	
Mean	Std. Error	95% Confidence Interval	
		Lower Bound	Upper Bound	
21.057	.151	20.755	21.359	


2. Species


Estimates	
Dependent Variable:   cont  	
Species	Mean	Std. Error	95% Confidence Interval	
			Lower Bound	Upper Bound	
RK	19.977	.213	19.550	20.403	
RP	22.137	.213	21.711	22.564	


Pairwise Comparisons	
Dependent Variable:   cont  	
(I) Species	(J) Species	Mean Difference (I-J)	Std. Error	Sig.b	95% Confidence Interval for Differenceb	
					Lower Bound	Upper Bound	
RK	RP	-2.161*	.301	.000	-2.764	-1.557	
RP	RK	2.161*	.301	.000	1.557	2.764	

Based on estimated marginal means	
*. The mean difference is significant at the .05 level.	
b. Adjustment for multiple comparisons: Least Significant Difference (equivalent to no adjustments).	


Univariate Tests	
Dependent Variable:   cont  	
	Sum of Squares	df	Mean Square	F	Sig.	Partial Eta Squared	
Contrast	98.042	1	98.042	51.419	.000	.479	
Error	106.776	56	1.907				

The F tests the effect of Species. This test is based on the linearly independent pairwise comparisons among the estimated marginal means.	


3. NitroS


Estimates	
Dependent Variable:   cont  	
NitroS	Mean	Std. Error	95% Confidence Interval	
			Lower Bound	Upper Bound	
Pep	26.670	.564	25.541	27.799	
Urea	14.612	.564	13.482	15.741	
YeEx	24.922	.564	23.792	26.051	
NCl	11.863	.564	10.734	12.993	
NS	28.272	.564	27.142	29.401	
Pep+YeEx	29.828	.564	28.699	30.958	
Urea+Pep	24.247	.564	23.117	25.376	
YeEx+Urea	28.462	.564	27.332	29.591	
Pep+NCl	12.312	.564	11.182	13.441	
Urea+NCl	10.002	.564	8.872	11.131	
YeEx+NCl	9.880	.564	8.751	11.009	
Pep+NS	26.473	.564	25.344	27.603	
Urea+NS	23.058	.564	21.929	24.188	
YeEx+NS	24.198	.564	23.069	25.328	


Pairwise Comparisons	
Dependent Variable:   cont  	
(I) NitroS	(J) NitroS	Mean Difference (I-J)	Std. Error	Sig.b	95% Confidence Interval for Differenceb	
					Lower Bound	Upper Bound	
Pep	Urea	12.058*	.797	.000	10.461	13.655	
	YeEx	1.748*	.797	.032	.151	3.345	
	NCl	14.807*	.797	.000	13.210	16.404	
	NS	-1.602*	.797	.049	-3.199	-.005	
	Pep+YeEx	-3.158*	.797	.000	-4.755	-1.561	
	Urea+Pep	2.423*	.797	.004	.826	4.020	
	YeEx+Urea	-1.792*	.797	.029	-3.389	-.195	
	Pep+NCl	14.358*	.797	.000	12.761	15.955	
	Urea+NCl	16.668*	.797	.000	15.071	18.265	
	YeEx+NCl	16.790*	.797	.000	15.193	18.387	
	Pep+NS	.197	.797	.806	-1.400	1.794	
	Urea+NS	3.612*	.797	.000	2.015	5.209	
	YeEx+NS	2.472*	.797	.003	.875	4.069	
Urea	Pep	-12.058*	.797	.000	-13.655	-10.461	
	YeEx	-10.310*	.797	.000	-11.907	-8.713	
	NCl	2.748*	.797	.001	1.151	4.345	
	NS	-13.660*	.797	.000	-15.257	-12.063	
	Pep+YeEx	-15.217*	.797	.000	-16.814	-13.620	
	Urea+Pep	-9.635*	.797	.000	-11.232	-8.038	
	YeEx+Urea	-13.850*	.797	.000	-15.447	-12.253	
	Pep+NCl	2.300*	.797	.006	.703	3.897	
	Urea+NCl	4.610*	.797	.000	3.013	6.207	
	YeEx+NCl	4.732*	.797	.000	3.135	6.329	
	Pep+NS	-11.862*	.797	.000	-13.459	-10.265	
	Urea+NS	-8.447*	.797	.000	-10.044	-6.850	
	YeEx+NS	-9.587*	.797	.000	-11.184	-7.990	
YeEx	Pep	-1.748*	.797	.032	-3.345	-.151	
	Urea	10.310*	.797	.000	8.713	11.907	
	NCl	13.058*	.797	.000	11.461	14.655	
	NS	-3.350*	.797	.000	-4.947	-1.753	
	Pep+YeEx	-4.907*	.797	.000	-6.504	-3.310	
	Urea+Pep	.675	.797	.401	-.922	2.272	
	YeEx+Urea	-3.540*	.797	.000	-5.137	-1.943	
	Pep+NCl	12.610*	.797	.000	11.013	14.207	
	Urea+NCl	14.920*	.797	.000	13.323	16.517	
	YeEx+NCl	15.042*	.797	.000	13.445	16.639	
	Pep+NS	-1.552	.797	.057	-3.149	.045	
	Urea+NS	1.863*	.797	.023	.266	3.460	
	YeEx+NS	.723	.797	.368	-.874	2.320	
NCl	Pep	-14.807*	.797	.000	-16.404	-13.210	
	Urea	-2.748*	.797	.001	-4.345	-1.151	
	YeEx	-13.058*	.797	.000	-14.655	-11.461	
	NS	-16.408*	.797	.000	-18.005	-14.811	
	Pep+YeEx	-17.965*	.797	.000	-19.562	-16.368	
	Urea+Pep	-12.383*	.797	.000	-13.980	-10.786	
	YeEx+Urea	-16.598*	.797	.000	-18.195	-15.001	
	Pep+NCl	-.448	.797	.576	-2.045	1.149	
	Urea+NCl	1.862*	.797	.023	.265	3.459	
	YeEx+NCl	1.983*	.797	.016	.386	3.580	
	Pep+NS	-14.610*	.797	.000	-16.207	-13.013	
	Urea+NS	-11.195*	.797	.000	-12.792	-9.598	
	YeEx+NS	-12.335*	.797	.000	-13.932	-10.738	
NS	Pep	1.602*	.797	.049	.005	3.199	
	Urea	13.660*	.797	.000	12.063	15.257	
	YeEx	3.350*	.797	.000	1.753	4.947	
	NCl	16.408*	.797	.000	14.811	18.005	
	Pep+YeEx	-1.557	.797	.056	-3.154	.040	
	Urea+Pep	4.025*	.797	.000	2.428	5.622	
	YeEx+Urea	-.190	.797	.812	-1.787	1.407	
	Pep+NCl	15.960*	.797	.000	14.363	17.557	
	Urea+NCl	18.270*	.797	.000	16.673	19.867	
	YeEx+NCl	18.392*	.797	.000	16.795	19.989	
	Pep+NS	1.798*	.797	.028	.201	3.395	
	Urea+NS	5.213*	.797	.000	3.616	6.810	
	YeEx+NS	4.073*	.797	.000	2.476	5.670	
Pep+YeEx	Pep	3.158*	.797	.000	1.561	4.755	
	Urea	15.217*	.797	.000	13.620	16.814	
	YeEx	4.907*	.797	.000	3.310	6.504	
	NCl	17.965*	.797	.000	16.368	19.562	
	NS	1.557	.797	.056	-.040	3.154	
	Urea+Pep	5.582*	.797	.000	3.985	7.179	
	YeEx+Urea	1.367	.797	.092	-.230	2.964	
	Pep+NCl	17.517*	.797	.000	15.920	19.114	
	Urea+NCl	19.827*	.797	.000	18.230	21.424	
	YeEx+NCl	19.948*	.797	.000	18.351	21.545	
	Pep+NS	3.355*	.797	.000	1.758	4.952	
	Urea+NS	6.770*	.797	.000	5.173	8.367	
	YeEx+NS	5.630*	.797	.000	4.033	7.227	
Urea+Pep	Pep	-2.423*	.797	.004	-4.020	-.826	
	Urea	9.635*	.797	.000	8.038	11.232	
	YeEx	-.675	.797	.401	-2.272	.922	
	NCl	12.383*	.797	.000	10.786	13.980	
	NS	-4.025*	.797	.000	-5.622	-2.428	
	Pep+YeEx	-5.582*	.797	.000	-7.179	-3.985	
	YeEx+Urea	-4.215*	.797	.000	-5.812	-2.618	
	Pep+NCl	11.935*	.797	.000	10.338	13.532	
	Urea+NCl	14.245*	.797	.000	12.648	15.842	
	YeEx+NCl	14.367*	.797	.000	12.770	15.964	
	Pep+NS	-2.227*	.797	.007	-3.824	-.630	
	Urea+NS	1.188	.797	.142	-.409	2.785	
	YeEx+NS	.048	.797	.952	-1.549	1.645	
YeEx+Urea	Pep	1.792*	.797	.029	.195	3.389	
	Urea	13.850*	.797	.000	12.253	15.447	
	YeEx	3.540*	.797	.000	1.943	5.137	
	NCl	16.598*	.797	.000	15.001	18.195	
	NS	.190	.797	.812	-1.407	1.787	
	Pep+YeEx	-1.367	.797	.092	-2.964	.230	
	Urea+Pep	4.215*	.797	.000	2.618	5.812	
	Pep+NCl	16.150*	.797	.000	14.553	17.747	
	Urea+NCl	18.460*	.797	.000	16.863	20.057	
	YeEx+NCl	18.582*	.797	.000	16.985	20.179	
	Pep+NS	1.988*	.797	.016	.391	3.585	
	Urea+NS	5.403*	.797	.000	3.806	7.000	
	YeEx+NS	4.263*	.797	.000	2.666	5.860	
Pep+NCl	Pep	-14.358*	.797	.000	-15.955	-12.761	
	Urea	-2.300*	.797	.006	-3.897	-.703	
	YeEx	-12.610*	.797	.000	-14.207	-11.013	
	NCl	.448	.797	.576	-1.149	2.045	
	NS	-15.960*	.797	.000	-17.557	-14.363	
	Pep+YeEx	-17.517*	.797	.000	-19.114	-15.920	
	Urea+Pep	-11.935*	.797	.000	-13.532	-10.338	
	YeEx+Urea	-16.150*	.797	.000	-17.747	-14.553	
	Urea+NCl	2.310*	.797	.005	.713	3.907	
	YeEx+NCl	2.432*	.797	.003	.835	4.029	
	Pep+NS	-14.162*	.797	.000	-15.759	-12.565	
	Urea+NS	-10.747*	.797	.000	-12.344	-9.150	
	YeEx+NS	-11.887*	.797	.000	-13.484	-10.290	
Urea+NCl	Pep	-16.668*	.797	.000	-18.265	-15.071	
	Urea	-4.610*	.797	.000	-6.207	-3.013	
	YeEx	-14.920*	.797	.000	-16.517	-13.323	
	NCl	-1.862*	.797	.023	-3.459	-.265	
	NS	-18.270*	.797	.000	-19.867	-16.673	
	Pep+YeEx	-19.827*	.797	.000	-21.424	-18.230	
	Urea+Pep	-14.245*	.797	.000	-15.842	-12.648	
	YeEx+Urea	-18.460*	.797	.000	-20.057	-16.863	
	Pep+NCl	-2.310*	.797	.005	-3.907	-.713	
	YeEx+NCl	.122	.797	.879	-1.475	1.719	
	Pep+NS	-16.472*	.797	.000	-18.069	-14.875	
	Urea+NS	-13.057*	.797	.000	-14.654	-11.460	
	YeEx+NS	-14.197*	.797	.000	-15.794	-12.600	
YeEx+NCl	Pep	-16.790*	.797	.000	-18.387	-15.193	
	Urea	-4.732*	.797	.000	-6.329	-3.135	
	YeEx	-15.042*	.797	.000	-16.639	-13.445	
	NCl	-1.983*	.797	.016	-3.580	-.386	
	NS	-18.392*	.797	.000	-19.989	-16.795	
	Pep+YeEx	-19.948*	.797	.000	-21.545	-18.351	
	Urea+Pep	-14.367*	.797	.000	-15.964	-12.770	
	YeEx+Urea	-18.582*	.797	.000	-20.179	-16.985	
	Pep+NCl	-2.432*	.797	.003	-4.029	-.835	
	Urea+NCl	-.122	.797	.879	-1.719	1.475	
	Pep+NS	-16.593*	.797	.000	-18.190	-14.996	
	Urea+NS	-13.178*	.797	.000	-14.775	-11.581	
	YeEx+NS	-14.318*	.797	.000	-15.915	-12.721	
Pep+NS	Pep	-.197	.797	.806	-1.794	1.400	
	Urea	11.862*	.797	.000	10.265	13.459	
	YeEx	1.552	.797	.057	-.045	3.149	
	NCl	14.610*	.797	.000	13.013	16.207	
	NS	-1.798*	.797	.028	-3.395	-.201	
	Pep+YeEx	-3.355*	.797	.000	-4.952	-1.758	
	Urea+Pep	2.227*	.797	.007	.630	3.824	
	YeEx+Urea	-1.988*	.797	.016	-3.585	-.391	
	Pep+NCl	14.162*	.797	.000	12.565	15.759	
	Urea+NCl	16.472*	.797	.000	14.875	18.069	
	YeEx+NCl	16.593*	.797	.000	14.996	18.190	
	Urea+NS	3.415*	.797	.000	1.818	5.012	
	YeEx+NS	2.275*	.797	.006	.678	3.872	
Urea+NS	Pep	-3.612*	.797	.000	-5.209	-2.015	
	Urea	8.447*	.797	.000	6.850	10.044	
	YeEx	-1.863*	.797	.023	-3.460	-.266	
	NCl	11.195*	.797	.000	9.598	12.792	
	NS	-5.213*	.797	.000	-6.810	-3.616	
	Pep+YeEx	-6.770*	.797	.000	-8.367	-5.173	
	Urea+Pep	-1.188	.797	.142	-2.785	.409	
	YeEx+Urea	-5.403*	.797	.000	-7.000	-3.806	
	Pep+NCl	10.747*	.797	.000	9.150	12.344	
	Urea+NCl	13.057*	.797	.000	11.460	14.654	
	YeEx+NCl	13.178*	.797	.000	11.581	14.775	
	Pep+NS	-3.415*	.797	.000	-5.012	-1.818	
	YeEx+NS	-1.140	.797	.158	-2.737	.457	
YeEx+NS	Pep	-2.472*	.797	.003	-4.069	-.875	
	Urea	9.587*	.797	.000	7.990	11.184	
	YeEx	-.723	.797	.368	-2.320	.874	
	NCl	12.335*	.797	.000	10.738	13.932	
	NS	-4.073*	.797	.000	-5.670	-2.476	
	Pep+YeEx	-5.630*	.797	.000	-7.227	-4.033	
	Urea+Pep	-.048	.797	.952	-1.645	1.549	
	YeEx+Urea	-4.263*	.797	.000	-5.860	-2.666	
	Pep+NCl	11.887*	.797	.000	10.290	13.484	
	Urea+NCl	14.197*	.797	.000	12.600	15.794	
	YeEx+NCl	14.318*	.797	.000	12.721	15.915	
	Pep+NS	-2.275*	.797	.006	-3.872	-.678	
	Urea+NS	1.140	.797	.158	-.457	2.737	

Based on estimated marginal means	
*. The mean difference is significant at the .05 level.	
b. Adjustment for multiple comparisons: Least Significant Difference (equivalent to no adjustments).	


Univariate Tests	
Dependent Variable:   cont  	
	Sum of Squares	df	Mean Square	F	Sig.	Partial Eta Squared	
Contrast	4400.007	13	338.462	177.510	.000	.976	
Error	106.776	56	1.907				

The F tests the effect of NitroS. This test is based on the linearly independent pairwise comparisons among the estimated marginal means.	


4. Species * NitroS	
Dependent Variable:   cont  	
Species	NitroS	Mean	Std. Error	95% Confidence Interval	
				Lower Bound	Upper Bound	
RK	Pep	21.490	.797	19.893	23.087	
	Urea	19.583	.797	17.986	21.180	
	YeEx	26.877	.797	25.280	28.474	
	NCl	13.733	.797	12.136	15.330	
	NS	14.030	.797	12.433	15.627	
	Pep+YeEx	32.177	.797	30.580	33.774	
	Urea+Pep	17.390	.797	15.793	18.987	
	YeEx+Urea	21.337	.797	19.740	22.934	
	Pep+NCl	11.057	.797	9.460	12.654	
	Urea+NCl	14.510	.797	12.913	16.107	
	YeEx+NCl	10.087	.797	8.490	11.684	
	Pep+NS	26.033	.797	24.436	27.630	
	Urea+NS	19.883	.797	18.286	21.480	
	YeEx+NS	31.487	.797	29.890	33.084	
RP	Pep	31.850	.797	30.253	33.447	
	Urea	9.640	.797	8.043	11.237	
	YeEx	22.967	.797	21.370	24.564	
	NCl	9.993	.797	8.396	11.590	
	NS	42.513	.797	40.916	44.110	
	Pep+YeEx	27.480	.797	25.883	29.077	
	Urea+Pep	31.103	.797	29.506	32.700	
	YeEx+Urea	35.587	.797	33.990	37.184	
	Pep+NCl	13.567	.797	11.970	15.164	
	Urea+NCl	5.493	.797	3.896	7.090	
	YeEx+NCl	9.673	.797	8.076	11.270	
	Pep+NS	26.913	.797	25.316	28.510	
	Urea+NS	26.233	.797	24.636	27.830	
	YeEx+NS	16.910	.797	15.313	18.507	


Post Hoc Tests


NitroS


Multiple Comparisons	
Dependent Variable:   cont  	
Tukey HSD  	
(I) NitroS	(J) NitroS	Mean Difference (I-J)	Std. Error	Sig.	95% Confidence Interval	
					Lower Bound	Upper Bound	
Pep	Urea	12.05833*	.797229	.000	9.26416	14.85251	
	YeEx	1.74833	.797229	.636	-1.04584	4.54251	
	NCl	14.80667*	.797229	.000	12.01249	17.60084	
	NS	-1.60167	.797229	.755	-4.39584	1.19251	
	Pep+YeEx	-3.15833*	.797229	.014	-5.95251	-.36416	
	Urea+Pep	2.42333	.797229	.155	-.37084	5.21751	
	YeEx+Urea	-1.79167	.797229	.598	-4.58584	1.00251	
	Pep+NCl	14.35833*	.797229	.000	11.56416	17.15251	
	Urea+NCl	16.66833*	.797229	.000	13.87416	19.46251	
	YeEx+NCl	16.79000*	.797229	.000	13.99583	19.58417	
	Pep+NS	.19667	.797229	1.000	-2.59751	2.99084	
	Urea+NS	3.61167*	.797229	.002	.81749	6.40584	
	YeEx+NS	2.47167	.797229	.135	-.32251	5.26584	
Urea	Pep	-12.05833*	.797229	.000	-14.85251	-9.26416	
	YeEx	-10.31000*	.797229	.000	-13.10417	-7.51583	
	NCl	2.74833	.797229	.058	-.04584	5.54251	
	NS	-13.66000*	.797229	.000	-16.45417	-10.86583	
	Pep+YeEx	-15.21667*	.797229	.000	-18.01084	-12.42249	
	Urea+Pep	-9.63500*	.797229	.000	-12.42917	-6.84083	
	YeEx+Urea	-13.85000*	.797229	.000	-16.64417	-11.05583	
	Pep+NCl	2.30000	.797229	.215	-.49417	5.09417	
	Urea+NCl	4.61000*	.797229	.000	1.81583	7.40417	
	YeEx+NCl	4.73167*	.797229	.000	1.93749	7.52584	
	Pep+NS	-11.86167*	.797229	.000	-14.65584	-9.06749	
	Urea+NS	-8.44667*	.797229	.000	-11.24084	-5.65249	
	YeEx+NS	-9.58667*	.797229	.000	-12.38084	-6.79249	
YeEx	Pep	-1.74833	.797229	.636	-4.54251	1.04584	
	Urea	10.31000*	.797229	.000	7.51583	13.10417	
	NCl	13.05833*	.797229	.000	10.26416	15.85251	
	NS	-3.35000*	.797229	.007	-6.14417	-.55583	
	Pep+YeEx	-4.90667*	.797229	.000	-7.70084	-2.11249	
	Urea+Pep	.67500	.797229	1.000	-2.11917	3.46917	
	YeEx+Urea	-3.54000*	.797229	.003	-6.33417	-.74583	
	Pep+NCl	12.61000*	.797229	.000	9.81583	15.40417	
	Urea+NCl	14.92000*	.797229	.000	12.12583	17.71417	
	YeEx+NCl	15.04167*	.797229	.000	12.24749	17.83584	
	Pep+NS	-1.55167	.797229	.792	-4.34584	1.24251	
	Urea+NS	1.86333	.797229	.536	-.93084	4.65751	
	YeEx+NS	.72333	.797229	1.000	-2.07084	3.51751	
NCl	Pep	-14.80667*	.797229	.000	-17.60084	-12.01249	
	Urea	-2.74833	.797229	.058	-5.54251	.04584	
	YeEx	-13.05833*	.797229	.000	-15.85251	-10.26416	
	NS	-16.40833*	.797229	.000	-19.20251	-13.61416	
	Pep+YeEx	-17.96500*	.797229	.000	-20.75917	-15.17083	
	Urea+Pep	-12.38333*	.797229	.000	-15.17751	-9.58916	
	YeEx+Urea	-16.59833*	.797229	.000	-19.39251	-13.80416	
	Pep+NCl	-.44833	.797229	1.000	-3.24251	2.34584	
	Urea+NCl	1.86167	.797229	.538	-.93251	4.65584	
	YeEx+NCl	1.98333	.797229	.434	-.81084	4.77751	
	Pep+NS	-14.61000*	.797229	.000	-17.40417	-11.81583	
	Urea+NS	-11.19500*	.797229	.000	-13.98917	-8.40083	
	YeEx+NS	-12.33500*	.797229	.000	-15.12917	-9.54083	
NS	Pep	1.60167	.797229	.755	-1.19251	4.39584	
	Urea	13.66000*	.797229	.000	10.86583	16.45417	
	YeEx	3.35000*	.797229	.007	.55583	6.14417	
	NCl	16.40833*	.797229	.000	13.61416	19.20251	
	Pep+YeEx	-1.55667	.797229	.789	-4.35084	1.23751	
	Urea+Pep	4.02500*	.797229	.000	1.23083	6.81917	
	YeEx+Urea	-.19000	.797229	1.000	-2.98417	2.60417	
	Pep+NCl	15.96000*	.797229	.000	13.16583	18.75417	
	Urea+NCl	18.27000*	.797229	.000	15.47583	21.06417	
	YeEx+NCl	18.39167*	.797229	.000	15.59749	21.18584	
	Pep+NS	1.79833	.797229	.593	-.99584	4.59251	
	Urea+NS	5.21333*	.797229	.000	2.41916	8.00751	
	YeEx+NS	4.07333*	.797229	.000	1.27916	6.86751	
Pep+YeEx	Pep	3.15833*	.797229	.014	.36416	5.95251	
	Urea	15.21667*	.797229	.000	12.42249	18.01084	
	YeEx	4.90667*	.797229	.000	2.11249	7.70084	
	NCl	17.96500*	.797229	.000	15.17083	20.75917	
	NS	1.55667	.797229	.789	-1.23751	4.35084	
	Urea+Pep	5.58167*	.797229	.000	2.78749	8.37584	
	YeEx+Urea	1.36667	.797229	.902	-1.42751	4.16084	
	Pep+NCl	17.51667*	.797229	.000	14.72249	20.31084	
	Urea+NCl	19.82667*	.797229	.000	17.03249	22.62084	
	YeEx+NCl	19.94833*	.797229	.000	17.15416	22.74251	
	Pep+NS	3.35500*	.797229	.006	.56083	6.14917	
	Urea+NS	6.77000*	.797229	.000	3.97583	9.56417	
	YeEx+NS	5.63000*	.797229	.000	2.83583	8.42417	
Urea+Pep	Pep	-2.42333	.797229	.155	-5.21751	.37084	
	Urea	9.63500*	.797229	.000	6.84083	12.42917	
	YeEx	-.67500	.797229	1.000	-3.46917	2.11917	
	NCl	12.38333*	.797229	.000	9.58916	15.17751	
	NS	-4.02500*	.797229	.000	-6.81917	-1.23083	
	Pep+YeEx	-5.58167*	.797229	.000	-8.37584	-2.78749	
	YeEx+Urea	-4.21500*	.797229	.000	-7.00917	-1.42083	
	Pep+NCl	11.93500*	.797229	.000	9.14083	14.72917	
	Urea+NCl	14.24500*	.797229	.000	11.45083	17.03917	
	YeEx+NCl	14.36667*	.797229	.000	11.57249	17.16084	
	Pep+NS	-2.22667	.797229	.258	-5.02084	.56751	
	Urea+NS	1.18833	.797229	.964	-1.60584	3.98251	
	YeEx+NS	.04833	.797229	1.000	-2.74584	2.84251	
YeEx+Urea	Pep	1.79167	.797229	.598	-1.00251	4.58584	
	Urea	13.85000*	.797229	.000	11.05583	16.64417	
	YeEx	3.54000*	.797229	.003	.74583	6.33417	
	NCl	16.59833*	.797229	.000	13.80416	19.39251	
	NS	.19000	.797229	1.000	-2.60417	2.98417	
	Pep+YeEx	-1.36667	.797229	.902	-4.16084	1.42751	
	Urea+Pep	4.21500*	.797229	.000	1.42083	7.00917	
	Pep+NCl	16.15000*	.797229	.000	13.35583	18.94417	
	Urea+NCl	18.46000*	.797229	.000	15.66583	21.25417	
	YeEx+NCl	18.58167*	.797229	.000	15.78749	21.37584	
	Pep+NS	1.98833	.797229	.430	-.80584	4.78251	
	Urea+NS	5.40333*	.797229	.000	2.60916	8.19751	
	YeEx+NS	4.26333*	.797229	.000	1.46916	7.05751	
Pep+NCl	Pep	-14.35833*	.797229	.000	-17.15251	-11.56416	
	Urea	-2.30000	.797229	.215	-5.09417	.49417	
	YeEx	-12.61000*	.797229	.000	-15.40417	-9.81583	
	NCl	.44833	.797229	1.000	-2.34584	3.24251	
	NS	-15.96000*	.797229	.000	-18.75417	-13.16583	
	Pep+YeEx	-17.51667*	.797229	.000	-20.31084	-14.72249	
	Urea+Pep	-11.93500*	.797229	.000	-14.72917	-9.14083	
	YeEx+Urea	-16.15000*	.797229	.000	-18.94417	-13.35583	
	Urea+NCl	2.31000	.797229	.210	-.48417	5.10417	
	YeEx+NCl	2.43167	.797229	.152	-.36251	5.22584	
	Pep+NS	-14.16167*	.797229	.000	-16.95584	-11.36749	
	Urea+NS	-10.74667*	.797229	.000	-13.54084	-7.95249	
	YeEx+NS	-11.88667*	.797229	.000	-14.68084	-9.09249	
Urea+NCl	Pep	-16.66833*	.797229	.000	-19.46251	-13.87416	
	Urea	-4.61000*	.797229	.000	-7.40417	-1.81583	
	YeEx	-14.92000*	.797229	.000	-17.71417	-12.12583	
	NCl	-1.86167	.797229	.538	-4.65584	.93251	
	NS	-18.27000*	.797229	.000	-21.06417	-15.47583	
	Pep+YeEx	-19.82667*	.797229	.000	-22.62084	-17.03249	
	Urea+Pep	-14.24500*	.797229	.000	-17.03917	-11.45083	
	YeEx+Urea	-18.46000*	.797229	.000	-21.25417	-15.66583	
	Pep+NCl	-2.31000	.797229	.210	-5.10417	.48417	
	YeEx+NCl	.12167	.797229	1.000	-2.67251	2.91584	
	Pep+NS	-16.47167*	.797229	.000	-19.26584	-13.67749	
	Urea+NS	-13.05667*	.797229	.000	-15.85084	-10.26249	
	YeEx+NS	-14.19667*	.797229	.000	-16.99084	-11.40249	
YeEx+NCl	Pep	-16.79000*	.797229	.000	-19.58417	-13.99583	
	Urea	-4.73167*	.797229	.000	-7.52584	-1.93749	
	YeEx	-15.04167*	.797229	.000	-17.83584	-12.24749	
	NCl	-1.98333	.797229	.434	-4.77751	.81084	
	NS	-18.39167*	.797229	.000	-21.18584	-15.59749	
	Pep+YeEx	-19.94833*	.797229	.000	-22.74251	-17.15416	
	Urea+Pep	-14.36667*	.797229	.000	-17.16084	-11.57249	
	YeEx+Urea	-18.58167*	.797229	.000	-21.37584	-15.78749	
	Pep+NCl	-2.43167	.797229	.152	-5.22584	.36251	
	Urea+NCl	-.12167	.797229	1.000	-2.91584	2.67251	
	Pep+NS	-16.59333*	.797229	.000	-19.38751	-13.79916	
	Urea+NS	-13.17833*	.797229	.000	-15.97251	-10.38416	
	YeEx+NS	-14.31833*	.797229	.000	-17.11251	-11.52416	
Pep+NS	Pep	-.19667	.797229	1.000	-2.99084	2.59751	
	Urea	11.86167*	.797229	.000	9.06749	14.65584	
	YeEx	1.55167	.797229	.792	-1.24251	4.34584	
	NCl	14.61000*	.797229	.000	11.81583	17.40417	
	NS	-1.79833	.797229	.593	-4.59251	.99584	
	Pep+YeEx	-3.35500*	.797229	.006	-6.14917	-.56083	
	Urea+Pep	2.22667	.797229	.258	-.56751	5.02084	
	YeEx+Urea	-1.98833	.797229	.430	-4.78251	.80584	
	Pep+NCl	14.16167*	.797229	.000	11.36749	16.95584	
	Urea+NCl	16.47167*	.797229	.000	13.67749	19.26584	
	YeEx+NCl	16.59333*	.797229	.000	13.79916	19.38751	
	Urea+NS	3.41500*	.797229	.005	.62083	6.20917	
	YeEx+NS	2.27500	.797229	.229	-.51917	5.06917	
Urea+NS	Pep	-3.61167*	.797229	.002	-6.40584	-.81749	
	Urea	8.44667*	.797229	.000	5.65249	11.24084	
	YeEx	-1.86333	.797229	.536	-4.65751	.93084	
	NCl	11.19500*	.797229	.000	8.40083	13.98917	
	NS	-5.21333*	.797229	.000	-8.00751	-2.41916	
	Pep+YeEx	-6.77000*	.797229	.000	-9.56417	-3.97583	
	Urea+Pep	-1.18833	.797229	.964	-3.98251	1.60584	
	YeEx+Urea	-5.40333*	.797229	.000	-8.19751	-2.60916	
	Pep+NCl	10.74667*	.797229	.000	7.95249	13.54084	
	Urea+NCl	13.05667*	.797229	.000	10.26249	15.85084	
	YeEx+NCl	13.17833*	.797229	.000	10.38416	15.97251	
	Pep+NS	-3.41500*	.797229	.005	-6.20917	-.62083	
	YeEx+NS	-1.14000	.797229	.974	-3.93417	1.65417	
YeEx+NS	Pep	-2.47167	.797229	.135	-5.26584	.32251	
	Urea	9.58667*	.797229	.000	6.79249	12.38084	
	YeEx	-.72333	.797229	1.000	-3.51751	2.07084	
	NCl	12.33500*	.797229	.000	9.54083	15.12917	
	NS	-4.07333*	.797229	.000	-6.86751	-1.27916	
	Pep+YeEx	-5.63000*	.797229	.000	-8.42417	-2.83583	
	Urea+Pep	-.04833	.797229	1.000	-2.84251	2.74584	
	YeEx+Urea	-4.26333*	.797229	.000	-7.05751	-1.46916	
	Pep+NCl	11.88667*	.797229	.000	9.09249	14.68084	
	Urea+NCl	14.19667*	.797229	.000	11.40249	16.99084	
	YeEx+NCl	14.31833*	.797229	.000	11.52416	17.11251	
	Pep+NS	-2.27500	.797229	.229	-5.06917	.51917	
	Urea+NS	1.14000	.797229	.974	-1.65417	3.93417	

Based on observed means.
 The error term is Mean Square(Error) = 1.907.	
*. The mean difference is significant at the .05 level.	


Homogeneous Subsets


cont	
Tukey HSDa,b  	
NitroS	N	Subset	
		1	2	3	4	5	6	
YeEx+NCl	6	9.88000						
Urea+NCl	6	10.00167						
NCl	6	11.86333	11.86333					
Pep+NCl	6	12.31167	12.31167					
Urea	6		14.61167					
Urea+NS	6			23.05833				
YeEx+NS	6			24.19833	24.19833			
Urea+Pep	6			24.24667	24.24667			
YeEx	6			24.92167	24.92167			
Pep+NS	6				26.47333	26.47333		
Pep	6				26.67000	26.67000		
NS	6					28.27167	28.27167	
YeEx+Urea	6					28.46167	28.46167	
Pep+YeEx	6						29.82833	
Sig.		.152	.058	.536	.135	.430	.789	

Means for groups in homogeneous subsets are displayed.
 Based on observed means.
 The error term is Mean Square(Error) = 1.907.	
a. Uses Harmonic Mean Sample Size = 6.000.	
b. Alpha = .05.	


Profile Plots


ÐÑÎv´Ãúúz*)!&&¦±±[¾JÊüü|æ;eÁeÚ>íñÖm LGù#±°xdnØ°ÁNùãNav$5L%uùÕÕÕ¤5ÜÄÒÒR¦ØÙrÍ¤ïÛ·^O8A)<â)))Ü]F;+¶æL/_N)åååÜóçÏ·-äÜü+W®´ó nÌv¶£ÝBRCn6~ÏbqUÙ«m»LÛ§ùò»Ï#­­­ÏÎÎ¦øÌ3G4kË×étÅÅÅÌæºuëh3''â$LN¦×Lq@ÀË¢üÚÌÏÏ§8S&+"¤´I¡tÛ¶T__Ïv2£W6ÝeÌ³zûí·SáÄ=ºT*e­h´ËBÈTh÷mÛ¶9v)Æûql1:ÚÙÚ¸1¸ÄÅÅÑ[K.ÃTIRâ¿=V¬XÁäÔh4Ì9³åÛSæh§íòäoù[¸p!Åèá9444Z­¡µåq7¦7z[ìHOw²Ò©ÐÐP¦RmÄògÍeqÂ¼EZ@ÝÝÝÌ¦EÃë5óçÏ'! õöö¦¸õálP(´81HÄlÙ?¶¹lÉö_ñ~nÌv¶6n.þþþÜËÅÙ³g©bÌ9ØlO£6äÈ¿1|>yÌ³Òe§üÙ£ÅÅÅMx¶ábã>¦-½ùæL½R|¼gEJaãX¶åo´ö_ñ~nå¦Ø¸1¸0CO,¡Ä1o?o·L®$òäïTTTlÞ¼ißt¢üSüÝwßÍÍÍJ¥6aª÷FjQ÷ÖÝÝ=¦-1Bnwø¬Lã¯ùíúë ãú86.Ý¸j2âÁ1KZ:Jóöc®*[yü(eBþüÆ!L×.¦O3°··7÷-ù0ËSS]]m0,)XÏÆo¾I9×			ar2ü^wÉiÛt:shzeªñì<+ëutCCCb±8**Ê1ù×AÇõqlqÔúÛñÆà²fÍ¦tæLâããÇ¼ý>tbùùù7oceÚ¸©?¦©üÙèóÇ`Y»v-ó;C/3ÔÀ1ùcUaöìÙl-ò­ô¦§§3»°3Û±CqmÛé»ñªô:®³±öùÙguLþÆuÐqnµóÆàÒØØhÑãÐßßíiCþ¬Gû~òÉ'i=gÒæ@þp?ùËå7n`Ì !!A¥R1oeffÒ£¬îÎ1ùÅ111ÞÞÞô8§Âé¹ÎNbQ¾ix:½ÐÐPr;Jß·o÷0Ræ9Ïg*ºØrì<«KÎÊÊ¢£½ÆÓÚöñÔþcãÒë vÞÔÕÕ­X±Â:w¬ígfîFæ.bS 0>ÆââbÓpókjj*mãÊäà0þ,Øºu+®@þOLL;w.ÓIM6á²ä@þ ò îÌ®]»ÊÊÊpò7-øè£pòùüAþ ?ÈäòùüAþ ?ÈääÃ$''n/--]¼xñ£1777**jæÌ^^^¾¾¾«W¯®««Ãùü'³fÍêèè°Gþ¸q8~üøcVÐÑá?È0yòG,[¶l4ù³'ÝNæÎK»'%%ÒfkkkTT¥,_¾ßäòLü­Y³^ÓÓÓG<6nQcÇ¦äääøúú>ûì³Lþúúú+VøÓØØÈëííMù¹õ|B¡R('¾Èä$ùJ¥þþþq@þfÌÁÖÞÌùùùq³Q±l2å¡//¯U«Vegg|?È0ÙòGÌÌL¬[·Îü¬Í·ß~4©á£¥KJ¡mR"_,/ÛÛoÃÜªAùü!ÄÊ+)^\<^ùl¿¿?7¥µµ6gÏÍ=hFFÆòåË½¼¼Ý||ñ]@þD$YÔHÒ¶`Áüü|nfë·ìIüðHùÅ~~~AAA*jÇm½eÎâhhNµhÑ"Ê°páB|?IOO_³f7%&&æÈ#Ù½7ý¦±ý=)?)Ä'hsÓ¦Mã?nL??Jä¾+Øü$£Ù!üÙ_ff¦Å]Èü"Ñh4sæÌ±ý=)ò·cÇ|`²Pù#-[ÆàQø·äÃ¸;3pxéÒ¥ÒañññÌ»ëÖ­£ÍgÏçÓæàààÆ)eÁ*ÈÌ?É%ô"44IÎý1añÃÂú-R,ä///OL-³fÍMþÍØØØwolldwgGûvww³L§@222 U¿GîÝæ7èííÍÍcý=)höà©Í¾©©©£É_ff&ÓÕoÄÝëêêV¬Xá=LLLw8!Ö¬Y3ölf¨GTTT^^¾Ès`~ah4¦ÝâÜÖoÙùäÏU?>3]PGGÇ%K(èÐ!Ð+ýþàf¶~ËÈ ®Biiipp°×/ùKf>ñâââ3f<<ïá:jë·ìIüò7MüòùüAþ ?ÈäòùüAþ ?ÈäòùüAþ ?öPÿ3føøø¬R,ã²ÈäÇÊ7±±±¸,òùàùòÇàííË ?S°O·þë§ÂþÑäÏ`0Ì9K¥Òèèh//¯¨¨(³ùy<ÏâÅÑ@ùüX¦wJ©#Ê_rròÍøøøí%$$°ù×®]K9ÓÒÒ(¾Èäp«:Ù³gÏÉåræ-???&¢R©Øü""Æ××òùÜLþìñÂ3fXç÷òòÂüAþ¿+ö(ÿàà i¸æÏßßòù<DþâããB!EÒÓÓ#""Øü7n¤HZZZbb". äòxüI$¨¨(//¯>ÏæOMMõöö^¾|9S ?`:Ê"üAþÈüAþ·ë@þ ?Èäòùü@þ ÀD?Ô­Fïb</üAþ@þäòL.ÚN§o?ä@þ umSÓ¼eN	¢5IöÈERRR(.J£££½¼¼¢¢¢(Îd(//¥DÿãÇã;üAþ¨Û¿ôôtJEñøøøðx¼&Cpppaa!E233q1!?ÀýäoÆì[ûùù1rÁÊ?Èàrøøøp7ÉöØ®Ï=ÆµCD²uëÖØØØàà`Èäò¸ÑÑÑ|>Ý¬««[²dµüØªÝÚÚùüAþÀ¨®ö~:A§mÃeí½QDFFæçç[Ë_||¼P(¤HzzzDDèããS__¯Ñh ?È8yßÞ®¦§ÇÚù?¢àÀ4Ê[¸ìÓ¹sçzyyÑë¡C¾Þs|N"DEEQ¶¦0;;;88Ø××799òùüÀÔ£8IòëüAþ?üAþ?üAþ?äò?äò?äò?àDFF³§[0¸?ÈL1FB*ZßÑ0ä¯³á'²¾=¸&`Djjj-ZÄnR¼ººÚ¶üá¢Aþ àr/öuÆÉ¥HþtîçýÇpYÀ¬X±YÕ£°°âcHäòùWC­("Û3ul³¯VuWÔø®[#S©ÉtJ¸Ùv[r]]]hh(EÂÂÂ¸ëüJ¥Òèèh//¯¨¨()­´´È+Wâ+üAþ`÷ío¥ÈxÛç¯«éi^ã¾ttl:ÿSÂ±4ÂW¯^÷Ê+¯pããã;::(ÂãñXù­Ï_MMMDDÁ` dv?ÈLC§¤¢õóù3ÕÓ+.@@×ØØÈMôóóc"**  ?å>FGG'%%áBþ 0yèuüEMOÉ$;ùo_Ä¶îVVÇ­Û1c=òWQQáååE²ë	ùüÀ$a4ª%¯ÛïløÇÖÿ$ù·>ßÝòÚ|ÁxåÏ×××l-Z´mÛ6Èä&FÐÓ)íúÉ¨#ÛvýäO);k4(pqÀxå/>>^(R$===""bLù«¯¯×h4!!!èóùüÀ£R5>¡è?BæÇ¤`gð(ò'H¢¢¢¼¼¼HæØQÀ6|,[¶¬¤¤"ùùùË/Ç%üAþ`!Ïëlx¿hù@þ ÏÂ¨ëïy¿»å9¶ÍÚ!Èäà9ô½ÂÞö)bý.äùü<­ênàç=²ü Èäà¨W:~¢µòüAþnVuL¢ø¹ý=p­¸í?ÛyFz].;òùLò¾½]MOÎúw<ðïj]Ê¨¡ãÁÿ&ù3ÛA£¼ËüAþS^×ÓÓò+IÇïP-öPì1gÂÆëëë£¢¢¼½½wïÞ=bùüÀ QUomxÉ_XXw5^VìD"QPPPFFÁ`)))?Èã,]ºtæÌ¦áEc|||¼¼¼bbb 0"JÙÙÎÇ3q)Óå/555))ÉZþvìØÁ¦×ÖÖ.òùsU«VÑ3cÆú1Á]fõêÕËÞååå$,ÈÏÏçf³~ËÈ`ì5>NxÓ¤C¶É)Aåê³«ñ²Óùóç·¶¶¦øF ãcÖ¬YtßÖÕÕQ$88X(RÒ+P§ÓÑÏö^9räEvïÞ½råJnNë·ìIü¦æy¯t¼ÖÝüõê`ºaiK³ôõwíÚµØØX±óòòU ¿qñÝ:Ð-¢HBB8cÆÇÜ²eË¶mÛØÑÏÏÏ`0PD£ÑÌ3Óú-R,ä/55µ&ªÛç[êBêï¾r»ò:®Æ4gêß=:IþH!`¢äoæÌtßH¥R¦°££ãòåË×üµ¶¶2-ÈÖ÷«Å½ký=)òwêÔ©6PZÚ<%lzW B¡0<<"¾¯6²²² ?Yºt)ÛÏ/  ­t¬ÏßòåË¹÷"·ÑÛÛÙú-RÐìLýGDO(eçq)À?Ô9Ê¦$&&r|äååAþ C79T*e|+22òQY(Åßß_£Ñ0í¶çf¶~ËÈ`Òèïy_ÔøVuL²ü©TªÐÐP6¥µµ5000''Ç4<ÔwÞ¼y?ÈÞÁ±±±¢½ZÌ cý=)?À$`4($¿·>9ÁÈÁãñ¸)åååaaa^^^sæÌÙ·oßhÈßÔßÁÅÅÅ3fÌ ,%%%Üw­ß²'òhôºÎîçú:ãHq5(áááôKÂ¢ÅÖáÑ¾	äàtÔÊ2QãS²¾=X½àòÇÌðÂu>£Ê!OE1p²³á'*ùEÇÊSçwèÐ!Îý~ãBþNÃ¨èù°«éiïx¸üy»o_QÈÀ9âgPôcÄ­ÏcõçËß¼yóHþ!é		_wó3Ò®D£Q«ð|ùcVòvGÿüfx¼o/.`ºÈßc£Ñ¾G.= j|B¥¸K¡þØc<Ï"åá½*'$$øùùyyymÙ²òç83F£Q7 NêüiÀÅ®#aaa*ÊZþV­ZÊÍÔh4ï+ù@þ`Ð[ïmÙ àj?2¼¤¤$kù³¨Ëå³gÏÆü9©Tùx:MCàçÒ®?agà0Zõý®¦§¤¢õªÞÑÑa!ÁÁÁ[·nÅN¿9sæ`Àt@);/j|Â<¼æñW¶Í)[ýÌ¨Þµk×bcc-ä¯²²2((×			ÙÙÙø b=ÚÃÏÏO.Cþ¬oOgÃãU.pÑúwªGòGÈMa(//ßµk×²eË¼¼¼N8+ùsfg±X@r>GuëÖAþQ'íúSwó3:¸¾ü	Âððpkùc©­­3g®äÏñ[¹·Èö("EfÎ	ùxý@Okd_çZ£A«ÜBþ¤¤¤ÔÔT6eÖ¬YôtæfvÇ4pQùóõõ¥«¸¸ÏçSdÓ¦MLS½<õÐõ®¦§û»ßvb'?q¾îf2¢Û¯rºÉJ¥eSâââ¾øâ¡PÈ¼µ÷î7ß|Wòç ìðn·¿ÈÀ­Ìì¨ÿ±¢ÿsmÈÒ^X34Þðm´;RèãëñmN7ù#^XrÅK.9s&=¯ýüüÞ÷]F+ùs¤¤$fº ÐE¿6ÜâóCþ£1(ÙáR«w´üá¸ü¹/?5F£º¯3®§5R¯ët³ü ?óÑiÛº[#ù#t©üþjjj|òI¦'múûû§§§CþnFUÕÙðø@Ï.83äà*òk±ªß½7äàF5>A¯®yz?«È3·sMM+%%%5käàu½ù¹òê?«È;É3+Ì$ÏçàôâÖçÅm/¸ÔðÈÀuåÏßßT©í#ùÓh47n¤¸[¬ù`£Ó4t	~îÃ;~pJã­ê³1æyþÎþFQZ/üPçôÂòññY¹r¥X,vVáåååì&Å)ÅáïÈCäïòåË#~ÁÌÒ?Ë¢_ìlx|P²Ãwp¹ºIysª)GCò'.oj¾ÿ#û7±±±Î*¼¦¦fÑ¢Eì&Å«««í<àiòGD¢ÐÐPf´/ýÔxòÉ'ÝâóCþ¶(úu<øßJÙy?Ï*=ÙQÿ³o¿ÀpþU¬2ìö´(åN	½µßòöövJ2¬X±"??"ïîÀ£äÏü01êúÞïüL§ipýmÈÒÞ;®QöË·©Ø>Ö©ø&ÝZYÿ¼ÓN	kîæ[aæÌLFGGyyEEEQÍÏãñ|||/^lÝ@lmouuuÌ^aaa|>M­ðÑ>>e+--e$råÊ¸ ?ÀbÐKÄm/¸þðþ7Ú¼×²þ[qiý#zéÜË=ÖeÖõ6û&''oØ°Ùïèè0/øÀæ_»v-åLKK£cÊ±zõê¸¸¸W^y8Zá£õù«©© ãD2;÷¿ÇÆùù¸ZÕÝ.ÁÏgÈí¹Ú+ÊùâÌryWmöåÒ¤Ä·	l?gÏMN&?¬-öóóc"**  Í/(¢Ñh|Ç6B Ð¦Eç®Ñ·qª¤ÑÑÑIIIøÖÜRþf¦z¸*ÅÎÈûöºøyÊ;ÕÕç^V|¤j¿®3êMôzö7$%ÉßÍª¼×½hó#?íñB¶vßú©=ZiÖécnMEEdß[Ê_PPÐ#G$;~~ÈÓr¾®¦§Õþÿ.º©+ûXEÚWTÓ/0pß	¥[ÍòWZ«ÂüÀ¸å­Ø³È?88h®ùó÷÷wXþF+ÜÆ©&$$,Z´hÛ¶møÖÜIþÄbñ®]»æÎË­a^±b»óü0èï~»Kðs¶Í5OO§46Ó^X3tyÃ W«Uìvä<üÅÇÇB¤§§GDD°ù7nÜH´´´ÄÄDåo´ÂG;úúúððp2Îôùs'ùcQ©T¥¥¥ÁÁÁ3fÌ`Epþüùt@þSA?Ð+·½`Ð»bëÄ`«¡òKuÖ+níTËÛ!àQäO"DEEyyyo±u)jjª··÷òåË*@Ç2Zá£õlYIyr~~>_ûÉ¼¼¼Ù³gcÀÀÐë:ÍÃ;ÄI®6¼C¯1µè9«ÌËuVÕùS(ògJUXXÌUû   ùLjeYWÓÓ.uVnCÝ	ÍùWåÛT=Uz£~ûBþäL±üY÷ùóóóE?ÀÔ;Vÿ±.ÁÏ5Ê[®sJ¤zeêÖR;6VòÎ£¬ÿ¦£üYtïÃh_K`ÔI»þÔÝüïÐ*÷yü7ßU6_Ô«ªòpQùÃ$ÏÁ èiìë3¦~éÛ>¾þÖNuÎ*Å½ãÁÖæèÈäÆHIþØf8Aþf&yL&:MCwó3½Níð½Æ¨¤@a«;¡!Ì ®Ñã<ªüy?¢]5äØºæÿ#÷ØòyìøÖý[¸ìJ~±«éiâÊ¼ÓPT³Ê<oK²¦â¡>ÒÚt ¶ÏÇÇgåÊb±×òùã¦_«ù¨¾ÚðØù4ìÕÕËîÈ$»Èü´ª»Srt£Þ$,Ö]ß¬¼7Ä?¥±sÞÜ]þØ¸Á`ÈÌÌÅõüAþÀä1¯à4®ÃtdxxXøâÌá¬ì5ÖÐT¾MÕU9y[îuj2*dÄ2ÔDBþY²u±8ùüÈpôºqëó×&x¸FOÂGÚGòçÀ¼-çkë¿îoX¹WDÁ)4tkqÃ¸:©Gá0 ¶Gþ(Dq©TíååEq&Cyyyhh(%úûû?~|g0fÎÉÄG,òóx<Å£òù?àZõý.ÁÏd&sxVaäj/Æçmë[VÄWüá«÷Ô:SCsB§ÌNùKOOW©T¦á¥xuIÈÁÁÁÉÌÌôõõµ_þÈü7lØÀlX8å_»v-åLKK£øþ!?ðù¶;ÞyæË÷$;°cîk¸ìîRv^ÔøJ~qÒ(n'«/¬¢×1áü)¨[É;í½EîÅÄýüü¹`@@rF[»9ölò9¹£pÊ&ï^FcmÀmäóü	x¢«/,s ¼û]Øñ^q.»aÔ·v6<®QUMÆÑs¿kÌ!ÈÕNí`Èî&ÙÂõÂÖdëÖ­±±±Ìz­¶µÒFWÂçæwà #yþëðÇç?ØëàùâgPôczZ#õºÎ	ÿ)ÂÌáüIòts«ß8CY|ý¡Nv4Æ O¢££ù|>»YWW·dÉký±î-<<<)));;»µµõQäoÄÂ)ÿàà i¸æÏßßß»Êgùó.÷ÖGíyýo¼%V¾òdtAàç=¼Ã¨7uUê¯oV:<Ã.ÚL%ÂI1À)--%Û#£¸@ ÌÏÏ·Öµøøx¡PHôôô&ÑÇÇ§¾¾ä,!!áQäoÄÂ)ÿÆ)oÊ3åO*ÆÅÅAþÀÄÚÑøBÅÿ¿(äï¥Íó<ÓÙËâ¨®ÐáZJs1nÌo¶8ôytfgèº%~¨qwÑ4!##cîÜ¹^^^ôzèÐ¡uM"DEEQ¶¦0;;;88Ø××799yÌ	bld±pÊêíí½|ùr¦¸½üÍ3¾fôùýõÕW/.»¨2èfßêÁ>¿KßÐ+®¡è?ÒÙðÞÁ.Â[T#ït]Iâßåw´ÇÃEð(ù#µ·íáççÇüü	â¯mTç8øãóÞîó·ùAÕßkpe<þ÷»Ö(¿=ðº*­1¥°ÿà·"¿5»ÏU+pcÈJùóöö¦/U,PÇãQdÝºu?0¡ü(§3»d­ÚóÜ;û._ùDÑRÀÂ"IW¿V«ä¾IÇïÄ­Ïëu=Î-Y÷ü«;&`0Ç°å+ýµUæ¿~ÝÛ½WîánÖÿð4ùcgý!Û£@ 0a'ûü	â_®ç5V´møÏ=	ï¼ßÒýüÍË/WT_½æNÉâ²d~¾¡l¿¹]ôÿö¾Öyâ¾|@eÀRW®^×ÙÝòT´ÞÃ;y[®nz¸ïDæp6wÛÕdzÃ÷Í¾-½Ú7÷à&Lüùúúêóù|lÚ´`ª0Ñd´7¯~mßÖIþþ°áè¦%_ýëÎ´^ÍF5v©eýâo:?mºûFmÙá?gþóÕ³/Tã½òY]mÕ¨&tèK5>¥è?â¬Éóîó4y¯s´_ì9ó5ô2¾b¶Ïßú¯d*áLü%&&²Ã;¸ÝþØÞ?0AX¶yMÚÿ<w<>j÷¾0wê¯ÿ¹¯³~ìU#å:]­¬?§§WsÝë*^¬,üéµsds®d,.»¸æNÉGõÕGÛ$]ä¸ÎbàdGý²³N)­«Rc*g¢æÐ­Ìá0*­±¬IõqNßêC]dg/ö0ò§Õ×é¦WÜ-©?"))iöìÙ)))¡`hh¨[|~È[³sÕqI?Ü¨=ùø´ÎhÌß£àÈMlQÊIøHûã_^¸|êÿ½ðuDInlÕU¶áNFÝ@Ï]?úðõ ±!KÿÆÐåC.>ÃÊpéÞÐÇçûÖî¦×´×u··(ø×»Íò×ÜåöÀsÕRùs_ nMRä^Þ³á?÷¼úÊÞËËJÒ«Î|VàÜ£¨zãy§Éÿ©8âBéèJèñ^½A?ð(åôÞ8oKù6´Áªúºtçk2Éù¾¼Ô_Ò¨T¨k´·¨ýú¥.¿ÊâîÁkíýî`Mdddyy9»IqJ±%£,Ý;nkRhùüç¼×XÑf^Þí­Oñ6_øà¹=;»Â<lo_ë÷ø·®dO-.»È!ù"Y#íA§mën~F*ZïðÎ:¥·%ÿ¡û<ÍÔ.Âk?Z½±¢YµçJÿyâ7÷¸:PÝ¦¶lÏUéLÝ=j¦Ïß¹jÅPnªéÆm¬©©©Y´h»IñêêêqI2ùòIÁäs· aÛÅ­Ì$ÏÙµ[8÷EqRäÞÝ¯¨<_7Ñh©^¤1ÂØª«%¹¤¸ÇèJh3¼CÞ·×1ó	5Ôç^VÜØ¢ÝtÁµáZ½rtísÃî¥CÂ>Ý¨wk·ÂÔÜOÿò«úø 9¼ÙaB?0+V¬`t+,,¤øx¥Í1ÛüM;ù£ÎÇÑ¾`¸z¢òç×ÿÇî×Vïÿü¥cÌhFGæòzú¨ýùûoHÚû§ö$-F/.»Èt%d§ó4réQã*ÅG.ìð`ó¯*î¤ªUR70¡ní¹jÅs¿OØ°k×]Üô@bªó/u|¿ÂGE'VlskÝ[;ÕN	YZnÉuuuL·û°°0vu5Óð²«ÑÑÑôh¢øFÙJKKr¥ÃòG   ÑÎ¡¼¼Nýýý?Ã=ä©ó;tèN§s»Ïùó4Jí£ö¼û·o­ëùÚjEg>+øpñWÇß9W_Ö2ù6à6³sÐL¯c£n@ÔÝòNÓ0®ý´cÝ	óÍÌ¼-.>C«7V·©ÓJß8ÞóÞ·½ÇK+UöÔÕèMmfÏ»Ñnjü¾æo@mNîÝÆ-´N	Ö¯^½:..îW^á&ÆÇÇwttPÇã%$$°f6Z¿Á@Éìè°ü¥§§«TªÑÎ!88ü"¾¾¾¸7ÜCþ>ÜôóCþ<¦Ùw´weÅõoª¶½xxçªãÑiWÊÇGÛ=Àú±ðÅ^aÑ¨¶¯~¡òK5ißídµæÈõE|åú»çkb·ÓþC¦û½¦òS½Ä¬z$¦ÚþíaùëWnLô£"è¹ÜØØÈMôóóøËS¥MÚ¸>FGG'%%qnDY´.íúEoAÚ8Û	þæÍGßÖàà ä<º÷,.»è@x#j÷/ßÙ?f¶ðgOüÇÔ#O>õ¯E9òiÓ]w¹2lÃñ;%²Çn·xNÓÐ%ø¹´+ÑNóÓ)í×uEo)/oj¾¨uåÁýºYÒYÉú¯RÍ»ãO­3	ÍnWÓmêß°Ke´ð:ÍòWÕecm70ÖÓ¤ª8ëNù¶e«¢¢ÂËË©´³]¾Ï~Ýl7çç H¶nÝùsùômÑwô?ÈKÑ«QÙ8ÞúõÞ?nÍ°3óooþ;ïôO·z6õäß®Üp©¶àqÁ6s/!#Wpi8v)#T]ïjzZ1pÒÌÌ"¼9«·vªÅ5z×ÌAzw¯Ssàê	ßyb?Ú÷m%Uø½æa&ùÈ-Ùìòn8 #¶¨Ú­EmÛ¶mÌÉ¸ýëêê,YbsÄsOJJÊÎÎnmmü¹ü=6ö­©©	£,(..6wdRL,ÖoÙùó<¶½xøòÁ²qí¢©¯Sµ=æ(«'*eÇ¥i8fººBÃ±¢ÿFUe;I^W¥þúæðºfU·a7é¬äÒ½!±Ì^9méÕÒ¾æpWQt£¯è¨èbWÑÍþ¢º¡é#³Í<ÛÈ`#ôatÈt¿øøx¡Ðüã!==]ËlÕ××i41ûüí½###Ù.7ççàããCÇ¢kBþÜFþfc£IÑ233)ríÚ5¦s@LLÌ#æ%>wïÞm1àÈú-R ?.ÛíÇß9÷áâ¯2¶äÛ³4âÇÒ®?õ´üJ¯³5R<lÌüÏ«úÈð²ªä7ìs¾F±þX÷ú#¢õ:×é2Ç¿î3ì?enöµ'§uhèÖâÅ4?DEf9¶ÎÆeËP4nùòåc1##cîÜ¹T>½:thÄ3ñ²³³!n#DNNÝ¦áþ¡L_QúY0gÎnë·ìI±?×Ü­/¤~»#ïQJ¨*©áý=ç¯K¾ÚµúØ¥×MépÝø¦ËüGïV¾sëêïJ/ý[ñ¹Ç/gü¯¼ôJqJ¡ôÝÕår>ÊZ«Úê£ÚêÓÒZÚaÑÉÙ¯ïêáßls©ÕÐÔ|¹²5%Ow´Eh³IÐâ@QÂÆæþViº¢­÷^k[Ó8ßk!ùÃÿwò ètºY³fþ3óýpk-j­ß²'ÅBþ<X²«7¿xõ¨áÝ_ì¢àÀ¼mÙÜ¸ëvöÁüí±·üz½u£°|~7««²n§_û äÊÿ)¾UpvÞÅoþóÇþ¿'Bò3hóWó¶¯¸qåö-¬­9×RÒPûæêJëwoÕ>»ºï\ïÕCMU·j¼uçXþ¿¬9ØþÖÖ½¹Ååw+êNõæ[u²2þF[oyÃÛµÒvë>ÉþPxà®òÇöêsn?ÜÜg¸¸ixZg·ìIA³¯Ë¢©/,Ìp¯¸iÄ3é¬þøòÏ%ó6_h«á«á6T_ÍÎAÃ4³X7«E])eg­	·ÕÖÑ+Å]°a÷÷iÝ)ýEüï×Øuè¶ÖZçê«í1¯Õaxþ=øL¥ü±½úÛçÏºbP£Ñ0í¶²X¿eOäØÉ@ñeS^OÅâfÄ1»x	3âøGù<¦+áëG6UüáD[)×z°XWü®y0 ×UæmaFì,1=ä(BÚGY?$¦Û<W_ÛiÈ]î ©¿	bþüù555)))aÇÆÆ2]Gé5&&Ùú-R `|q½áÎ¥©ñßâi÷eâo¼ÿYåëïÔÅV]eæ ù§3ÙùôÞñUÒ£»ÒÛ]aªjÚPÄW¦öÿ>Í<b7«JnÿÝQQjMMR³óÕözF®ê#$Ì0 Æ	pQù+//_°`×¢ED"ss[qqq@@À3G¦ïFY¿eOä8¸µ/cKþ¿âm¾ ¸Ý2º.KzÛ_6Ïálx8¸F_¾Mó"ÿPÿ±»ÍÜÅKHÙ9hãåëµv~l£YõHøHûZÌhË:µæj¼É=îLÓäo´>Ì"ÍÜeaÈwµL]t¬bÌQ¦-ØÅ|tAWÓÓò¾½¦áy[¹ÚqCÅï*ÅºçmQôì4LÃñ¼Óì4ÌTÕNÆù»ßßÃ½úlWõ'ÉÓÃÏz´goüÁ 7ÔµNÈÚµ?/¥DÒÞkb69ùEQãSJÙÙ>¾þQs°0SU³0SU¯¹SÂ.^2fÃñ4ì>¼	¾«ê«è»ªïìj®#Ço +ðØù4v¤@×÷ç´x¨sÂ>>>+W®ÅN,ÇãY¤°ñúúú¨¨(ooïÀÀÀÝ»wxüétº3g.[¶YÞ^###,X`ò¾uv!gÈð<HûHþHÑ,ì5ü»àBgA¢ÂDæ`Fmo´Ñpé¢lÒ°û½êÌ¶w³ÃT'6ûßø?eõ`ñÅeIþØÂw¯&ßÿ<1233cccXxXXw©_öp"(((útP¡PùódùòÉ'é«eFÔ2QÊ%K(iÿüÏF)SgÖ|þÒ1Ó°-ØhT·W½us÷79«d·vªûøÚ½m8þ¸ñîKå7óÍãy¿¼w·ÌiäêðÝåO5Ùß29Éþ»;åÁz3K£ÍÔÔTno.6CBBÂ;ØôÚÚÚBþ<Yþfîû¿¬óÑ/ÈVn·ó6_>mÁF½©£¼­`cQîÚûéCS2oVo,i47ì®ÿº'é¬$£BÖÑ¯ã6³sÐ0ÇË.2]	íl8Ìáª>r>G«ú Àö!ÁÆ[+6ü4]£§ðÌ3¸T*¦'rTTÅÙü<ÏÇÇgñâÅÖÄÖòG¯áááìR¿lùóç3ûÚã£Àä/  ¾ÚeËÉåæ1zôJq¦µ·´´Í¾À~dªÁ¯®íÌp¥þòD|fÀm/>å©mÁÌ"¼Öö]ùË¥¶ë×'^±LéÞÙÞÃÝ_^27ìJäc©^¤éJÈ63]	-Ýv¢ªúúzï5ñO7VºP¾Ó)(zðÆÕèUdÚþMÌ/99yÃÌf||<#mdl_|Ê¿víZÊFì¿k×®±MÉlµ<?Ï¿ÜÜÜGûæçç3uëÖAþ]½¯xÅüÉØñ@iêÄÞPy¾.åõôÏ_:VY£yÈDk|ý­êUª·%ÑUÕ¤Zo0ÝëÔdTÈþÌ¯ÿºçÀÕ§Øeº~Ý!øcÍ+Õ×~Yzáì7:®7Ý*åªºÚRïãïË]7Æ*n§Ü©øÜ)hÔø3-êfÏM>ÇÔËWºg"**  ÍÏÌª¦Ñh|­aájÉ) äoZË!|òIfxÅ)ùÊ£££]ùóCþ<È_umË^[­(cKþÖ_8óYû¶ë5¦KÚDååæ9ÄÍïÛ^Ðë&£>iÄÝ	9i¤TÅTõi¥÷%Y]m;÷þx¯i8þ¹Çæfç aecÝPHòÿïÀ¶üÙãìÂ§ÜüÖ7bÍóÐç¦ïúYYY??÷òù4	?éØá¬ú²7Z,Y7gâÖNu¿À@Â'¾(­7'¶.Ó±]ÑèÍ«n0½úÈÿF¯LlQÊÙãßÜ.úéµs?Êçq¶7NÐâ%?ð(òÇVìYägfêqÔÑäHJJJMMeS¹>òòòÂÂÂ ,sæÌ	ª­­üÈß0¦Æ»=æ(¹ +·³ðæ¿1Ä?¥asè4]MOË$»&î¸LÃî¦ÌÞµGº÷T¶ª3óÈÒdêS>ìÕGòçè2½5;UõºË.r§ª~ëþ­yâZY?åtø»ï#ùËêj´ºFàIòÏ4Ê¥§§GDD°ù7nÜH´´48ûåO¥R²)­­­999¦á¡¾óæÍËÌÌüy²üyyy¹ïWùüMâÖ¾3lñ0½vÖ»Ö¼Ê^cÝ	ÍùW%©:K¿_C¥¸ÒÙðøÐ`¦Ó¨Òï¶«Sûß8ÞóæIqZÉ _¤@ç#´Sìá}6«úFg4Vöóù½Q[Yÿ/×sþçÅO½T°kyñ/î©HþMõW/ÝMyÙF <¹¿P±mIå'ÿ¿ó¿.~±lÌ],¢£(¦¹üI$¨¨(zdðù|6jjª··÷òåË*@ûçñxÜòòò°°0*Î9ûöí³çË3°cóæÍR©Ô`0¸×çü¹z½Ú±ÎìÛ³âÎîr`ÇiãkÖw¦Æ¯¸iÊÛExÏ½¬ ùStÿàdÌs87>¥ºîÄÃuôë®ÜÚ']s¸^¯Õ+'ª3IDiâ÷nwZúMCÚÉ¿È-Jyá²³ÅÇ]>øÙù=oÞöòÉ¤eÇ7Çe|ünÖä¼½^pw³,£³:WR×Xý[^¥Ü8øÉ¥|y!rRÄþ Uôá/,Èß·Î°ýI!ÀÞºì~ÍÂÏ)8°c§°ØE>;3Aà¶¹©èìÅØExÞ2/Â«×üÐj©hwó3:À9öÓ«=^:øvFïïÓÌ»Í*g.¿1L¯¾Nól)«o4ºÔÊF,§§Ý¢pÎ'Ï<^¹ùAÕOHþ(s¿Vó£|äLüÍWÛòçalÉ¾±Ó>¤½?gWñ¨ý[ò[îtNÂÁç_UÜÜ¡6``æám/ôuÆG9é]e«Toíî7OOÉÄÚmØ¨®&³ê1Umã[×hQÊÿp·tYÅÏoÉüäo@Úh4èæv¸û ,²þÖòçÖ@þÜÜºó]¹öÑß2^ýúë½^øP¥öFÆÝ¯ 0AÅé5s0Ô¥U,aæáJvC×¹awÏþWvm9×wºR>á»ß_G½I 5÷ê3Wõ)M£ÞE/ºW½ï|öf¿û'ïÔ² ÿý®%R©4..ò&+õÝõÒE%[ÎÞøb÷Õ/#S~%UJ=æÖµçÜÖ_Èßc Gî2åÚ£s/+Ê·©º*õ6VæÌ5>¡_tà(-½Úô²³$¿Oëþ"_ZÖ¤Ri'Ë½HòzL÷Ýµªo×)+vtûÜÅü/HþøòÏKÖðð0ò7gÎfÌ/úüÉdåÑÿÎçÕäïyò3o)?äaS*ÌK)!<åp[0I©ÞõÍÊU?eï*6ØûiWÓÓ:MýÐê÷:5ÌÝ¿¤6'W¼Èó¤õõº>Ö76d¯ª,Øþ>Éß¥o>o¬ºWb4ðG05òb=ÚÃÏÏ]Rò&ðÿ¢îó^p=éÌµñxýÿ³êXìúË7Ûnvtè=¨G¼N£»qçóQ¨È®ÕØ]¡¥áÍ¨ø]eûuÝðê¾Î¸^a«weú"¾òãókwoË57ìvè&õÒè&ñð¼"Sû Wàõ»Ð*4êA&¨Urýü¾ÞLôÜá-µ_¶äóÐÒxÖbù]þÝ#ìP¤º;©5·v2#(P³Ï¼Cò§3­6é4ðG05òçííM¶'(BÎÇÌýãÊKúBþ<ÿ:¸´MÚjð^ü19ßëß¬M[Iø»ã¿LùÕO·Ðëk'^yçÜ[;>O¯â]Hj÷o¬h;þÎ¹¤È½g>+lÍË%®ÑßØb·åv²ye»<J×ÓÓò+¿1;ù5tk©×Ýóå¥þFåä5ì²Öß¯ijÊë5µ-®î!ëa²ÖÃÐ#ºÉµ±öÖË¬QÔf=¬áþIî¨ðÊ/Y£p¯zûWàZÎ³RùÜÃÑÑûÄwÙ6rÏy$ëZ­ØìPÉ:å@ÞÙHþúë¯+bÔüêV£w'h</KÏúÑT__Ez¸÷î>0òÇ.ùL¶G@`0(2sæLÈP¾º¶ÄL®ÐÜì»ßÿÙ]¿ ×ïUÆ 'Õ#á;Sy 45)ï£ß§¿¾4õyÂ°ÏJîH¸5óíÒ¤îRY((ò÷ßødÙÁÌ¬/kù)ç´ùo$*[.i-æm±VuWÔøÄ xëhæ§Õ«ÛÔÌ»¤$¤ã­³0sFÊÅ50³U¤Ü¿½ÿþT&åçzÅ-kc;Ü³"YdOxH.b?£A7ß5IÞý«UïóM"Éßý´?T§Ä¯à/ZùS©TÖDAAA$B¡0"""%%òçiòçëëK_gqq1Ï§È¦M¦zÚ¹¯Þø¸ðzÒÉ9¿1÷®½OiÄWê/«H#ùû0÷µ¼5LeáâäE+þwÂ7·|ÆTÞé¨vÍqÄ½¡*¿/îY`Á]wwH/ü¾«2¥·§Nja`¶kÂCõ5k[êSYUjm:ÿ°9ò^zåíoJN^?PV¾¿ª2µ¶Úñú0	3×Ùg`æVNÖÔÜoîÕÇ,Ëa0NûßÙµ_¸iö­½|ÿôÆ^"þ¸÷/½±K¬pJà¬9qòg½Ô[jjjRRuî:¿µµµ.üyü%&&²Ã;¸ÝþØ¥!` ?¸WRú §àúß_zïAS.9ÁðHH(»e]µ¢Ú|þÅ#åÊÂØc/=·ûYòÂ¥©ÏÓæ¦óïÙßl4è¸CB3ÙYfÑ9©+<ýù?ïüðW»öÆï)>³oD³QÖÚðYcí«Ý9Üóá7ßË©x°+·öïwO_Ý$P($SSÆ¬À¿×¬ÂAWàuSúj/5ÛJüûüéz¼zçRº³ðGÀilí9!Ç)áÓý7í?¤¤¤gäöòò¢8¡¼¼<44ýýý?>¦üÑkxxxGGEùóç·¶¶ÚcÀå ÷=6EJJJ(B"H7[|~Èÿõ¼ÿÍ´ÞÄíÛÒxNÒsÇN³P1ÒF®ý»nq[	[/Öñ3nÕ¤ÜÞ_P¾ûÊÏr¯nÉ*ø ýÒ[yÅüýzé'¥åÛoV|Quëï»êW~Ém»ä6MZØõaÜÎaýmýµ_÷_X3ÄÌÛ2Ô¯¾|°lë¯$¯áUåñíY,ÎhT÷¶¿ÜÓiÐKLßØ=pu`ÄÝ)@©5µ/ËÁï>U,*I_Ã7ïxõÎ®ÿ:äufânÊË:åþ1í+éééLCm||<#m</!!ÉÌÌL___äïÚµk±±±l´ûAþ<JþÜÈ[S;éó^xýo§®¼O:Uwç@íí=Ü!ÜvI³î%ÖÒxkcÂâÑºõ÷ñÐ¢ªèjìª½Ñp%óö×?ÿðülçÂçv?ËÔ&åt 45·îüÍ¶>è¤oÌAÚWwÂrÞr¾;¤ÆK»çA!:m[wó3ýÝoKdCE|åú×îN:+¹toH,ÒÚ5f®¾Úó}í&í´Ù`4èº»ËÓù_>8þÇÝ+ÈðgþÚ|îïw¿ZI"xùÒNFþ(ÛÝäßà/°Ç®Øi×è-áá%??¿?0Tªå¸|+7É) äòù¿æFm6Â-Y×·µ·^éh+àòìUô2NÒ«x;>ßx6ñµ¯°^ì%Ja½pÌÎêAó`qæy[ÅcÌÛÒÝ$9óYÁ¨ý¼Í·Û-ºÞ^ÿÔõê]dë¿îI)4ØõÖlÃVõ=<vù²]ÍuóNÿäJÆÜ|Þ?6'çàÏî<B¯sÎù¼ãsóOþäò)ÊÃ;0'çð_¿yäï'Ó½ÿò¾fßµ'õñc:àããóQÂ¯'åH$[·n%³ï »)ÃÃÃ¹)!!!YYY?¿ÇÆ<¦³­¨¥ñÑh`Éöêî¤*äîrþäydä|¬2?yõB¦s¡¹¾°ìþ]ç_5ÏÛ"£&L)S«Øs4åõôÊóuJ¥ö^§&÷æ¡úÚüò|fF6õS^³fQÕ§óÀª>N£´IùÅ¢ã¼ìÏÞ8ö.§þxaÿ;%g>¨.ú+¿ò£úêÃ_ïßJÈÙÃKûÉíµ±àøh9G-J9þbL¢££ù|>»YWW·dÉkñ²nÕ5wàKJJÊÎÎnmmµ_þLÃ]¿RSSÙÄÄDî¼¼¼°°0È§ÉßQÀh_0áOS¶¡îëÆûß^ÿÛéâ¿ùõnzÆGc½ð`ÉÁ/w:üúÝ#/7¬ëãOYúük'^a½ÐÎÁÈâí·õÆ~óÎ¢¯6m­ºúï=Òø¨ì²WÕG¶'k«Weó¿lH»êóçï¥¾*8ó×ö½¤ZEßxl»qäÏhÐãÿ>ÒÒR²=f¼@ ÌÏÏ·¯øøx¡PHôôtvt¦O½F£IHHü©TªÐÐP6cê;oÞ¼ÌÌLÈ§É_PPÐ#G$;~~È»c4è$=wÌòWäFu~ö k©sVá×<|Þ«ujDp³í&y!;s¡ÁÈ¥-¼ò^¶a÷Fcÿò×N~øÖ¨#¶O¢M=SM·¹ª¯KîU®AÁÍÎkGÏþínÊËUÛÿ£þDB[Þ=g(ýÑç2òÿõÀ6sçÎõòò¢×C¨kôÈ¢<!!!lMavvvpp°¯¯orròfYÜÝ,//£òçÌ³oß¾Ñöî'b±x×®]to±8öì+V466BþcÈT_]Ûã@¸|í£Íé¿u`Ç+õ]ð:ê¬ÊÁ6`¼ïÛêÓ¿¸÷å#ïÿëÎW¾ã?B¶,üÌìïéîÝÇËk"ÏÖ*¼[tþxÁi¿t¬<³F)Ä*7¶ª¯¡Ï$sãÉJHæu<Ò;<R=¾ô·IþúêHmìÛ×Ïbm7BõÙ?v¤ êÁ_À£Êß÷õ*Uii)ý1c+óçÏOOOüqÑ-ëLù?vÜ^ðë||õ ±!KKÎw³²«RogãD®mÄ.yaKWF+îµ»ë¾,Ú¾3ùÿñÍï,þüÛRÎíctt8<÷¯êc*öºJO¶ÿô^ê«Õ;òÅS\-k«WÅÞ´ÑbÎEBÃí$ìHA­êÃ_ÓäK^^ÞìÙ³1àL2÷ktuÜpßóïãëo'«/¬ª=ªwÏ:úu2Û#výG:WÉ/ZïÎt.¼Xzyç_¼¿d×Û¿ýtÍ§ëN<2îYÕÇÏÔäu¥ÖH`&^iH6)Þü9é¸hö¸ü©TªÂÂBfd8·# ÅÏ?ù³@¯1µ$*Ç½/g*æ?óÄ¶FìuýÝow	~¦ÓÆ,V§ÑU¯Ky=inïî´0ò^ènUZE¬­[±G¯§Jw`|äàiògÝçÏÏÏ/66þäoLäÚ£U[;Õ|ÛwjC_RØÿû4»¦bÖë:Z#û:×q^[­(cKþÖ_8óY¤½ß:;tÂz!3èdË·J¯W44ßºÃ7Ïhí+#s+öÒß¾òrÍîõ'¦°bòpuù³èÞÑ¾ò7&F½©«R³òbÜÿF«°k0^VlÌü,pÌ½tAWÓÓ½®À«ÛöâáÃ	Yõe-c/§3è:ûÕ·ÛäMµUei¥ÁÈ¤ÜÁÈøÕµ=v®ìD=qUvËùOùÇâ¹¶Çg8kní~q¼Ì;R7WàÀiòIÓ*cZî¶ö;ÿ9°ã¤Í£ì5íóÙ9iØ=Y&[ÿuÐ¦ýËo(egEOrÂòÝbqÛc«yLcîÕwKd~¥W;´äõBB®(M=Sé/$ë«+è¼v[±×÷¤&OÑQ7µÇs>ÜôIØdûñf:Y^^ÎnRRÆû(wL	,úzqË©¯¯òööÜ½÷yÈß±À$Ï`ÜOkõ w)ÞIÂæ¼þPâù69,v³ªä¬±+ïÛ+j|J£ªròií;óYÁ¶Ó+ÅÍI:¹3_M·9<B¯>®î,ú|ÓWF=öweäÑ#³3*s'^i>û7¿É¯Ø H?y(jjj-ZÄnR¼ººÚ¶´9G&,,,L¥RY,222P(HIIü¹«üy?0AhFA®½ð:Ö°kÑ¨ÖÛ^Ðë&jR7¥L]Y³ÅÔ5ßÜÙ_nhLè^¦s!wedrAf0òò/ÿõý/=wà·7vþºò³¿øõúxò<+V0«zR|LisØö,6SSS¬3$$$pW«­­]¸p!äòùLh`æmsÞníÉ2Ùybv­!áëiùÉ)àD¶ïªúÕ¢ú÷ÿéÌç/+:V!(&úªZÏ¨Õon§ý¡8sÓ·g7í½¸;èìpìÁÈ?0µiLwÚpo×ÕÕR$,,»Î¯T*öòò¢øúEÙJKKråòg^¸££Ã"Ãüùóåæ¨òù.Qoëß5æäjGÌ¡Ò+U®v?Ì¤ßµôjýèZõý.ÁÏd]¦´#÷ê´÷ç¥lýõÓ_n«9ñÜ¥ÒÆ5£²Z§îè°1Ùbed×ùó|úÆì;N	¦òf²W¯^÷Ê+¯pããã3ãñx			¬~Öç¯¦¦&""Â`0D²Jg[þ®]»kÁF0ÈäòÜe¯±îæÂ!î"¼eúK÷¶ë[s¸â¹ÞY' _mçG©ê»Ýe»W3($åõôq§*²kuq/f+öØ'bâWFvÁÈ?àDyÅÌk~~~ÿb¨Töèéctt´Ecî²ÈCòGùüAþÇÂæmÞ^múMÙÛ½¿Oë>|m°²U­Òâ­]iÕ÷üÙdóæª>¥½uõâôò¶þú@^JÉÃA!£`1£òÏ°1Ô;ÄôqJ¼òÆñt·òª§ã°­_¤nÜa£ÏnÂððpnJHHÉè÷¿ô¬¬,ÈäòÜ­âá¼-#æÐê$yûHøþÌü5tk¥3ß¨uÒ®?9yx·ª¯Ga^¥cüHÚûsvoÚâýó÷¯7ôëÉö^wéUôr#o<øÚWÆ;ò¦|íÉÆ%!!aÑ¢EÛ¶m³_þ¤¤¤ÔÔT6%11;à#///,,òç®òyþÀ4¤¯¿µS³JQTc1C"77ìnÏ2ùÛ°k³z3w8TÕ7º÷In¥Ø³óÓ_oã­ûÃí/~ãâëg8æ£Fæ®w¢òkò¶ÛíR¥ÔÁ¯Û ÿêÚÅÉÏü­8¼,ÿÁxå/>>^(R$===""bLýª¯¯×h4!!!vöùcP©T¡¡¡lJkkk```NNix¨ï¼yó233!î*Ü)ýF?Ìó<ð¶ôj³ªäïÛûÆñ//õW4«&¤ÏB­Tw;WôqBYÎ¨ê3YÍ¨É¿±Í¸÷/^Iß|öe¿ pÀ]okþMçßûÝñß>»ëdodÿãéõäßÞÉ <üîû¶+ß9÷Ök'^9Xzv'×¤®Ô_ÆL0.ùH$QQQôh&cGÛð±lÙ²äçç/_¾|ãñxÜòòò°°0:ô9söíÛgã$«ËKnn.«V­¤Mz¥;R!`TÂþÉWîÙ>%e¯ñÞqËExIïªÛÔ®ðý%]V2ØÐ­Õ&é*)eg»V+ù¾ª>ëWÆlÆÕ(µ×¿©Úµ?5þ[¦-xÚÞç·Û+ÉÞÈ?Ìý áÌ+/ÿéöÞÌFþêÚ35G ¿D¶GvÈ6ûÞl»¹8yþb¦LþüüüHõtºïÇ÷i4JaAþ½%½ú½8þÜGÁÏx&F½©÷y0ÇùWYçmQ¨×ê_^ê_¤Ë¹>ÇßxDúÞïn~æwè¦öÁñVõ8>£!ýíÎkGûêìoÆ%¬<_¼÷É²y)%~%n½Aß n`#ï¾úå¦óïÅgü!jßüüÐ§?[°òèÿzÛçäO¦ÄELü1µÄ$|ò>`¼¨¤Æâwo£åØJc÷yr>z·ýºN£21kìnÊìó¤xÑùß$4ìà£Fu_g¸í^â`jÓ¹ª¯u`Ìª>fbü,?NËÎ-ù.þêÄûç;A çñí¿åt§£zÁgü&>»ëC!ÀÔÈ¿¿?©^LLÌÐù/_ºt)¥P:äL_ÿFöívù#"m0ÜÜa®ê«Þ«h7T·©Sû×ÝtVrºRÞ$ÖNó1è´mÝÍÏH»MÆqO¡gÒèVõÕözF¬ê³nÆiö¬IEÇ*¶ÇÝýêòÌ&´I[Iõèmö%=ö®`Êä¯°°pÄ£?àúò§4¶èy¯Ý;§É¿­ØÛ÷û´î/ò¥yµ	±k'ÚÿÛÞ@5uàsx9<Æ×ñ¼§¯¯Ï8ãø8Æéuì C©uPkºT-J)uo]qEDDQRERd"(,a_C @6B¶÷¯¦IIøÎ=ËÍÍnRóí]Eå¿2äôÎn«]ç¦>Ý¸¹gìé+³aoä/³Ç:dÇ^IzÒÑÂÃ[ZÃµìþ¾ÛKñç÷ðì|¿wXV¼±øS¼ºøôéÓ---ÍÌÌ¬¬¬fÏÝÜÜl?âo,Ç_,çl÷üÑ1¼alj¾tå¥ùÝ£åt>÷Zë÷;½C,U4ò9Ü&å¼=Ê¿Ec7.³a¯&ÁÀ¯/%^i(eGx&z,ÝSú¤oluY5Yë®¯¡øóJô,caÀ?ãøñÇ%y·ðÎ:~wûÕÖ/ÃÙ1¹üvÏh;µíÑÄú¤§¦ß=+R(ê¼¬áUæ²GWÅ/¹æòÆï1 Â®î¤ 'Ç.lÏ+Â¾`?XüÚåßvG®à]wéüêçÈ¶áhØ±«M.ãsê×µÖ:ÒHß3ÄâåÜ»1MQ×÷0gã­¯ñjÎå÷ÏÐ£øQe Ë-æfq:.Þç?èûC]í>+W®d³ÙC¸ð°°0)ÌÇsss³¶¶6776m§§'^Ó¿üüü3fXYY1gøN81<<ñ#CÔ#§ø;Þ¥gâïÝùa«xgöp/ErTÞàÙRÞ½£êÝ¶Go§wHE<AsyKÎ÷µqgK·æz/-ð[Éºµ¿1=´£<Cy~Øt.¡BÙ÷ýÉv®¸E±Õ!þð<èï3Õ¸L&zõ.|îÜ¹ê·úUý8''§ÀÀ@æ¢ob±xÏ+x9L9þ<«ßÒ÷óóCüÁpÉå¯	jºæØu|GûaìÚ¶kò®Õó/tU×M·B»^Öõþ¿¨[ð¨±ü7üê%ÂNVVKö-Ö÷¯l|~zIqàÆêØk]AÉ¿ÐÂ®îÌÈüSÁ§W]£úñ ?þCµ4úÏÃÃCzñx¼	&àå0åø4i½üùùùªøKOO§ñqãÆ!þ`XÝ/:Òý/~è?».­ï¸óÏ% ¥¶­'¥DøMX7Ü±ëûüÂän¹Ô8þ"æî"ÞCAsy[ÑÚ$ÿ²ð]¹gìóýÓHÝ¶ÌxaösyV­¢;ø;ðTVú¤ê[§Ý¥1u³8ÄééîæÖV&ÉÀnzÚ[®Éd2+++f¼½½éÒ¥Tivvv4®?,,ÌÒÒráÂÚ;uÞÀ×ÖÖVu«_Õ666^^^xYÇPü©n¨?z·áÞ¾0NnZÉoy.Uîö=ÚåÍ§÷ãì[º¢>áçtóãbÊ¯TçTÝû¤$ü/E^Q±×ÉÊR^QY,UÔw)Oà-lQ°ùXé9uÜé]nåÝ9Fnø3%Riw[ë!x]µ:s>ýýý·mÛÆ|ëââÂDÕjþ6Ð!!!4Câ/--Mµ+Y5CNNÎ´iÓ¦LBKÎÎÎÆKlâñÇÙÚGñ'wìØAãô&@üÁ;rýu$ª¸ã#ÿgÊ$b§PXzÝ-ßoy¯CÑÕÅåÑËZóî_þx~EWôãm9D8ïõ'$bINá§S®eÑ¾àúú¬¬Ìuþx"¸ÜXùPW3aÂê9ïõÉpÖÖÖÌH$RÝfklTÞb>»Ç¯½õÔ£ø£Ô®ÃÌÌL___ssóë×¯ãå0åøKJJÒùFaÞ?PuÕäö¸³&ãeîS¡KÙ¿-ïbö­Óãgô	a#ðw©_Q¹äúiºû´¹òO­ÇrzXª¨éPnê+nUnêÉñöÐõ¬.lÿÝÏGx&ÅÍâÎ§£Éúï1ýéBÕýWÕç×Þ_§sË©­­µµµÕóãbâoPèÿæÌÃíkii9cÆòòr£øû£kCN]KÅTþ?·úþQö2ü3c¥Kñ÷·/²~¿ÿKhÙyv8þWTfn¦qEeæôAçw?>¹M¨ÜÔ÷¬ilÕ7-<c¹@`¨³¾£~$nI7Þ!c<þTö4æïìTnÅÚ7eí-þG`` jÊ¸qãd²¡:ÖL6þâoTr»Zö(þg@ÊÛþWÎ0ñwcï£ßø×~©ÿKèWÙ`iÀ»8¾T~bá«µdÊ;ð>oR±!6õþr¼ºYË-Å¸Y þT\GÏ7O5ÿ;h$$$ÄÝÝ½ÿñ'æÌ£âìììããÃ,òóósuuÅËaÊñ§¾ÑÜÜ<cÆ©S§"þ`XÉ¥ôC¢¸Oùïo>µ_ó`$6ö¨6ìQáQçQíøVir	·yWså$âåm9[xk:°©o±«Û"<,<¶ÿî¿@  þÃ±³³3775kVIIjþÀÀ@eË1û¿ð°°0Õ6mooÏì´¶¶Þ³gX,ÆË1¶â^rí#¦)GJñ²õ0¯aXNù¤£¤£°£¼£ÈSß°G	hÀ­Òä2~kÝGÜÚ/äÕ-§¯6õá¨¾á"ìêN¹=Æ/`@,âO©S§þL/ þ`8Pü=üüðP-­ßÆ-Ôq£2ÆÙ°WrÍ¥*î8ûyôàoÖ#*i/väf*³¯¦Cyb?âfq?Ä!ª««Í^Q:¤Ê/""ñFBve[á½ºå+*WÜþ9?Ã½K%¹Ò'9Òü"å®^lé27£#èÍ`îÿc1þT(õb/âÏ$É$âÖ¼¸W»¿æ×õç)=ü¶Îªï+c¼¯l|vbÑÀ5Ì¹¥$Â¡¾UE^«@QÄe½äåt½À«öÆ17;½êÑ] `TÄNíííÎÎÎ<1==öìÙÌ©W<y2M9sfbb¢úÌÚõgâÏdHE¼â`ç¡Ûñ·mÿ¦Úu¨º¢²òÂ+þ+¯Ô&m+¼÷+*9Dyeæ§­</»Ê^*iÁ«6ª¨_ p¬Ý,2eÊª+cþ4Îé§éÓ§3W1c8::^½zFüüüV®>³öCý¢ñññ`Ê¢<_~·¯åÎeümÙÏÎÎÈóûgÃ³øÒì´oË£læmç¿f«N¹ÚTÒV_>ì¿VG§°#Éo=mè®`w²YM¬÷ZjÝ::ØxÉF§zVSÜùÔ#K/|vóIt.·uÃñF³fÍÒ>ÛÃÚÚZuKYZZ*^Ý¹t¤X,Ö¸b¸öCý¢>>>÷atKLÙ£¸ÿÃás·ì>º%âïîc®?ì~ø|G`ÒÁËiB²÷[¨g	þ9Cøû?~ð°*5Oô¨²ùaQnò÷<zâWyÙÎxqG¿?Ü;÷ýÕßx,¹ðÍWa	±X'0L`ôñgaaAµÇf³'MD#Ô|Ìµ6mÚ4Åfdd0KP? PãàBíú3»G­ÖViÀþg]KÏV°ûäàÊ¤Íþ_Öýìå9÷êþ/áûëCqàÕ§¼-G]§ê^aWÅÿøøÞÈ4²ËÍâ~üP×:wÎç¥ÅÒg½ÎDàææfmmM¹Ó¦MóôôÄëbñ§ºå3µ°X,LF#¹µKgg§££#³íP÷±ÆyIÚõgâoÔ¶Êï®ô¸óAeìG-4ÂÄDX³¼¾ÿKÈ¿<¸#ý<ª£qoÇ·õû×wï#Äç:åÌÜ,çâOc±sçÎDÚ?ÈÉÉ)00P"Q^r_,ïy/©Åßøñãé%OMM-))¡¯¾ú1øàúúúõë×76¾þî'2×¾ó öCýø3%¿ZÆ¾5/îÙ¿,_ôå?<5|z`Ø°L®sSßkrIÓçìÚeÒ¼F&àEjÅ·¨^ÁÍâ`,Çö­Þ¨ð<<<´gÐøôçñx&LÀKcjñçîî®:½Cý°?Õ­$))iÑ¢El6[5eõêÕ/_¦úêèè¨>³öCýø3Uo¯a÷mÉ5gäm~·ì´KÙÿ[òòÙVþv(·T.êK(ú²´7õ1¤võnó.¹W1)ÍXßTì¡"h./¸ðÑ1^ý?¹páÂ´iÓ¯®È±téRª4;;;gfÈÌÌ3gM8qbhhhñG_mmmëëë5f°±±ñòòÂKlâñG¨ý®OOO§Az¶¨)S¦¨$MIMM4i-sòäÉ´|õ7öCýø3]Q)åSRrï·Ücçx0ëJê[hJÓcSÊ'ý½~ÅîÎRn× ~³©¯°åõ¦>î==ÝÅM¬ßóÚ¿ÁKcªÅ^uÙ,K°NÀ`â¦!Ô¯Nª?þÂÃÃµ...L´¹¹¹©¢-99F"##µïÑ¥3þÒÒÒV¯^­1CNN%&Ó£££³³³ñZfü/Ä±£°£Â¶AÁùµ]$u?¹^OUCGpLÍBçÒÏ§¯­Å%UüßóE%÷õ¦¾n½Y <½ùåÙ5¡»c<í.a_0®u­øSøN1×¾P¼º3B-8iÒ$=ËÑyûVõ(þ´iüèÌÌL___ssóë×¯ã¥Aü!þ ÝEz­CÁÇ~<·¬ëú2ú[õoêM.éd5±þÐ#*Àk8¦pê¸ñçÒ9í¿ËzVos´ÿ%ÉTSÔãLçEy9ÅMÇª¾­­­µµµUô~paaa¡öuÖÀâoÆÌ_gÄßFÉÕÑmÀ@ñWwùx?gÕònÜouõ­ïÒ´z?çÈeQné¿H¢¨éPdÖ+Ïçhöy^¹ßZëHà$bINßë§W]Ã¾`x³.]ZRR¢ú¶¨¨hÑ¢EÚõ¦½WWñê>èèèêêêþÇâÕ¡_ª)ãÆSmbdæê0JãoúôéêÁ§b7üEü|ïåÕþú,)·Ö²ólÕï>®»¦ól¤4¹Tþ´^ÑÈSnì×§>«õnË>Þ¤*¯!lÿ]O»K	Ò9uyTTo4Îb±/^¬º¯©z®¹¸¸ÔÖÖÒHxx¸êìLKKËÒÒR±Xìææ6 øDsæÌQMqvvöññaOùùù¹ººâ¥1µø£Î£Þp¥oc9þ^ã+ê?®Ge³·©ø¯¥õ+v·û×]X¡ÿdánÁ£Æòßð¹×°þA]GâïÄW®¸Eõ¶/¦'=É¡½·+"""Þzë-sssúÊB;×8Í3kÖ,ÕÂèèhñãÇûûû÷y;0ãl6ÛÞÞÞÊÊòÀÚÚzÏ=Ì×À¤â^ZõÃH`ñ'+ØEnòÍ5ªkõÉEâ®[÷·¬üíª©­)94Q³¹W©üºO°òA'Twïåá§W]Ë.ÔØWèrk Ã?úÒ`Àih(eãE!¿ÌÌL¿mÛ¶ãÍª£¸£)çØ_Gr¨ôÒsT¸¤â¯f¡sÙ/Ö.qíé©jPÈ%ÜM¬?HÄ,¼dÐ'Ö³º íâÏ¥r_0%#ÅV)¼ùø#o½õö©à8á¼±D,ää'0°¿i8ÓÇlÏïrÞáÄ§h$'&òêúu~®æ'<nÁúõ?jnLkH¶å§¦joèMOñG	xÅ-ªøQ¥a7CüÀh¿iÓ¦á&NÍBg[9­Nõ:ÃüÏjþøiÍï×ÕØ~ZóMêqà×cNï(9Øêq[>éï¯7ôë-$É.<½êÚ¯¤æs?0Êøc²/77×ÿ~Äß¨"åvµØp0°uçyiâKÁ7÷Z÷h?ÔêìÓúOëþ­.i?½+ª¿¯¾öéôÛRùQÿULu¨·þõÑC~C90E5¡»c¾ï~(¡*¯¿ÿóøÑ'NÄ	ðfHd¼fEA"³Ayïµìå	O¬¼!GV½¢¤UÁõy­¾>ñ¹Wwï=ïmáæè@Afs Æ½F´uqø	Ò=í.ù¯£°ës_0âFKü%%%Qü¹»»3wDüÁÈ)kSpmÇ±+ìQ^ùyò¶j'ðmÞÕ_ÒSÓ¯­kQmdn(G]ô æPBýwÌ!()è	!âFüý¬8á]vÃëÂ£¼fåW*¿RÎP-^y÷º8õë¸¤äPüUÏ[Ïlì¼sD@Röí£Iz»YÜã?a®(½Z¼xqff¦ê[§)ý7¬ÂÂÂ4¦0#<ÏÍÍÍÚÚÚÜÜ|Ú´ixL!þÌz>`$âÙ¥Y¯xÁVînæ+*æÎ¯NïøÛ:nê©jèiX½¯ìÍâ*¼z µ]ÊµìSÁgB3#óÔëâð]nyÚ]¢ø;°ð|ôédÃÎ¿`ÁÕ·4®ÿ|ÃROçræÎ«¾Pµd''§ÀÀ@Dù©X,Þó^)£?£ø3n|Í¯¶É1»IaËë)ÓÍOi(ûï!¿f6²~íÈlä'<Ææ@ÐFmWð ì[ßï¾N¡	ÒÆ¼ øãs®fa-ûKÜÞØ9$ÆiãË/gnéLãFÁµ§ñ-ö[x<Þ	ð@ü!þÀPüWùQíQüUræ7+d=¿cdîÞÁl¬]âÊläÆ%c@[sçÊwö½sïw2sÌ§KQF­¡âÃ+C2Dx&ª/¹¨¨hÎ942wî­ÛHûÒ¥K)Åìììh¼Ïø£Ù222reñG_mmmëëë5f°±±ñòòÂ+n"ñ§:ªÇüÁÔÙ­¾ôZÅ:EyrÏï`È%íMîÍUó¥;WÙÈÞë_1Õ¦-ÇtÞPÆ¬G7ÇKK¹Ì!HuÂäë95Æ¸µk×:;;òÉ'ê]\2sssS÷vÌ_~~þ¼yód2E¤*éôÇ_ZZÚêÕ«5fÈÉÉ6mÚ)SèFGGgggã2âøSÕcþàÍSíö´£¥zq[Ãó¦þªîÅÛ7±­àAYø¡ÚÙ¾Â®nlù=X,µWyy¹úDkkkfD$M4IgÃi |t©ÆÎ±¨ZÅ% ö333¨®_¿×ÈXãÏ4 þ¸¬õûÎÖã?½cH¨n(ÇldïõÇæÀ1ÏzýãâGªø>|_Öèût×ª:»æôÇ_vv6µÎë¸éÜòGjkkmmmõ,¹°°pÊ)x>þ´÷ð677Ï1cêÔ©?0øñ~h(ûoa×ÑùÇõT5p3VïÃæÀ1¨*¯ÁÓîRÀ¦p¿Îßù­¹¾À@ãoüøñýMÛN8Ñÿø#ª)ãÆÓ¸^ ?±XL±Û"þº8¾#pzÇÄ7Í¬_;¾¾¡6|®0êä¿ebaV4þ\$<<|Þ¼yÆ_ii©­­- Ï5«Çü½þßihÎ9ª)ÎÎÎ>>>Ì¦üüü]ñküM:õgzéüÄè!îæ·2Dç¦VøYEùØ5ïäéC¶®JªÊOú;n(7Fà0øãp8vvvæææsª³õðáààN#Ë-ÐSMa³ÙöööVVVfffÖÖÖöì¡ Äd¬ñW]]ÍØ¡:@_DDâ¤Íÿ«SøHgÎÈåÆ½ûLÊíÒ¸¡rs D·â`ãOÅXNìEüAÇHåÔqÇõþú9ïVë0¡§T7£d® MQHi÷â`ãÏ¨!þFnò>ÚìûØçµäÔµ0Ë'ý¹´(·CÌî|Å«ÃH---ÍÍÍ0PÒî®b/[C/]ôëÏ;Ûó?ë,:À|+¨5íUÊsärÕÛk;o&bs ââääÄó'ÉÔ]»v-âD&å´7n5`pÜzÁïÒ'Í¶¹©âËg´ÕoPMò»÷fÌæÀúw0ÙûOð®CüâoÀÆG©QTTD#666µµµ4BÓ0 r^wÈW+^ôÞ­gÑÕ/;Î¿ÝuiiwÈ^õé=nÍUMÙ×z(°ò·«Ê¬ÿÖ¸Þ%iâà8bXÏêt8ñá?HCc'V;Yü©Î_°`0wÄ½Áøëlú¬3`X±åÂýzTà½¢Ãë7¼ïi?$Nãë¼§ª¡#8¦îý/J>¿êwsÑ# æZ¢ÃùÞ;GñgÀi(z^ÕCVVVzíííÌ&Àúúú¤¤$lù´bKÀEß:âwÜ0k8¿qüÇ-_ø°~ùaõß>9Ðuë>6ÄðOtX÷h<®B¬v²ø³··WçÇÜ+ÇüÁ©ièôxâ¸%à¨Ç®ÑÅR©Ñ¶GSÅÿEÏ±¢DÅ½x¹¡_6¶yâr£Á£æ8?¬xóñGæÎkffFå×ÞÞ®xuå¿ÅÅßø3jMlþ£.G¬ØpÎû«/¥|æÓ2iGkÝGìê%2)¶]N.wE¥4o?YùÛUåSÒ %GÆ`Í þ`¬ÇñBü5J½Ð¨"Å¿wû¤Îûï=È¨ôÔ4WÍç¶ì3ö»w*âª6ßuïñò?þD_;chVâ?9Ë·Þ$1âïìÉ/ÙvôýÿØÆò_uµÃú&2ð¹¡oW½¾¡7ëDü©ÇÆù¼ú¿EüÁ0Å_Õ¾²ÿVÆßÏrÞÚ´ÇoÝß¤´¦tß¶ÑL[ÚæZ³Ð¹|ÒßÊIêZ°ZøCüÁ°Øsò[¿o¾ë»öÕ³>^ã~ÚóÜwnGß[wkã¾näe<oà	z°®ÛÅÜP®bªµ`ë¡@ñ?Ä±â¼÷>ÞzäÓglùt½ß3§¹-gé!q4·¸%øv!à·÷ß£qBÓ±Þ¤äPüUÏ[O!ÈlÄåøCüÁh(ù¯¦=®<tÜpüðw/³æ´×nÕM(ä6_(Ø~ø¾ý¦HÊÁèûì6²:ì+HSÿ17£"Ä9"Ê¥Q5AîY¯ÉdßÃ:Äâo,jÊxKô2T¡vçÖÔ?s|¢ç)L¿õÁæ('÷¸SAÙ÷UãÁá&Í¬_;2;o&âþó+Þ¼`k|Ý·íOv<]þ¸%«¿1§-pFëÌÙ¾;nÙ×çÿ'ß÷G·¤Å²Úy·¾Íuß'<ûBpýî³!ÏR³jÃÙØ°z_Ù/Ö,tæÆ¤õÁÍÞýtEL¬Úí[Òñìó,¬@ü!þÆÁáeUë_þ'ÅßyïE¿á_vú¬ëùJxv#=Êÿümþ?ö/QN¼qX¾ça¸ôÅ#YS¥×®¾¨òjnx^ï4Açý÷p¦È`6²÷úWLu ¡iË1ÑÕÃmÕvæ=ã<¤àó)úòHÞ¦ ²#¯QóçýÍu#ú!þ`¸QÌ	O|,ã5+·üùîgSêÉjryg+¥eÍO	(<ç,8dOsR¾z ººG®çþ5IîýªÜòðØ"Á%n»IÆ"# §ªAuC9úJã¦·9°G&nën©ä¿àfg²ï%6GÕ[q:àåS®Ç¶îÍùèó,ûéÞ¹d÷ÓGó7ûï¦æó+Ú8oUà/´(¾â#f177GüÁpë¾éÅwÿ£2þ<·òw-gôÿ¹ònS/-&ÉÇ_ì8!ºèªÜ^¸÷]Þö9ôô£,ïÃ½oØþù×1Á7²(5î CÙØ´å³9½×ôoìsÕ%Ï(é(Ñâê®ETP´~ñt;.ßñî¦ÇÜ³?ðÈýRJâ/©ñ=rN¡EIåÿAÉH©GÕ¨ÚíK?wtüÄ	±k'|Ú%ÝõDdRy³¬úäi¼8!Hu¦3pWæ×o6ïØ¾æäªµA^Û¼c¯	:FÒ<ÒÊ<êHEJ=UãÁÌæÀÚ%®Á1#¹9°G&n5Vt´?¡êJnúZ-¤ü$Suy(é¨ç¨Æ>Ï²§Â£Î£¤£G©ü(Îè)ôD&éh9"©p0¿Lvë'Ïí¤Xê±ûé*B¼Cñø3zR©¼Í7`ø«SøaÏ<Ñ°Ó;Zjêâo?<ê¹jsøªÏnxy!zÏîÊÝËÛw-øXxÎYy!Ó¥Ù²¦Jtá`ÈEbÕå¹9I:ê(ÝTû^©±(ì(ï(ò(õèQÊ>?J@¦ê(i9#¹ã~çó%ûè»[½CÌÁ»?SÀdÜHÇ/eþwNxXIËùÈ-ãþî¤	óÒFíú¬²gºFT]ØÅz.qIUë¡@Íª­tLÒ1[é´÷½RÏ1I§sC=Qµïutþí¸È3 þ¦FÔÓÎÊ4`Mú]bÂG<±]>´ñJ!xØ?¹´Æ"?9õ$êòäW§PÒðºC¾¢hfW2=e,¿(Âôìx=ö|óîöÛïýzhcò¼/îýÍ#íããy[(é­tÚû^ý	Ä þ¦>é³m$ÊáøCÔo.§35õt¿îÂ(qBP÷ÃÊM'>æïZÀÛüfW2M1]É!Áìx¥¯zv¼ªo¥>)`6þ|þÈøÄâ$rÚ·0¸eÌ½öì<Ï½6ÜvÞ.nbóû¿yg«´2OW²ö&C*È7µÉ°·3$cétÁ;^%u-¯o(7e©é];ñ?ÄâïõðEmXÆüÑÚ!ÿúÁKY	+ZÁ¥¹ÉBPß&CÎJîçºÞÎ`¥á3$ä)sC¹êyë_þÇLãÚ?@ü!þL%=oÀàþíwæðDIîý7õÇj)2øÔ^ÚO|,øê=æìÎS«ë.­/¹íþüÞ¡Çîçù~_äRzÌKòOU7£4êÍ?@ü!þL.þ:[>ë¾Lz'âú<Q4þpU®ØC!oð=Ep)ôù;2N[~êÁâ]qíÝ³oß?÷§çþ^tåð½üK¦tV2séÖC¬_;¾ÙÍ5ü2zi:ÐËG/O¤WÄâÏ¤ìxº<ª&Èþ©T®óL~·pÄ.eÒ¯³5nlg%3k¸R²~ùaóö¼Ø4O02?^¾>·^züC?Äßè#)ª9[e_3ä¹­¼7øçê¿ÉÑçÛ·ÞÙöß>»§ß]íýÝç>½plWÜ>ÿßØ¥LTjÜ(y×QuêÉ0Wn=XùÛUõîàEIêpÿ@ü!þ`prÏ¸¢ó©êöR&<AêAûMÃqàu¡ö©'Lú¬íåW1TuÇÔ½ÿEéÏçS¶ì<ËOÊK¤Cþ$¹÷©GrPî¸@ü!þLåQ`©ÛûÆaÔXR¹tø~Öh¸bDÎ.,Ífnü«R~õsJ2u¡êäÑÐæí')Ë~±°áßÆK[¹C¶J2¢8D·ù74v¬¶ÿâñgôê¬íK"«9Þ7ùT¡ëé_t×§@Â£¤«áQ=ã<Lm®½JË¼Z~Ì·hÇ¨½	£¦¡9@ð½u·T!ÈnÍKH]È©gNI¦òS<´ª$I]Kw(st`ÕÛkØûO»P®Ð£$ñw¿oØ¡QþÅÄâÏèQíQ¨ÑÅ_qôM/ÜO©ÞhT.mëniÕiï=_òú3Þ¥Ï×ÐD¿âÝÔ|T~´XZNn[ºQDñÓKI/Ùp»ïýª¡qÕGæ®'RnWWTJóöåSOúÓ¦#]·îÓDÅ÷á#ºïdà_@ü!þÞçYö"©FZ½Ãn¥zsÏþbnCú×=Gã»®8¿jOçI¯&p³×ÞB0+¯Ù/üÞº[Ì=Er:uv¡êdõ»PþxJrBÐ0]ªF[ÚæZ³Ð¹ì«ÿ²s<¦ôÿé=U]nq<Ã"?](ïåéºï°¬Û)éÂ«üËNëÆ9@ÐçÊSæL¯Ï<+©iè4á?y/U#»nÝoQ1Õa½dð¸?ÅÞÕøCü]AeG¾­8­ø÷n_ÁÝOW´?ÁÑ©²®#.uðìãÛc>ØuüRkLÞ0_ªF«¼¡Ü_6þ|~í×6ïÐîÂÄ þ0dºz¸T¥¯¶ü|ýÌiAèOÇìõN£;ÁçÊÓGOëÛ¸¢±»FôR5ÒVnGpLãzò)KÊñbÓC9Ä þ à75VûçTXÞE¿êîJ¥¬Jå/Ym»N>æ»û~8ò,+¯'ÀÉ¡?íB.U#H)9ì½þU¿ûøåü©zÞzÎñ`7ú*ÏÞàè=@ü!þÆ¬.ùéòÊÝ¾·¾)¿¥¼MÖ"IQ9'0<Bð½u·¾<r9¢ àe+Ö¾.ìÏ¥jncÏ#ºÎû7ýk[ÙÿÏúµcóö7ûysÛkþfg?þçowßôRÈp`+Pü±Ùì)S¦¨¾ÍÌÌ<y²¹¹ùÌ3ÕçÔ~¨?SüÂª>IDAT&jOV®ø÷1ò GçµX3CÖSnÜ÷ý¦HÊÁ¸dÖKVÖÌÞªKÕÜ»Jñ'ººGyJòáeÊSOÜÿÜ¹ý¯¾Óòîlöª:N?±ktïJIÇVRY2»å¼vZ8þ"Ö0Hü¥¦¦ÚØØüìg?þbW¯*¯åæçç·råJõµêÏÄé|¸IR%¯ãïêeytüjºübª<6_òRZÚÛ È©VäÕõ:Ô+*[û¸#0WØëÀë6±õÌàÙgkvÞ]±=æTPvôýÊº¼$ÊÛeµ%ÔÝ1W;|Ö¾q)gÙï:ÖÌîÚüëÛ)ïºoz1§$ã/	®Äfó])¯ØwìäñSþ?cãHüÙÙÙ¨ÇµµµL¦Ü'Õ·ê|¨?S4âïèÑ£1`'â¢#SaÙßÏ­÷m>[òMìËÞª¸ó±½µþ±-gb[ÏÄõ6tãímïãr»R¯^NKÖùuÿªôèübimô6dÞÓÛð(<öþí^ÛÁÑgNG9ï½µäÓï9÷ùÁÛG¿¿~3ïÆÁºsÿ_Ær×ß¾ÿÒú/þñÏMy:YÃv/t?(r½Úù/zÿT|¸gïw;?ùáÖ¬6#øÄ®_K-þÌÍÍuë|¨?S4âïþýû0NÒ¨çÒøáxß,º&á	eþÉÒÒ¦ÑþÄRO÷ÐÜ!eµèJdÏktÙÒäCRråô2È®gÊB2z.?ùÝ×3ÈÏ$õq?3Iúp!EßO§áz¦_^9Ð_§ó¯~5ÈÒÊt¯«ÒüZÝ+Y5Ð«ÐÛÄáI¸üá~t7µ¶]¨ùÛÒÏgÍýâkép^b¦à­^¡óH°ôÐ¸ÿ)Ä þú?333Õ¸úlÚõgvûX~>E«ÜíóüÒCyÂ¬§~s¹÷ÖÝÚsðÞ­°¼¢§u=:÷ÓDýw¡­`ëÛ)OCzúò%ÊôÏå×èRåçô:ôÙ¾Çîê:ô¦Îèãw áf6ý¶=xºwîéü×g¼M_+/õ"vì§q:ñÞÓ¿'Åbf¿-«Ï¦ýP¦ þF­¸¤ãÙÀ¬â(ö©ëi!'K'psôôFa5VûàõËÀ]F"ÓwH(m|Em[LCY»7»¿ìXÄûlòönûÎo|u[Êæâý¦«W¯¾|ù2ÐWGGGõÙ´êÏÄß¨Õ*jhÅß÷¡»xbPÙ¬öáÃÜ#Ê9X3"J|*ùú¶¼#8tâOVýB&ÜvkL6þRSS'Mdff6yòäôôtõ´êÏÄß¨%K©ÿ$~÷ø÷ðÄ®l;é=ZFè[5Ó[ý%_ßìVÆøj²d_?üVTüÄiPGZõ`/« ì×;å¼Â[¿â¯cíiÞx¬@ü!þ?ã£q¦2jÆÐûâñ?AIcôLÄ þøt)Düâñgr$²¾¯¡kP^ÚCÛÊÃZ7º¾_16Düâñgr¸Â>.;äCtÖºÑà®ýLii¶8öü@îÝÊÛ»ðDdz¼ñødò>®;ä@µnt)bD!Ø%ôY7ÐA´×âÏ'Ò «-ÁÛ?Á5;ïÁô??J¡Û'V¹Û× C#¢¼Uñø0ÁÌÊkýgð=ÂFøî;xâñ`ÊFó)ÃÝÙÅ]A±#9Hê[ñÄâ`, ý¦HÜS?+j:qs9@ü!þ`ÌÑ>eØçÊS?[!¸dÃmÜS?+Øm3EnDí~a 8*?èú"Cx`ðíÂ¿:ä[ÜW?pöÏP)ò £Æ°-j´(ª±ô3`à	pg@ü!þÀ êÚo±)=)þ°þñøÑEåÄ þ0&B00<hDüâñ£ú"Ë·Þ9òìÞ£ê®nÄ þ0&BÐ+àÉí1kvÞ¥ÌxÞÐÆ!þñøWßÌû>±ìàÙÇlÚ~ø~À¼¬¼Æ~)øÄâXQ9'<®d¯wý¦HI7¢õ2Ly,âÏ?ôyMC'Ö þ`¬T§o?|ùÖ;»N>¤¬¬ëJåÌ·J?r=ñê:áù4ú?0ÌxÞ@·fç]¼¯Ï<ºz»pÙ¨úfj·oËå`Ö þ`Rxê<×Ã|zÅöÍûï1ñ'Ê?Ø%I°ñøS¿"0<¿¾çuáêjAÜ±?0A/[ÔSíöÍÊk¤)X3øCüiò¹òÔyÿ½ayqÉ,'÷8ê?¬@ü!þÀdQóº'âo¯wZnqV þ`âpg@ü!þñøCüâñøÄâñ?@ü!þøCü þñøàÛq#9àÒøCüÀÓÄæSäâñ?ÄâñøCü þ?ÄâñøCü þ?ÄâñøCüâñøCü þ?Äâoûúë¯SFËEâï©®®¦þ;0RªªªÐøÄ þñ?@ü þñ?³þÅÌ3||ÔgÐ~V`` ÖÒûÌÌÌÒÒröìÙþþþXuøüPTÿV&%''O>]õ±§(?ïó¢¢¢yóæùúúbíâLùCQXXH:g@ùÁXx«úoêÔ©XøÓÿP$æææÚ3 ü`,¼ÏÕYXX`íâLÿC155U»»;ÖöûQQÑ¬Y³°öñ¦ü¡(BCC'N¢>ß¶mÛlmm±ÆÀßç*ÌA3fÌÂÚÄÚ¢ºñãÇ;::&$$¨Ï ÚÛëéééíí&ù>W7nÜ8õ`:ÌL&;vìÃÑAuÍ3gÎõ¦÷>ÇZÄ­E§§ç'|¢gEa½i¿Ï0V>	µÜÝÝUGJêûñcåCÍfÛØØTWW÷6Ç=vss3ÖðûñcåCäçç;88è!>>~õêÕX`ÚïsÄ þøÄ þñ?@üâøÄ þøÄ þñ?@üâ&>>ÞÎÎÎÊÊÊÜÜ|üøñk×®-**ÞÁ@üÀHýqãÆkÿ!þðf¼õÖ[ÔaômuuµMY¶lVâL¥úv¾ÚÚZbiiùúWbccgÍenn>sæÌõ%Ð·666ôÍ@³©?8ölz¶páB¥¾Ì>Àáp6mÚ4nÜ8zÈÚÚÚÕÕÇãá%@üá-[FFuåää-É4ÿ!xÅÌÌL¿pFFóhnnnoeffjìMVmMT?=KpppÐXÂ¶mÛð þÀpl6öìÙêGûQ`kÄ££cgg'ª£éômbb"§¦¦ÒøÊ+/_Nß:;;Åbæ!+++íøÓ³&«««iÂT	øÃEDDPÏ3Yfii¬jÌ·4Â4"ó-h,¢þÎµêñ§g	LN6222R à@üÀH$QQQ, ä¢ðÒ5ÕÊDõGÕõöDñ§g	%%%[%é×ÃËøÃY[[SW©NÅ 2L;ïT3477«o³²²¢oÅb±öMzÌIÄzâOÏÙÙÙû÷ïgv7O</â·iÓ&ªwÞy§¤¤¾¥VÛ±cM9s¦z¨ÙÙÙ±Ùl@°zõjúÖÉÉyi2WWWJÆøøx5kósÌ»»;=HãS§NÕ?=K`ùV¼ÚHãxÉ`¸êêê'jïP5KKKÕCT`ªKÃdddh<1<<yHûlßcÇiÇ%¬Rã¡6à%@üÀ °X¬õë×O0<;;;õ+ù1Õ6öl33³9sæ¤¦¦ª?=66&ÒC'O¾xñ¢úC´æYôÆ2ûÇÛ±cÇ¤IhfúõÜÜÜD"^/Äç?¸âødñÖâøÄ þñ?@üâ?@üâøÄ þñÆúï.C»üaýýy<ÞÚµk-,,&LàââÒÜÜLÅb±Î¿(>>ÞüfJaaá¼yóô,¿´´ÔÎÎ?yòd??¿û»ñ0gÔËwsskll¤LFqfkkKãÔvöööÚ33ÙGh²|ùò¤¤¤ÞNK6mZDD-¼¶¶2ñÂ?@üâï-ßÊÊJ5NfiiI#¡¡¡®®®z~f$??ÿwÞÑ_ÞÞÞªogÏøÄfüÑC.6mÆ8Í^¼x±¹¹ù¢E8öÌ)//§f¢éÌÄÌÌÌ9sæÐ&NH¡¦½fÿ¬Ù¬Y³RSSõüªÔ|Ì¸X,¦oiÄÙÙ9<<{f-ªë4úôêêê7µø®øëí?¡DãV!!!L¢mÚ´Ifå»¸¸PeddÐ666ÉÉÉ49~üxí%P1Q¨êÖ®]ëççGË§'Òò_ÞÞÞòÔêúé<É>B¿y~~þô¯ÕÞaÄ þÀtâOÏCªjêã&L`Æé«zº©fÐXjë =QÏ/ ¾ª7'''*?D¢ÿ÷ooo_½z5UÚÔ©S£¢¢-ô[1íH¤¾¤@Ô~"ýôôtíS@ø±«ò¨·åèrÐËË¢ÍÆÆFã8<ÕÌåqãÆåææöóo)--<y²öt¦Õegg3ý´OQ7kÖ,¥úRúñ?[ñ7~üxfÅÅYñ'­­­¶¶¶ÑÑÑÕÕÕ:ãO[W¯^ÕÙs:EFF:99i/D;þìììÒÓÓZ§hpwwW?áqîÜ¹?@üÀØ¿60;U)Ôùëm!»ví¢´´4777f¢¥¥eii©X,¦):óËÆÆ&>>^ñï£ôôüþ4'5°X,Jºf"ý8¦üBBB4ÌÌÌTí§Ëµ)¥gll¬âÕ©¾S§Ne~â`Äñ§çñÇf³-ZDµ´xñbõ³[>¥Õ)S.]ÚÙÙÉL¦>?~¼¿¿¿ÎG6sæL333æä=?joîÜ¹4ÛôéÓ#""TôZXXPe®]»V×-¡222qõS@tþþô0Ë§?áâÅýfÄ þñøÄ þñ?@üâøÄôêÿ`¯"|röJÏIEND®B`
